# Exome sequencing of 20,791 cases of type 2 diabetes and 24,440 controls

**DOI:** 10.1038/s41586-019-1231-2

**Published:** 2019-05-22

**Authors:** Jason Flannick, Josep M. Mercader, Christian Fuchsberger, Miriam S. Udler, Anubha Mahajan, Jennifer Wessel, Tanya M. Teslovich, Lizz Caulkins, Ryan Koesterer, Francisco Barajas-Olmos, Thomas W. Blackwell, Eric Boerwinkle, Jennifer A. Brody, Federico Centeno-Cruz, Ling Chen, Siying Chen, Cecilia Contreras-Cubas, Emilio Córdova, Adolfo Correa, Maria Cortes, Ralph A. DeFronzo, Lawrence Dolan, Kimberly L. Drews, Amanda Elliott, James S. Floyd, Stacey Gabriel, Maria Eugenia Garay-Sevilla, Humberto García-Ortiz, Myron Gross, Sohee Han, Nancy L. Heard-Costa, Anne U. Jackson, Marit E. Jørgensen, Hyun Min Kang, Megan Kelsey, Bong-Jo Kim, Heikki A. Koistinen, Johanna Kuusisto, Joseph B. Leader, Allan Linneberg, Ching-Ti Liu, Jianjun Liu, Valeriya Lyssenko, Alisa K. Manning, Anthony Marcketta, Juan Manuel Malacara-Hernandez, Angélica Martínez-Hernández, Karen Matsuo, Elizabeth Mayer-Davis, Elvia Mendoza-Caamal, Karen L. Mohlke, Alanna C. Morrison, Anne Ndungu, Maggie C. Y. Ng, Colm O’Dushlaine, Anthony J. Payne, Catherine Pihoker, Broad Genomics Platform, Wendy S. Post, Michael Preuss, Bruce M. Psaty, Ramachandran S. Vasan, N. William Rayner, Alexander P. Reiner, Cristina Revilla-Monsalve, Neil R. Robertson, Nicola Santoro, Claudia Schurmann, Wing Yee So, Xavier Soberón, Heather M. Stringham, Tim M. Strom, Claudia H. T. Tam, Farook Thameem, Brian Tomlinson, Jason M. Torres, Russell P. Tracy, Rob M. van Dam, Marijana Vujkovic, Shuai Wang, Ryan P. Welch, Daniel R. Witte, Tien-Yin Wong, Gil Atzmon, Nir Barzilai, John Blangero, Lori L. Bonnycastle, Donald W. Bowden, John C. Chambers, Edmund Chan, Ching-Yu Cheng, Yoon Shin Cho, Francis S. Collins, Paul S. de Vries, Ravindranath Duggirala, Benjamin Glaser, Clicerio Gonzalez, Ma Elena Gonzalez, Leif Groop, Jaspal Singh Kooner, Soo Heon Kwak, Markku Laakso, Donna M. Lehman, Peter Nilsson, Timothy D. Spector, E. Shyong Tai, Tiinamaija Tuomi, Jaakko Tuomilehto, James G. Wilson, Carlos A. Aguilar-Salinas, Erwin Bottinger, Brian Burke, David J. Carey, Juliana C. N. Chan, Josée Dupuis, Philippe Frossard, Susan R. Heckbert, Mi Yeong Hwang, Young Jin Kim, H. Lester Kirchner, Jong-Young Lee, Juyoung Lee, Ruth J. F. Loos, Ronald C. W. Ma, Andrew D. Morris, Christopher J. O’Donnell, Colin N. A. Palmer, James Pankow, Kyong Soo Park, Asif Rasheed, Danish Saleheen, Xueling Sim, Kerrin S. Small, Yik Ying Teo, Christopher Haiman, Craig L. Hanis, Brian E. Henderson, Lorena Orozco, Teresa Tusié-Luna, Frederick E. Dewey, Aris Baras, Christian Gieger, Thomas Meitinger, Konstantin Strauch, Leslie Lange, Niels Grarup, Torben Hansen, Oluf Pedersen, Philip Zeitler, Dana Dabelea, Goncalo Abecasis, Graeme I. Bell, Nancy J. Cox, Mark Seielstad, Rob Sladek, James B. Meigs, Steve S. Rich, Jerome I. Rotter, DiscovEHR Collaboration, CHARGE, LuCamp, ProDiGY, GoT2D, ESP, SIGMA-T2D, T2D-GENES, AMP-T2D-GENES, David Altshuler, Noël P. Burtt, Laura J. Scott, Andrew P. Morris, Jose C. Florez, Mark I. McCarthy, Michael Boehnke

**Affiliations:** 1 Program in Metabolism, Broad Institute, Cambridge, MA USA; 2 Division of Genetics and Genomics, Boston Children’s Hospital, Boston, MA USA; 3 Department of Pediatrics, Harvard Medical School, Boston, MA USA; 4 Program in Medical & Population Genetics, Broad Institute, Cambridge, MA USA; 5 Center for Genomic Medicine, Massachusetts General Hospital, Boston, MA USA; 6 Diabetes Research Center (Diabetes Unit), Massachusetts General Hospital, Boston, MA USA; 7 Department of Biostatistics, University of Michigan, Ann Arbor, MI USA; 8 Institute for Biomedicine, Eurac Research, Bolzano, Italy; 9 Center for Statistical Genetics, University of Michigan, Ann Arbor, MI USA; 10 Wellcome Centre for Human Genetics, Nuffield Department of Medicine, University of Oxford, Oxford, UK; 11 Oxford Centre for Diabetes, Endocrinology and Metabolism, Radcliffe Department of Medicine, University of Oxford, Oxford, UK; 12 Department of Epidemiology, Fairbanks School of Public Health, Indiana University, Indianapolis, IN USA; 13 Department of Medicine, School of Medicine, Indiana University, Indianapolis, IN USA; 14 Diabetes Translational Research Center, Indiana University, Indianapolis, IN USA; 15 Regeneron Genetics Center, Regeneron Pharmaceuticals, Tarrytown, NY USA; 16 Instituto Nacional de Medicina Genómica, Mexico City, Mexico; 17 Human Genetics Center, Department of Epidemiology Human Genetics and Environmental Sciences, School of Public Health, The University of Texas Health Science Center at Houston, Houston, TX USA; 18 Human Genome Sequencing Center, Baylor College of Medicine, Houston, TX USA; 19 Cardiovascular Research Unit, Department of Medicine, University of Washington, Seattle, WA USA; 20 Department of Medicine, University of Mississippi Medical Center, Jackson, MS USA; 21 Broad Institute of MIT and Harvard, Cambridge, MA USA; 22 Department of Medicine, University of Texas Health Science Center, San Antonio, TX USA; 23 Cincinnati Children’s Hospital Medical Center, Cincinnati, OH USA; 24 Biostatistics Center, George Washington University, Rockville, MD USA; 25 Department of Medicine and Epidemiology, University of Washington, Seattle, WA USA; 26 Department of Medicine, The University of Chicago, Chicago, IL USA; 27 Department of Human Genetics, The University of Chicago, Chicago, IL USA; 28 Department of Laboratory Medicine and Pathology, University of Minnesota, Minneapolis, MN USA; 29 Division of Genome Research, Center for Genome Science, National Institute of Health, Chungcheongbuk-do, South Korea; 30 Department of Neurology, Boston University School of Medicine, Boston, MA USA; 31 National Heart Lung and Blood Institute’s Framingham Heart Study, Framingham, MA USA; 32 Steno Diabetes Center Copenhagen, Gentofte, Denmark; 33 National Institute of Public Health, University of Southern Denmark, Copenhagen, Denmark; 34 Greenland Centre for Health Research, University of Greenland, Nuuk, Greenland; 35 Department of Public Health Solutions, National Institute for Health and Welfare, Helsinki, Finland; 36 University of Helsinki and Department of Medicine, Helsinki University Central Hospital, Helsinki, Finland; 37 Minerva Foundation Institute for Medical Research, Helsinki, Finland; 38 Institute of Clinical Medicine, Internal Medicine, University of Eastern Finland, Kuopio, Finland; 39 Department of Medicin, Kuopio University Hospital, Kuopio, Finland; 40 Geisinger Health System, Danville, PA USA; 41 Department of Clinical Medicine, Faculty of Health and Medical Sciences, University of Copenhagen, Copenhagen, Denmark; 42 Center for Clinical Research and Prevention, Bispebjerg and Frederiksberg Hospital, Copenhagen, Denmark; 43 Department of Clinical Experimental Research, Rigshospitalet, Copenhagen, Denmark; 44 Department of Biostatistics, Boston University School of Public Health, Boston, MA USA; 45 Genome Institute of Singapore, Agency for Science Technology and Research, Singapore, Singapore; 46 Department of Medicine, Yong Loo Lin School of Medicine, National University of Singapore, National University Health System, Singapore, Singapore; 47 Saw Swee Hock School of Public Health, National University of Singapore, Singapore, Singapore; 48 Department of Clinical Sciences, Diabetes and Endocrinology, Lund University Diabetes Centre, Malmö, Sweden; 49 Department of Clinical Science, University of Bergen, Bergen, Norway; 50 Department of Medicine, Harvard Medical School, Boston, MA USA; 51 Clinical and Translational Epidemiology Unit, Massachusetts General Hospital, Harvard University, Boston, MA USA; 52 University of North Carolina Chapel Hill, Chapel Hill, NC USA; 53 Department of Genetics, University of North Carolina Chapel Hill, Chapel Hill, NC USA; 54 Human Genetics Center, Department of Epidemiology Human Genetics and Environmental Sciences, School of Public Health, The University of Texas Health Science Center at Houston, Houston, TX USA; 55 Center for Diabetes Research, Wake Forest School of Medicine, Winston-Salem, NC USA; 56 Center for Genomics and Personalized Medicine Research, Wake Forest School of Medicine, Winston-Salem, NC USA; 57 Department of Biochemistry, Wake Forest School of Medicine, Winston-Salem, NC USA; 58 Seattle Children’s Hospital, Seattle, WA USA; 60 Division of Cardiology, Department of Medicine, Johns Hopkins University, Baltimore, MD USA; 61 Charles R. Bronfman Institute of Personalized Medicine, Mount Sinai School of Medicine, New York, NY USA; 62 Cardiovascular Health Research Unit, University of Washington, Seattle, WA USA; 63 Kaiser Permanente Washington Health Research Institute, Seattle, WA USA; 64 Department of Medicine, University of Washington, Seattle, WA USA; 65 Department of Epidemiology, University of Washington, Seattle, WA USA; 66 Department of Health Services, University of Washington, Seattle, WA USA; 67 Preventive Medicine & Epidemiology, Medicine, Boston University School of Medicine, Boston, MA USA; 68 Department of Human Genetics, Wellcome Trust Sanger Institute, Hinxton, UK; 69 Department of Epidemiology, University of Washington, Seattle, WA USA; 70 Instituto Mexicano del Seguro Social SXXI, Mexico City, Mexico; 71 Department of Pediatrics, Yale University, New Haven, CT USA; 72 Department of Medicine and Therapeutics, The Chinese University of Hong Kong, Hong Kong, China; 73 Li Ka Shing Institute of Health Sciences, The Chinese University of Hong Kong, Hong Kong, China; 74 Hong Kong Institute of Diabetes and Obesity, The Chinese University of Hong Kong, Hong Kong, China; 75 Institute of Human Genetics, Technische Universität München, Munich, Germany; 76 Institute of Human Genetics, Helmholtz Zentrum München, German Research Center for Environmental Health, Neuherberg, Germany; 77 Health Science Center, Department of Biochemistry, Faculty of Medicine, Kuwait University, Safat, Kuwait; 78 Department of Pathology and Laboratory Medicine, The Robert Larner M.D. College of Medicine, University of Vermont, Burlington, VT USA; 79 Department of Biochemistry, The Robert Larner M.D. College of Medicine, University of Vermont, Burlington, VT USA; 80 Department of Nutrition, Harvard School of Public Health, Boston, MA USA; 81 Department of Biostatistics and Epidemiology, University of Pennsylvania, Philadelphia, PA USA; 82 Department of Public Health, Aarhus University, Aarhus, Denmark; 83 Danish Diabetes Academy, Odense, Denmark; 84 Singapore Eye Research Institute, Singapore National Eye Centre, Singapore, Singapore; 85 Duke-NUS Medical School Singapore, Singapore, Singapore; 86 Department of Ophthalmology, Yong Loo Lin School of Medicine, National University of Singapore, National University Health System, Singapore, Singapore; 87 Department of Medicine, Albert Einstein College of Medicine, New York, NY USA; 88 Faculty of Natural Science, University of Haifa, Haifa, Israel; 89 Department of Genetics, Albert Einstein College of Medicine, New York, NY USA; 90 Department of Human Genetics, University of Texas Rio Grande Valley, Edinburg, TX USA; 91 South Texas Diabetes and Obesity Institute, Brownsville, TX USA; 92 Medical Genomics and Metabolic Genetics Branch, National Human Genome Research Institute, National Institutes of Health, Bethesda, MD USA; 93 Department of Epidemiology and Biostatistics, Imperial College London, London, UK; 94 Department of Cardiology, Ealing Hospital NHS Trust, Southall, UK; 95 Imperial College Healthcare NHS Trust, Imperial College London, London, UK; 96 Ophthalmology & Visual Sciences Academic Clinical Program (Eye ACP), Duke-NUS Medical School, Singapore, Singapore; 97 Department of Biomedical Science, Hallym University, Chuncheon, South Korea; 98 Endocrinology and Metabolism Service, Hadassah-Hebrew University Medical Center, Jerusalem, Israel; 99 Unidad de Diabetes y Riesgo Cardiovascular, Instituto Nacional de Salud Pública, Cuernavaca, Mexico; 100 Centro de Estudios en Diabetes, Mexico City, Mexico; 101 Institute for Molecular Genetics Finland, University of Helsinki, Helsinki, Finland; 102 National Heart and Lung Institute, Cardiovascular Sciences, Imperial College London, London, UK; 103 Department of Internal Medicine, Seoul National University Hospital, Seoul, South Korea; 104 Department of Clinical Sciences, Medicine, Lund University, Malmö, Sweden; 105 Department of Twin Research and Genetic Epidemiology, King’s College London, London, UK; 106 Folkhälsan Research Centre, Helsinki, Finland; 107 Department of Endocrinology, Abdominal Centre, Helsinki University Hospital, Helsinki, Finland; 108 Research Programs Unit, Diabetes and Obesity, University of Helsinki, Helsinki, Finland; 109 Diabetes Prevention Unit, National Institute for Health and Welfare, Helsinki, Finland; 110 Center for Vascular Prevention, Danube University Krems, Krems, Austria; 111 Diabetes Research Group, King Abdulaziz University, Jeddah, Saudi Arabia; 112 Instituto de Investigacion Sanitaria del Hospital Universario LaPaz (IdiPAZ), University Hospital LaPaz, Autonomous University of Madrid, Madrid, Spain; 113 Department of Physiology and Biophysics, University of Mississippi Medical Center, Jackson, MS USA; 114 Instituto Nacional de Ciencias Medicas y Nutricion, Mexico City, Mexico; 115 Center for Non-Communicable Diseases, Karachi, Pakistan; 116 Cardiovascular Health Research Unit, University of Washington, Seattle, WA USA; 117 Department of Epidemiology, University of Washington, Seattle, WA USA; 118 Department of Business Data Convergence, Chungbuk National University, Gyeonggi-do, South Korea; 119 The Mindich Child Health and Development Insititute, Icahn School of Medicine at Mount Sinai, New York, NY USA; 120 Clinical Research Centre, Centre for Molecular Medicine, Ninewells Hospital and Medical School, Dundee, UK; 121 Section of Cardiology, Department of Medicine, VA Boston Healthcare, Boston, MA USA; 122 Brigham and Women’s Hospital, Boston, MA USA; 123 Intramural Administration Management Branch, National Heart Lung and Blood Institute, NIH, Framingham, MA USA; 124 Pat Macpherson Centre for Pharmacogenetics and Pharmacogenomics, Medical Research Institute, Ninewells Hospital and Medical School, Dundee, UK; 125 Division of Epidemiology and Community Health, University of Minnesota, Minneapolis, MN USA; 126 Department of Molecular Medicine and Biopharmaceutical Sciences, Graduate School of Convergence Science and Technology, Seoul National University, Seoul, South Korea; 127 Department of Internal Medicine, Seoul National University College of Medicine, Seoul, South Korea; 128 Life Sciences Institute, National University of Singapore, Singapore, Singapore; 129 Department of Statistics and Applied Probability, National University of Singapore, Singapore, Singapore; 130 Department of Preventive Medicine, Keck School of Medicine, University of Southern California, Los Angeles, CA USA; 131 Human Genetics Center, School of Public Health, The University of Texas Health Science Center at Houston, Houston, TX USA; 132 Instituto de Investigaciones Biomédicas, Departamento de Medicina Genómica y Toxicología, Universidad Nacional Autónoma de México, Mexico City, Mexico; 133 Research Unit of Molecular Epidemiology, Institute of Epidemiology, Helmholtz Zentrum München, German Research Center for Environmental Health, Neuherberg, Germany; 134 German Center for Diabetes Research (DZD e.V.), Neuherberg, Germany; 135 Deutsches Forschungszentrum für Herz-Kreislauferkrankungen (DZHK), Partner Site Munich Heart Alliance, Munich, Germany; 136 Institute of Medical Informatics, Biometry and Epidemiology, Chair of Genetic Epidemiology, Ludwig-Maximilians-Universität, Neuherberg, Germany; 137 Department of Medicine, University of Colorado Denver, Aurora, CO USA; 138 Novo Nordisk Foundation Center for Basic Metabolic Research, Faculty of Health and Medical Sciences, University of Copenhagen, Copenhagen, Denmark; 139 Faculty of Health Sciences, University of Southern Denmark, Odense, Denmark; 140 Department of Pediatrics, University of Colorado Anschutz Medical Campus, Aurora, CO USA; 141 Department of Epidemiology, Colorado School of Public Health, Aurora, CO USA; 142 Vanderbilt Genetics Institute, Vanderbilt University, Nashville, TN USA; 143 Department of Laboratory Medicine & Institute for Human Genetics, University of California, San Francisco, San Francisco, CA USA; 144 Blood Systems Research Institute, San Francisco, CA USA; 145 Department of Human Genetics, McGill University, Montreal, Quebec, Canada; 146 Division of Endocrinology and Metabolism, Department of Medicine, McGill University, Montreal, Quebec Canada; 147 McGill University and Génome Québec Innovation Centre, Montreal, Quebec Canada; 148 Division of General Internal Medicine, Massachusetts General Hospital, Boston, MA USA; 149 Center for Public Health Genomics, University of Virginia School of Medicine, Charlottesville, VA USA; 150 Department of Pediatrics, Los Angeles BioMedical Research Institute at Harbor-UCLA Medical Center, Torrance, CA USA; 151 Department of Medicine, Los Angeles BioMedical Research Institute at Harbor-UCLA Medical Center, Torrance, CA USA; 152 Institute for Translational Genomics and Population Sciences, Los Angeles BioMedical Research Institute at Harbor-UCLA Medical Center, Torrance, CA USA; 153 Department of Genetics, Harvard Medical School, Boston, MA USA; 154 Department of Biology, Massachusetts Institute of Technology, Cambridge, MA USA; 155 Department of Molecular Biology, Massachusetts General Hospital, Boston, MA USA; 156 Department of Biostatistics, University of Liverpool, Liverpool, UK; 157 Oxford NIHR Biomedical Research Centre, Oxford University Hospitals Trust, Oxford, UK

**Keywords:** Genomics, Protein sequencing, Genome-wide association studies, Next-generation sequencing

## Abstract

Protein-coding genetic variants that strongly affect disease risk can yield relevant clues to disease pathogenesis. Here we report exome-sequencing analyses of 20,791 individuals with type 2 diabetes (T2D) and 24,440 non-diabetic control participants from 5 ancestries. We identify gene-level associations of rare variants (with minor allele frequencies of less than 0.5%) in 4 genes at exome-wide significance, including a series of more than 30 *SLC30A8* alleles that conveys protection against T2D, and in 12 gene sets, including those corresponding to T2D drug targets (*P* = 6.1 × 10^−3^) and candidate genes from knockout mice (*P* = 5.2 × 10^−3^). Within our study, the strongest T2D gene-level signals for rare variants explain at most 25% of the heritability of the strongest common single-variant signals, and the gene-level effect sizes of the rare variants that we observed in established T2D drug targets will require 75,000–185,000 sequenced cases to achieve exome-wide significance. We propose a method to interpret these modest rare-variant associations and to incorporate these associations into future target or gene prioritization efforts.

## Abstract (web summary)

Exome-sequencing analyses of a large cohort of patients with type 2 diabetes and control individuals without diabetes from five ancestries are used to identify gene-level associations of rare variants that are associated with type 2 diabetes.

## Main

Human genetics offers a powerful approach for better understanding and treating disease by identifying molecular alterations that are causally associated with physiological traits^1^. Common-variant array-based genome-wide association studies (GWAS) have associated thousands of genomic loci with hundreds of human traits^2^, and further analyses indicate that heritability of most complex traits is attributable to modest-effect common regulatory variants^3^. However, non-coding GWAS associations are challenging to assign to causal variants or genes^4^.

Protein-coding variants with strong effects on protein function or disease can offer molecular ‘probes’ into the pathological relevance of a gene^5^ and potentially establish a direct causal link^6^ between gene gain- or loss-of-function and disease risk^7^—especially when there is evidence of multiple independent variant associations (an ‘allelic series’) within a gene^8^. Several lines of evidence^9^ predict that strong-effect variants (allelic odds ratios > 2) will usually be rare (minor allele frequency (MAF) < 0.5%) and, in many cases, difficult to accurately study through current array-based GWAS and imputation strategies^5^. Whole-genome or whole-exome sequencing, by contrast, allows interrogation of the full spectrum of genetic variation.

Previous exome-sequencing studies have identified relatively few exome-wide significant rare-variant associations for complex diseases such as T2D^10^. This paucity of findings is in part due to the limited sample sizes of previous studies, the largest of which included less than 10,000 disease cases and fall short of the sample sizes that analytic^9^ and simulation-based calculations^11^ predict are needed to identify rare disease-associated variants under plausible disease models. To increase rare coding variant analysis power, we collected and analysed exome-sequencing data from 20,791 T2D cases and 24,440 controls—one of the largest analyses of exome-sequenced cases for T2D, specifically, and for any disease, more generally.

## Genetic discovery from association analysis

**Extended Data Fig. 1:**
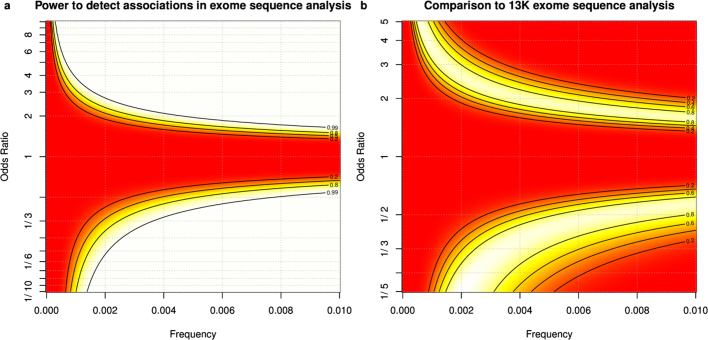
Power analysis. The power to detect associations (using a two-sided test) at *P* < 5 × 10^−8^ for variants (or collections of variants) with a given minor allele frequency (*x* axis) and odds ratio (*y* axis) measured as the average across all ancestries. **a**, Cells are shaded according to the power of the current study of 20,791 T2D cases and 24,440 controls, with white indicating high power and red indicating low power. The 20%, 50%, 80% and 99% contour lines are labelled. **b**, Cells are shaded according to the difference in power between the current study and a previously published study of 12,940 individuals^10^, with yellow–white indicating a large increase in power and red indicating a small increase in power. The 20%, 40%, 60% and 80% contour lines are labelled.

Study participants (Supplementary Table 1) were drawn from five self-reported ancestries: (Hispanic/Latino (effective size (*n*_eff_) = 14,442; 33.8%), European (*n*_eff_ = 10,517; 24.6%), African-American (*n*_eff_ = 5,959; 13.9%), East-Asian (*n*_eff_ = 6,010; 14.1%) and South-Asian (*n*_eff_ = 5,833; 13.6%)) and yielded equivalent statistical power to detect associations as a balanced study of around 42,800 individuals or a population-based study (assuming T2D prevalence of 8% and no ascertainment bias) of around 152,000 individuals. Power was improved compared to the previous largest T2D exome-sequencing study^10^ of 6,504 cases and 6,436 controls, increasing, for example, from 5% to 90% for a variant with MAF = 0.2% and odds ratio = 2.5 (Extended Data Fig. 1).

**Extended Data Fig. 2:**
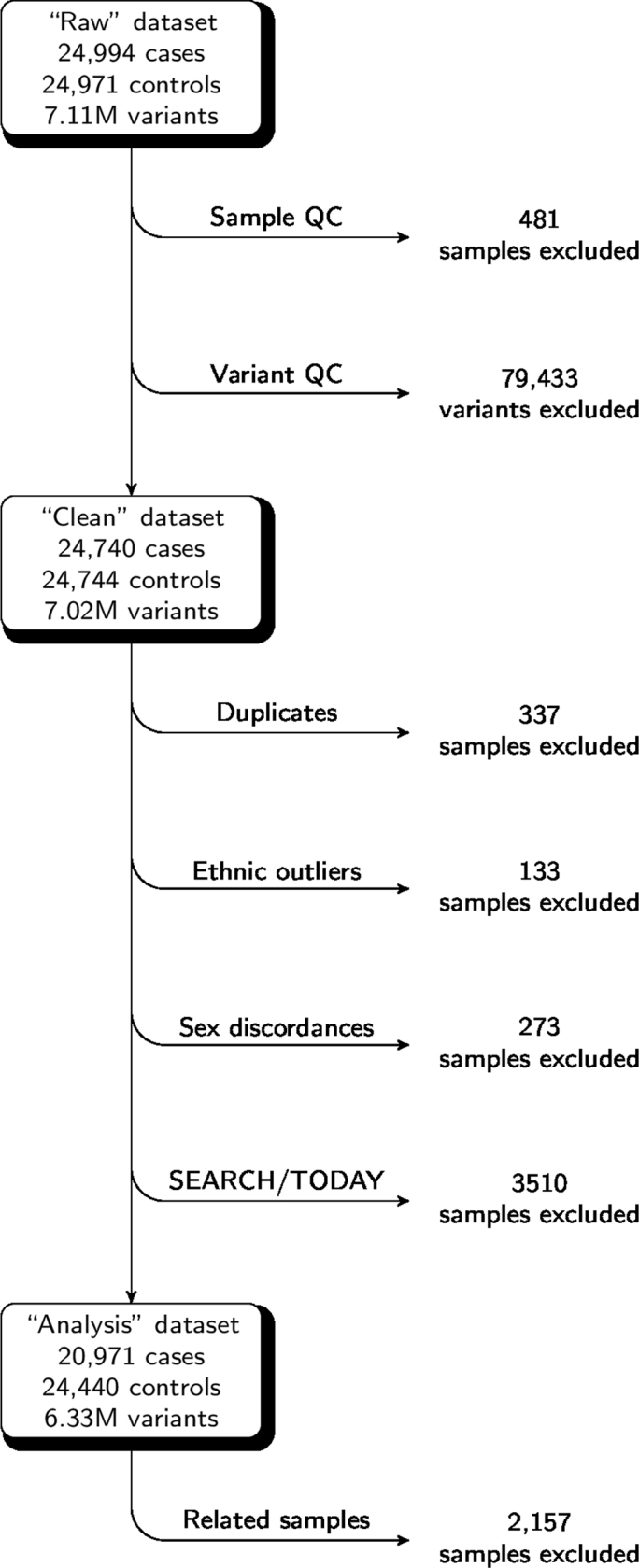
Data quality control workflow. A schematic of the steps involved in sample and variant quality control is shown. Quality control was conducted as described in the Methods to construct a set of samples and variants included in the association analysis. Each step is depicted as an arrow, with the number of samples or variants excluded by the step shown at the end of the arrow. The final set of samples and variants analysed are represented by the ‘Analysis’ dataset; we further excluded samples of high relatedness to other samples in the dataset from some but not all analyses. After each step that removed samples, we also removed newly monomorphic variants (hence the decrease in variants between the ‘Clean’ and ‘Analysis’ datasets).

Exome sequencing to 40x mean depth, variant calling and quality control (Extended Data Fig. 2, Supplementary Methods, Supplementary Figs. 1–3 and Supplementary Table 2) produced a dataset with 6.33 million variants: 2.3% common (MAF > 5%), 4.2% low-frequency (0.5% < MAF < 5%) and 93.5% rare (MAF < 0.5%) (Supplementary Table 3). These include 2.26 million nonsynonymous variants and 871,000 insertions and deletions (indels), more than twice the number of variants that were analysed in a previous T2D exome-sequencing study^10^.

**Fig. 1:**
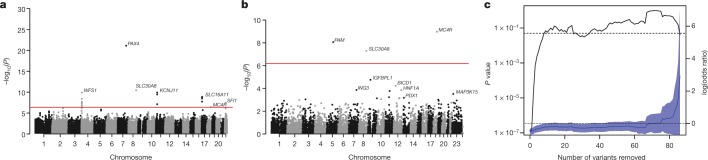
Exome-wide association analysis. **a**, Single-variant associations were calculated using the two-sided EMMAX test. Red line, *P* = 4.3 × 10^−7^. **b**, Gene-level *P* values, corrected for four analyses performed using the two-sided minimum *P*-value test. The most-significant genes are labelled. Red line, *P* = 6.5 × 10^−7^. **c**, *SLC30A8* gene-level *P* values (left *y* axis, black line), calculated using a two-sided burden test after removing variants (in order of increasing single-variant *P* value) from the 1/5 1% mask (the strongest signal for *SLC30A8*). Dashed line, *P* = 0.05. Right *y* axis (blue line), estimated effect size (log_10_(odds ratio)). Blue shading, 95% confidence interval. Dotted line, effect size = 0. Single-variant *n* = 45,231 individuals. Gene-level *n* = 43,074 unrelated individuals.

**Extended Data Fig. 3:**
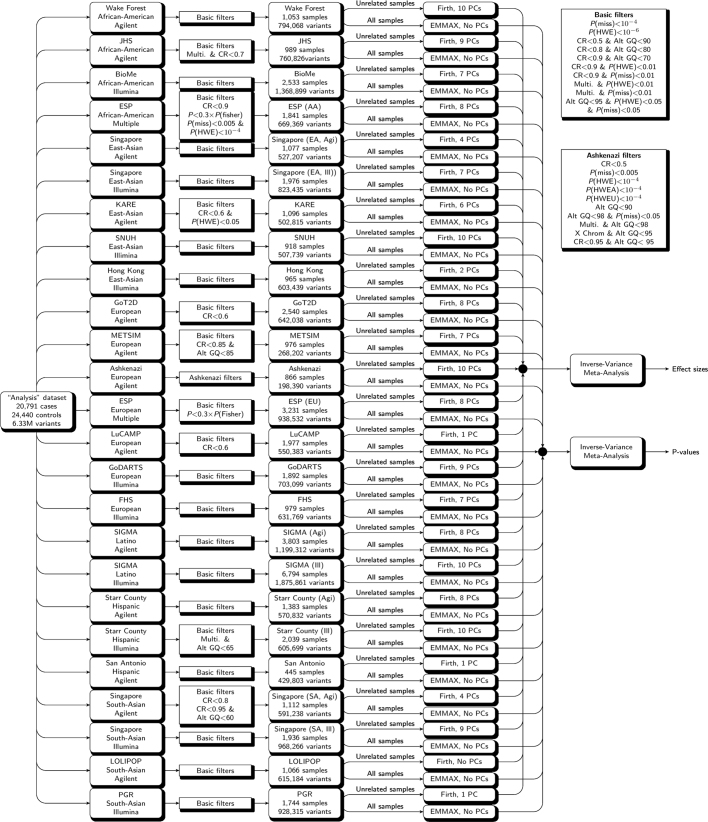
Single-variant association analysis workflow. A schematic of the steps involved in single-variant exome-sequencing association analysis is shown, as described in the Methods. We began analysis with a division of samples in the ‘Analysis’ dataset (leftmost column) into 25 different subgroups (second column from the left) based on cohort, ancestry and sequencing technology. We then filtered variants according to metrics computed separately for each subgroup; we applied the filters listed in the ‘Basic filters’ box to all subgroups and for some subgroups we applied additional (more stringent) filters as indicated by boxes in the third column from the left. The resulting number of variants and samples advanced for analysis in each subgroup are indicated in the fourth column from the left. We analysed each subgroup with both the EMMAX test (to measure association strength) and the Firth test (to measure allelic odds ratios), each of which are two-sided; the number of principal components included as covariates in the Firth test is shown in the fifth column from the left. Finally, we combined each of the EMMAX and Firth subgroup-level results using a 25-group meta-analysis to produce the final *P* values and odds ratios reported for each variant. Multi, variant is multiallelic; CR, call rate; *P*, variant subgroup-level *P* value; *P*(Fisher), variant subgroup-level *P* value from Fisher’s exact test; *P*(miss), *P* value for subgroup-level variant differential missingness between T2D cases and controls; *P*(HWE), *P* value for deviation from subgroup-level Hardy–Weinberg equilibrium; Alt GQ, mean genotype quality of non-reference genotypes (across all samples); X Chrom, variant is on X chromosome.

**Extended Data Fig. 4:**
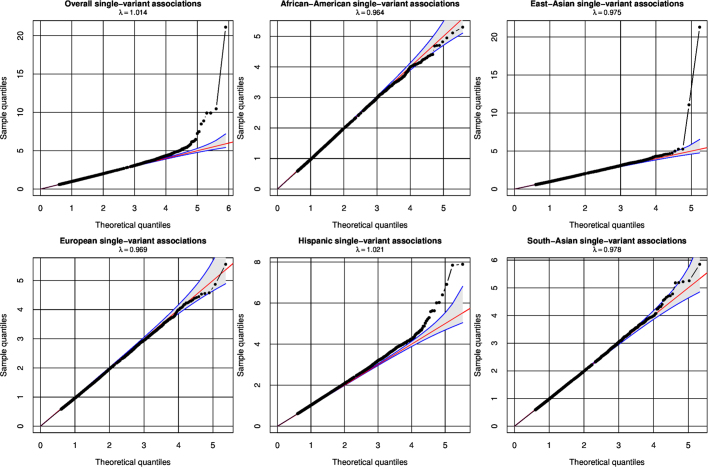
Calibration of single-variant analysis. To assess whether our single-variant association statistics (two-sided, calculated by the EMMAX test) were well-calibrated, we computed quantile–quantile plots of associations across all samples (Overall) and within each ancestry (total *n* = 45,231 individuals). To avoid deflation of the quantile–quantile plot from rare variants (for which the expected *P* values are discrete rather than uniformly distributed), only variants with minor allele counts of 20 or greater (either overall or within the relevant ancestry) are shown. Variants were also LD-pruned before plotting, to avoid induced variance from correlated *P* values of these variants, using the ‘clump’ method implemented in PLINK. The *λ* values indicate genomic control, as measured by the ratio in observed median *χ*^2^ statistic to that expected under the null hypothesis. Red line, expectation of *P* values under the null distribution. Blue lines (and grey region), 95% confidence interval of expectations under the null distribution.

We first tested each variant, regardless of allele frequency, for T2D association (‘single-variant’ test; Methods and Extended Data Figs. 3, 4). Fifteen variants (in seven loci) exceeded exome-wide significance (*P* < 4.3 × 10^−7^ for coding variants^12^, *P* < 5 × 10^−8^ for synonymous or non-coding variants), including ten nonsynonymous variants (Fig. 1a and Extended Data Table 1). These 15 associations are a substantial increase over the single association that was reported in a previous T2D-exome sequencing study^10^ and illustrate again the value of multi-ancestry association analyses^13^—as only 9 out of 15 variants achieved *P* < 0.05 in European samples. However, only two variants were not previously reported by GWAS: a variant in *SFI1* (rs145181683, Arg724Trp; Supplementary Fig. 4) that failed to replicate in an independent cohort (*n* = 4,522, *P* = 0.90; Methods) and a low-frequency (in Hispanic/Latino individuals; MAF = 0.89%) moderate-effect (odds ratio = 2.17, 95% confidence interval = 1.63–2.89) *MC4R* variant (rs79783591, Ile269Asn) that has previously been shown to decrease MC4R activity and to be associated with obesity and T2D in smaller studies^14^. Conditioning on body-mass index reduced but did not eliminate the *MC4R* Ile269Asn T2D association (*P* = 1.0 × 10^−5^).

**Extended Data Fig. 5:**
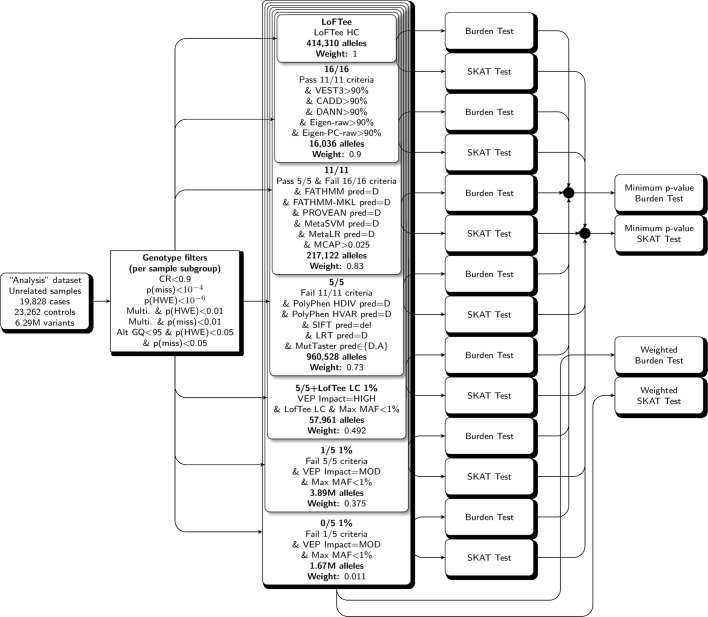
Gene-level association analysis workflow. A schematic of the steps involved in gene-level exome-sequencing association analysis, as described in Methods, is shown. We began analysis with subgroup-level genotype filtering (second column from the left) of unrelated samples in the ‘Analysis’ dataset (leftmost column); we then applied genotype filters for each subgroup (filtering genotypes for either all or no samples in each subgroup), similar to those used in subgroup-level single-variant analyses. We then annotated each non-reference variant allele with 16 different bioinformatics algorithms to assess allele deleteriousness, and we grouped alleles into one of seven nested masks (third column from the left; the number of variants and weights shown correspond to alleles absent from ‘higher’, or more stringent, nested masks). We computed burden and SKAT analyses (both of which are two-sided) using one of two approaches to combine alleles across masks (Methods): first, by analysing all alleles at once with weights assigned according to the most stringent mask containing the allele (weighted test); and second, by analysing each mask independently and then calculating the lowest *P* value corrected for the effective number of tests (minimum *P*-value test). Multi, variant is multiallelic; CR, call rate; *P*(miss), *P* value for subgroup-level variant differential missingness between T2D cases and controls; *P*(HWE), *P* value for deviation from subgroup-level Hardy–Weinberg equilibrium; Alt GQ, mean genotype quality of non-reference genotypes (across all samples).

**Extended Data Fig. 6:**
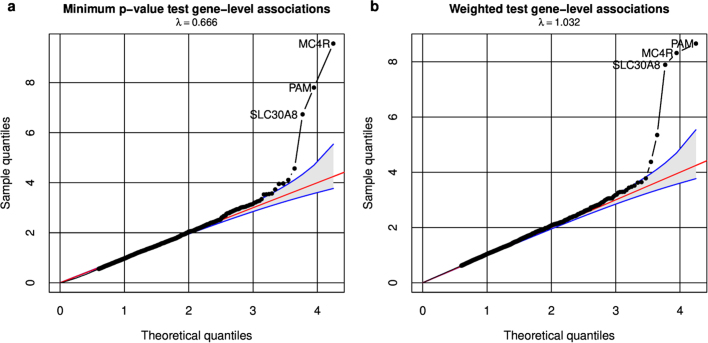
Calibration of gene-level association analyses. For both the burden and SKAT tests, we tested for gene-level association within seven different allelic masks. As this produced seven *P* values for each test, we developed two means to consolidate these results (Methods). **a**, **b**, The quantile–quantile plots of associations are shown for the minimum *P*-value burden test (**a**) and the weighted burden test (**b**). Each test is two-sided and consists of *n* = 43,071 unrelated individuals. Only genes with combined minor allele count of 20 or greater are shown in the quantile–quantile plots, to avoid deflation from genes with too few variants to produce *P* values asymptotically uniform under the null distribution. The *λ* values indicate genomic control, as measured by the ratio in observed median *χ*^2^ statistic to that expected under the null hypothesis. The three genes with exome-wide significant associations are labelled. Red line, expectation of *P* values under the null distribution. Blue lines (and grey region), 95% confidence interval of expectations under the null distribution.

Because single-variant analyses have limited power to detect rare-variant associations^9^, we next performed association tests for aggregations of variants within genes. Because numerous variant aggregation approaches (that is, ‘masks’) and gene-level tests are available, we developed a method (Methods, Extended Data Figs. 5, 6 and Supplementary Figs. 5, 6) to consolidate information across 14 analyses into four results per gene: burden^9^ and SKAT^15^ analyses, each of which were either summarized as the ‘minimum *P* value’ across masks or ‘weighted’ to estimate the effect of gene haploinsufficiency. We used an exome-wide gene-level significance threshold of *P* = 6.57 × 10^−7^ (Methods).

Using this strategy, gene-level associations were exome-wide significant for *MC4R*, *SLC30A8* and *PAM* (Fig. 1b, Extended Data Table 2 and Supplementary Table 4), with variants from multiple ancestries contributing to each signal (Methods). All three genes lie within reported T2D GWAS loci and contain previously identified coding variant signals: the common variant Arg325Trp and 12 rare protective protein-truncating variants (PTVs) for *SLC30A8*^7,16^, the low-frequency variants Asp563Gly and Ser539Trp for *PAM*^10,17^ and the low-frequency variant Ile269Asn for *MC4R*.

The associations in *MC4R* (combined MAF = 0.79%, minimum *P* = 2.7 × 10^−10^, odds ratio = 2.07, 95% confidence interval = 1.65–2.59) and *PAM* (combined MAF = 4.9%, weighted *P* = 2.2 × 10^−^^9^, odds ratio = 1.44, 95% confidence interval = 1.28–1.62) result largely from effects of the previously identified coding variants in these genes, although the *MC4R* signal remained nominally significant after removing Ile269Asn (*P* = 8.6 × 10^−3^; Supplementary Fig. 7) and the *PAM* signal remained nominally significant (*P* < 0.05) after removing the 35 strongest individually associated *PAM* variants (Supplementary Fig. 8). As illustrated by a recent study that identified a novel T2D risk mechanism through cellular characterization of PAM Asp563Gly and Ser539Trp^18^, variants identified in our study (uniquely from sequencing)^6^ could yield further insights into the T2D risk mechanism mediated by *PAM*.

In contrast to *MC4R* and *PAM*, the *SLC30A8* signal (103 variants, combined MAF = 1.4%, weighted *P* = 1.3 × 10^−8^, odds ratio = 0.40, 95% confidence interval = 0.28–0.55) was not primarily driven by an individual variant (Arg325Trp (MAF > 1%) was not included in the gene-level analysis). The association was instead driven by 90 missense variants (weighted *P* = 3.9 × 10^−7^) and remained nominally significant (*P* < 0.05) even when we removed the 32 strongest individually associated *SLC30A8* variants (Fig. 1c and Supplementary Fig. 9). Although *SLC30A8* was first implicated in T2D over a decade ago^16^, the disease-associated molecular mechanism(s) through which SLC30A8 acts remain poorly understood^19^—in part because the common risk-increasing allele Arg325Trp and the rare risk-decreasing PTVs were both initially thought to decrease protein activity^7,19^. The protective allelic series from our analysis argues that decreased T2D risk is the typical effect of *SLC30A8* missense variation—that is, it is not unique to haploinsufficiency—and provides many additional alleles that can be characterized to gain mechanistic insights.

To evaluate association evidence for genes other than *MC4R*, *PAM* and *SLC30A8*, we assessed the 50 most-significant gene-level associations from our study in two independent exome-sequencing datasets: 12,467 European or African-American individuals (3,062 T2D cases) from the CHARGE discovery sequencing project^20^ (Supplementary Table 5; 50 genes available) and 49,199 European individuals (12,973 T2D cases) from the Geisinger Health System (Supplementary Table 6; 44 genes available). In a meta-analysis of the three studies (Methods and Supplementary Table 7), *MC4R* (*P* = 6.9 × 10^−14^), *PAM* (*P* = 3.0 × 10^−9^) and *SLC30A8* (*P* = 3.3 × 10^−8^) each became more significant. In addition, one gene, *UBE2NL* (*P* = 5.6 × 10^−7^)—which has few prior links to T2D or other complex traits—newly achieved exome-wide significance (http://www.type2diabetesgenetics.org/). All aspects of this association passed quality control (Methods and Supplementary Table 8), although further replication will be important to establish *UBE2NL* as a novel T2D-relevant gene.

More broadly, we observed an excess of directionally consistent associations (both odds ratio > 1 or both odds ratio < 1) between the original and replication analyses (31 out of 46 in CHARGE, one-sided binomial *P* = 0.013; 23 out of 40 in the Geisinger Health System, *P* = 0.21; overall *P* = 0.011; Supplementary Table 7), suggesting that several more of our top gene-level signals will reach exome-wide significance in future studies.

## Further insights from gene-level analyses

Even if a gene-level association does not achieve exome-wide significance, it might still be of use to prioritize a gene as relevant to T2D^8^ or predict whether loss or gain of protein function increases disease risk^7^. To investigate potential insights that could be obtained by sub-exome-wide significant gene-level associations, we analysed 16 gene sets that were connected to T2D based on a variety of sources of evidence (for example, genes that contained diabetes-associated Mendelian variants, T2D drug targets^21^ or genes that have been implicated in diabetes-related phenotypes in mouse models^22^; Methods and Supplementary Table 9).

**Fig. 2:**
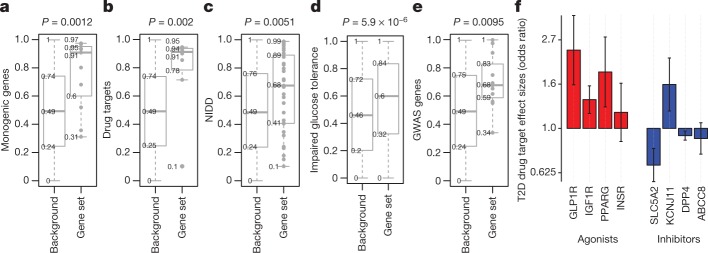
Gene set analysis. **a**–**e**, Rank percentiles (1 = highest) for gene-level associations (compared to matched genes) within 11 monogenic diabetes genes (548 matched genes) (**a**), 8 T2D drug targets (400 matched genes) (**b**), 31 genes linked to NIDD in mice (1,499 matched genes) (**c**), 323 genes linked to impaired glucose tolerance in mice (10,043 matched genes) (**d**) and 11 genes with common likely causal coding variants (537 matched genes) (**e**). *P* values are from a one-sided Wilcoxon rank-sum test between each gene set and comparison set. Labels indicate minimum, 25th percentile, median, 75th percentile and maximum. **f**, Estimated odds ratios, from the weighted burden test of the 5/5 mask, for 8 T2D drug targets (red, agonists; blue, inhibitors). Data are mean ± s.e. of log(odds ratio) from the burden test. *n* = 43,071 unrelated individuals.

First, for each gene set, we investigated whether the genes within the set had more significant gene-level associations than expected by chance (Methods). In total, 12 out of 16 gene sets achieved *P* < 0.05 set-level associations (Fig. 2a–e and Supplementary Fig. 10), including T2D drug targets (*P* = 2.1 × 10^−3^), genes previously reported in mouse models of non-insulin-dependent diabetes (NIDD; *P* = 5.2 × 10^−3^) or impaired glucose tolerance (*P* = 7.2 × 10^−6^) and genes that contained common likely causal coding-variant T2D associations^6^ (*P* = 8.8 × 10^−3^ after conditioning on the common variants nearby these genes). Additionally, as previously described^10^, we observed a significant set-level association (*P* = 1.2 × 10^−3^) for genes implicated in maturity onset diabetes of the young (MODY; Fig. 2a, Supplementary Table 10), with nominal associations in four genes including *PDX1* (weighted *P* = 1.7 × 10^−4^, odds ratio = 3.45, 95% confidence interval = 1.78–6.71, 65 variants). Rare variants in genes associated with MODY also demonstrated aggregate association with lower body-mass index (minimum *P* = 5.7 × 10^−3^) and lower fasting insulin (minimum *P* = 0.028), consistent with the known predominant variant risk mechanism of reduced insulin secretion in MODY^23^. Most gene set signals were driven by multiple genes in the set (Supplementary Table 11) and—compared with previous studies focused on PTVs^24^—consisted of substantial contributions from missense variants. Indeed, set-level *P* values from PTVs alone were >0.05 for almost all gene sets (Supplementary Fig. 11).

Collectively, these results suggest that association strength at the gene level can be used as a potential metric to prioritize candidate genes relevant to T2D. For example, the set of 40 genes within T2D GWAS loci with gene-level *P* < 0.05 had a significant excess of protein–protein interactions among them (Methods and Supplementary Table 12), suggesting that this set may be enriched for ‘effector genes’ that mediate T2D GWAS associations^6^. Fully evaluating the relevance to T2D of these and other candidate genes will require further experimental work^4^.

In addition to prioritizing genes that are potentially relevant to T2D, we assessed whether gene-level analysis could help to predict whether gene inactivation increases or decreases T2D risk, as this is of high interest for the development of therapeutics^8^. We compared the odds ratios that were estimated from a gene-level weighted burden analysis to directional relationships that have been previously reported (Methods). Seven out of eight T2D drug targets showed concordance between genetic and therapeutic directions of effect (three out of four inhibitor targets had an odds ratio < 1, four out of four agonist targets had an odds ratio > 1; one-sided binomial *P* = 0.035; Fig. 2f). The only exception was *KCNJ11* (odds ratio = 1.59, inhibited by sulfonylureas), for which the gene-level signal was driven by a known^25^ activating missense mutation (His172Arg); an analysis without this variant predicted the correct (odds ratio < 1) directional relationship. This finding is consistent with the known reciprocal roles of *KCNJ11* in both diabetes and persistent hyperinsulinaemic hypoglycaemia of infancy.

Concordances between gene-level estimates of odds ratios and knockout effects in mice were more equivocal (for example, 7 out of 11 diabetes-associated genes had an odds ratio > 1, binomial *P* = 0.27; 137 out of 240 genes associated with increased circulating glucose had an odds ratio > 1, *P* = 0.016; Supplementary Fig. 12). The lower concordances for these gene sets, despite a trend towards lower-than-expected gene-level *P* values within them (Supplementary Fig. 10), highlight the known limitations of animal models^26^, which can be highly dependent on model conditions^27^, to predict human physiology. Candidate genes with significant but directionally unexpected gene-level associations may provide valuable insights into seemingly promising preclinical results: for example, the protective gene-level signal for *ATM* in our analysis (burden test of PTVs odds ratio = 0.50, *P* = 0.003) contradicts previous expectations—based on insulin resistance and impaired glucose tolerance in *Atm* knockout mice^28^—that *ATM* loss-of-function should increase T2D risk. Evidence is even less favourable that *ATM* haploinsufficiency strongly increases T2D risk, rejecting an odds ratio > 2 at *P* = 1.3 × 10^−8^. These observations could be relevant in the ongoing study of whether *ATM* has a role in metformin response^29^ or whether *ATM* activators are considered able to treat cardiovascular disease^30^.

## Comparison of rare and common variant associations

Despite early arguments that rare-variant studies would considerably advance our understanding of complex diseases^5^, most genetic discoveries continue to be provided by studies of common variants, which can be studied in much larger sample sizes through array-based genotyping and imputation^31^. Previous quantitative analyses have similarly emphasized the main contribution of common variants to T2D heritability^6,10^, but they have lacked the sequencing data that are needed to fully evaluate the value added by rare variants (that is, direct sequencing in addition to array-based genotyping and imputation) to discover disease-associated loci, explain disease heritability and elucidate allelic series.

**Fig. 3:**
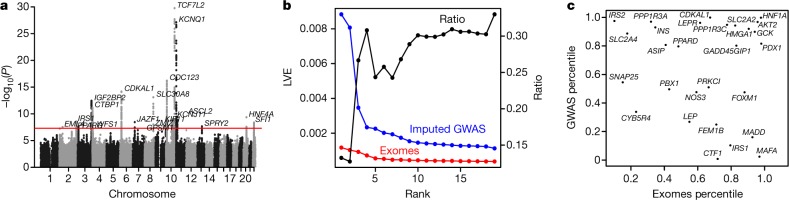
Comparison of exome-sequencing to array-based GWAS. **a**, Single-variant associations, calculated by two-sided Firth logistic regression, from an array-based imputed GWAS of the subset (*n* = 34,529) of samples in the exome-sequencing analysis for which array data were available. Labels and axes as described in Fig. 1a. **b**, The observed liability variance explained (LVE) by the top 19 exome-sequencing gene-level associations (red) and the top 19 imputed GWAS single-variant associations (maximum of 1 per 250 kb; blue) and their ratio (black). Signals ranked by liability variance explained. **c**, Gene rank percentiles from exome-sequencing gene-level analysis (*x* axis) and a previous multi-ancestry T2D GWAS^13^ (*y* axis). Genes are from the mouse NIDD gene set (Fig. 2c).

To compare discoveries that were possible from sequencing and array-based studies, we collected genome-wide array data within the same individuals that we sequenced (available for 34,529 (76.3%) individuals; 18,233 cases), imputed variants using best-practice reference panels^32,33^ and conducted a single-variant association analysis (‘imputed GWAS’; Methods and Supplementary Table 13). Out of 10 exome-wide significant nonsynonymous single-variant associations from the sequence analysis, 8 were detected in the imputed GWAS analysis (*PAX4* Arg192His and *MC4R* Ile269Asn were not imputable), together with genome-wide significant non-coding variant associations in 14 additional loci (Fig. 3a and Supplementary Table 14). All 10 variants with significant single-variant sequence associations were also present on the Illumina Exome Array^6^. These results demonstrate the limited power of sequencing to detect single-variant associations beyond array-based genotyping and imputation, even before considering the much larger sample sizes enabled by the substantially lower cost of array-based genotyping.

We next compared the contributions to T2D heritability of the strongest (common) single-variant associations from the imputed GWAS to those of the strongest (mostly rare-variant) gene-level associations from the sequencing analysis (Methods). The three exome-wide significant gene-level signals explain an estimated 0.11% (*MC4R*), 0.092% (*PAM*) and 0.072% (*SLC30A8*) of T2D genetic variance, only 10–20% of the variance explained by the three strongest independent common-variant associations in the imputed GWAS (*TCF7L2*, 0.89%; *KCNQ1*, 0.81%; *CDC123*, 0.35%; Fig. 3b). More broadly, fitting a previous exponential model of heritability^34^ to our data (Methods) estimated that the top 100 gene-level signals associated with T2D explained only 1.96% of genetic variance within our study. These results argue against a large contribution to T2D heritability from even the strongest gene-level signals, even after accounting for potential sources of downward bias in our calculations (see Methods).

We finally assessed whether an array-based GWAS could have detected the many potential allelic series that we observed from direct sequencing. Among the variants that contributed to the exome-wide significant gene-level associations in *SLC30A8*, *MC4R* and *PAM*, we estimate that 95.3% of variants are not imputable (*r*^2^ > 0.4; Methods) in the 1000 Genomes multi-ancestry reference panel^32^, 74.6% of those in Europeans are not imputable in the larger European-focused Haplotype Reference Consortium panel^10^ and 90.2% (79.7% of European variants) are absent from the Illumina Exome Array. Additionally, gene set associations (using gene ‘scores’; see Methods) from the imputed GWAS showed suggestive associations (four gene sets achieved *P* < 0.05, nine achieved *P* < 0.1; Supplementary Fig. 13) but were weaker than gene set associations from the sequencing analysis. Some of these gene set associations are detectable in larger array-based studies: analysis of a 110,000-sample multi-ancestry GWAS^13^ produced *P* < 0.05 for 12 out of 16 gene sets that we studied (Supplementary Fig. 14); however, the genes (and corresponding variants) that are responsible for the array-based gene set associations were mostly different from those responsible for the sequence-based associations, as the two methods often produced uncorrelated rank orderings of genes within gene sets (for example, *r* = −0.11, *P* = 0.57 for the mouse NIDD gene set; Fig. 3c).

Collectively, these results demonstrate the complementarity of array-based GWAS and exome sequencing, with the former favouring locus discovery and the latter enabling full enumeration of potentially informative alleles.

## Inferences from nominally significant associations

**Fig. 4:**
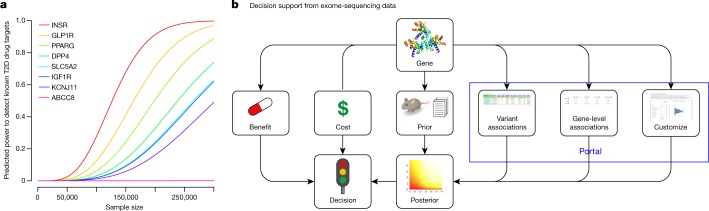
Decision support from exome-sequencing data. **a**, Estimated power, as a function of future sample size (*x* axis), to detect T2D gene-level associations (two-sided test at *P* = 6.25 × 10^−7^) with aggregate frequency and odds ratios equal to those estimated from our analysis of 8 T2D drug targets (Fig. 2f). **b**, A proposed workflow for using exome-sequencing data in gene characterization. Depending on the prior belief in the disease relevance of a gene, the cost of experimental characterization and the benefit of gene validation, a decision to conduct the experiment could be informed by the posterior probability of the disease relevance of the gene, as estimated from exome-sequencing association statistics (available through http://www.type2diabetesgenetics.org/).

The T2D drug targets analysed here illustrate the opportunities and challenges of using current exome-sequencing datasets in translational research. Rare-variant gene-level associations are significant across these targets as a set (Fig. 2b) and predict the correct T2D directional relationship for all but one gene (Fig. 2f). However, to detect—at exome-wide significance—the effect sizes estimated from our study with 80% power would require 75,000–185,000 sequenced cases (150,000–370,000 exomes in a balanced study, or 600,000–1,275,000 exomes from a population with a prevalence of T2D of 8%; Fig. 4a and Methods).

As a consequence, many of the modest associations (for example, *P* = 0.05) in current samples may point to clinically or therapeutically relevant variants or genes (Supplementary Fig. 15). The false-positive rate for these associations is expected to be greater than the false-positive rate for exome-wide significant associations^35^ and be further influenced by imperfect calibration of association test statistics. If this false-positive rate can be quantified using independent ‘truth’ data^36^, however, then a modest association signal could help to justify further experimentation on a gene based on the likelihood that it is a true association, the cost of the experiment and the benefit of success^37^ (Fig. 4b).

**Extended Data Fig. 7:**
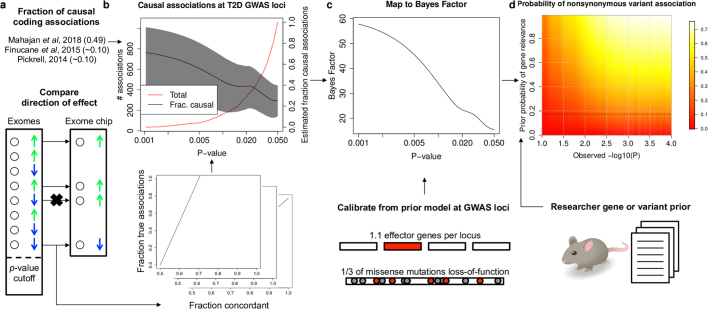
PPA calculation workflow. **a**, We estimated the PPAs for nonsynonymous variants in our sequence analysis based on concordance with independent exome array data and previously published^6,78,80^ estimates of the fraction of causal coding associations (Methods). **b**, PPA estimates for nonsynonymous variants within T2D GWAS loci are shown as a function of *P* value (right *y* axis, black line; 95% confidence interval, grey shading) together with the total number of such variants (left *y* axis, red line). **c**, For variants outside of T2D GWAS loci, we developed a method to further compute Bayes factors, which measure the odds of true and causal association, as a function of *P* value, using a model of the prior odds of true and causal association for variants in GWAS loci (Methods). **d**, These Bayes factors can be combined with a subjective prior belief in the T2D relevance of a gene (*y* axis) to produce the estimated posterior probability of true and causal association for any nonsynonymous variant in the exome-sequence dataset based on its observed log_10_(*P*) (*x* axis). Posterior estimates are shaded proportional to value (red, low; white, high). Values are shown for the default modelling assumptions of 33% of missense variants that caused gene inactivation and 30% of true missense associations that represented the causal variant.

**Extended Data Fig. 8:**
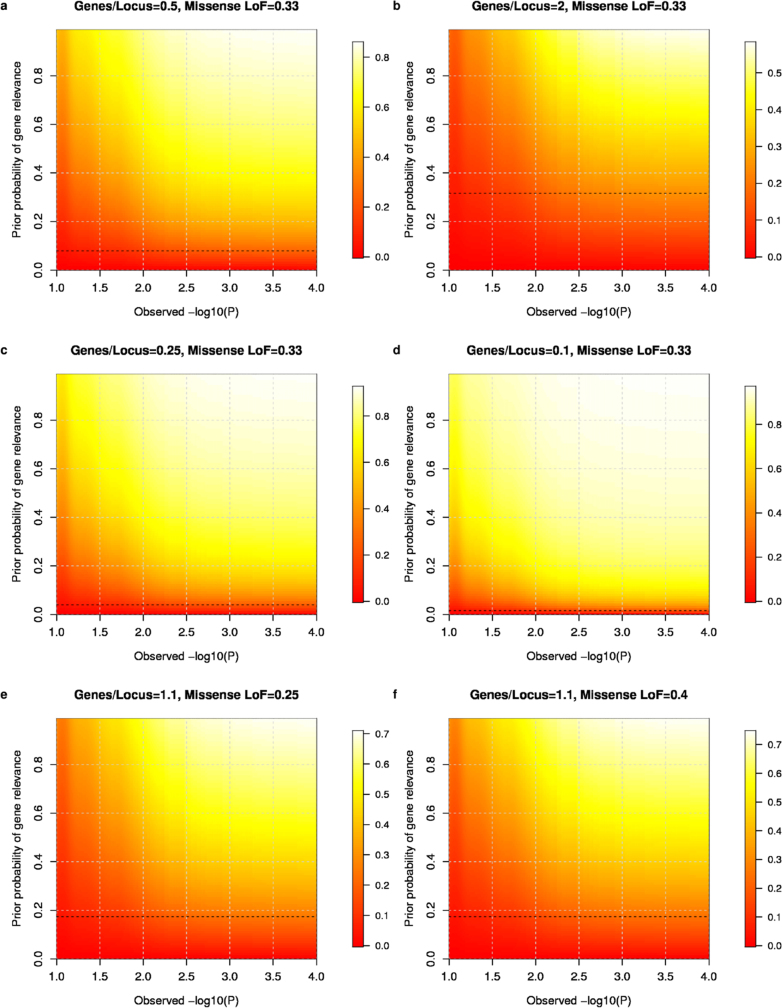
Estimated posterior probability of associations for different prior hypotheses. We estimated the posterior probability of association for nonsynonymous variants that met various single-variant *P*-value thresholds (two-sided EMMAX test, *n* = 45,231 individuals) in our analysis, as described in the Methods and shown in Extended Data Fig. 7. To perform the needed calculations, we assumed that, on average, 1.1 genes that are found within each T2D GWAS locus are relevant to T2D and 33% of missense mutations within these genes cause gene loss-of-function. **a**–**f**, To assess the sensitivity of our analysis to these assumptions, we repeated the calculations with different assumptions of 0.5 (**a**), 2.0 (**b**), 0.25 (**c**) and 0.1 (**d**) T2D-relevant genes within each GWAS locus, as well as 25% (**e**) and 40% (**f**) of missense variants leading to loss-of-function. All analyses assume the default modelling parameters that 30% of true nonsynonymous associations are causal associations; different values for this parameter would scale posterior probability estimates linearly.

We developed and evaluated a method to quantify the false-positive association rate for nonsynonymous variants in our dataset by using independent data, modelling assumptions and prior data to map single-variant *P* values to estimated posterior probabilities of true, causal associations (PPAs) (Methods and Extended Data Fig. 7). Model parameters in the middle of the range that we explored (Methods and Extended Data Fig. 8) predict that 1.5% (95% confidence interval = 0.74–2.2%) of nonsynonymous variants that achieve *P* < 0.05 in our study are truly, causally associated with T2D, increasing to 3.6% (95% confidence interval = 1.4–5.9%) for *P* < 0.005 and 9.7% (95% confidence interval = 3.9–15.0%) for *P* < 5 × 10^−4^ (Supplementary Fig. 16). In this model, 541 (95% confidence interval = 270–810) of the 36,604 nonsynonymous variants with *P* < 0.05 in our study represent true, causal associations.

We next applied this method to variants within a curated set of 94 T2D GWAS loci (Methods), which might be expected to show further enrichment of true associations. Our model predicted that nonsynonymous variants within these loci had even higher PPAs: 2.0% (95% confidence interval = 0.048–4.0%) of such variants overall, 8.1% (3.6–12.4%) with *P* < 0.05 in our study and 17.2% (7.7–24.1%) with *P* < 0.005 were estimated to represent true, causal T2D associations. Of particular note are variants in these loci that not only achieve nominal significance (*P* < 0.05) in our analysis but also have moderate-to-large estimated effects on T2D risk (Supplementary Tables 15, 16), as we predict that a substantial number of these variants (for example, 76 (95% confidence interval = 29–117) out of 746 with estimated odds ratio > 2 and 50 (95% confidence interval = 19–77) out of 503 with estimated odds ratio > 3) show true, causal associations.

Outside of GWAS loci, many genes are suspected to be involved in T2D because of prior evidence from non-genetic sources (for example, animal studies^22^ or because of implication in related disorders^23^). To evaluate variants in such genes, we extended our PPA estimation approach to incorporate gene prior probabilities (or ‘priors’)^38^ (Methods and Extended Data Fig. 7d) and applied it to two sets of genes.

First, using a prior of 100% for genes associated with MODY—thus assuming that all genes implicated in MODY are relevant to T2D—our model predicts 24 variants (combined MAF = 1.1%) to have PPA ≥ 40% (Supplementary Table 17). Nine have estimated odds ratio > 3 in our study; as none of these were previously reported to be pathogenic MODY variants, they are therefore novel rare-variant candidates for use in the prediction of T2D risk. On the other hand, these results show that, once false-positive rates are empirically estimated rather than assumed, nominally significant variants (*P* = 0.05) in genes associated with MODY are still, in absolute terms, more likely to be false-positive rather than true associations^39^.

Second, as an example of a gene prior that was derived objectively (rather than subjectively), we used a mixture model approach^40^ to estimate the proportion of non-null associations across the mouse NIDD gene set (Methods), leading to a prior of approximately 23% for genes of which knockout causes NIDD in mice. Our model with this prior (Supplementary Table 18) predicts nonsynonymous variants that achieved *P* < 0.05 to have PPAs of 9.9% (PPAs of 24.6% for *P* < 0.005). In particular, we predict several nonsynonymous variants in *MADD* and *NOS3* to have PPA ≥ 14% (Supplementary Table 19), suggesting links between variation in these genes and T2D based on combined evidence from human genetic studies and mouse models^41,42^.

Although these PPA calculations have limitations (Methods), they present a framework to use suggestive genetic signals to support cost–benefit estimates of ‘go/no-go’ decisions^43^ in the language of decision theory^37^ (Fig. 4b). To enable this strategy, we have made our exome-sequencing association results publically available through the AMP T2D Knowledge Portal (http://www.type2diabetesgenetics.org/), which supports queries of precomputed associations and further enables dynamic recomputations of associations with custom covariates and sample- and/or variant-filtering criteria.

## Discussion

Our results provide a nuanced description of rare variation and its association with T2D, which might also apply to other complex diseases. Rare-variant gene-level signals are likely to be distributed across numerous genes; however, the vast majority of signals individually explain vanishingly small amounts of T2D heritability: more than one million samples may be required for rare-variant signals in validated therapeutic targets to become significant exome-wide. Even among the four genes that reached exome-wide significance in our analysis, two (*MC4R* and *PAM*) do not include unusually strong rare-variant associations but rather typically modest rare-variant associations that are boosted from nominal to exome-wide significance by low-frequency variants.

Thus, for biological discovery in many complex traits, such as T2D, exome sequencing and array-based GWAS seem complementary: locus discovery and fine mapping are achieved most efficiently using larger array-based GWAS, whereas rare coding variant allelic series—that could aid experimental gene characterization^44^ or provide confidence in disease-gene identification—are best discoverable through sequencing. For personalized medicine, exome sequencing may produce some rare variants with sufficient effect sizes (Supplementary Tables 12, 17) to provide viable contributions to the prediction of genetic risk; however, these are sufficiently rare to be best viewed as complements to rather than replacements for GWAS-derived polygenic risk scores^45^. Whole-genome sequencing might soon become sufficiently cost-effective to subsume both array-based GWAS and exome sequencing; even now, it is essential to expand imputation reference panels to power higher-resolution GWAS across all major ethnicities.

Our results suggest that, for now, maximizing the utility of exome sequencing will require drawing insights from associations that do not (yet) reach exome-wide significance. To help to interpret these suggestive associations, we present a principled and empirically calibrated Bayesian approach (Fig. 4, Extended Data Fig. 7 and Supplementary Table 18) to estimate the association probability for any variant in our dataset, highlighting its use to interpret variants in known disease genes and prioritize genes from animal model studies for further investigation. Results and customized analyses from our study can be accessed through a public web portal (http://www.type2diabetesgenetics.org/), advancing the use of exome-sequencing data across many branches of biomedical research.

## Methods

A full description of the methods used in this study is available as Supplementary Methods.

### Data reporting

The experiments were not randomized and the investigators were not blinded to allocation during experiments and outcome assessment.

### Sample selection

We drew samples for exome sequencing from six consortia, most of which consisted of multiple studies and are described fully in Supplementary Table 1. T2D case status was determined according to study-specific criteria described in full in in Supplementary Table 1 and the Supplementary Methods. All individuals provided informed consent and all samples were approved for use by their institution’s institutional review board or ethics committee, as previously reported^10,46–48^. Samples that were newly sequenced at The Broad Institute as part of T2D-GENES, SIGMA and ProDiGY are covered under Partners Human Research Committee protocol 2017P000445/PHS ‘Diabetes Genetics and Related Traits’.

### Data generation

The details of data generation, variant calling, quality control and variant annotation are described in full in the Supplementary Methods. In brief, for each consortium, sequencing data were aggregated (if previously available) or newly generated (if not) and then processed through a standard variant calling pipeline. We then measured samples and variants according to several metrics indicative of sequencing quality, excluding those that were outliers relative to the global distribution (Supplementary Fig. 1, Supplementary Table 2). These exclusions produced a ‘clean’ dataset of 49,484 samples and 7.02 million variants.

Following initial sample and variant quality control, we performed additional rounds of sample exclusion from association analysis (Extended Data Fig. 2). We also excluded the 3,510 childhood diabetes cases from the SEARCH and TODAY studies based on an analysis that suggested their lack of matched controls would induce artefacts in gene-level association analyses (Supplementary Fig. 17). These exclusions produced an ‘analysis’ dataset of 45,231 individuals and 6.33 million variants. A power analysis of this dataset is presented in the Supplementary Methods.

After these three rounds of sample exclusions, we estimated—within each ancestry—pairwise identity-by-descent values, genetic relatedness matrices and principal components for use in downstream association analyses. We used the identity-by-descent values to generate lists of unrelated individuals within each ancestry, excluding 2,157 individuals to produce an ‘unrelated analysis’ set of 43,090 individuals (19,828 cases and 23,262 controls) and 6.29 million non-monomorphic variants. We used this set of individuals and variants for single-variant and gene-level tests (described below) that required an unrelated set of individuals.

We annotated variants with the ENSEMBL Variant Effect Predictor^49^ (VEP, version 87). We produced both transcript-level annotations for each variant as well as a ‘best guess’ gene-level annotation using the –flag-pick-allele option (with ranked criteria described in the Supplementary Methods). We used the VEP LofTee (https://github.com/konradjk/loftee) and dbNSFP (version 3.2)^50^ plugins to generate additional bioinformatics predictions of variant deleteriousness; from the dbNSFP plugin, we took annotations from 15 different bioinformatics algorithms (listed in Extended Data Fig. 5) and then added annotations from the mCAP^51^ algorithm. As these annotations were not transcript-specific, we assigned them to all transcripts for the purpose of downstream analysis.

Although we incorporated both transcript-level and gene-level annotations into gene-level analyses (see below), all single-variant analyses reported in the manuscript or figures are annotated using the ‘best guess’ annotation for each variant.

### Single-variant association analysis in sequencing data

To perform single-variant association analyses, we first stratified samples by cohort of origin and sequencing technology (with some exceptions described in the Supplementary Methods), yielding 25 distinct sample subgroups (Extended Data Fig. 3). For each subgroup, we performed additional variant quality control beyond that used for the ‘clean’ dataset, excluding variants according to subgroup-specific criteria described in Extended Data Fig. 3; in general, these criteria were strict—particularly for multiallelic variants and X-chromosome variants. We verified that these filters led to a well-calibrated final analysis through inspection of quantile–quantile plots within and across ancestries (Extended Data Fig. 4).

For each of the 25 sample subgroups, we then conducted two single-variant association analyses: one of all (including related) samples using the (two-sided) EMMAX test^52^ and one of unrelated samples using the (two-sided) Firth logistic regression test^53^. Both analyses included covariates for sequencing technology, and the Firth analysis included covariates for principal components of genetic ancestry (those among the first 10 that showed *P* < 0.05 association with T2D).

We then conducted a 25-group fixed-effect inverse-variance weighted meta-analysis for each of the Firth and EMMAX tests, using METAL^54^. We used EMMAX results for association *P* values and Firth results for effect size estimates.

### Additional analysis of rs145181683

To assess whether the rs145181683 variant in *SFI1* (*P* = 3.2 × 10^−8^ in the exome-sequencing analysis) represented a true novel association, we obtained association statistics from 4,522 Latinos^55^) who did not overlap with the current study. On the basis of the odds ratio (1.19) estimated in our analysis and the MAF (12.7%) in the replication sample, the power was 91% to achieve *P* < 0.05 under a one-sided association test. The observed evidence (*P* = 0.90, odds ratio = 1.00) did not support rs145181683 as a true T2D association. Further investigation of this lack of replication evidence suggested that, although the association from our sequence analysis is unlikely to be a technical artefact (genotyping quality was high), it could possibly be a proxy for a different (Native American-specific) non-coding causal variant (full details are available in the Supplementary Methods). Further fine-mapping and replication efforts will be necessary to test this hypothesis.

### Gene-level analysis

For each gene, following previous studies^10,56,57^, we separately tested seven different ‘masks’ of variants grouped by similar predicted severity (defined in Extended Data Fig. 5). For each gene and each mask, we created up to three groupings of alleles, corresponding to different transcript sets of the gene; for many genes, two or more of these allele groupings were identical.

Before running gene-level tests, we performed additional quality control on sample genotypes. For each of the 25 sample subgroups (the same as used for single-variant analyses), we identified variants that failed subgroup-specific quality control criteria (shown in Extended Data Fig. 5) and set genotypes for these variants in all individuals in the subgroup as ‘missing’.

We conducted two gene-level association tests: a burden test, which assumes all analysed variants within a gene are of the same effect, and SKAT^15^, which allows variability in variant effect size (and direction); each of these tests is two-sided. We performed each test across all unrelated individuals with 10 principal components of genetic ancestry, sample subgroup and sequencing technology as covariates. As this ‘mega-analysis’ strategy was different from the meta-analysis strategy that we used for single-variant analyses, as a quality control exercise we conducted a single-variant mega-analysis and found that its results showed broad correlation with those from the original meta-analysis (Supplementary Fig. 18).

We then developed two methods to consolidate the 2 × 7 = 14 *P* values produced for each gene (described in full in Extended Data Fig. 5, Supplementary Methods and Supplementary Figs. 5, 6). First, we corrected the smallest *P* value for each gene according to the effective number of independent masks tested for the gene (variable, but on average 3.6), based on the gene-specific correlation of variants across masks^58^ (referred to as the minimum *P*-value test; Supplementary Fig. 19). Second, we tested all nonsynonymous variants (that is, missense, splice site and protein-truncating mutations), but weighted each variant according to its estimated probability of causing gene inactivation^9^ (referred to as the weighted test, which essentially assessed the effect of gene haploinsufficiency from combined analysis of protein-truncating and missense variants; Supplementary Fig. 6). We verified that these two consolidation methods were well-calibrated (Extended Data Fig. 6) and broadly consistent yet distinct: across the 10 most significantly associated genes, *P* values were nominally significant using both methods for 8 genes but varied by 1–3 orders of magnitude (Extended Data Table 2).

Because each gene mask could in fact represent up to three sets of alleles (owing to the transcript-specific annotation strategy that we used), for each of the four analyses multiple *P* values were possible for some genes. To produce a single gene-level *P* value for each of the four analyses, we thus collapsed (for each gene) the set of *P* values across transcript sets into a single gene-level *P* value using the minimum *P*-value test.

We used a conservative Bonferroni-corrected gene-level exome-wide significance threshold of *P* = 0.05/(2 tests × 2 consolidation methods × 19,020 genes) = 6.57 × 10^−7^. For each gene referenced in the manuscript, we report the *P* value and odds ratio from the analysis that achieved the lowest *P* value for the gene.

### Gene-level analysis near T2D GWAS signals

In principle, a nearby common-variant association could lead to over- or underestimation of the strength of a gene-level association^59^. To assess whether differential patterns of rare variation across common-variant haplotypes could significantly affect our gene-level results, we conducted two analyses (described in the Supplementary Methods) and found no evidence that confounding from common-variant haplotypes was primarily responsible for the associations that were observed in our gene-level analyses.

### Further exploration of significant gene-level associations

For our exome-wide significant gene-level associations (*MC4R*, *PAM* and *SLC30A8*), we conducted additional gene-level analyses to dissect the aggregate signals that were observed. First, we performed tests by progressively removing alleles in order of lowest single-variant analysis *P* value, in order to understand the (minimum) number of alleles that contributed statistically to the aggregate signal. Second, we performed tests conditional on each allele in the sequence (that is, calculating separate models with each individual allele as a covariate), and we then compared the resulting *P* values to the full gene-level *P* value, in order to assess the contribution of each allele individually to the signal. Finally, for *MC4R*, we conducted an analysis with an added sample covariate for body-mass index and found that it, as shown previously^60,61^, reduces the significance of both the Ile269Asn single-variant signal (*P* = 1.0 × 10^−5^) and the gene-level signal not attributable to Ile269Asn (*P* = 0.035).

To evaluate which ancestries contributed variants to *MC4R*, *SLC30A8*, and *PAM*, we calculated the proportion of variants in each signal unique to an ancestry and also compared the significance and direction of effect of each signal across ancestries. Across the three signals, 68.4% (287 out of 419) of variants in total were unique to one ancestry (63.9% for *MC4R*, 67.0% for *SLC30A8* and 71.6% for *PAM*). Each signal had a direction of effect that was consistent across all five ancestries and each signal achieved *P* < 0.05 in at least two ancestries (*MC4R* in East-Asians and Hispanics; *SLC30A8* in all ancestries other than African-Americans; and *PAM* in Europeans, South-Asians and Hispanics).

### Analysis of exomes from the Geisinger Health System

We obtained gene-level association results that were previously computed from an analysis of 49,199 individuals (12,973 T2D cases and 36,226 controls) from the Geisinger Health System (GHS). Association statistics were available for 44 out of the 50 genes with the strongest gene-level associations from our study. A power analysis of the GHS replication analysis is available in the Supplementary Methods.

GHS sequencing data were processed and analysed as previously described^24^, and variants were grouped into four (nested) masks (roughly corresponding to the LofTee, 5/5, 1/5 1% and 0/5 1% masks; more details are available in the Supplementary Methods). For each mask, association results were computed using two-sided logistic regression under an additive burden model (with phenotype regressed on the number of variants carried by each individual) with age, age^2^ and sex as covariates. To produce a single GHS *P* value for each gene, we applied the minimum *P*-value procedure across the four mask-level results.

### Analysis of exomes from the CHARGE consortium

We collaborated with the CHARGE consortium to analyse the 50 genes with the strongest gene-level associations from our study in 12,467 individuals (3,062 T2D cases and 9,405 controls) from their previously described study^62,63^. A power analysis of the CHARGE replication analysis is available in the Supplementary Methods.

Variants in the CHARGE exomes were annotated and grouped into seven masks using the same procedure as for the original exome-sequencing analysis. Burden and SKAT association tests were then performed in the Analysis Commons^64^ using a two-sided logistic mixed model^65^ assuming an additive genetic model and adjusted for age, sex, study, race and kinship. To produce a single CHARGE *P* value for each gene, we applied the minimum *P*-value procedure across the seven mask-level results, as for the GHS analysis.

### Meta-analysis with CHARGE and GHS

We conducted a meta-analysis among our original burden analysis and those of CHARGE and GHS. For each gene, we selected the mask that achieved the lowest *P* value in our original analysis and conducted a two-sided sample-size weighted meta-analysis with the results from CHARGE and GHS for the same mask (or an analogous mask as defined in the Supplementary Methods).

### Investigation of the *UBE2NL* association

We investigated the novel association that was found in the gene-level meta-analysis (*UBE2NL*, meta-analysis *P* = 5.6 × 10^−7^) in more detail. The *UBE2NL* burden signal was due to five PTVs in the original analysis (observed in 29 cases and 1 control; all of which had high (>45×) sequencing coverage; Supplementary Table 8) and was replicated at *P* = 0.02 in CHARGE; *UBE2NL* results were not available in GHS. As *UBE2NL* lies on the X chromosome, we conducted a sex-stratified analysis of the original samples and observed independent associations in both men (*P* = 5.7 × 10^−4^) and women (*P* = 1.6 × 10^−3^). *UBE2NL* does not lie near any known GWAS associations (http://www.type2diabetesgenetics.org/) and has few available references^66–68^, suggesting that it may be a novel T2D-relevant gene, although further replication will be important to establish its association.

### Evaluation of directional consistency between exome-sequencing, CHARGE and GHS analyses

We examined the concordance of direction of effect size estimates (that is, both odds ratios of >1 or <1) between burden tests from our original exome-sequencing analysis and those from CHARGE and GHS. For the 46 genes advanced for replication with burden *P* < 0.05 for at least one mask (that is, ignoring those with evidence for association only under the SKAT model), we compared the direction of effect estimated for the mask with lowest *P*-value mask to that estimated for the same (or analogous) mask in the GHS or CHARGE analysis. We then conducted a one-sided exact binomial test to assess whether the fraction of results with consistent direction of effects was significantly greater than expected by chance.

### Gene set analysis in sequencing data

We curated 16 sets of candidate T2D-relevant genes, defined in Supplementary Table 9 with criteria as specified in the Supplementary Methods. For each gene set, we constructed sets of matched genes with similar numbers and frequencies of variants within them (details are provided in the Supplementary Methods). A sensitivity analysis of this matching strategy is presented in the Supplementary Methods.

To conduct a gene set analysis, we then combined the genes in the gene set with the matched genes. Within the combined list of genes, we ranked genes using the *P* values observed for the minimum *P*-value burden test. We then used a one-side Wilcoxon rank-sum test to assess whether genes in the gene set had significantly higher ranks than the comparison genes.

### Use of gene-level associations to predict effector genes

To assess whether gene-level associations from exome sequencing—which are composed mostly of rare variants independent of any GWAS associations—could prioritize potential effector genes within known T2D GWAS loci, we first assessed whether predicted effector genes (based on common-variant associations) were also enriched for rare coding variant associations. Our analysis (described in full in the Supplementary Methods) indicated that effector genes predicted from common coding variant associations do show significant enrichments (*P* = 8.8 × 10^−3^), but effector genes predicted from transcript-level associations do not (*P* = 0.72).

We then curated a list of 94 T2D GWAS loci, and 595 genes that were within 250 kb of any T2D GWAS index variant, from a 2016 T2D genetics review^69^ and observed 40 with a *P* < 0.05 gene-level signal (Supplementary Table 12), greater than the 595 × 0.05 = 29.75 expected by chance (*P* = 0.038). Only three (*SLC30A8*, *PAM* and *HNF1A*) were from the list that we curated of 11 genes with causal common coding variants^6^. We found that these 40 genes were significantly more enriched for protein interactions (*P* = 0.03; observed mean = 11.4, expected mean = 4.5) than the 184 genes implicated based on proximity to the index SNP (*P* = 0.64; observed mean = 21.1, expected mean = 21.9), although evaluation of the biological candidacy of these genes will ultimately require in-depth functional studies^70^. Rare coding variants could therefore, in principle, complement common-variant fine-mapping^71,72^ and experimental data^4,70^ to help to interpret T2D GWAS associations; however, our results indicate that much larger sample sizes and/or orthogonal experimental data will be required to clearly implicate specific effector genes. A full description of this analysis is included in the Supplementary Methods.

### Use of gene-level associations to predict direction of effect

To assess whether gene-level association analyses of predicted deleterious variants could be used to predict therapeutic direction of effect, we compared odds ratios estimated from a modified weighted burden test procedure (described in the Supplementary Methods) to those expected for T2D drug targets (assuming agonist targets to have true odds ratios > 1 and inhibitors to have true odds ratios < 1). For a similar comparison to expectations for mouse gene knockouts, we used the relationship between mouse phenotype and human phenotype specified in the Supplementary Methods. Genes present in two gene sets with opposite expected direction of effects were excluded from this analysis.

### Collection and analysis of SNP array data

To compare discoveries from our exome-sequencing analyses to discoveries possible from common-variant GWAS of the same samples, we aggregated all available SNP array data for the exome-sequenced samples (18,233 cases and 17,679 controls; Supplementary Table 13). After sample and variant quality control (described in the Supplementary Methods), we imputed variants from the 1000 Genomes Phase 3^32^ (1000G) and Haplotype Reference Consortium^33^ (HRC) reference panels using the Michigan Imputation Server^73^. We used 1000G-based imputation for all association analyses and HRC-based imputation to assess the number of exome-sequence variants imputable from the largest available European reference panel (details available in the Supplementary Methods).

After imputation, we performed sample and variant quality control, as well as two-sided association tests, analogous to the exome-sequence single-variant analyses. In contrast to the exome-sequencing analyses, a quantile–quantile plot suggested that the associations from the EMMAX test were not well calibrated, and we therefore used only the Firth test (that is, for both *P* values and odds ratios) in the imputed GWAS analysis.

To conduct gene set analysis with the imputed GWAS data, we first used the method implemented in MAGENTA^74^ to calculate gene scores from the imputed GWAS single-variant association results. Following the same protocol as for gene set analysis from the exome-sequencing results, we then conducted a one-sided Wilcoxon rank-sum test to compare the gene scores to those of matched comparison genes. We followed the same approach for the gene set analysis that we conducted in a larger, previously published^13^ GWAS.

### LVE calculations

To calculate LVEs, we used a previously presented formula^75^ (equations are available in the Supplementary Methods) to calculate the LVE of a variant with three genotypes (AA, Aa and aa) and corresponding relative risks (1, RR_1_ and RR_2_). When presenting the strongest LVE values for the imputed GWAS analysis, we only considered variants that were genotyped in at least 10,000 individuals to avoid potential artefacts that result from a spurious association in a small-sample subgroup. For gene-level LVE calculations, we used the variant mask with lowest *P* value to calculate LVEs. We also conducted a sensitivity analysis to bound the extent to which our gene-level LVE estimates might be biased downwards due to their inclusion of benign alleles; this analysis (described in full in the Supplementary Methods) produced upper bounds of gene-level LVEs that were at most twofold higher than the point estimates.

### Prediction of LVE explained by the top 100 and top 1,000 gene-level associations

To forecast the LVE that will be explained once 100 (or 1,000) significant T2D gene-level associations are detected, we applied a previously suggested model^34^ in which the LVE of a gene is related to its rank in the overall gene-level *P*-value distribution. Specifically, the model is LVE_*n*_ = e^*an* + *b*^ where LVE_*n*_ is the LVE of the gene with *n*th lowest gene-level *P* value. We fitted this model using linear regression to the top 50 genes in our analysis (Supplementary Fig. 20), yielding estimates of *a* = −0.044 and *b* = −7.07. We then calculated the LVE of the top 100 (or 1,000) genes by summing the actual LVE of the top three signals (which achieved exome-wide significance in our analysis) with the LVE predicted by the model for genes ranked 4–100 (or 4–1,000).

### Estimated power to detect gene-level associations with T2D drug targets

To estimate the power of future studies to detect gene-level associations in genes with effect sizes similar to those for established T2D drug targets, we used aggregate allele frequencies and odds ratios estimated from our gene-level analysis and an assumed prevalence of *K* = 0.08 to calculate a proxy for true population frequencies and relative risks. For each gene, we used odds ratios and frequencies from the variant mask that yielded the strongest gene-level association. Because, on average, these drug targets had five effective tests per mask, we used an exome-wide significance threshold of *α* = 1.25 × 10^−7^ for power calculations. We calculated power as previously described^76^.

The ranges given in the main text (75,000–185,000 disease cases) represent the numbers from the power calculations for *INSR* (the drug target with the highest observed effect size) and *IGF1R* (the drug target with the lowest observed effect size other than *KCNJ11* and *ABCC8*). We excluded *KCNJ11* and *ABCC8* from this reported range, given that a mixture of risk-increasing and risk-decreasing variants in these genes probably diluted their burden signals. We did not account for uncertainty in estimated odds ratios or aggregate variant frequency in these calculations, as no genes had 95% confidence intervals that that did not overlap odds ratio = 1.

### Interpretation of suggestive associations

We quantified the PPA for nonsynonymous variants observed in our dataset as a function of association strength measured by single-variant *P* values. We define a true association as a variant that, when studied in larger sample sizes, will eventually achieve statistical significance owing to a true odds ratio ≠ 1. We distinguish true associations from causal associations: causally associated variants are the subset of truly associated variants in which the variant itself is causal for the increase in disease risk, as opposed to being truly associated due to linkage disequilibrium (LD) with a different causally associated variant (that is, an ‘LD proxy’). An overview of the method that we developed for PPA calculations is provided in Extended Data Fig. 7, and a full description of the method is included in the Supplementary Methods. Here, we outline the steps in the approach.

First, for various single-variant *P*-value thresholds in the exome-sequencing analysis, we calculated the fraction of variants that reached this threshold with directions of effect concordant with those of an independent exome array study^10^. For example, 61.3% of nonsynonymous variants within T2D GWAS loci that reached *P* < 0.05 in the exome-sequencing analysis had concordant directions of effect with the independent study, a fraction that decreased (as expected) for higher *P*-value thresholds (for example, 49.4% at *P* > 0.5) or when only variants outside of T2D GWAS loci were analysed (51.9% at *P* < 0.05).

Second, we derived an equation to convert the fraction of concordant associations to an estimated proportion of true associations. This value provides a PPA estimate, as a function of *P* value, for an arbitrary variant in the set initially used to calculate direction of effect concordances. We computed separate mappings for arbitrary nonsynonymous variants (using all exome-wide nonsynonymous variants) and one for nonsynonymous variants within GWAS loci (using only nonsynonymous variants within the 94 T2D GWAS loci). We note that the mapping produced from our analysis applies only to the results from the current study: because other studies have different sample sizes and may apply different statistical tests, the mapping would need to be recomputed to interpret the associations of other studies using the same method.

Third, we converted PPA estimates to estimates of the posterior probability of causal associations (PPA_c_). This conversion requires estimates of the fraction of coding variant associations that are causal (as opposed to LD proxies). We explored several values for this parameter, as described in the Supplementary Methods and shown in Extended Data Fig. 8.

Fourth, we extended PPA estimates to incorporate gene-specific priors by mapping posterior odds of causal association (PO_c_) to a Bayes factor for causal association (BF_c_). This calculation requires a set of training variants with a known prior. For this training set, we use nonsynonymous variants within GWAS loci and modelling assumptions for their prior. Details of this model are described in the Supplementary Methods and a sensitivity analysis of its assumptions is shown in Extended Data Fig. 8.

Finally, as a preliminary estimate of a principled prior likelihood for genes in the mouse NIDD gene set, we estimated the proportion of non-null associations across all genes in the set. To use true prior data (rather than associations from the current study), we calculated gene-level *P* values for each gene in the set using the MAGENTA^74^ algorithm applied to a recent transethnic T2D GWAS^13^. We then used a previously developed approach^40,77^ that models the distribution of observed *P* values as a mixture of uniform (representing the null distribution) and beta (representing the non-null distribution) distributions, yielding a prior value of 23.2%.

Our PPA_c_ calculations currently have several limitations. They apply only to single-variant associations and not (yet) to gene-level associations; extending them to apply to gene-level associations would avoid the possibility of conflicting results among variants within a gene but would require larger-scale gene-level replication data than that we had available in the current analysis. Additional work will also be needed to generate data and develop methods to estimate objective rather than subjective gene priors (researchers can often overestimate evidence of disease relevance for genes in which they have invested considerable effort), to reduce dependence of our conclusions on modelling assumptions (Extended Data Fig. 8) and to explore the extent to which the large number of variant associations that we predict from our data localize to specific gene or variant functional annotations^78^.

### Reporting summary

Further information on research design is available in the Nature Research Reporting Summary linked to this paper.

## Online content

Any methods, additional references, Nature Research reporting summaries, source data, extended data, supplementary information, acknowledgements, peer review information; details of author contributions and competing interests; and statements of data and code availability are available at 10.1038/s41586-019-1231-2.

### Supplementary information

Supplementary Information This file contains Supplementary Methods, Supplementary Tables 2, 3, 8, 10-17, 19, Supplementary Figures 1-21, a full list of consortia members and their affiliations and Supplementary references.

Reporting Summary

Supplementary Tables This file contains Supplementary Tables 1, 4, 5-7, 9 and 18.

Supplementary Data This file contains the software program.

## Figures and Tables

**Extended Data Table 1:**
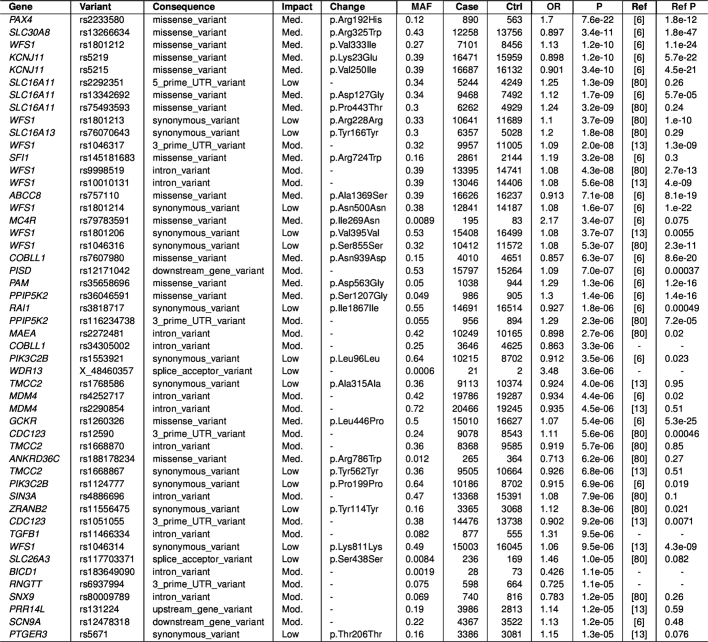
Most significant single-variant associations from exome-sequencing analysis

The most significant results from exome-sequencing single-variant association analyses are shown (*n* = 45,231 individuals). Gene, the closest gene to the variant. Variant, a unique identifier for the variant within our exome-sequencing analysis. Consequence, the predicted consequence of the variant, defined by sequence ontology annotation and produced using VEP. Impact, the effect of the variant, as predicted using VEP (Med, medium; Mod, modifier). Change, the predicted protein change, defined according to the ‘best guess’ transcript as described in the Methods. MAFs are calculated as the maximum across all ancestries. Case, the number of samples with T2D carrying the variant. Ctrl, the number of samples without T2D carrying the variant. OR, the odds ratio, calculated from the Firth analysis. P, the *P* value, calculated from the (two-sided) EMMAX analysis. Ref P: the *P* value of the variant in one of three previous GWAS or exome array analyses, referenced in the Ref column^6,13,79^.

**Extended Data Table 2:**
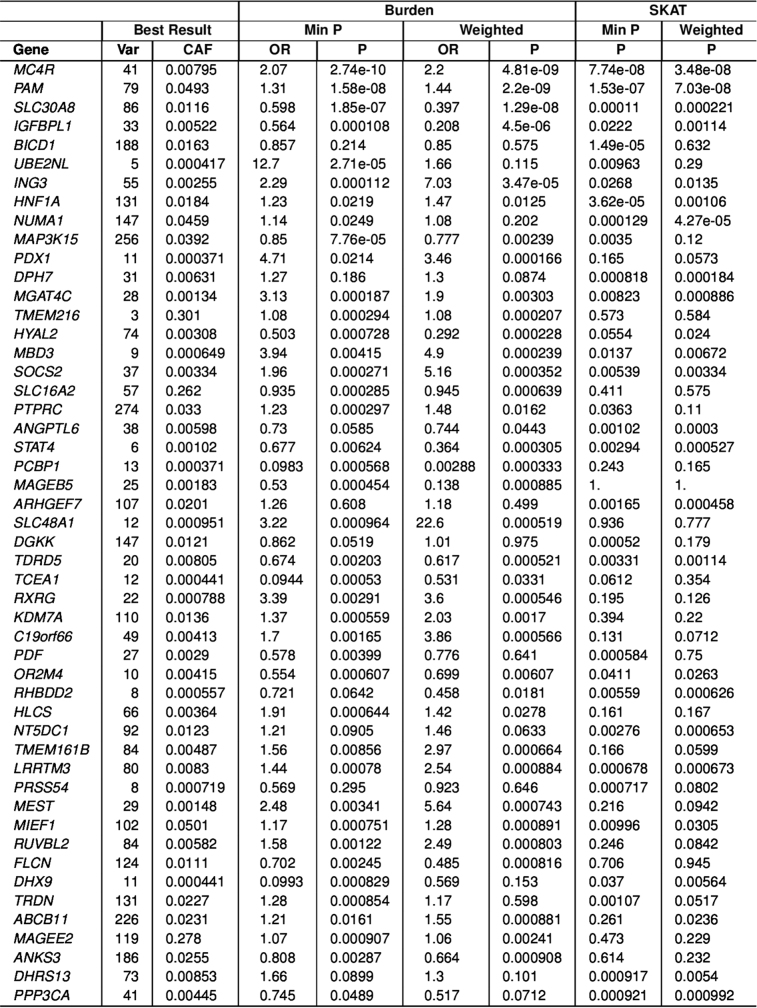
Most significant gene-level associations from exome-sequencing analysis

The most significant results from gene-level analyses are shown (*n* = 43,071 unrelated individuals). Genes are ranked according the (two-sided) minimum *P* value achieved across the four gene-level analyses. Gene, a unique identifier for the gene within our exome-sequencing analysis. Var (CAF), the number of alleles (combined allele frequency (CAF) of variants) in the mask achieving the strongest association across the four tests (that is, the 0/5 1% mask for the weighted test, or the mask with the minimum *P* value for the minimum *P*-value test). Burden, results from the (two-sided) burden analysis. SKAT, results from the (two-sided) SKAT analysis. Min P, results from the (two-sided) minimum *P*-value analysis. Weighted, results from the (two-sided) weighted analysis. OR, the odds ratio as estimated from the burden analysis. P, the (two-sided) *P* value for the indicated analyses.

## Data Availability

Sequence data and phenotypes for this study are available via the database of Genotypes and Phenotypes (dbGAP) and/or the European Genome-phenome Archive, as indicated in Supplementary Table 1.

## Code availability

Available for download are scripts for calculating the minimum *P*-value gene-level test, gene set enrichment analyses and the proportion of true associations as a function of variant *P* values.
