# Supplementary material for: Exome sequencing of 20,791 cases of type 2 diabetes and 24,440 controls
Source: Nature. 2019 May 22;570(7759):71–6. doi: 10.1038/s41586-019-1231-2 (PMC6699738; doi:10.1038/s41586-019-1231-2)
Supplement: Supplementary file 1 — This file contains Supplementary Methods, Supplementary Tables 2, 3, 8, 10-17, 19, Supplementary Figures 1-21, a full list of consortia members and their affiliations and Supplementary references. [file 41586_2019_1231_MOESM1_ESM.pdf]

nature
SUPPLEMENTARY INFORMATION
https://doi.org/10.1038/s41586-019-1231-2
In the format provided by the authors and unedited.

# Exome sequencing of 20,791 cases of type 2 diabetes and 24,440 controls

Jason Flannick[1,2,3,4*], flannick@broadinstitute.orgJosep M. Mercader[1,4,5,6,50,158], Christian Fuchsberger[7,8,9,158],
Miriam S. Udler[1,4,5,6,50,158], Anubha Mahajan[10,11,158], Jennifer Wessel[12,13,14],
Tanya M. Teslovich[15], Lizz Caulkins[1,4], Ryan Koesterer[1,4],
Francisco Barajas-Olmos[16], Thomas W. Blackwell[7,9], Eric Boerwinkle[17,18], Jennifer A. Brody[19], Federico Centeno-Cruz[16], Ling Chen[6,50], Siying Chen[7,9], Cecilia Contreras-Cubas[16], Emilio Córdova[16],
Adolfo Correa[20], Maria Cortes[21], Ralph A. DeFronzo[22], Lawrence Dolan[23], Kimberly L. Drews[24],
Amanda Elliott[1,4,6,50], James S. Floyd[25], Stacey Gabriel[21],
Maria Eugenia Garay-Sevilla[26,27], Humberto García-Ortiz[16], Myron Gross[28], Sohee Han[29], Nancy L. Heard-Costa[30,31], Anne U. Jackson[7,9],
Marit E. Jørgensen[32,33,34], Hyun Min Kang[7,9], Megan Kelsey[24],
Bong-Jo Kim[29], Heikki A. Koistinen[35,36,37], Johanna Kuusisto[38,39],
Joseph B. Leader[40], Allan Linneberg[41,42,43], Ching-Ti Liu[44], Jianjun Liu[45,46,47], Valeriya Lyssenko[48,49], Alisa K. Manning[50,51], Anthony Marcketta[15],
Juan Manuel Malacara-Hernandez[26,27], Angélica Martínez-Hernández[16],
Karen Matsuo[7,9], Elizabeth Mayer-Davis[52], Elvia Mendoza-Caamal[16],
Karen L. Mohlke[53], Alanna C. Morrison[54], Anne Ndungu[10],
Maggie C. Y. Ng[55,56,57], Colm O'Dushlaine[15], Anthony J. Payne[10],
Catherine Pihoker[58], Broad Genomics Platform[59], Wendy S. Post[60],
Michael Preuss[61], Bruce M. Psaty[62,63,64,65,66], Ramachandran S. Vasan[31,67],
N. William Rayner[10,11,68], Alexander P. Reiner[69], Cristina Revilla-Monsalve[70], Neil R. Robertson[10,11], Nicola Santoro[71], Claudia Schurmann[61],
Wing Yee So[72,73,74], Xavier Soberón[16], Heather M. Stringham[7,9],
Tim M. Strom[75,76], Claudia H. T. Tam[72,73,74], Farook Thameem[77],
Brian Tomlinson[72], Jason M. Torres[10], Russell P. Tracy[78,79],
Rob M. van Dam[46,47,80], Marijana Vujkovic[81], Shuai Wang[44], Ryan P. Welch[7,9], Daniel R. Witte[82,83], Tien-Yin Wong[84,85,86], Gil Atzmon[87,88,89], Nir Barzilai[87,89], John Blangero[90,91], Lori L. Bonnycastle[92],
Donald W. Bowden[55,56,57],
John C. Chambers[93,94,95], Edmund Chan[46], Ching-Yu Cheng[96],
Yoon Shin Cho[97], Francis S. Collins[92], Paul S. de Vries[54],
Ravindranath Duggirala[90,91], Benjamin Glaser[98], Clicerio Gonzalez[99],
Ma Elena Gonzalez[100], Leif Groop[48,101], Jaspal Singh Kooner[102],
Soo Heon Kwak[103], Markku Laakso[38,39], Donna M. Lehman[22], Peter Nilsson[104],
Timothy D. Spector[105], E. Shyong Tai[46,47,85], Tiinamaija Tuomi[101,106,107,108],
Jaakko Tuomilehto[109,110,111,112], James G. Wilson[113],
Carlos A. Aguilar-Salinas[114], Erwin Bottinger[61], Brian Burke[24], David J. Carey[40], Juliana C. N. Chan[72,73,74], Josée Dupuis[31,44], Philippe Frossard[115],
Susan R. Heckbert[116,117], Mi Yeong Hwang[29], Young Jin Kim[29],
H. Lester Kirchner[40], Jong-Young Lee[118], Juyoung Lee[29], Ruth J. F. Loos[61,119],
Ronald C. W. Ma[72,73,74], Andrew D. Morris[120], Christopher J. O'Donnell[3,121,122,123],
Colin N. A. Palmer[124], James Pankow[125], Kyong Soo Park[102,126,127],
Asif Rasheed[115], Danish Saleheen[81,115], Xueling Sim[47], Kerrin S. Small[105],
Yik Ying Teo[47,128,129], Christopher Haiman[130], Craig L. Hanis[131],
Brian E. Henderson[130], Lorena Orozco[16], Teresa Tusié-Luna[114,132],
Frederick E. Dewey[15], Aris Baras[15], Christian Gieger[133,134],
Thomas Meitinger[75,76,135], Konstantin Strauch[133,136], Leslie Lange[137],
Niels Grarup[138], Torben Hansen[138,139], Oluf Pedersen[138], Philip Zeitler[140],
Dana Dabelea[141], Goncalo Abecasis[7,9], Graeme I. Bell[26,27], Nancy J. Cox[142], Mark Seielstad[143,144], Rob Sladek[145,146,147], James B. Meigs[21,50,148],
Steve S. Rich[149], Jerome I. Rotter[150,151,152], DiscovEHR Collaboration[59], CHARGE[59], LuCamp[59], ProDiGY[59], GoT2D[59], ESP[59], SIGMA-T2D[59],
T2D-GENES[59], AMP-T2D-GENES[59], David Altshuler[1,4,6,50,153,154,155],
Noël P. Burtt[1,4], Laura J. Scott[7,9], Andrew P. Morris[10,156], Jose C. Florez[1,4,5,6,50], Mark I. McCarthy[10,11,157] & Michael Boehnke[7,9] A list of authors and their affiliations appears in the online version of the paper

# Exome sequencing of 20,791 type 2 diabetes cases and 24,440 controls

## Supplementary Information

## Contents


**1 Supplementary Methods** **3**
1.1 Sample selection . . . 3
1.2 Data generation . . . 3
1.2.1 Sample Sequencing . . . 3
1.2.2 Variant calling and quality control . . . 4
1.2.3 Additional quality control for association analysis in sequence data . . . 5
1.2.4 Variant annotation . . . 5
1.3 Power analysis . . . 6
1.4 Single-variant analysis in sequence data . . . 6
1.4.1 Subgroup-level analysis and quality control . . . 6
1.4.2 Single-variant meta-analysis . . . 7
1.4.3 Additional analysis of rs145181683 . . . 7
1.5 Gene-level analysis in sequence data . . . 8
1.5.1 Allelic mask creation . . . 8
1.5.2 Additional variant quality control . . . 8
1.5.3 Mask-level analysis . . . 9
1.5.4 Consolidation of tests across masks . . . 9
1.5.5 Gene-level analysis near T2D GWAS signals . . . 11
1.5.6 Further exploration of significant gene-level associations . . . 11
1.6 Replication of gene-level associations . . . 12
1.6.1 Analysis of exomes from the Geisinger Health System (GHS) . . . 12
1.6.2 Analysis of exomes from the CHARGE consortium . . . 13
1.6.3 Meta-analysis with CHARGE and GHS . . . 13
1.6.4 Investigation of the *UBE2NL* association . . . 13
1.6.5 Evaluation of directional consistency between exome sequence, CHARGE, and GHS analyses . . . 14
1.7 Gene set analysis in sequence data . . . 14
1.7.1 Generation of candidate T2D-relevant genes sets . . . 14
1.7.2 Gene set testing strategy . . . 15
1.7.3 Sensitivity analysis of gene matching strategy . . . 15
1.8 Application of gene-level associations . . . 16
1.8.1 Use of gene-level associations to predict effector genes . . . 16
1.8.2 Use of gene-level associations to predict direction of effect . . . 17

1.9 Imputed GWAS analysis . . . 17
1.9.1 Aggregation and generation of SNP array data . . . 17
1.9.2 Analysis of SNP array data . . . 18
1.9.3 Gene set analysis using SNP array data . . . 18
1.10 LVE analysis . . . 18
1.10.1 LVE calculations . . . 18
1.10.2 Prediction of LVE explained by the top 100 and top 1000 gene-level associations . . 20
1.10.3 Estimated power to detect gene-level associations with T2D drug targets . . . 20
1.11 Interpretation of suggestive associations . . . 20
1.11.1 Estimated fraction of true associations . . . 20
1.11.2 Probability of causal association . . . 22
1.11.3 Incorporation of prior likelihood into posterior probability estimations . . . 22
1.11.4 Inference of Bayes factors from GWAS variant posteriors . . . 23
1.11.5 Sensitivity of $PPA_c$ to modeling parameters and other limitations of the calculations 24
1.11.6 Estimation of prior for genes in the Mouse NIDD gene set . . . 24

**2 Supplementary Tables 26**

**3 Supplementary Figures 38**

**4 List of consortia members 57**
4.1 AMP-T2D-GENES . . . 57
4.2 T2D-GENES . . . 57
4.3 SIGMA . . . 58
4.4 GoT2D . . . 58
4.5 LuCAMP . . . 59
4.6 PRODiGY . . . 59
4.7 ESP . . . 59
4.7.1 BroadGO . . . 59
4.7.2 HeartGO . . . 59
4.7.3 ISGS and SWISS . . . 60
4.7.4 LungGO . . . 60
4.7.5 SeattleGO . . . 60
4.7.6 WHISP . . . 60
4.7.7 NHLBI GO ESP Project Team . . . 61
4.8 CHARGE . . . 61
4.9 DiscovEHR . . . 62
4.10 Broad Genomics Platform . . . 62
4.11 Affiliations . . . 63

# 1 Supplementary Methods

## 1.1 Sample selection

We drew samples for exome sequencing from six consortia (**Supplementary Table 1**):

1. The T2D-GENES (Type 2 Diabetes Genetic Exploration by Next-generation sequencing in multi-Ethnic Samples) consortium, an NIDDK-funded international research consortium seeking to identify genetic variants for T2D through multiethnic sequencing studies [1]
2. The Slim Initiative in Genomic Medicine for the Americas: Type 2 Diabetes (SIGMA T2D), an international research consortium funded by the Carlos Slim Foundation to investigate genetic risk factors of T2D within Mexican and Latin American populations and translate those findings to improved methods of treatment and prevention [2].
3. The Genetics of Type 2 Diabetes (GoT2D) consortium, an NIDDK-funded international research consortium seeking to understand the allelic architecture of T2D through low-pass whole-genome sequencing, deep exome sequencing, and high-density SNP genotyping and imputation [1].
4. The Exome Sequencing Project (ESP), an NHLBI-funded research consortium to investigate novel genes and mechanisms contributing to heart, lung, and blood disorders through whole exome sequencing [3].
5. The Lundbeck Foundation Centre for Applied Medical Genomics in Personalised Disease Prediction, Prevention, and Care (LuCamp) study, which researches whole exome variation in Danish metabolic diseases including diabetes [4].
6. The ProDiGY (Progress in Diabetes Genetics in Youth) consortium, an NIDDK-funded research consortium to investigate genetic variants for childhood T2D.

Each consortium provided individual-level information on T2D case-control status according to study-specific criteria as well as key covariates including age, sex, and BMI (**Supplementary Table 1**). In addition, several consortia provided data on fasting glucose, 2-hour glucose following glucose challenge, and use of anti-hyperglycemic medications. We excluded as controls individuals with a 2-hour glucose value $\geq$ 11.1 mmol/L (which meets diagnostic criteria for T2D) or with any two of the following features suggestive of T2D: fasting glucose $\geq$ 7 mmol/L, hemoglobin A1c $\geq$ 6.5%, or recorded as taking an anti-hyperglycemic medication. We opted to require two of the previous features since there is room for error in each: fasting values used in T2D diagnostic criteria are required to represent at least an eight-hour fast, accuracy varies across hemoglobin A1c assays, and anti-glycemic medications are occasionally taken by non-diabetic individuals.

All individuals provided informed consent and all samples were approved for use by their home institution's institutional review board or ethics committee, as previously reported [1–4]. Samples newly sequenced at The Broad Institute as part of T2D-GENES, SIGMA, and ProDiGY are covered under Partners Human Research Committee protocol # 2017P000445/PHS "Diabetes Genetics and Related Traits".

## 1.2 Data generation

### 1.2.1 Sample Sequencing

For roughly half the study participants (some of T2D-GENES [1] and all of GoT2D [1], SIGMA-T2D [2], LuCAMP [4], and ESP [3]), exome sequence data were available from previous studies. For these individuals (**Supplementary Table 1**), we obtained access to and aggregated BAM files containing unaligned sequence reads, which were generated and analyzed as previously described [1–4].

For the remaining participants, de-identified DNA samples were sent to the Broad Institute in Cambridge, MA, USA where samples with (a) sufficient total DNA quantity and minimum DNA concentrations (as estimated by Picogreen) and (b) high quality genotypes (as measured by a 24 SNP Sequenom iPLEX assay) were advanced for subsequent sequencing. Library construction was performed as previously described [5] with some slight modifications. Initial genomic DNA input into shearing was reduced from 3$\mu$g to 50ng in 10$\mu$L of solution and enzymatically sheared. For adapter ligation, dual-indexed Illumina paired end adapters were replaced with palindromic forked adapters with unique 8 base index sequences embedded within the adapter and added to each end.

In-solution hybrid selection was performed using the Illumina Rapid Capture Exome enrichment kit with 38Mb target territory (29Mb baited), including 98.3% of the intervals in the Refseq exome database. Dual-indexed libraries were pooled into groups of up to 96 samples prior to hybridization, with liquid handling automated on a Hamilton Starlet Liquid Handling system. The enriched library pools were quantified via PicoGreen after elution from streptavidin beads and then normalized to a range compatible with sequencing template denature protocols.

Following sample preparation, the libraries prepared using forked, indexed adapters were quantified using quantitative PCR (KAPA Biosystems), normalized to 2 nM, and pooled by equal volume using the Hamilton Starlet. Pools were then denatured using 0.1 N NaOH. Denatured samples were diluted into strip tubes using the Hamilton Starlet.

Cluster amplification of the templates was performed according to the manufacturer's protocol (Illumina) using the Illumina cBot. Flowcells were sequenced on HiSeq 4000 Sequencing-by-Synthesis Kits and then analyzed using RTA2.7.3.

#### 1.2.2 Variant calling and quality control

Sequencing reads for all samples (both newly sequenced and previously sequenced) were processed and aligned to the human genome (build hg19) using the Picard (broadinstitute.github.io/picard/), BWA [6], and GATK [7] software packages, following best-practice pipelines; data from previously published studies were treated the same as data from the new study (i.e. beginning from unaligned reads) to ensure uniformity of processing. Single nucleotide and short indel variants were then called using a series of GATK commands (version nightly-2015-07-31-g3c929b0): ApplyRecalibration, CombineGVCFs, CombineVariants, GenotypeGVCFs, HaplotypeCaller, SelectVariants, and VariantFiltration. Variants were called within 50bp of any region targeted for capture in any sequenced cohort.

We computed hard calls (the GATK-called genotypes but set as missing at a genotype quality [GQ]$<$20 threshold) and dosages (the expected alternate allele count, defined as $\Pr(RX|data) + 2\Pr(XX|data)$, where $R$ is the reference allele and $X$ the alternative allele) for each individual at each variant site. We used hard calls for quality control and dosages in downstream association analyses. We computed dosages on the X chromosome (outside of the pseudo-autosomal region) accounting for sex, treating males as haploid.

To perform data quality control, we first calculated a range of metrics measuring sample sequencing quality. We then stratified samples by ancestry and sequence capture technology and excluded from further analysis samples that were outliers according to any metric, based on visual inspection by comparison to other samples within the same stratum (**Supplementary Table 1**). A full list of metrics used for exclusion and the number of samples excluded based on each metric is shown in **Supplementary Table 2**.

After exclusion of samples, we calculated an additional set of variant metrics and excluded any variant with overall call rate $<$0.3, heterozygosity of 1, or heterozygote allele balance of 0 or 1 (i.e. 100% or 0% of reads called non-reference for heterozygous genotypes). We intentionally chose these non-stringent initial variant quality-control thresholds due to the heterogeneity of capture and sequencing technologies used in our study; we performed much more stringent variant quality control during single-variant or gene-level association analysis. We refer to the 49,484 samples and 7.02M variants passing this first round of

non-stringent quality control as the “clean” dataset.

### 1.2.3 Additional quality control for association analysis in sequence data

Following initial sample and variant quality control, we performed additional exclusions of samples from association analysis. First, we computed a set of “transethnic” SNPs for use in identity-by-descent (IBD) and principal component (PC) analysis. We began this analysis with variants in the clean dataset (a) with genotype call rate $>$95%, (b) with minor allele frequency (MAF) $>$1% in each ancestry, and (c) further than 250Kb from the HLA region or an established T2D association signal. We LD-pruned variants using PLINK [8] based on maximum $r^2 = 0.2$ (parameters `-indep-pairwise 50 5 0.2`). We used the remaining 171K variants to estimate pairwise individual IBD using PLINK, and the top 10 PCs of genetic ancestry using EIGENSTRAT [9]. For each pair of individuals with IBD$>$0.9, we excluded the individual with the lower call rate (337 duplicate exclusions in **Extended Data 2**). We then excluded, for each of the five ancestries, any individual who appeared, based on visual inspection of the first two transethnic PCs, to lie outside of the main PC cluster corresponding to that ancestry (133 ethnic outliers in **Extended Data 2**). Finally, we used the subset of transethnic ancestry SNPs on the X chromosome to compare genetic sex to reported sex, using PLINK, and excluded all discordant individuals (273 sex discordances in **Extended Data 2**).

At this stage we also excluded the 3,510 childhood diabetes cases from the SEARCH and TODAY studies. Although these samples had no matched controls, at the outset of our study we hoped to include them as cases in both single-variant and gene-level analysis, using the other samples as “pseudo-matched” controls with PCs or linear mixed models used to adjust for ancestry differences. However, while single-variant association statistics (computed via a meta-analysis of ancestry-level associations) remained well-calibrated with these studies included (**Supplementary Figure 17ab**), gene-level analysis yielded a dramatically inflated quantile-quantile (QQ) plot (**Supplementary Figure 17cd**). These results suggested that, while the samples in our study may provide suitable matched controls for common-variant analyses, they are inadequate for rare-variant analyses — consistent with previous simulations [10]. Exclusion of the SEARCH and TODAY study samples, samples failing quality control, and variants that became monomorphic as a result of these sample exclusions, yielded an “analysis” dataset of 45,231 individuals and 6.33M variants.

After these three rounds of sample exclusions, we identified five sets of ancestry-specific “ancestry” SNPs. We used the same procedure as for the transethnic SNPs (described above), except that we applied the MAF threshold only within the appropriate ancestry. We used these ancestry SNPs to estimate, for each ancestry, pairwise IBD values, genetic relatedness matrices (GRMs), and PCs for use in downstream association analysis.

Additionally, from the IBD values, we generated a list of unrelated individuals within each ancestry by excluding the individual with the lower call rate in any pair of individuals with IBD$>$0.3 (leading to 2,157 excluded individuals). The resulting “unrelated analysis” set consisted of 43,090 individuals (19,828 cases and 23,262 controls) and yielded 6.29M non-monomorphic variants. We used this set of individuals and variants for single-variant and gene-level tests (described below) that required an unrelated set of individuals for analysis.

### 1.2.4 Variant annotation

We annotated variants with the ENSEMBL Variant Effect Predictor [11] (VEP, version 87). Annotations were produced for all ENSEMBL transcripts with the `-flag-pick-allele` option used to assign a “best guess” annotation to each variant according to the following ordered criteria for transcripts [12]: transcript support level (TSL, i.e. supported by mRNA), biotype (i.e. protein_coding), APPRIS isoform annotation (i.e. principal), deleteriousness of annotation (i.e. prefer transcripts with higher impact annotations), CCDS [13] status of transcript (i.e. a high-quality transcript set), canonical status of transcript, and transcript

length (i.e. longer preferred). We used the VEP LofTee (https://github.com/konradjk/loftee) and dbNSFP (version 3.2) [14] plugins to generate additional bioinformatic predictions of variant deleteriousness; from the dbNSFP plugin, we took annotations from 15 different bioinformatic algorithms (listed in **Extended Data 6**) as well as the mCAP [15] algorithm. As these annotations were not transcript-specific, we assigned them to all transcripts for the purpose of downstream analysis.

While we incorporated both transcript-level and gene-level annotations into gene-level analysis (see below), all single-variant analyses reported in the manuscript or figures are annotated using the "best guess" annotation for each variant.

## 1.3 Power analysis

We carried out power calculations [16] for single-variant or gene-level tests assuming a disease prevalence of 0.08 to convert population frequencies and odds ratios [ORs] to case and control frequencies, and a sample size (19,828 cases and 23,262 controls) from an analysis of only unrelated individuals. Our calculations assumed that allelic effects were homogeneous across ancestries.

## 1.4 Single-variant analysis in sequence data

### 1.4.1 Subgroup-level analysis and quality control

To perform single-variant association analysis, we stratified samples by cohort of origin and sequencing technology (i.e. samples from the same cohort but sequenced at different times were analyzed separately). Samples from the ESP, SIGMA, and GoT2D consortia were treated slightly differently, due to the large number of cohorts within them. We stratified ESP samples by ancestry (rather than cohort) but not further by sequencing technology, instead using sequencing technology as a covariate in downstream analysis. We stratified SIGMA samples by sequencing technology but not further by cohort; cohort-stratified analyses produced results highly concordant with those produced by analyses stratified only by sequencing technology. We did not stratify GoT2D samples, as all were sequenced via the same technology. This procedure yielded 25 distinct sample subgroups (**Extended Data 3**).

Within each of the 25 sample subgroups, we performed additional variant quality control beyond that used to create the "clean" dataset. For each cohort, we excluded variants according to "basic filters" on subgroup-specific measures of call rate, Hardy-Weinberg equilibrium (HWE), differential case-control missingness, and alternate allele genotype quality. Specific criteria for these filters — which, particularly for multiallelic and X-chromosome variants, were strict — are shown in **Extended Data 3**.

For each of the 25 sample subgroups, we then conducted two single-variant association analyses. In both single-variant analyses, we collapsed all non-reference alleles at multiallelic sites into a single "non-reference" allele.

First, we analyzed all (including related) samples via the (two-sided) EMMAX test [17], as implemented in the EPACTS (genome.sph.umich.edu/wiki/EPACTS) software package, using the GRM computed from the ancestry-specific "ancestry" variants. We included in the model covariates for sequencing technology (where appropriate) but not for PCs of genetic ancestry. We did not include covariates for age, sex, or BMI.

Second, we analyzed unrelated samples via the (two-sided) Firth logistic regression test [18], also as implemented in EPACTS; we included in the model covariates for sequencing technology and for PCs of genetic ancestry (computed from the ancestry-specific "ancestry" variants). The number of PCs we included varied by subgroup; to select the PCs to be included, we regressed T2D status on sequencing technology and the first ten PCs, and we then included in the model any PC that demonstrated nominal ($p<0.05$) association with T2D (as well as all higher-order PCs).

For each of the $25\times2=50$ single-variant analyses, we inspected QQ plots of variant association statistics and quality control metrics for variants with the strongest associations. For subgroups for which these

metrics suggested an enrichment of association artifacts among the strongest associations, we developed stricter variant quality control filters beyond the "basic filters" used across all subgroups. In general, the degree of stringency necessary was inversely correlated with the date and quality of sequencing. In particular, the Ashkenazi subgroup from the T2D-GENES study showed heterogeneity in sequencing quality between cases and controls (owing to resequencing performed subsequent to the original study publication [1]) and required significant filters to remove artifactual associations. In addition, due to a significant imbalance between the number of cases and controls in the ESP studies, we excluded any variant from those subgroups with an association $p$-value less than 0.3 times the $p$-value from Fisher's exact test (under the assumption that, in these cases, covariates in the analysis were inducing statistical artifacts). By contrast, none of the newly sequenced subgroups required significantly stricter filters. The filters shown in **Extended Data 3** represent the final values at which we arrived. We verified that these filters led to a well-calibrated final analysis through inspection of QQ plots within and across ancestries (**Extended Data 4**).

#### 1.4.2 Single-variant meta-analysis

We then conducted a 25-group fixed-effect inverse-variance weighted meta-analysis for each of the Firth and EMMAX tests, using METAL [19]. We used EMMAX results for association $p$-values and Firth results for effect size estimates. For comparison, we conducted two additional meta-analyses with association Z-scores weighted by (a) sample-size and (b) the number of variant carriers. We found that the sample-size weighted meta-analysis had significantly reduced power to detect association for variants with frequencies that varied widely by sample subgroup; for example, 1,425 East-Asian individuals carried p.Arg192His in PAX4 (N=6,032; $p$=1.2$\times$10$^{-21}$) compared to only 28 carriers across all other ancestries (N=39,199; $p$>0.2), yielding an inverse-variance weighted meta-analysis $p$=7.6$\times$10$^{-22}$ and a sample-size weighted meta-analysis $p$=1.0$\times$10$^{-6}$. By contrast, the number-of-carrier weighted meta-analysis yielded similar results as the inverse-variance weighted meta-analysis. We elected to use the inverse-variance weighted method due to its widespread precedence [19]. We did not conduct random-effects meta-analyses.

#### 1.4.3 Additional analysis of rs145181683

To assess whether the rs145181683 variant in SFI1 ($p$=3.2$\times$10$^{-8}$ in the exome sequence analysis) represented a true novel association, we obtained association statistics from the 4,522 Latinos previously analyzed as part of an 8,214 sample Latino GWAS (published by the SIGMA-T2D consortium [2]) who did not overlap with the current study. Based on the OR (1.19) estimated in our analysis and the MAF (12.7%) in the replication sample, power was 91% to achieve $p$<0.05 under a one-sided association test. The observed evidence ($p$=0.90, OR=1.00) did not support rs145181683 as a true T2D association.

We investigated two potential reasons for the lack of replication evidence for rs145181683. First, we examined whether the original exome sequence association might have been due to a technical artifact. However, genotyping quality was high (call rate 98.0% across all samples, 100% in the cohorts responsible for the association) and the variant did not fail any quality control metrics. Second, we noted that the Latino samples we sequenced had significantly lower Native American ancestry than did the 4,522 Latino samples in which we sought replication, because of the manner in which they were ascertained [20]. We thus assessed whether the original rs145181683 association might in fact be tagging another causal variant enriched in Native Americans by extending the replication meta-analysis to all variants at the locus (rather than just rs145181683). In this meta-analysis, additional non-coding variants in the same locus (including rs149762669 and 22-31988846-G-A) remained genome-wide significant. The rs149762669 and rs145181683 variants are in partial linkage disequilibrium in the Mexican population ($r^2 = 0.48$) but not in other populations ($r^2 < 0.001$ all cases). In fact, while rs145181683 seems to be in enriched in Mexican individuals (Mexican MAF=0.15, European MAF=0.00055), rs149762669 appears to be common in both Mexican and European populations (Mexican MAF=0.32, European MAF=0.15). Thus, it is possible

that rs145181683 does not replicate because it tags another (possibly non-coding) causal variant such as rs149762669. Further fine-mapping and replication efforts will be necessary to test this hypothesis.

## 1.5 Gene-level analysis in sequence data

### 1.5.1 Allelic mask creation

We first filtered variants (or, more accurately, alleles, since in contrast to single-variant analysis, we treated multiallelic variants as collections of independent biallelic variants) according to seven different annotation “masks”, ranked in order of increasing deleteriousness. The strongest mask consisted of alleles predicted to cause loss of function by the LofTee algorithm (https://github.com/konradjk/loftee), while weaker masks also included alleles predicted deleterious by progressively fewer bioinformatic algorithms. Each mask included all alleles in higher ranked masks as well as additional alleles specific to the mask. In the two lowest ranked masks (the 1/5 1% and 0/5 1% masks, which included alleles predicted deleterious by one or zero tools, respectively), we filtered alleles specific to each mask according to allele frequency using a cutoff of MAF=1%, with MAF computed as the maximum MAF across the five ancestries of the study. A full list and definitions of masks are shown in **Extended Data 6**; the criteria listed in the figure are for alleles specific to each mask.

To validate that the severity ordering of masks corresponded to an increasing likelihood that an allele in the mask was deleterious, we used previously published data assessing the extent to which missense variants in the gene *PPARG* impede adipocyte differentiation (i.e. were annotated as causing *PPARG* loss of function). These data showed a trend whereby alleles in more severe masks had lower predicted functionality (**Supplementary Figure 5**).

For each mask, we grouped alleles by gene according to VEP annotations of impacted transcript; we assigned variants in transcripts of multiple genes to all such genes. For each gene, we created up to three groupings of alleles, corresponding to different transcript sets of the gene. First, the “best” grouping consisted of alleles in the mask according to the “best guess” allele-level annotations. Second, the “all” grouping consisted of alleles in the mask according to any transcript of the gene. Third, the ”filter” grouping consisted of alleles in the mask according to protein-coding transcripts of the gene with TSL$<$3. For many genes, two or more of these allele groupings were identical.

Additionally, we assigned mask-specific weights to alleles according to their aggregate predicted deleteriousness. To calculate weights, we used a previously published model [21] in which missense variants are a mixture of fully benign variants and fully loss-of-function variants, with a parameter $0 \leq x \leq 1$ determining the fraction of loss-of-function variants. We assumed all alleles in the LofTee mask were full loss-of-function variants ($x = 1$) and that all synonymous alleles were fully benign ($x = 0$). We then calculated the (binned) frequency distribution, truncated at MAF$<$1%, of biallelic LofTee and biallelic synonymous alleles, using these as reference distributions of the frequencies of loss-of-function and benign alleles, respectively. For each mask, we then calculated the binned and truncated frequency distribution for alleles specific to the mask (**Supplementary Figure 6**) and estimated a value for x (by enumerating and testing a range of possible values between 0 and 1) that maximized the likelihood of the observed frequency distribution. We then used the estimated values of $x$ for allele weights, as shown in **Extended Data 6**. Because each mask consisted not only of alleles specific to the mask but also of alleles present in higher ranked masks, alleles within any given mask had a range of weights.

### 1.5.2 Additional variant quality control

Prior to running gene-level tests, we performed additional quality control on sample genotypes. For each of the 25 sample subgroups (the same subgroups used for single-variant analysis), we identified all variants with low subgroup-specific call rates, high subgroup-specific deviations from HWE, or high subgroup-specific differences between case and control call rates (specific criteria are shown in **Extended Data**

**6**). For each variant failing any of these subgroup-specific criteria, all genotypes for individuals in the subgroup were set as "missing"; for multiallelic variants, all subgroup genotypes were set as missing if any allele failed any quality control criterion.

#### 1.5.3 Mask-level analysis

We then conducted a series of tests across the masks. We used a burden test and SKAT [22], both two-sided and implemented in the EPACTS software package. The burden test assumes that the effect sizes of all analyzed variants are the same, while the SKAT test allows effect sizes to vary [23]. We conducted each test across all unrelated individuals pooled together (i.e. in contrast to single-variant analysis, we performed a "mega-analysis" rather than a meta-analysis) and included 10 PC covariates (computed from the transethnic ancestry SNPs) as well as covariates for sample subgroup (the same as defined in single-variant analysis) and sequencing technology. In support of this mega-analysis analysis strategy, single-variant associations tests (using the same logistic regression test and covariates as gene-level burden tests) showed broad correlation between mega- and meta-analysis strategies (**Supplementary Figure 18**). We did not include covariates for age, sex, or BMI in our analysis, as they had little effect on our results.

We implemented subgroup-specific genotype filters (as defined in the previous quality control step) by modifying the EPACTS software to set specified genotypes to missing during association testing; we achieved allele-specific tests for multiallelic variants (i.e. in which only one allele was present in the mask) in a similar manner by setting non-reference genotypes to missing for samples that carried an allele outside of the mask. We also modified the EPACTS software to accept allele-specific weights by multiplying genotypes (or more accurately, genotype dosages) by the relevant weight prior to conducting the formal burden or SKAT analysis.

#### 1.5.4 Consolidation of tests across masks

Historically, exome sequencing studies have produced separate gene-level association results for each allelic mask. While straightforward to report, interpreting multiple *p*-values for each gene can be challenging. To address this challenge, we developed two methods to collapse association results across different allelic masks.

The first method ("weighted test") collapses associations under a model whereby the phenotypic effects of alleles are directly proportional to their bioinformatically estimated deleteriousness. In the "weighted burden" test, we used the sum of the weights of alleles carried by an individual as a predictor variable in place of the total number of alleles carried. In the "weighted SKAT" test, we multiplied the default weights used in the SKAT EPACTS implementation by the allelic weights we calculated. For these weighted tests we included all alleles in the 0/5 1% mask in the analysis.

Because bioinformatically predicted severity is an imperfect proxy to actual phenotypic severity, we developed a second method, the "minimum *p*-value test", to collapse associations across masks. We chose the minimum *p*-value test to provide a principled extension of an *ad hoc* but intuitive method to interpret multiple *p*-values for a given gene: take the smallest *p*-value observed across each mask and then correct for the effective number of tests performed for the gene.

To conduct these minimum *p*-value tests, we first ran the burden and SKAT analyses for each of the seven masks separately, following usual exome sequence analysis protocols by using no weights and including all alleles in each mask. For each gene, we then converted the seven *p*-values into a single *p*-value via the formula

$$1 - (1 - p_{min})^e$$

where $e$ is the effective number of independent tests performed across the masks. This number is variable across genes and, for each gene, depends on the specific correlation of variants across masks.

To estimate a specific value of $e$ for each gene, we applied a previous approach [24] originally developed to compute the effective number of independent $p$-values across a set of SNPs:

$$M - \sum_{i=1}^{M} \left[ I\left(\lambda_i > 1\right)\left(\lambda_i - 1\right) \right]$$

where in our case $M$ equals the number of masks (usually seven, except for genes that lack variants in one or more masks or for which two masks are identical), $\lambda_i$ are the eigenvalues of the $M \times M$ matrix of correlations among the $p$-values of the mask-level tests, and $I$ is an indicator function (taking the value of 1 if its argument is true and 0 otherwise). To compute the mask $p$-value correlation matrix, we followed the previous approach by first calculating (for each gene) the mask genotype correlation matrix (i.e., for each mask, producing a vector with the number of variants in the mask carried by each individual, and then calculating correlations of the vectors) and then transforming the genotype correlation matrix according to the previously empirically derived [24] polynomial equation:

$$y = 0.2982x^6 - 0.0127x^5 + 0.0588x^4 + 0.0099x^3 + 0.6281x^2 - 0.0009x$$

where $x$ is the measured correlation between the number of alleles carried and $y$ is the estimated correlation between $p$-values.

We used this approach to estimate gene-specific correlation matrices, rather than a single empirical $p$-value correlation matrix across all genes, because (a) the number of effective gene-level tests can vary widely across genes and (b) we wished to develop a method that could be applied even absent genome-wide statistic distributions. We note that the polynomial we used was initially developed to translate single-variant genotype correlations to $p$-value correlations, rather than aggregate genotype correlations to $p$-value correlations. However, in our analysis we predominantly applied it to the burden test, which (as for single-variant analysis) is a logistic regression of phenotype on aggregate genotype. To verify that this polynomial applied to $p$-values from burden tests in which individuals carry more than two alleles, we repeated the previous simulations [24] and observed the expected fit between aggregate genotype and burden test $p$-value correlation (**Supplementary Figure 19**).

By contrast, this polynomial may not be the best function to map aggregate genotype correlations to SKAT $p$-value correlations. Developing an improved mapping would require further work, including non-trivial simulations to interrogate the number of different genotype configurations that could enter a SKAT analysis. Such validation should take place in the context of a SKAT-focused future study.

Genomic control estimates ($\lambda_{gc} = 0.67$) and QQ plots (**Extended Data 7**) suggested that if anything the minimum $p$-value test was conservative for most genes. We further note that, even if our gene-level $p$-values were (more stringently) Bonferroni corrected for seven independent masks, the results of our study would remain largely unchanged: each of *SLC30A8*, *MC4R*, and *PAM* would still exceed exome-wide significance (for both the weighted and minimum $p$-value tests), and the gene set test results (described below) would remain nearly identical (as they are based on gene-level $p$-value ranks rather than absolute $p$-values). Future work could investigate the application of other methods previously developed to correct for correlated $p$-values [25, 26].

The application of two different methods for collapsing $p$-values across masks for each of two tests yielded four analyses for each gene, corresponding to a weighted burden analysis, a weighted SKAT analysis, an minimum $p$-value burden analysis, and an minimum $p$-value SKAT analysis. In fact, for each of the four analyses, multiple $p$-values were possible for each gene (corresponding to the different transcript sets used for annotation). To produce a single gene-level $p$-value for each of the four analyses, we thus collapsed (for each gene) the set of $p$-values across transcript sets into a single gene-level $p$-value using the same procedure as for the minimum $p$-value test (i.e. taking the minimum $p$-value across transcript sets and correcting for the effective number of tests performed).

We verified that the minimum $p$-value and weighted consolidation methods were both well-calibrated (**Extended Data 7**) and between them produced broadly consistent but distinct results: across the ten

most significantly-associated genes, $p$-values were nominally significant under both methods for eight genes but varied by one-to-three orders of magnitude (**Extended Data 8**).

#### 1.5.5 Gene-level analysis near T2D GWAS signals

In principle, a nearby common-variant association could lead to over- or under-estimation of the strength of a gene-level association [27]. To assess whether differential patterns of rare variation across common-variant haplotypes could significantly affect our gene-level results, we conducted two analyses. First, for 17 genes with common coding variant signals, [28] we conducted gene-level analysis conditional upon all common coding variants and found minimal differences between unconditional and conditional gene-level associations: all genes had conditional gene-level $p$-values within a factor of 1.5 of the unconditional $p$-values except for *PAM* (unconditional $p$-value $6.6\times10^{-6}$ times less than conditional $p$-value, expected from the inclusion of the known common variants p.Asp563Gly and p.Ser539Trp in gene-level analysis) and *SLC30A8* (conditional $p$-value 2.2 times less than unconditional $p$-value). Second, for each of the three genes that achieved exome-wide significant associations, we conducted gene-level burden tests of rare (MAF$<$1%) synonymous variants. Associations were statistically insignificant for *SLC30A8* ($p$=0.72) and *MC4R* ($p$=0.61) and nominally significant ($p$=0.036, OR=1.03) for *PAM*, far weaker than observed for either the unconditional (OR=1.44) or conditional (OR=1.22) analyses of rare nonsynonymous *PAM* variants. These analyses suggest that confounding from common-variant haplotypes is not primarily responsible for the associations observed in our gene-level analysis.

#### 1.5.6 Further exploration of significant gene-level associations

For our exome-wide significant gene-level associations (*MC4R*, *SLC30A8*, and *PAM*), we conducted additional gene-level analyses to dissect the aggregate signals observed. First, we performed tests for each mask separately, including only alleles specific to the mask (rather than all alleles), to understand whether the aggregate signal was observed in only one as opposed to multiple masks. Second, we performed tests by progressively removing alleles in order of lowest single-variant analysis $p$-value, to understand the (minimum) number of alleles that contributed statistically to the aggregate signal. Third, we performed tests conditional on each allele in sequence (i.e. calculating separate models with each individual allele as a covariate), with the resulting $p$-values compared to the full gene-level $p$-value, to assess the contribution of each allele individually to the signal.

These analyses showed the *MC4R* (combined MAF=0.79%; minimum $p$=$2.7\times10^{-10}$, OR=2.07 [1.65-2.59]) and *PAM* (combined MAF=4.9%; weighted $p$=$2.2\times109$, OR=1.44 [1.28-1.62]) gene-level signals are due largely — but not entirely — to effects from individual variants (p.Ile269Asn for *MC4R*, p.Asp563Gly and p.Ser539Trp for *PAM*). For *MC4R*, gene-level association decreased but remained nominally significant after removing p.Ile269Asn ($p$=$8.6\times10^{-3}$; **Supplementary Figure 7**). Similarly, as shown previously [29, 30], association was less significant after conditioning on sample BMI, both for the p.Ile269Asn single-variant signal ($p$=$1.0\times10^{-5}$) and the gene-level signal not attributable to p.Ile269Asn ($p$=0.035).

The gene-level signal in *PAM* remained nominally significant ($p$$<$0.05) even after removing the 35 strongest individually associated *PAM* variants, indicating a contribution from substantially more variants than p.Asp563Gly and p.Ser539Trp (**Supplementary Figure 8**). Cellular characterization of p.Asp563Gly and p.Ser539Trp recently identified a novel mechanism for T2D risk through altered insulin storage and secretion [31]. Our results provide many more genetic variants — identifiable only through sequencing [28] — that could be characterized for further insights into the T2D risk mechanism mediated by *PAM*.

By contrast, the *SLC30A8* signal (103 variants, combined MAF=1.4%, weighted $p$=$1.3\times10^{-8}$, OR=0.40 [0.28-0.55]) was not primarily driven by an individual variant (p.Arg325Trp [MAF$>$1%] was not included in gene-level analysis). The association was instead driven by 90 missense variants (weighted $p$=$3.9\times10^{-7}$) and remained nominally significant ($p$$<$0.05) even when we removed the 32 strongest individually associated SLC30A8 variants (**Supplementary Figure 9**).

To evaluate which ancestries contributed variants to *MC4R*, *SLC30A8*, and *PAM*, we calculated the proportion of variants in each signal unique to an ancestry and also compared the significance and direction of effect of each signal across ancestries. Across the three signals, 68.4% (287 of 419) of variants in total were unique to one ancestry (63.9% for *MC4R*, 67.0% for *SLC30A8*, and 71.6% for *PAM*). Each signal had direction of effect consistent across all five ancestries, and each signal achieved $p<0.05$ in at least two ancestries (*MC4R* in East-Asians and Hispanics; *SLC30A8* in all ancestries other than African-Americans; and *PAM* in Europeans, South-Asians, and Hispanics).

## 1.6 Replication of gene-level associations

### 1.6.1 Analysis of exomes from the Geisinger Health System (GHS)

We obtained gene-level association results previously computed from an analysis of 49,199 individuals (12,973 T2D cases and 36,226 controls) from the Geisinger Health System. We requested association summary statistics for the 50 genes with the strongest gene-level associations from our study (according to the lowest *p*-value observed across our four analyses); 44 genes had precomputed (two-sided) summary statistics available; pseudogene *UBE2NL* and X chromosome genes *MAP3K15*, *SLC16A2*, *MAGEB5*, *DGKK*, and *MAGEE2* were not available. Assuming (optimistically) that the three exome-wide significant signals from our analysis had equivalent aggregate frequencies and effect sizes in the GHS samples, power was >99% to detect the signals in *MC4R*, *PAM*, and *SLC30A8* at $p$=0.05. More conservatively, assuming effect sizes (log(OR)) were two-fold lower in the GHS study (e.g. due to winner's curse or differences in analytical protocols), and repeating calculations with aggregate frequencies equivalent to those actually observed in the GHS study, power at $p$=0.05 was 42%, 28%, and 31% for these three genes respectively.

GHS sequence data were processed and analyzed as previously described [32] and association results were produced for four (nested) variant masks:

1. M1: predicted loss-of-function variants, according to the VEP, with MAF<1% — similar to the LofTee mask but with an additional MAF<1% filter and without the LofTee filter on protein-truncating variants annotated by the VEP.

2. M2: nonsynonymous variants predicted deleterious by 5/5 prediction algorithms with MAF<1% — similar to the 5/5 mask but with an additional filter on MAF<1%.

3. M3: all nonsynonymous variants predicted deleterious by ≥1/5 bioinformatic algorithms with MAF<1% — similar to the 1/5 1% mask, although not identical as the 1% filter was used for all variants including those in the LofTee and 5/5 masks.

4. M4: all nonsynonymous variants with MAF<1% — similar to the 0/5 1% mask, although not identical as the 1% filter was used for all variants including those in the LofTee and 5/5 masks.

For each mask, association results were computed via logistic regression under an additive burden model (with phenotype regressed on the number of variants carried by each individual) with age, age$^2$, and sex as covariates. Although this analysis procedure was broadly consistent with the one we used for our exome sequence analysis, we were not able to synchronize our procedures for quality control, annotation, and consolidation of mask-level association statistics.

To produce a single GHS *p*-value for each gene, we applied the minimum *p*-value procedure across the four mask-level results. We estimated the correlation matrix using the same procedure as for our exome sequence analysis, using the combined GHS allele frequencies reported across the four (nested) masks.

### 1.6.2 Analysis of exomes from the CHARGE consortium

We collaborated with the CHARGE consortium to analyze the 50 genes with the strongest gene-level associations from our study (according to the lowest *p*-value observed across our four analyses) in 12,467 individuals (3,062 T2D cases and 9,405 controls) from their previously described study [33]. CHARGE DNA samples were processed at Baylor College of Medicine Human Genome Sequencing Center using the VCRome 2.1 design and sequenced in paired-end mode in a single lane on the Illumina HiSeq 2000 or the HiSeq 2500 platform with a mean 78-fold coverage. All samples were called together, with details on sequencing, variant calling, and variant quality control described previously [34]. Assuming (optimistically) that the three exome-wide significant signals from our analysis had equivalent aggregate frequencies and effect sizes in the GHS samples, power was 94%, 83%, and 65% to detect the signals in *MC4R*, *PAM*, and *SLC30A8* at $p$=0.05. More conservatively, assuming effect sizes (log(OR)) were two-fold lower in the CHARGE study, and repeating calculations with aggregate frequencies equivalent to those actually observed in the CHARGE study, power at $p$=0.05 was 7.9%, 29.5%, and 14.9% for these three genes respectively.

Variants in the CHARGE exomes were annotated and grouped into seven masks using the same procedure as for the original exome sequence analysis. For each mask, CHARGE burden and SKAT association tests were performed in the Analysis Commons [35] using a two-sided logistic mixed model [36] assuming an additive genetic model and adjusted for age, sex, study, race, and kinship.

To produce a single CHARGE *p*-value for each gene, we applied the minimum *p*-value procedure across the four mask-level results, as for the GHS analysis.

### 1.6.3 Meta-analysis with CHARGE and GHS

We conducted a meta-analysis among our original burden analysis and those of CHARGE and GHS. For each gene, we selected the mask that achieved the lowest *p*-value in our original analysis and conducted a two-sided sample-size weighted meta-analysis with the results from CHARGE and GHS within the same mask. As the masks analyzed for GHS did not precisely match those of our original analysis, we used the following approximate mapping between masks: LofTee to M1; 15/15, 10/10, 5/5, and 5/5+LofTee LC to M2; 1/5 1% to M3; and 0/5 1% to M4.

Each of the *MC4R*, *SLC30A8*, and *PAM* gene-level associations had weaker effects in the CHARGE and GHS studies as compared to the original analysis (**Supplementary Tables 5-6**). This observation — which could be due to a winner's curse effect, population differences among studies, and/or different procedures for variant calling, quality control, annotation, and association testing — illustrates that even the strongest T2D gene-level signals may show inconsistent replication across studies.

### 1.6.4 Investigation of the *UBE2NL* association

We investigated the novel association emerging from gene-level meta-analysis (*UBE2NL*, meta-analysis $p$=5.6$\times 10^{-7}$) in more detail. The *UBE2NL* burden signal was due to five PTVs in the original analysis (observed in 29 cases and 1 control; all of high [>45x] sequencing coverage; **Supplementary Table 8**) and was replicated at $p$=0.02 in CHARGE; *UBE2NL* results were not available in GHS. As *UBE2NL* lies on the X chromosome, we conducted a sex-stratified analysis of the original samples and observed independent associations in both men ($p$=5.7$\times 10^{-4}$) and women ($p$=1.6$\times 10^{-3}$). *UBE2NL* does not lie near any known GWAS associations [37], and has few available references [38–40], suggesting it may be a novel T2D-relevant gene, although further replication will be important to establish its association.

#### 1.6.5 Evaluation of directional consistency between exome sequence, CHARGE, and GHS analyses

We examined the concordance of direction of effect size estimates (i.e. OR>1 or OR<1) between our original exome sequence analysis and those from CHARGE and GHS. We used burden test statistics for this analysis, as SKAT tests do not produce direction of effects. Of the 50 genes advanced for replication, we considered the 46 that reached burden $p<0.05$ for at least one mask (i.e. ignoring those with evidence for association only under the SKAT model). We compared the direction of effect to that estimated by burden analysis of the same (or analogous) mask in the GHS or CHARGE analysis. For CHARGE, we compared direction of effect for the same mask. For GHS, we used the following approximate mapping between masks: LofTee to M1; 15/15, 10/10, 5/5, and 5/5+LofTee LC to M2; 1/5 1% to M3; and 0/5 1% to M4. We then conducted a one-sided exact binomial test to assess whether the fraction of results with consistent direction of effects was significantly greater than expected by chance.

## 1.7 Gene set analysis in sequence data

#### 1.7.1 Generation of candidate T2D-relevant genes sets

To assess whether gene-level association strength could be an informative metric to use when prioritizing candidate genes for further study or experimentation, we compared gene-level associations within a variety of gene sets (**Supplementary Table 9**) to gene-level association statistics within random sets of genes matched to the target set (as described below). We did so for 16 sets of genes:

1. Eleven genes harboring mutations that cause Maturity Onset Diabetes of the Young (MODY). We selected genes from a set previously described [1] after excluding two genes (*ABCC8* and *KCNJ11*) that can cause monogenic diabetes or congenital hyperinsulinism depending on whether the mutations they harbor are activating or inactivating.

2. Eight genes annotated as targets for antidiabetic medications. We downloaded medications annotated as "Drugs Used in Diabetes" or "Blood Glucose Lowering" from the DrugBank database version 5.0 [41]. After exclusion of medications with more than two annotated targets, we advanced for analysis only genes (a) annotated as a target of at least two compounds and (b) for which the therapeutic target modulation strategy was consistently annotated across all medications, where annotations of "inhibitor", "antagonist", and "inverse agonist" were interpreted as reducing activity, while annotations of "agonist", "activator", or "inducer" were interpreted as increasing activity. These restrictions initially excluded *ABCC8* (annotated as the target of both an inhibitor and an agonist) and *KCNJ11* (both medications in DrugBank targeting it listed as having more than two targets) from analysis in favor of *KCNJ1* and *KCNJ8*. However, based on multiple lines of evidence [42] indicating inhibition of *ABCC8*/*KCNJ11* to be the appropriate anti-diabetic strategy, we elected to replace *KCNJ1* and *KCNJ8* with *ABCC8* and *KCNJ11* in our analysis. The resulting gene set was thus *GLP1R*, *IGF1R*, *PPARG*, *INSR*, *SLC5A2*, *DPP4*, *KCNJ11*, and *ABCC8*.

3-14. Twelve sets of genes reported as relevant to T2D in mouse models. Within the Mouse Genome Informatics Database, we searched for genes matching various diabetes-relevant "phenotypes, alleles, and disease models" under the broader category of "mouse phenotypes and mouse models of human disease". We constructed a gene set for each phenotype defined in the database, many of which overlapped. For phenotypes associated with increased diabetes risk, we used: (3) "type 2 diabetes or type ii diabetes" (i.e. non-insulin dependent diabetes; 31 genes), (4) "diabetes mellitus" (72 genes), (5) "impaired glucose tolerance" (327 genes), (6) "increased circulating glucose" (365 genes), (7) "insulin resistance" (181 genes), and (8) "decreased insulin secretion" (133 genes). For phenotypes associated with decreased diabetes risk, we used: (9) "improved glucose tolerance"

(239 genes), (10) “decreased circulating glucose” (481 genes), (11) “increased insulin sensitivity” (178 genes), and (12) “increased insulin secretion” (51 genes). For phenotypes associated with diabetes risk but with unclear direction of effect, we used (13) “decreased circulating insulin” (321 genes) and (14) “increased circulating insulin” (215 genes).

15. Eleven genes suspected of harboring common coding causal variants within T2D GWAS loci. We analyzed the set of genes from a recent exome array analysis [28] which contained a coding variant GWAS signal for which the unweighted posterior probability of causality exceeded 25%. Although the final values reported by the study include an elevated prior for coding variants, we elected to use a 25% unweighted posterior threshold to enrich for the genes with the highest likelihood of mediating the observed GWAS signal. For analysis of this gene set, we recomputed gene-level association statistics within the set by conditioning on all GWAS tag SNPs (within the locus) reported in the exome array analysis [28]; we used *p*-values from these conditional gene-level associations in this (but no other) gene set analysis.

16. Twenty genes with T2D-associated transcript levels. We selected genes with significant associations in a pre-publication [43] tissue-wide T2D association analysis (i.e. testing for association between the genetic component of tissue-level gene expression and T2D), with associations considered significant if they survived Bonferroni correction for all tested genes and all tested tissues. Results were computed with the MetaXcan software package [44] using SNP regression coefficients taken from a large trans-ethnic T2D GWAS meta-analysis [45] and gene expression prediction models from the PredictDB website (http://predictdb.org).

#### 1.7.2 Gene set testing strategy

For each gene set, our goal was to compare the gene level *p*-values within the set to those of matched genes chosen at random from the genome. To control for gene variability in the number and frequency of variants within them, which could confound comparisons, we constructed comparison gene sets by matching genes on four properties: the (1) number of alleles across all masks; (2) total allele counts across all masks; (3) number of tests across all masks and transcript sets; and (4) effective number of tests across all masks and transcript sets (as computed for the minimum *p*-value test). We scaled each property to zero mean and unit variance. For each gene, we then used the 50 nearest neighbors (defined using Euclidean distance in the scaled property space) as matched comparison genes.

To conduct a gene set analysis, we then combined the genes in the gene set with all of the comparison genes matched to each gene in the set. Within the combined list of genes, we ranked genes using the *p*-values observed for the minimum *p*-value burden test. We then used a one-side Wilcoxon rank-sum test to assess whether genes in the gene set had significantly higher ranks than the comparison genes.

For gene set analysis, we used the minimum *p*-value test, rather than the weighted test, under the rationale that (a) we aimed to detect associations with as many genes as possible using information from as many variants as possible and (b) the weighted test might not detect genes that did not follow its model of a strong correlation between variant effect sizes and molecular annotation. We used the burden test rather than SKAT based on a desire to have more interpretable association statistics (e.g. effect size estimates). However, we did not quantitatively and systematically compare the power of each of our analyses in this setting.

#### 1.7.3 Sensitivity analysis of gene matching strategy

To assess the effects of our strategy for constructing comparison gene sets on our results, we performed three sensitivity analyses. First, for each gene set we constructed 100 comparison gene sets by random sampling (e.g. with no attempt to match genes according to any properties). Second, for each gene set we

constructed a comparison gene set via the original matching approach but with genes within 250kb of a T2D GWAS association excluded from consideration; we conducted this analysis to evaluate whether linkage disequilibrium between rare variants and common SNPs could affect our gene set results. Third, we combined these two sensitivity analyses by constructing comparison gene sets at random after excluding genes near a T2D GWAS association. For all gene sets, the *p*-values observed from these sensitivity analyses were within a factor of 2 of the original *p*-values, and nominal significance ($p<0.05$) was unaffected by the matching strategy used.

## 1.8 Application of gene-level associations

### 1.8.1 Use of gene-level associations to predict effector genes

In most situations, GWAS associations implicate common regulatory variants, which seldom localize to specific genes. To assess whether gene-level associations from exome sequencing — which are composed mostly of rare variants independent from any GWAS associations — could prioritize potential effector genes within known T2D GWAS loci, we first assessed whether predicted effector genes (based on common-variant associations) were also enriched for rare coding variant associations (i.e. exhibited a significant set-level association).

We constructed two sets of predicted effector genes (each described above): a curated list of 11 genes harboring likely causal common coding variants (gene set 15) and 20 genes significant in a transcript association analysis with T2D [43] (gene set 16). Genes with likely causal coding variants demonstrated a significant set-level association relative to comparison gene sets ($p$=8.8$\times10^{-3}$) and to genes within the same loci ($p$=0.028; **Figure 2e**), even when we conditioned gene-level associations on all significant common-variant signals. Most of this signal was due to the gene-level *SLC30A8* and *PAM* associations ($p$=0.082 for the other nine genes). By contrast, the transcript-association based gene set did not exhibit a significant association ($p$=0.72).

To then assess whether genes within T2D GWAS loci with nominally significant gene-level associations ($p<0.05$ for the minimum *p*-value burden test) were good candidates for effector genes, we curated a list of 94 T2D GWAS loci, and 595 genes that lay within 250 kb of any T2D GWAS index variant, from a 2016 T2D genetics review [46].

Among these 595 genes, 40 achieved a $p<0.05$ gene-level signal (**Supplementary Table 12**), greater than the 595$\times$0.05=29.75 expected by chance ($p$=0.038); only three (*SLC30A8*, *PAM*, and *HNF1A*) were from the list we curated of 11 genes with causal common coding variants [28]. We compared this set of 40 candidate effector genes to a set of 184 candidate effector genes defined based on proximity to an index SNP, as specified by the criteria used in the DAPPLE [47] implementation (at some loci DAPPLE annotated more than one candidate effector gene).

As accurately assessing which of these two gene sets is more enriched for true effector genes would require (at minimum) significant experimental work, we used the relative number of protein interactions within each gene set as one (imperfect) measure of their respective biological "coherence". To assess whether each set encodes proteins with more interactions than would be expected by chance, we ran DAPPLE through the public GenePattern portal (https://software.broadinstitute.org/cancer/software/genepattern) with default values for all parameters. The 40 genes with minimum $p<0.05$ were significantly more enriched for protein interactions ($p$=0.03; observed mean=11.4, expected mean=4.5) than were the 184 genes implicated based on proximity to the index SNP ($p$=0.64; observed mean=21.1, expected mean=21.9).

These results provided modest evidence that the set of effector genes predicted by rare coding variants has greater biological coherence than the set of effector genes predicted by proximity to an index SNP. We note, however, that our analysis does not implicate any specific genes and that DAPPLE is only one means to assess biological coherence of a gene set (through direct and indirect protein interactions). Evaluation of the biological candidacy of these genes will ultimately require in-depth functional studies [48]. Rare coding variants could therefore, in principle, complement common-variant fine mapping [49, 50] and experimental

data [48, 51] to help interpret T2D GWAS associations, but our results indicate that much larger sample sizes and/or orthogonal experimental data will be required to clearly implicate specific effector genes.

#### 1.8.2 Use of gene-level associations to predict direction of effect

In therapeutic development, it is often valuable to know the direction of effect linking gene modulation to disease risk — that is, whether inactivation or activation of a protein increases disease risk. We thus assessed whether gene-level association analysis of predicted deleterious variants could be used to predict this direction of effect. For this analysis, we used ORs estimated from a modified weighted burden test procedure, which only included alleles from the four masks with the predicted most deleterious variants: LofTee, 16/16, 11/11, and 5/5 (**Extended Data 6**). Weights for variants were identical to those used in the exome-wide weighted burden test. We chose these four masks for analysis to balance a desire for greater aggregate allele count per gene (i.e. missense variants in addition to protein-truncating variants) with a need to strongly enrich for deleterious variants (>73% estimated to be deleterious in masks analyzed vs. <50% in the other masks [**Extended Data 6**]). In addition, we used the weighted test because it was explicitly designed to estimate an effect of gene haploinsufficiency based on both protein-truncating and missense variants.

To compare these direction of effect estimates to those expected for T2D drug targets, we assumed agonist targets to have true OR>1 and inhibitors to have true OR<1. For a comparison to expectations for mouse gene knockouts, we first excluded 473 genes annotated, based on membership in multiple gene sets, to have both expected OR>1 and expected OR<1 (these genes were excluded only from the direction of effect comparisons; they were maintained in all other gene set analyses). This left 389 genes with an expected OR>1, associated exclusively with mouse traits indicative of increased risk (overlapping sets of 11 "type 2 diabetes or type ii diabetes", 46 "diabetes mellitus", 204 "impaired glucose tolerance", 245 "increased circulating glucose", 104 "insulin resistance", and 63 "decreased insulin secretion"), and 467 genes with an expected OR<1, associated exclusively with traits indicative of decreased risk (overlapping sets of 164 "improved glucose tolerance" genes, 358 "decreased circulating glucose" genes, 95 "increased insulin sensitivity" genes, and 18 "increased insulin secretion" genes). Gene sets for "decreased circulating insulin" and "increased circulating insulin" were excluded from this direction of effect comparison due to the unclear relationship between these phenotypes and T2D risk.

### 1.9 Imputed GWAS analysis

#### 1.9.1 Aggregation and generation of SNP array data

Because the most significant single-variant associations that emerged from our exome sequence analysis were with common variants, we asked whether an array-based genome-wide association study in the same samples could have provided a less expensive method to detect these same associations. To address this question, we aggregated all available SNP array data for the exome-sequenced samples (**Supplementary Table 13**). Data for the GoT2D [1], SIGMA [2], and T2D-GENES consortia have been previously analyzed (unpublished T2D-GENES data were collected from a range of SNP arrays including Affymetrix 5.0 and 6.0, Illumina HumanHap 610K and 1M, and the Illumina CardioMetabochip). The newly sequenced samples from the T2D-GENES and SIGMA consortia were genotyped on a custom "Genomes For Life" (G4L) Illumina Infinium array, including 243,662 variants chosen to uniquely identify each individual in a study and to provide a backbone for imputation of common variation. The G4L array was processed by the Arrays lab of Broad Genomics and called using the Illumina GenCall (Autocall) algorithm.

### 1.9.2 Analysis of SNP array data

After genotyping, the 34,529 samples (18,233 cases and 17,679 controls; **Supplementary Table 13**) both in the exome sequence analysis and with a SNP array call-rate $>$95% were advanced for imputation. To omit variants that might degrade imputation quality, prior to imputation we excluded variants with low genotype call rate ($<$95%), strong deviation from Hardy-Weinberg equilibrium ($p<10^{-6}$), differential genotype call rate between cases and controls ($p<10^{-5}$), or low frequency (MAF$<$1%). We then imputed autosomal variants (SNVs, short indels, and large deletions) via the Michigan Imputation Server [52] for each of two reference panels: the all ancestries 1000 Genomes Phase 3 (1000G) reference panel of 2,504 individuals [53] and the Haplotype Reference Consortium (HRC) Panel of 32,470 individuals [54]. We used the 1000G-based imputation for all association analyses and the HRC-based imputation to assess the number of exome sequence variants imputable from the largest available European reference panel. We note that the HRC panel includes only SNPs (i.e. no indels) and only variants observed at least five times in the HRC are included in the imputation panel.

After imputation, we performed sample and variant quality control, as well as two-sided association tests, analogous to the exome sequence single-variant analysis. By contrast with the exome sequence analysis, we found that the EMMAX test produced more suspicious looking associations than did the Firth test and thus used only the Firth test (i.e. for both *p*-values and ORs) in the imputed GWAS analysis.

To determine which variants in the exomes dataset were imputable from the 1000G or HRC panel, we calculated which of the exome variants passed imputed GWAS quality control in any sample subgroup, with a further restriction of achieving $r^2 > 0.4$ in that subgroup. Only variants in the exomes dataset polymorphic in samples with SNP array data were included in this analysis. For calculations involving the HRC-imputed GWAS (given that the HRC panel is European-specific), we only considered variants variable in four European cohorts (METSIM, Ashkenazi, GoDARTS, and FHS) in the analysis.

### 1.9.3 Gene set analysis using SNP array data

In addition to single-variant analysis, we conducted gene set analysis with the imputed GWAS data. We first used the method implemented in MAGENTA [55] to assign gene scores from the imputed GWAS single-variant association results; MAGENTA gene scores are based on proximity to a GWAS lead SNP after correction for potential confounding factors. Following the same protocol as for gene set analysis from the exome sequence results, we then conducted a one-sided Wilcoxon rank-sum test to compare the gene scores to those of matched comparison genes.

As the imputed GWAS gene set analysis produced fewer significant gene set associations than did the exome sequence gene set analysis, we investigated whether a larger array-based association study would produce more significant gene set associations (i.e. whether the lack of gene set associations in the imputed GWAS was due to a fundamental lack of associated common variants near the genes in the gene set or simply due to an insufficient sample size). For this analysis, we downloaded single-variant association statistics from the largest available multi-ethnic array-based GWAS for T2D [45], converted them to MAGENTA gene scores, and then for each gene set conducted a one-sided Wilcoxon rank-sum test as described above.

## 1.10 LVE analysis

### 1.10.1 LVE calculations

To calculate liability variance explained (LVE), we used a previously presented formula [56] to calculate the LVE of a variant with three genotypes ($AA$, $Aa$, and $aa$) and corresponding relative risks (1, $RR_1$, and $RR_2$). For these calculations we assumed HWE, implying the frequencies of the three genotypes to be $P_{aa} = P_a^2$, $P_{Aa} = 2P_a(1 - P_a)$, and $P_{AA} = (1 - P_a)^2$, where $P_a$ is the minor allele frequency. Under this

assumption, LVE can be expressed as

$$LVE = P_a^2\left(\mu_{aa}-\mu\right)^2 + 2P_a\left(1-P_a\right)\left(\mu_{Aa}-\mu\right)^2 + \left(1-P_a\right)^2\left(\mu_{AA}-\mu\right)^2$$

where $\mu = 2P_a\left(1-P_a\right)\mu_{Aa} + \left(1-P_a\right)^2\mu_{AA}$, and

$$\mu_{aa} = 0; \mu_{Aa} = T - \Phi^{-1}\left(1-f_{Aa}\right); \mu_{AA} = T - \Phi^{-1}\left(1-f_{AA}\right)$$

Here $\Phi^{-1}$ is the normal quantile distribution, $T = \Phi^{-1}\left(1-f_{aa}\right)$, and $f_{aa}$, $f_{Aa}$, and $f_{AA}$ are defined as

$$f_{aa} = \frac{K}{P_a^2 + 2P_a\left(1-P_a\right)RR_1 + \left(1-P_a\right)^2 RR_2}; f_{Aa} = RR_1 f_{aa}; f_{AA} = RR_2 f_{aa}$$

where $K$ is the disease prevalence.

The inputs to these formulae are estimates of allele frequency (for either individual variants or sets of variants, depending on whether variant-level or gene-level variance is to be calculated), relative risk, and disease prevalence. For individual variants, we used the point estimate of the MAF from our analysis to estimate allele frequency, while for genes we used the point estimate of combined allele frequency (across all alleles) in place of MAF. We estimated relative risks from analysis ORs and MAFs ($\hat{P}_a$) under an assumed prevalence of $K = 0.08$ and an additive genetic model, by iteratively solving two equations [56]:

$$f_{aa} = \frac{K}{\hat{P}_a^{\,2} + 2\hat{P}_a\left(1-\hat{P}_a\right)RR_1 + \left(1-\hat{P}_a\right)^2 RR_2}$$

$$RR_i = \frac{OR_i}{1 + f_{aa}\left(OR_i - 1\right)}$$

where $i = 1, 2$ correspond to the heterozygous and major-allele homozygous genotypes. We used a multiplicative model for odds-ratios; i.e. $OR_2 = OR_1^2$.

We performed LVE calculations as an integral over the distribution of potential relative risks, assuming that the logarithm of odds ratio $OR_i$ followed normal distributions with means and variance equal to those estimated from our analysis. When presenting the strongest LVE values for the imputed GWAS analysis, we only considered variants genotyped in at least 10,000 individuals to avoid potential artifacts resulting from a spurious association in a small sample subgroup. For gene-level LVE calculations, we used the variant mask with lowest *p*-value to calculate LVE.

Under this model, the three exome-wide significant gene-level signals explain an estimated 0.11% (*MC4R*), 0.092% (*PAM*), and 0.072% (*SLC30A8*) of T2D genetic variance. These estimates are only 10–20% of the variances explained by the three strongest independent common-variant associations in the imputed GWAS of the same samples (*TCF7L2*, 0.89%; *KCNQ1*, 0.81%; and *CDC123*, 0.35%) and if anything overstate the heritability explained by rare variants in the gene-level signals, since the *MC4R* and *PAM* estimates are attributable mostly to the low-frequency p.Ile269Asn (70.9% of the gene-level total) and p.Asp563Gly (83.3%) alleles. We obtained similar results in a broader comparison between all (19) previously identified index SNPs achieving $p<5\times10^{-8}$ in the imputed GWAS and the top 19 gene-level signals from the exome sequence analysis (**Figure 3b**).

These results argue against a large contribution to T2D heritability from rare variants in the strongest observed gene-level signals, with one caveat: as gene-level tests may include benign alleles that can dilute evidence for association, their aggregate effects might underestimate the true contribution of rare functional variants to T2D heritability [21]. To therefore calculate an upper bound on the LVE by only disease-associated alleles, we performed a series of LVE calculations for progressively larger sets of alleles, at each step including alleles by order of decreasing single-variant significance. We performed two calculations for each gene, one for risk alleles and one for protective alleles, taking the maximum of the

two as the final upper bound estimated for LVE by the gene. We did not calculate an LVE bound under a model whereby alleles within the gene can both increase and decrease risk of disease. These calculations showed that, among all subsets of variation in the three most significant gene-level signals, none explained more than 20% of the heritability of the single-variant *TCF7L2* association (maximum of 0.18% for *MC4R*, 0.15% for *PAM*, 0.17% for *SLC30A8*).

#### 1.10.2 Prediction of LVE explained by the top 100 and top 1000 gene-level associations

To forecast the LVE that will be explained once 100 (or 1000) significant T2D gene-level associations are detected, we applied a previously suggested model [57] relating the LVE of a gene to its rank in the overall gene-level *p*-value distribution. Specifically the model is $LVE_n = e^{an+b}$, where $LVE_n$ is the LVE of the gene with n^th^ lowest gene-level *p*-value. We fit this model using linear regression to the top 50 genes in our analysis (**Supplementary Figure 20**), yielding estimates of $a = -0.044$ and $b = -7.07$. We then calculated the LVE of the top 100 (or 1000) genes by summing the actual LVE of the top three signals (which achieved exome-wide significance in our analysis) with the LVE predicted by the model for genes ranked $4 - 100$ (or $4 - 1000$).

#### 1.10.3 Estimated power to detect gene-level associations with T2D drug targets

To estimate the power of future studies to detect gene-level associations in genes with effect sizes similar to those for established T2D drug targets, we used aggregate allele frequencies and ORs estimated from our gene-level analysis and an assumed prevalence of $K = 0.08$ to calculate a proxy for true population frequencies and relative risks. For each gene, we used ORs and frequencies from the variant mask yielding the strongest gene-level association. Because on average these drug targets had roughly 5 effective tests per mask, we used an exome-wide significance threshold of $\alpha = 1.25 \times 10^{-7}$ for power calculations. We calculated power as previously described [16].

The ranges given in the main text (75,000-185,000 disease cases) represent the numbers from the power calculations for *INSR* (the drug target with highest observed effect size) and *IGF1R* (the drug target with lowest observed effect size other than *KCNJ11* and *ABCC8*). We excluded *KCNJ11* and *ABCC8* from this reported range, given that a mixture of risk-increasing and risk-decreasing variants in these genes likely diluted their burden signals. We did not account for uncertainty in estimated OR or aggregate variant frequency in these calculations, as no genes had 95% confidence intervals that that did not overlap OR=1.

## 1.11 Interpretation of suggestive associations

### 1.11.1 Estimated fraction of true associations

We sought to quantify the proportion of true associations (*PPA*) for nonsynonymous variants observed in our dataset as a function of association strength measured by single-variant *p*-value. We define a true association as a variant which, when studied in larger sample sizes, will eventually achieve statistical significance owing to a true OR≠1. We distinguish true association from causal association: causally associated variants are the subset of truly associated variants in which the variant itself is causal for the increase in disease risk, as opposed to being truly associated due to LD with a different causally associated variant.

To estimate *PPA*, we used as training data a previous exome array study from the GoT2D consortium spanning 13 European cohorts [1]. As two of the 13 cohorts included in the previous study contributed samples to the current exome sequence analysis, we re-calculated a fixed-effects inverse-variance weighted meta-analysis for every variant in the exome array study after excluding all samples from these two overlapping cohorts. This yielded a collection of exome array association statistics for 206,373 variants, with a maximum sample size of 50,567 (maximum effective sample size 41,967).

We then compared variant direction of effect estimated from our exome sequence analysis of 45,231 individuals to those estimated from the independent exome array analysis of 50,567 individuals. To produce an uncorrelated set of association tests for this analysis, we pruned all collections of variants using the LD-clump procedure (parameters `-clump-p1 0.1 -clump-p2 0.1 -clump-r2 0.01`) of the PLINK software package [8], which required variants to have pairwise $r^2 < 0.01$. We performed this procedure for (a) nonsynonymous variants within 94 previously established T2D GWAS loci and (b) nonsynonymous variants exome-wide. Within established T2D GWAS loci, 1,059 nonsynonymous variants achieved $p$<0.05 in the exome sequence analysis, 191 of which were also analyzed in the independent exome array analysis. Of these 191 variants, the directions of effect were concordant (both OR>1 or both OR<1) between the exome sequence and exome array analysis for 61.3% of variants. This fraction decreased (as expected) for higher $p$-value thresholds (e.g. 49.4% at $p$>0.5) and when only variants outside of T2D GWAS loci were analyzed (51.9% at $p$<0.05).

To estimate the fraction of true associations among the set of variants achieving significance below a threshold $p$ (e.g. $p$<0.05), we modeled the set of variants as a mixture of proportions $x_p$ of truly associated variants (OR≠1) and $(1 - x_p)$ of non-associated variants (OR=1). We assumed non-associated variants have a 50% chance of a concordant direction of effect between the two analyses, and truly associated variants have a greater chance according to their estimated effect size. Specifically, assuming that the observed effect size for a variant follows a normal distribution with mean equal to the true effect and variance that scales inversely with sample size, we estimated the probability $p_i$ of producing a concordant effect for variant $v_i$ as

$$p_i = \Pr\left(\mathcal{N}\left(|\hat{\beta}|, \hat{\sigma}\sqrt{\frac{N_{ex}}{N_{ea}}}\right) > 0\right)$$

where $|\hat{\beta}|$ is the absolute value of the estimated (from the exome sequence analysis) logarithm of the OR, $\hat{\sigma}$ is the estimated standard error of the logarithm of the OR, $N_{ex}$ is the effective sample size of the exome sequence analysis, and $N_{ea}$ is the effective sample size of the exome array analysis.

The expected fraction of variants exhibiting concordant direction of effect is then

$$f_p = \frac{\sum_{i=1}^{V_p} p_i x_p}{V_p} + 0.5\,(1 - x_p)$$

where $V_p$ is the number of variants in the set. Based on the observed fraction $\hat{f}_p$ of variants with concordant directions of effect, we thus estimated $x_p$ by

$$\hat{x_p} = \frac{\hat{f}_p V_p - 0.5 V_p}{\sum_{i=1}^{V_p} p_i - 0.5 V_p} \tag{1}$$

To calculate a 95% confidence interval (CI) for $x_p$, we first estimated a 95% CI for $f_p$ using the Jeffreys interval method [58], as implemented in the R software package (https://www.r-project.org), and we then used equation (1) to convert its lower and upper bounds to lower and upper bounds on the corresponding confidence interval for $x_p$.

Because the $p$-value to *PPA* mapping depends on the set of variants under consideration, we computed separate mappings for arbitrary nonsynonymous variants (using all nonsynonymous variants exome-wide) and one for nonsynonymous variants within GWAS loci (using only nonsynonymous variants within the 94 T2D GWAS loci). We also note that the mapping produced from our analysis applies only to the results from the current study. Because other studies have different sample sizes and may apply different statistical tests, the mapping would need to be re-computed to interpret the associations of other studies via the same method.

### 1.11.2 Probability of causal association

The estimated values for $x_p$ can be interpreted as estimates of the posterior probability that a variant with $p<0.05$ in our analysis is truly associated with T2D rather than due to chance (i.e. the *PPA* for a variant with $p<0.05$). As our ultimate goal was to quantify the probability of *causal* association, rather than just true association, we modeled the probability of variant association as a function of (a) the probability of causal association ($PPA_c$), influenced in turn by the likelihood that the variant impairs the function of a gene relevant to T2D; and (b) the prior probability of indirect association ($PPA_i$), influenced in turn by the likelihood that the variant is in LD with a nearby but different variant that is causally associated with T2D. Under the (not always accurate) assumption that causal and indirect associations are disjoint events, this model expresses *PPA* as

$$PPA = PPA_c + PPA_i$$

Precisely determining which coding variant associations are in fact causal requires fine mapping of all nearby variants in large sample sizes [49], which is currently infeasible for the mostly rare variants observed in our study. Since we could not accurately calculate specific values of $PPA_c$ and $PPA_i$ for each variant, we instead used estimates of the average the proportion of associations that are causal ($\alpha$) We note that $\alpha$ is the probability of causal association conditional on true association, rather than the absolute probability of causal association. We considered two means to estimate $\alpha$.

First, recent analyses have attempted to assess the contribution of nonsynonymous variants to T2D or similar traits, either by directly estimating the proportion of associations that are due to nonsynonymous variants [59] or by measuring the proportion of heritability explained by nonsynonymous variants [60]. These analyses suggest that roughly 10% of T2D associations are likely to be due to nonsynonymous variants. As these calculations apply to all associations in the genome, rather than those in which at least one nonsynonymous variant achieves significance, they likely underestimate the proportion of nonsynonymous associations that are causal.

Second, a recent exome array study identified 40 exome-wide significant nonsynonymous variant associations and then calculated the probability of causal association for each (via credible set analysis) [50]. The reported average probability of causal association across these variants of 49.2% provides a direct estimate of $\alpha$. This estimate is likely less biased than that based on genome-wide analyses of all T2D associations, but it is based on a small number of associations and thus has a high variance. Additionally, this estimate is the average across all of the 40 reported variants and does not account for dependence of the posterior on MAF: as rarer variants in general have a higher posterior probability of causal association than common variants (**Supplementary Figure 21**), and most variants in an exome sequencing study are rarer than those in a SNP array study, 49.2% likely underestimates $\alpha$ for variants in our study.

Because a rigorous estimation of $\alpha$ is beyond the scope of the current study, we chose to conduct analyses with multiple values for $\alpha$: 10%, 30%, and 50%. We used 30% as our default value for analyses reported in the main manuscript. For any value of $x_p$, representing the fraction of true associations (*PPA*) at a given *p*-value threshold, we calculated a value for $x_p^c$, representing the fraction of causal associations ($PPA_c$) at a given *p*-value threshold, as $x_p^c = \alpha x_p$. Under this model, using a different value for $\alpha$ (e.g. 50% or 10%) would scale $PPA_c$ estimates linearly (e.g. 5/3 or 1/3 as large).

### 1.11.3 Incorporation of prior likelihood into posterior probability estimations

Following previous work [61], the posterior probability of causal association $PPA_c$ can be expressed as a combination of the prior odds of causal association for the variant, $\pi$ (i.e. the belief, prior to observing any genetic association data, that the variant is causally associated with T2D), and the Bayes factor for causal association of the variant calculated from genetic association data, $BF_c$:

$$PO_c = BF_c \frac{\pi}{1-\pi} \tag{2}$$

where $PO_c$ is the posterior odds of causal association expressed as

$$PO_c = \frac{PPA_c}{1 - PPA_c} \tag{3}$$

We use a "c" subscript in $PO_c$ and $BF_c$ to emphasize that they are posterior odds (and Bayes factors) for causal association, rather than just true association.

Given an estimate $x_p^c$ of the posterior probability of causal association (i.e. $PPA_c$) for a class of variants (e.g. those satisfying $p<0.05$), as well as a prior probability of causal association $\pi$ for the same class of variants, we can calculate an estimate of the average Bayes factor for variants in the class as:

$$BF_p^c = \frac{x_p^c}{1 - x_p^c}\frac{1-\pi}{\pi} \tag{4}$$

Here, $BF_p^c$ denotes the average Bayes factor for causal association (i.e. the ratio of the likelihood of the observed data under the model of causal association to the likelihood of the observed data under the model of no association) for variants with $p$-value below a given $p$-value $p$. We note that this equation indirectly infers an average Bayes factor from a direct estimate of an average posterior ($x_p^c$) and a specified prior $\pi$, which is different from how Bayes factors are usually calculated.

### 1.11.4 Inference of Bayes factors from GWAS variant posteriors

To calibrate the relationship between $p$-value and $BF_p^c$ (as expressed via equations (1)-(4)), we required a set of variants for which both the posterior and prior likelihood of association could be reasonably estimated. We elected to use nonsynonymous variants within GWAS loci for model calibration: there were over $>$1000 such variants achieving $p<0.05$ in our analysis (enabling relatively accurate posterior calculations), and it was further possible to develop an explicit prior model at these loci. We note that our methodology implicitly assumes that the relationship between a variant's $\pi$ and $PO_c$ is, given its observed $p$-value, conditionally independent of all other variant properties (i.e. dependence on properties such as sample size is entirely captured by the observed $p$-value).

Our GWAS locus prior model was inspired by the often implicit expectation that the associations within a GWAS locus usually act through a single effector gene (although multiple effector genes may be more common than previously thought [62]). We assumed a simple (previously suggested [21]) model in which variants cause only full protein-inactivation or have no effect on protein function, and in which only variants causing full protein-inactivation are associated with disease risk. This model does not account for other classes of coding variants (e.g. hypermorphs) or the possibility that some effector genes may be relevant to T2D only through regulatory — but not coding — changes.

Specifically, our model assumed (a) on average 1.1 genes within 250kb of each GWAS signal harbor coding variants associated with T2D; (b) missense variants are a mixture of fully benign and fully protein-inactivating variants [21]; (c) only inactivating missense variants are associated with disease risk; and (d) one-third of missense variants are inactivating. This estimate of one-third was calculated as mean weight of variants in our dataset (as computed for the "weighted" gene-level test), as these weights were designed to directly estimate the probability that variants in a mask cause full loss of function; this calculation produced a prior estimate of 34.2% for nonsynonymous variants in our dataset, not far from a previously reported value of 25% [21]. Based on the 595 genes within the 94 T2D GWAS loci in our analysis, this yielded a prior estimate of causal association for coding variants within GWAS loci of $0.057 = \left(\frac{1.1\times 94}{595}\right) \times 0.33$.

Through this prior of 0.057, and equations (1)-(4) above, we produced a lookup table mapping variant $p$-values to Bayes factors of causal association ($BF_c$). For any subsequent variant $v$ with observed $p$-value $p(v)$ and a user-specified prior on the relevance of its gene to T2D, we then calculated its posterior likelihood of association by mapping $p(v)$ to $BF_c(v)$ and then employing equations (2) and (3) to calculate an estimated posterior probability of causal association ($PPA_c(v)$). Although not presented here, lower

and upper confidence intervals on $PPA_c$ can also be estimated by repeating this procedure using the lower and upper confidence intervals for $x_p^c$ in equation (4).

#### 1.11.5 Sensitivity of $PPA_c$ to modeling parameters and other limitations of the calculations

The above procedure relies on two parameters, the specific values of which will affect final $PPA_c$ estimates for variants in our dataset. First, conversion of *PPA* (estimated from concordance of variant effect sizes in equation (1)) to $PPA_c$ requires a parameter for the proportion of true nonsynonymous associations that are causal. As described above and in the text, we used a value — of 30% — in between a published estimate of the proportion of nonsynonymous associations within GWAS loci that are causal (49.2%) and a published estimate of the proportion of causal associations that are nonsynonymous (roughtly 10%). Using a different value (e.g. 50% or 10%) would scale the final $PPA_c$ estimates linearly (e.g. 5/3 or 1/3 as high).

Second, for calculations of $PPA_c$ in light of a user-specified prior, calibration of our model requires a parameter for the proportion of nonsynonymous variants in GWAS loci that causally influence T2D risk (based only on data obtained prior to any exome sequence analysis). We note that this parameter does not affect our reported $PPA_c$ estimates genome-wide or within GWAS loci, as we directly estimate $PPA_c$ for these genes from our data and therefore do not require a user-specified prior. In developing the prior model at GWAS loci, we decompose this parameter into two — a parameter for the proportion of genes within T2D GWAS loci that are relevant to disease and a parameter for the proportion of missense variants within a gene that result in loss of function. However, only the product of the two parameters is used in the model.

To gain intuition as to the sensitivity of our final $PPA_c$ estimates to these parameters, we repeated our calculations with different values for them. To assess sensitivity to the assumption of 1.1 effector genes per T2D GWAS locus, we repeated all calculations with the alternate choices of 0.1, 0.25, 0.5, and 2 genes per GWAS locus (**Extended Data 10ab**). A value of 2 could represent an extreme where multiple effector genes are common at GWAS loci, while a value of 0.1 could represent an extreme where either many GWAS associations act in *trans* or where many effector genes do not affect T2D risk through coding changes.

To assess sensitivity of the assumption of 33% of coding variants leading to protein-inactivation, we also repeated all calculations with values of 40% and 25% for this parameter (**Extended Data 10cd**).

Our $PPA_c$ calculations have other limitations beyond sensitivity to modeling parameters. They apply only to single-variant associations and not (yet) gene-level associations; extending them to apply to gene-level associations would avoid the possibility of conflicting results among variants within a gene but require larger-scale gene-level replication data than we had available in the current analysis. Additional work will also be needed to generate data and develop methods to estimate objective rather than subjective gene priors (researchers can often over-estimate evidence of disease-relevance for genes in which they have invested significant effort), reduce dependence of our conclusions on modeling assumptions (**Extended Data 10**), and explore the extent to which the large number of causal variant associations we predict from our data localize to specific gene or variant functional annotations [60].

#### 1.11.6 Estimation of prior for genes in the Mouse NIDD gene set

As a preliminary estimate of the prior likelihood of T2D-relevance for genes in the Mouse NIDD gene set, we estimated the proportion of non-null associations across all genes in the set. To use true "prior" data (rather than associations from the current study), we calculated gene-level *p*-values for each gene in the set using the MAGENTA [55] algorithm applied to a recent transethnic T2D GWAS [45]. We then used a previously developed approach [63, 64] that models the distribution of observed *p*-values as a mixture of

uniform (representing the null distribution) and beta (representing the non-null distribution) distributions as

$$f\left(p|a,\lambda\right)=\lambda+\left(1-\lambda\right)ap^{a-1},$$

estimating $\hat{\lambda}$ and $\hat{a}$ via maximum likelihood and calculating the prior as

$$1-\left(\hat{\lambda}+\left(1-\hat{\lambda}\right)\hat{a}\right).$$

This procedure produced estimated values $\hat{\lambda}=0.74$, $\hat{a}=0.12$, and a prior value of 23.2%.

## 2 Supplementary Tables

**Supplementary Table 1**: **Samples included in analysis.** Shown are characteristics of the samples analyzed in the study. Subgroup: the label used for the sample collection throughout the manuscript and figures. Ancestry: the ancestry of the samples. Consortium: the consortium in which samples where first collected and/or analyzed. Study: the study (i.e. cohort) from which samples were drawn. Citation(s): references describing the samples in more detail. T2D Case (Control) Ascertainment: criteria used to define and/or select T2D cases (controls). T1D and MODY exclusion criteria: criteria used (if applicable) to exclude type 1 diabetes or MODY cases from the study. Whole exome sequencing technology: the sequence capture technology used for exome sequencing of the samples. dbGAP (EGA): accession number for download of subgroup data from dbGAP (EGA).

[See separate Excel file]

**Supplementary Table 2**: **Samples excluded from analysis by quality control.** To identify samples with evidence of poor sequencing quality, we computed a range of metrics. We then excluded samples who appeared as visual outliers on plots (stratified by sample ancestry and sequencing technology) of these metrics; example plots are shown in **Supplementary Figure 1**. Shown are the number of samples excluded according to each metric, as well as the total number of samples excluded across all metrics.

| **Metric** | **Samples Removed** |
|---|---:|
| Average (allele balance - 50%) | 6 |
| Average allele balance | 14 |
| Call rate | 260 |
| GWAS concordance (GoT2D samples) | 202 |
| GWAS concordance (SIGMA samples) | 17 |
| GWAS concordance (T2D-GENES samples) | 10 |
| GWAS concordance (newly sequenced samples) | 26 |
| Heterozygosity | 27 |
| Heterozygosity at common variants | 8 |
| Heterozygosity at low frequency variants | 41 |
| Number of heterozygous genotypes | 15 |
| Number of homozygous non-reference genotypes | 12 |
| Number of minor alleles | 227 |
| Number of non-reference SNP alleles | 2 |
| Number of singleton variants | 45 |
| Number of variants | 241 |
| Transition:Transversion | 30 |
| **Total** | **481** |

**Supplementary Table 3**: **Variants identified from exome sequencing.** Shown are the number of variants identified by exome sequencing and then advanced for association analysis after quality control. Variant counts are stratified by sequence ontology [65] annotation, produced by the Variant Effect Predictor [11], and further by minor allele frequency (MAF), calculated as the maximum across all ancestries. Rows in the table are shown in decreasing order or predicted deleteriousness.

| **Annotation** | **MAF$>$0.05** | **0.005$<$MAF$<$0.05** | **MAF$<$0.005** | **Total** |
|---|---|---|---|---|
| splice_acceptor_variant | 82 | 252 | 13431 | 13765 |
| splice_donor_variant | 113 | 297 | 16898 | 17308 |
| stop_gained | 429 | 1061 | 57132 | 58624 |
| frameshift_variant | 756 | 1501 | 41963 | 44221 |
| stop_lost | 29 | 48 | 1763 | 1840 |
| start_lost | 34 | 89 | 3809 | 3932 |
| inframe_insertion | 218 | 260 | 3326 | 3804 |
| inframe_deletion | 492 | 1175 | 14830 | 16497 |
| missense_variant | 25660 | 60344 | 2011159 | 2097179 |
| protein_altering_variant | 10 | 11 | 145 | 166 |
| splice_region_variant | 5894 | 10074 | 221362 | 237335 |
| incomplete_terminal_codon_variant | 2 | 1 | 27 | 30 |
| stop_retained_variant | 18 | 42 | 988 | 1048 |
| synonymous_variant | 27748 | 46978 | 994052 | 1068784 |
| coding_sequence_variant | 21 | 21 | 188 | 230 |
| mature_miRNA_variant | 4 | 11 | 421 | 436 |
| 5_prime_UTR_variant | 2779 | 5020 | 88785 | 96586 |
| 3_prime_UTR_variant | 4748 | 8278 | 148107 | 161135 |
| non_coding_transcript_exon_variant | 3438 | 5461 | 95771 | 104671 |
| intron_variant | 62536 | 101810 | 1798630 | 1963024 |
| upstream_gene_variant | 4790 | 8956 | 175482 | 189234 |
| downstream_gene_variant | 5730 | 10705 | 220197 | 236636 |
| TF_binding_site_variant | 2 | 2 | 5 | 9 |
| regulatory_region_variant | 31 | 58 | 487 | 576 |
| intergenic_variant | 137 | 138 | 1700 | 1975 |
| Other | 894 | 589 | 5270 | 6753 |
| **Total** | **146595** | **263182** | **5915928** | **6325798** |

**Supplementary Table 4**: **Associations by allele mask for most significant gene-level associations.** For the 11 strongest gene-level associations, as determined by the weighted burden, weighted SKAT, minimum *p*-value burden, and minimum *p*-value SKAT analyses (all two-sided, N=43,071 unrelated individuals), shown are statistics for each mask and each of the burden test and SKAT. We performed analyses without the use of allele weights and included all alleles in each mask (so that the sets of alleles are nested within masks). Gene: a unique identifier for the gene within our exome sequence analysis. Trans: the transcript set used for the analysis (All: all transcripts. Best: "best-guess" transcript). Mask: the allele mask used for analysis. Var: the number of alleles included in the mask. CAF: the combined allele frequency of all alleles in the mask. OR: the aggregate odds-ratio for alleles in the mask, computed by the burden test. Burden: the (two-sided) *p*-value from burden analysis of alleles in the mask. SKAT: the (two-sided) *p*-value from SKAT analysis of alleles in the mask.

[See separate Excel file]

**Supplementary Table 5**: **Evaluation of association signals in CHARGE.** Shown are results from gene-level analysis within the CHARGE dataset (N=12,467 individuals), which included the 50 genes with lowest (two-sided) *p*-value from the original exome sequence analysis. Results are shown for each mask. Var: the number of alleles in the mask; CAC: the combined count of all alleles in the mask; Score: the score statistic from a burden analysis of the mask (positive values denote increased risk, negative values denote decreased risk); Burden: the (two-sided) *p*-value from a burden analysis of the mask; SKAT: the (two-sided) *p*-value from a SKAT analysis of the mask. Best Burden (SKAT) indicate *p*-values from a (two-sided) minimum *p*-value test across all masks for the burden (SKAT) analyses.

[See separate Excel file]

**Supplementary Table 6**: **Evaluation of association signals in GHS.** Shown are results from the precomputed gene-level analysis of the GHS dataset (N=49,199 individuals). As custom analytical results were unavailable, the precise masks and testing methodologies are only broadly similar to those used in our exome-wide gene-level analysis. Genes are sorted in order of increasing *p*-value in the GHS dataset. The top 50 genes from the original exome sequence analysis were advanced for analysis in the GHS dataset, but only results for the top 44 genes were available. Mask: the grouping of alleles used in the GHS analysis; Var: the number of alleles in the mask; CAF: the combined allele frequency of all alleles in the mask; OR: the aggregate odds-ratio calculated from a burden analysis of the mask; Burden: the (two-sided) *p*-value from a burden analysis of the mask. M1: predicted loss-of-function variants, according to the Variant Effect Predictor, with MAF$<$1% (similar to the LofTee mask but without an additional filter on LofTee and with an additional filter on MAF); M2: nonsynonymous variants predicted deleterious by 5/5 prediction algorithms with MAF$<$1% (similar to the 5/5 mask but with an additional filter on MAF); M3: all nonsynonymous variants predicted deleterious by $\geq$1/5 bioinformatic algorithms with MAF$<$1% (similar to the 1/5 1% mask); M4: all nonsynonymous variants with MAF$<$1% (similar to the 0/5 1% mask); Best: the result of applying the minimum *p*-value test across all four masks, as described in **Methods**.

[See separate Excel file]

**Supplementary Table 7**: **Meta-analysis and evaluation of association signal concordance in CHARGE and GHS.** For each of the 50 genes from our exome sequence analysis with lowest gene-level *p*-value (N=43,071 unrelated individuals), we conducted a sample-size weighted meta-analysis among our analysis and those in the CHARGE (N=12,467 individuals) and GHS datasets (N=49,199 individuals). We in addition compared the direction of effect among the three analyses; in this comparison, we only included genes which achieved $p<0.05$ for the burden test (i.e. we excluded genes significant under SKAT but not the burden test). In both the meta-analysis and the direction of effect comparison, for each gene we analyzed the mask achieving the lowest *p*-value in our original analysis (for GHS, as discussed in **Methods**, we matched the LofTee mask to M1; the 15/15, 10/10, 5/5, and 5/5+LofTee LC mask to M2; the 1/5 1% mask to M3; and the 0/5 1% mask to M4). Best Test, $\log(OR)$, and P: the test with the lowest *p*-value within the chosen mask as well as the logarithm of the estimated odds-ratio and *p*-value; for genes in which the lowest *p*-value is achieved by the SKAT test, no direction of effect is shown and no comparison with CHARGE and GHS is performed (genes achieving $p<0.05$ for both SKAT and burden analyses are shown as two separate rows of the table). CHARGE Var (CAC, Score, P): the number of alleles (combined allele count, score statistic, and *p*-value) in the analogous mask in the CHARGE analysis. GHS Var (CAF, $\log(OR)$, P): the number of alleles (combined allele frequency, logarithm of odds-ratio, *p*-value) in the matched mask in the GHS analysis. Meta-analysis Dir: the direction of effect in the exomes, CHARGE, and GHS analysis ($+$ indicates effect size $>0$, $-$ indicates effect size $<0$). Meta-analysis P: the *p*-value from the meta-analysis. All *p*-values are two-sided.

[See separate Excel file]

**Supplementary Table 8**: ***UBE2NL*** **variants.** The *UBE2NL* burden signal achieved (two-sided) $p = 1.0 \times 10^{-5}$ in our original analysis (N=43,071 unrelated individuals) and reached exome-wide significance after meta-analysis with the CHARGE results. Shown are the variants contributing to the original burden signal (prior to meta-analysis). Columns are analogous to those in **Extended Data Item 5**. *P*-values are two-sided.

| Gene | Variant | Consequence | Impact | MAF | Case | Ctrl | OR | P |
|---|---|---|---|---|---|---|---|---|
| *UBE2NL* | rs201008812 | stop_gained | High | 0.00027 | 12 | 0 | 3.04 | 0.048 |
| *UBE2NL* | var_X_142967469 | stop_lost | High | 0.00022 | 7 | 0 | 2.09 | 0.058 |
| *UBE2NL* | var_X_142967455 | frameshift_variant | High | 0.00047 | 7 | 0 | 1.66 | 0.22 |
| *UBE2NL* | var_X_142967605 | frameshift_variant | High | 0.00013 | 2 | 0 | 7.57 | 0.3 |
| *UBE2NL* | rs368038086 | stop_gained | High | 6.7e-05 | 1 | 1 | 0.802 | 0.95 |

**Supplementary Table 9**: **Genes included in gene set analysis.** We selected various sets of genes, as described in **Methods**, to test for stronger-than-expected gene-level associations. Shown are the set of genes used to define each gene set.

[See separate Excel file]

**Supplementary Table 10**: **Gene-level associations within the Monogenic gene set.** Shown are gene-level association results (two-sided, N=43,071 unrelated individuals) for all genes within the Monogenic gene set. Columns are analogous to those in **Extended Data Item 8**. The number of variants (Var) and combined allele frequency (CAF) are shown separately for the mask with lowest *p*-value (chosen by the minimum *p*-value test) as well as for the 0/5 1% mask (used in the weighted test).

| | Burden | | | | | | | | SKAT | |
|---|---|---|---|---|---|---|---|---|---|---|
| | Min P | | | | Weighted | | | | Min P | Weighted |
| Gene | Var | CAF | OR | P | Var | CAF | OR | P | P | P |
| *PDX1* | 11 | 0.000371 | 4.71 | 0.0272 | 65 | 0.00893 | 3.45 | 0.000166 | 0.165 | 0.0573 |
| *GCK* | 5 | 0.000162 | 38.5 | 0.00823 | 69 | 0.00364 | 2.47 | 0.00641 | 0.746 | 0.543 |
| *HNF1A* | 131 | 0.0184 | 1.23 | 0.0274 | 131 | 0.0184 | 1.47 | 0.0089 | 3.62e-05 | 0.00106 |
| *PAX4* | 11 | 0.00151 | 2.16 | 0.0237 | 124 | 0.0421 | 1.14 | 0.148 | 0.342 | 0.34 |
| *KLF11* | 146 | 0.0189 | 1.19 | 0.0793 | 146 | 0.0189 | 1.32 | 0.127 | 0.403 | 0.221 |
| *INS* | 3 | 6.96e-05 | 20.9 | 0.134 | 5 | 0.000672 | 1.19 | 0.852 | 0.875 | 0.962 |
| *BLK* | 161 | 0.0351 | 1.1 | 0.225 | 192 | 0.042 | 1.16 | 0.145 | 0.739 | 0.678 |
| *NEUROD1* | 15 | 0.000765 | 1.98 | 0.145 | 81 | 0.00897 | 1.17 | 0.532 | 0.0266 | 0.22 |
| *HNF1B* | 30 | 0.00283 | 1.35 | 0.535 | 127 | 0.0118 | 1.23 | 0.268 | 0.996 | 0.963 |
| *HNF4A* | 15 | 0.00111 | 1.4 | 0.884 | 115 | 0.0139 | 1.24 | 0.358 | 0.905 | 0.422 |
| *CEL* | 52 | 0.00283 | 1.32 | 0.58 | 155 | 0.0248 | 1.11 | 0.561 | 0.339 | 0.148 |

**Supplementary Table 11**: **Dissection of gene set associations.** Shown are statistics on the number of genes that contribute to each gene set association signal (as reported in **Supplementary Figure 10**). In addition to examining the number of genes in the gene set with gene-level *p* (as calculated by the [two-sided] minimum *p*-value test) reaching various thresholds, we also conducted an analysis in which we progressively removed genes from the gene set (in order of increasing *p*-value) and recalculated the overall gene set association using a one-sided Wilcoxon test. Total # genes: the number of genes in the gene set (**Supplementary Table 9**). # (%) w/ $p<0.05$ (0.2, 0.5): the number (%) of genes in the gene-set with gene-level $p<0.05$ (0.2, 0.5). # (%) to ablate signal: the number (%) of genes removed from the gene set, during the progressive analysis, at which point the gene set no longer achieved $p<0.05$. Analysis N=43,071 unrelated individuals.

| Gene set | Total | w/ p<0.05 | | w/ p<0.2 | | w/ p<0.5 | | to ablate signal | |
|---|---|---|---|---|---|---|---|---|---|
| | # genes | # | % | # | % | # | % | # | % |
| Monogenic | 11 | 3 | 27.3% | 6 | 54.5% | 9 | 81.8% | 4 | 36.4% |
| Drug targets | 8 | 1 | 12.5% | 6 | 75.0% | 7 | 87.5% | 4 | 50.0% |
| GWAS genes | 11 | 2 | 18.2% | 3 | 27.3% | 10 | 90.9% | 2 | 18.2% |
| Mouse NIDD | 31 | 5 | 16.1% | 11 | 35.5% | 20 | 64.5% | 4 | 12.9% |
| Mouse impaired glucose tolerance | 323 | 29 | 9.0% | 87 | 26.9% | 192 | 59.4% | 30 | 9.3% |
| Mouse improved glucose tolerance | 238 | 11 | 4.6% | 59 | 24.8% | 139 | 58.4% | 11 | 4.6% |
| Mouse increased circulating glucose | 360 | 35 | 9.7% | 80 | 22.2% | 194 | 53.9% | 4 | 1.1% |
| Mouse decreased circulating glucose | 477 | 29 | 6.1% | 101 | 21.2% | 243 | 50.9% | 0 | 0.0% |
| Mouse insulin resistance | 179 | 13 | 7.3% | 42 | 23.5% | 98 | 54.7% | 1 | 0.5% |
| Mouse increased insulin sensitivity | 176 | 11 | 6.2% | 45 | 25.6% | 103 | 58.5% | 6 | 3.4% |
| Mouse decreased insulin secretion | 132 | 13 | 9.8% | 40 | 30.3% | 78 | 59.1% | 11 | 8.3% |
| Mouse increased insulin secretion | 51 | 2 | 3.9% | 11 | 21.6% | 28 | 54.9% | 0 | 0.0% |
| Mouse decreased circulating insulin | 318 | 24 | 7.5% | 75 | 23.6% | 180 | 56.6% | 17 | 5.3% |
| Mouse increased circulating insulin | 214 | 19 | 8.9% | 49 | 22.9% | 106 | 49.5% | 0 | 0.0% |

**Supplementary Table 12**: **Genes within T2D GWAS loci with nominally significant gene-level associations.** Shown are all genes within established T2D GWAS loci that achieved a $p<0.05$ for the minimum $p$-value burden analysis (two-sided, N=43,071 unrelated individuals). Columns are analogous to those in **Extended Data Item 8**. Locus: an identifier for the T2D GWAS locus containing the gene.

| | | | | Burden | | | | SKAT | |
|---|---|---|---|---|---|---|---|---|---|
| | | Best Result | | Min P | | Weighted | | Min P | Weighted |
| Gene | Locus | Var | CAF | OR | P | OR | P | P | P |
| *MC4R* | *MC4R* | 41 | 0.00795 | 2.07 | 2.74e-10 | 2.2 | 4.81e-09 | 7.74e-08 | 3.48e-08 |
| *PAM* | *PAM* | 79 | 0.0493 | 1.31 | 1.58e-08 | 1.44 | 2.2e-09 | 1.53e-07 | 7.03e-08 |
| *SLC30A8* | *SLC30A8* | 86 | 0.0116 | 0.598 | 1.85e-07 | 0.397 | 1.29e-08 | 0.00011 | 0.000221 |
| *MNT* | *SRR* | 105 | 0.00939 | 0.708 | 0.00131 | 0.444 | 0.0151 | 0.962 | 0.922 |
| *ZFP1* | *BCAR1* | 120 | 0.0134 | 0.762 | 0.00664 | 0.552 | 0.00755 | 0.157 | 0.0561 |
| *SLC30A3* | *GCKR* | 74 | 0.00823 | 1.44 | 0.00699 | 2.19 | 0.00808 | 0.189 | 0.00168 |
| *RASGRP1* | *RASGRP1* | 22 | 0.000812 | 3.1 | 0.00879 | 1.63 | 0.123 | 0.856 | 0.693 |
| *TH* | *IGF2* | 35 | 0.00223 | 0.509 | 0.00879 | 0.641 | 0.003 | 0.0601 | 0.0121 |
| *WFS1* | *WFS1* | 120 | 0.146 | 1.09 | 0.0118 | 1.12 | 0.000944 | 0.0261 | 0.00167 |
| *ACADS* | *HNF1A* | 53 | 0.00684 | 1.44 | 0.013 | 1.41 | 0.0175 | 0.472 | 0.719 |
| *NUDCD3* | *GCK* | 60 | 0.0048 | 1.5 | 0.013 | 1.77 | 0.0239 | 0.825 | 0.758 |
| *FSCN3* | *GCC1/PAX4* | 35 | 0.0029 | 1.7 | 0.0145 | 1.25 | 0.115 | 0.123 | 0.237 |
| *BCL11A* | *BCL11A* | 115 | 0.00737 | 1.35 | 0.0166 | 1.73 | 0.0466 | 0.413 | 0.263 |
| *INS-IGF2* | *IGF2* | 43 | 0.00728 | 0.743 | 0.0187 | 0.462 | 0.0708 | 0.239 | 0.179 |
| *TMEM19* | *LGR5* | 73 | 0.0164 | 1.25 | 0.0213 | 1.52 | 0.00955 | 0.154 | 0.0752 |
| *PDX1* | *PDX1* | 11 | 0.000371 | 4.71 | 0.0214 | 3.46 | 0.000166 | 0.165 | 0.0573 |
| *SYN2* | *PPARG* | 36 | 0.00167 | 0.5 | 0.0214 | 0.689 | 0.138 | 0.556 | 0.274 |
| *HNF1A* | *HNF1A* | 131 | 0.0184 | 1.23 | 0.0219 | 1.47 | 0.0125 | 3.62e-05 | 0.00106 |
| *IGF2* | *IGF2* | 53 | 0.00457 | 0.66 | 0.0235 | 0.264 | 0.0912 | 0.867 | 0.649 |
| *PHLPP1* | *BCL2* | 271 | 0.0501 | 0.898 | 0.0236 | 0.822 | 0.156 | 0.261 | 0.182 |
| *PAX4* | *GCC1/PAX4* | 11 | 0.00151 | 2.16 | 0.0237 | 1.14 | 0.176 | 0.342 | 0.34 |
| *HIBADH* | *JAZF1* | 65 | 0.00459 | 1.38 | 0.0265 | 1.98 | 0.0551 | 0.255 | 0.24 |
| *POLM* | *GCK* | 180 | 0.0597 | 1.12 | 0.0266 | 1.1 | 0.101 | 0.704 | 0.379 |
| *MEF2B* | *CILP2* | 20 | 0.00162 | 1.92 | 0.0294 | 1.35 | 0.349 | 0.698 | 0.7 |
| *VPS33B* | *PRC1* | 23 | 0.0013 | 1.97 | 0.0306 | 1.21 | 0.193 | 0.025 | 0.577 |
| *PLEKHH2* | *THADA* | 146 | 0.0185 | 1.21 | 0.031 | 1.22 | 0.0167 | 0.165 | 0.0441 |
| *CCND2* | *CCND2* | 50 | 0.00408 | 1.47 | 0.0329 | 1.34 | 0.741 | 0.0632 | 0.0971 |
| *MYCBP* | *MACF1* | 26 | 0.00339 | 0.643 | 0.0344 | 0.593 | 0.0373 | 0.0999 | 0.0653 |
| *FAM135A* | *C6orf57* | 7 | 0.000348 | 4.39 | 0.0371 | 1.08 | 0.46 | 0.0627 | 0.113 |
| *DNLZ* | *GPSM1* | 40 | 0.00821 | 1.32 | 0.0373 | 1.77 | 0.0613 | 0.386 | 0.159 |
| *ATG16L2* | *ARAP1* | 166 | 0.0293 | 1.17 | 0.038 | 1.26 | 0.0101 | 0.982 | 0.868 |
| *UBE2E2* | *UBE2E2* | 23 | 0.00371 | 1.5 | 0.0391 | 1.51 | 0.0355 | 0.879 | 0.733 |
| *BORCS8-MEF2B* | *CILP2* | 21 | 0.00165 | 1.96 | 0.0397 | 1.36 | 0.275 | 0.888 | 0.634 |
| *ATXN7* | *PSMD6* | 199 | 0.106 | 1.08 | 0.0428 | 1.12 | 0.0192 | 0.0651 | 0.0288 |
| *LPP* | *LPP* | 160 | 0.0284 | 0.855 | 0.0432 | 0.754 | 0.00952 | 0.592 | 0.132 |
| *ZNF14* | *CILP2* | 106 | 0.0109 | 0.794 | 0.0432 | 0.728 | 0.0392 | 0.911 | 0.842 |
| *FAH* | *ZFAND6* | 119 | 0.0447 | 1.15 | 0.0437 | 1.16 | 0.0399 | 0.00492 | 0.00104 |
| *PHGDH* | *NOTCH2* | 50 | 0.0115 | 0.791 | 0.0445 | 0.792 | 0.0304 | 0.576 | 0.233 |
| *GLP1R* | *KCNK16* | 17 | 0.00116 | 1.97 | 0.0465 | 1.72 | 0.0194 | 0.0738 | 0.0171 |
| *ST20-MTHFS* | *ZFAND6* | 48 | 0.00631 | 0.742 | 0.0468 | 0.78 | 0.31 | 0.818 | 0.587 |

**Supplementary Table 13**: **Sample and variant counts for imputed GWAS analysis.** Shown are the sample subgroups with SNP array data analyzed as part of the imputed GWAS analysis. Subgroup, Ancestry, Sequence tech: subgroup characteristics. SNP array: technology used for imputed GWAS genotyping. Samples (Cases, Ctrls): Number of samples (T2D cases, controls) included in the imputed GWAS analysis. Variants: Number of variants passing quality control and included in the imputed GWAS analysis. Prior to analysis, all subgroups had genotypes imputed from the 1000G Phase 3 reference panel.

| Subgroup | Ancestry | Sequence tech | SNP array | Samples | Cases | Ctrls | Variants |
|---|---|---|---|---|---|---|---|
| Wake Forest | African-American | Agilent | Affy6 | 1053 | 531 | 522 | 27,973,694 |
| JHS | African-American | Agilent | Affy6 | 989 | 513 | 476 | 27,748,674 |
| BioMe | African-American | Illumina | G4L | 2518 | 1233 | 1285 | 26,084,594 |
| Singapore | East-Asian | Agilent | Illumina610/Illumina1M | 1077 | 591 | 486 | 10,897,305 |
| Singapore | East-Asian | Illumina | G4L | 1969 | 985 | 984 | 9,209,756 |
| KARE | East-Asian | Agilent | Affy5 | 1096 | 567 | 529 | 7,770,075 |
| SNUH | East-Asian | Illumina | G4L | 917 | 474 | 443 | 8,839,178 |
| Hong Kong | East-Asian | Illumina | G4L | 960 | 481 | 479 | 9,231,055 |
| GoT2D | European | Agilent | HumanOmni2.5 | 2657 | 1326 | 1331 | 17,692,443 |
| METSIM | European | Agilent | HumanOmni2.5 | 970 | 494 | 476 | 14,135,914 |
| Ashkenazi | European | Agilent | Illumina Cardio-MetaboChip | 732 | 359 | 373 | 2,602,793 |
| GoDARTS | European | Illumina | G4L | 1886 | 941 | 945 | 12,197,555 |
| FHS | European | Illumina | G4L | 973 | 584 | 389 | 10,939,434 |
| SIGMA | Latino | Agilent | HumanOmni2.5 | 3542 | 1712 | 1830 | 35,256,845 |
| SIGMA | Latino | Illumina | G4L | 5851 | 3020 | 2831 | 19,591,358 |
| Starr County | Hispanic | Agilent | Affy6 | 1383 | 673 | 710 | 20,401,781 |
| Starr County | Hispanic | Illumina | G4L | 933 | 608 | 325 | 16,157,686 |
| San Antonio | Hispanic | Agilent | Illumina Cardio-MetaboChip | 445 | 202 | 243 | 2,996,739 |
| Singapore | South-Asian | Agilent | Illumina610 | 1112 | 576 | 536 | 14,471,389 |
| Singapore | South-Asian | Illumina | G4L | 1932 | 882 | 1050 | 11,989,365 |
| LOLIPOP | South-Asian | Agilent | Illumina610 | 1199 | 599 | 600 | 15,256,850 |
| PGR | South-Asian | Illumina | G4L | 1718 | 882 | 836 | 12,580,193 |

**Supplementary Table 14**: **Loci with most significant associations from the imputed GWAS analysis.** Shown are the most significant associations from the imputed GWAS analysis (N=34,529 individuals), with only one association shown per 250kb of genomic sequence. Closest Gene: the closest gene to the variant. rsID: the dbSNP ID of the variant (as predicted by the Variant Effect Predictor), if applicable. Chrom/Position: the chromosome and position of the variant. E.A./O.A.: the effect and non-effect alleles of the variant. Samples: the number of samples analyzed for the variant (i.e. the number of samples within subgroups in which the variant was polymorphic and passed quality control). MAF: the minor allele frequency of the variant, calculated across all samples. OR: the estimated odds-ratio of the variant. P: the *p*-value of the variant, calculated by a (two-sided) Firth logistic regression.

| Closest Gene | rsID | Chrom | Position | E.A. | O.A. | Samples | MAF | OR | P |
|---|---|---|---|---|---|---|---|---|---|
| *TCF7L2* | rs7903146 | 10 | 114758349 | T | C | 30683 | 0.25 | 1.34 | 1.48e-41 |
| *KCNQ1* | rs2237896 | 11 | 2858440 | A | G | 27249 | 0.26 | 0.734 | 1.23e-32 |
| *CDC123* | rs11257600 | 10 | 12255657 | T | G | 34529 | 0.31 | 1.16 | 6.14e-17 |
| *CDKAL1* | rs9460550 | 6 | 20719561 | A | G | 34527 | 0.32 | 1.15 | 6.63e-15 |
| *SLC30A8* | rs3802177 | 8 | 118185025 | A | G | 27745 | 0.31 | 0.862 | 7.7e-14 |
| *IGF2BP2* | rs4414887 | 3 | 185506892 | T | C | 33555 | 0.32 | 1.13 | 3.72e-13 |
| *CTBP1* | rs72501962 | 4 | 1246038 | A | T | 33797 | 0.28 | 1.15 | 7.56e-12 |
| *ASCL2* | rs17737404 | 11 | 2270342 | A | G | 30237 | 0.23 | 1.22 | 3.7e-10 |
| *KCNJ11* | rs10734252 | 11 | 17404839 | A | G | 34524 | 0.38 | 0.9 | 3.83e-10 |
| *HNF4A* | rs6103716 | 20 | 42999630 | A | C | 34529 | 0.39 | 0.892 | 4.54e-10 |
| *KIF11* | rs2153827 | 10 | 94424073 | T | G | 34442 | 0.34 | 1.11 | 9.97e-10 |
| *ZMIZ1* | rs703978 | 10 | 80944147 | C | G | 34528 | 0.31 | 1.11 | 1.91e-09 |
| *IRS1* | rs2943657 | 2 | 227123439 | T | C | 34529 | 0.26 | 1.12 | 2.82e-09 |
| *JAZF1* | rs1635852 | 7 | 28189411 | T | C | 27745 | 0.34 | 1.12 | 3.67e-09 |
| *SFI1* | rs2236033 | 22 | 32001050 | A | G | 34511 | 0.3 | 1.11 | 4.37e-09 |
| *GPSM1* | rs376993806 | 9 | 139246588 | A | G | 29705 | 0.26 | 0.885 | 1.82e-08 |
| *SPRY2* | rs1359790 | 13 | 80717156 | A | G | 34529 | 0.31 | 0.896 | 2.27e-08 |
| *EML4* | - | 2 | 42506923 | T | TA | 2657 | 0.44 | 1.43 | 3.02e-08 |
| *PPARG* | rs4684848 | 3 | 12395645 | A | G | 34529 | 0.2 | 0.883 | 4.11e-08 |
| *WFS1* | - | 4 | 6301628 | T | TTG | 28470 | 0.21 | 1.14 | 4.45e-08 |
| *SOX11* | rs896911 | 2 | 5376965 | T | C | 32372 | 0.38 | 0.908 | 5.84e-08 |
| *CCND2* | rs76895963 | 12 | 4384844 | T | G | 4826 | 0.023 | 2.84 | 1.14e-07 |
| *AUTS2* | - | 7 | 69534694 | G | GT | 29821 | 0.23 | 0.878 | 1.17e-07 |
| *NTRK2* | rs1573219 | 9 | 87387622 | A | G | 26568 | 0.3 | 0.903 | 2.1e-07 |
| *COBLL1* | rs12692738 | 2 | 165558252 | T | C | 34529 | 0.23 | 1.12 | 2.25e-07 |

**Supplementary Table 15**: **Most significant nonsynonymous variants within T2D GWAS loci.** Shown are the 50 nonsynonymous variants within established T2D GWAS loci that achieved the lowest *p*-values in the exome sequence single-variant analysis (two-sided, N=45,231 individuals). Columns are analogous to those in **Extended Data Item 5**. Locus: an identifier for the T2D GWAS locus containing the variant.

| Gene | Locus | Variant | Consequence | Change | MAF | Case | Ctrl | OR | P |
|---|---|---|---|---|---|---|---|---|---|
| *PAX4* | *GCC1/PAX4* | rs2233580 | missense_variant | p.Arg192His | 0.12 | 890 | 563 | 1.7 | 7.6e-22 |
| *SLC30A8* | *SLC30A8* | rs13266634 | missense_variant | p.Arg325Trp | 0.43 | 12258 | 13756 | 0.897 | 3.4e-11 |
| *WFS1* | *WFS1* | rs1801212 | missense_variant | p.Val333Ile | 0.27 | 7101 | 8456 | 1.13 | 1.2e-10 |
| *KCNJ11* | *KCNJ11* | rs5215 | missense_variant | p.Val250Ile | 0.39 | 16687 | 16132 | 0.901 | 3.4e-10 |
| *ABCC8* | *KCNJ11* | rs757110 | missense_variant | p.Ala1369Ser | 0.39 | 16626 | 16237 | 0.913 | 7.1e-08 |
| *MC4R* | *MC4R* | rs79783591 | missense_variant | p.Ile269Asn | 0.0089 | 195 | 83 | 2.17 | 3.4e-07 |
| *COBLL1* | *COBLL1/GRB14* | rs7607980 | missense_variant | p.Asn939Asp | 0.15 | 4010 | 4651 | 0.857 | 6.3e-07 |
| *PAM* | *PAM* | rs35658696 | missense_variant | p.Asp563Gly | 0.05 | 1038 | 944 | 1.29 | 1.3e-06 |
| *PPIP5K2* | *PAM* | rs36046591 | missense_variant | p.Ser1207Gly | 0.049 | 986 | 905 | 1.3 | 1.4e-06 |
| *GCKR* | *GCKR* | rs1260326 | missense_variant | p.Leu446Pro | 0.5 | 15010 | 16627 | 1.07 | 5.4e-06 |
| *TM6SF2* | *CILP2* | rs58542926 | missense_variant | p.Glu167Lys | 0.1 | 2899 | 2694 | 1.14 | 2.6e-05 |
| *FES* | *PRC1* | var_15_91434859 | missense_variant | p.Pro536Ser | 0.0071 | 63 | 24 | 1.82 | 3.1e-05 |
| *SENP2* | *IGF2BP2* | rs6762208 | missense_variant | p.Thr301Lys | 0.49 | 18375 | 17667 | 1.07 | 3.8e-05 |
| *PPARG* | *PPARG* | rs1801282 | missense_variant | p.Pro12Ala | 0.12 | 4241 | 4935 | 0.894 | 6.5e-05 |
| *HNF1A* | *HNF1A* | var_12_121437091 | missense_variant | p.Glu508Lys | 0.006 | 93 | 33 | 2.25 | 9.1e-05 |
| *TCF19* | *HLA-B* | rs2073721 | missense_variant | p.Met211Val | 0.3 | 11485 | 11721 | 1.07 | 0.00013 |
| *NUCB2* | *KCNJ11* | rs757081 | missense_variant | p.Gln338Glu | 0.36 | 13223 | 13362 | 1.08 | 0.00017 |
| *RREB1* | *SSR1/RREB1* | rs9379084 | missense_variant | p.Asp1171Asn | 0.13 | 3641 | 4117 | 0.916 | 0.00026 |
| *NUCB2* | *KCNJ11* | rs3842269 | inframe_deletion | p.401-402LeuGln/Leu | 0.5 | 10255 | 11247 | 0.934 | 0.0003 |
| *GPSM1* | *GPSM1* | var_9_139235415 | missense_variant | p.391-392SerGlu/LeuGlu | 0.28 | 8404 | 8458 | 0.931 | 0.00033 |
| *C6orf136* | *POU5F1/TCF19* | rs150233869 | missense_variant | p.Arg220Cys | 0.0086 | 69 | 92 | 0.527 | 0.00038 |
| *GTF2H4* | *POU5F1/TCF19* | rs140816086 | missense_variant | p.Arg453His | 0.001 | 16 | 34 | 0.476 | 0.00043 |
| *SDCCAG3* | *GPSM1* | rs1131992 | missense_variant | p.Val356Met | 0.26 | 7300 | 7616 | 0.928 | 0.00043 |
| *THADA* | *THADA* | rs35720761 | missense_variant | p.Cys1605Tyr | 0.15 | 4491 | 4736 | 0.927 | 0.00048 |
| *WFS1* | *WFS1* | rs734312 | missense_variant | p.Arg611His | 0.89 | 21467 | 21951 | 1.05 | 0.0005 |
| *RBL2* | *FTO* | rs199555150 | missense_variant | p.Ser995Gly | 0.01 | 51 | 97 | 0.619 | 0.0005 |
| *HNF1A* | *HNF1A* | rs1800574 | missense_variant | p.Ala98Val | 0.061 | 1059 | 915 | 1.17 | 0.00054 |
| *VPS33B* | *PRC1* | rs11073964 | missense_variant | p.Gly514Ser | 0.58 | 13333 | 15422 | 1.07 | 0.00071 |
| *NOTCH1* | *GPSM1* | rs61751489 | missense_variant | p.Val2285Ile | 0.16 | 2748 | 2756 | 0.882 | 0.00079 |
| *SDCCAG3* | *GPSM1* | rs3812577 | missense_variant | p.Arg281Gln | 0.26 | 7124 | 7422 | 0.929 | 0.00086 |
| *C6orf15* | *HLA-B* | rs2233977 | missense_variant | p.Val81Ala | 0.37 | 9445 | 9951 | 0.947 | 0.00086 |
| *ACMSD* | *TMEM163* | var_2_135621062 | missense_variant | p.Thr116Met | 0.0032 | 9 | 29 | 0.462 | 0.00089 |
| *IFT172* | *GCKR* | rs139229844 | missense_variant | p.Gln866Arg | 0.0017 | 27 | 5 | 3. | 0.00089 |
| *LST1* | *HLA-B* | rs184203129 | missense_variant | p.Ala46Thr | 0.00067 | 16 | 4 | 2.96 | 0.00093 |
| *TM6SF2* | *CILP2* | rs187429064 | missense_variant | p.Leu156Pro | 0.019 | 415 | 363 | 1.3 | 0.00093 |
| *SLC30A8* | *SLC30A8* | rs73317647 | missense_variant | p.Arg165Cys | 0.0061 | 34 | 70 | 0.516 | 0.001 |
| *MUC22* | *POU5F1* | rs117024916 | missense_variant | p.Thr71Ala | 0.013 | 80 | 112 | 0.638 | 0.0012 |
| *FAT3* | *MTNR1B* | var_11_92620216 | missense_variant | p.Lys665Glu | 0.0013 | 9 | 31 | 0.51 | 0.0015 |
| *GATAD2A* | *CILP2* | rs370240766 | inframe_insertion | p.67Thr/ThrAlaMet | 0.00066 | 4 | 15 | 0.464 | 0.0016 |
| *RCCD1* | *PRC1* | rs75390535 | missense_variant | p.Leu249Val | 0.099 | 583 | 701 | 0.828 | 0.0016 |
| *MC4R* | *MC4R* | var_18_58038669 | missense_variant | p.Arg305Gln | 0.00033 | 8 | 0 | 4.89 | 0.0017 |
| *FSCN3* | *GCC1/PAX4* | rs144391719 | missense_variant | p.Arg356His | 0.00023 | 11 | 1 | 3.85 | 0.0018 |
| *FLT3* | *PDX1* | rs62636526 | missense_variant | p.Val16Leu | 0.0089 | 94 | 52 | 1.7 | 0.0018 |
| *MDGA1* | *ZFAND3* | rs143644874 | missense_variant | p.Glu756Gln | 0.0069 | 54 | 72 | 0.492 | 0.0018 |
| *WFS1* | *WFS1* | rs1801208 | missense_variant | p.Arg456His | 0.091 | 3063 | 2828 | 1.1 | 0.0019 |
| *C6orf15* | *HLA-B* | rs2233978 | missense_variant | p.Ala145Pro | 0.37 | 7733 | 7907 | 0.938 | 0.0019 |
| *SLC30A8* | *SLC30A8* | rs145677283 | missense_variant | p.Arg165His | 0.0014 | 15 | 34 | 0.447 | 0.0021 |
| *LAMA1* | *LAMA1* | rs115759032 | missense_variant | p.Leu1932Val | 0.006 | 33 | 60 | 0.564 | 0.0021 |
| *CARD9* | *GPSM1* | rs4077515 | missense_variant | p.Ser12Asn | 0.54 | 19891 | 20405 | 0.956 | 0.0022 |
| *F2RL1* | *ZBED3* | rs148584357 | missense_variant | p.His135Arg | 0.00026 | 8 | 0 | 5.74 | 0.0022 |

**Supplementary Table 16**: **Most significant protein-truncating variants within T2D GWAS loci.** Shown are all protein-truncating variants (as annotated by the Variant Effect Predictor) within established T2D GWAS loci that achieved $p<0.05$ in the exome sequence single-variant analysis (two-sided, N=45,231 individuals). Columns are analogous to those in **Supplementary Table 15**.

| Gene | Locus | Variant | Consequence | Change | MAF | Case | Ctrl | OR | P |
|---|---|---|---|---|---|---|---|---|---|
| *STOX1* | *VPS26A* | var_10_70644927 | stop_gained | p.Gln459Ter | 0.00066 | 8 | 0 | 4.85 | 0.0034 |
| *CRIPAK* | *MAEA* | var_4_1388335 | stop_gained | p.Cys12Ter | 0.0045 | 65 | 101 | 0.657 | 0.004 |
| *PPM1N* | *GIPR* | var_19_46005322 | frameshift_variant | p.-106-107Ter | 0.001 | 7 | 23 | 0.323 | 0.0046 |
| *CDSN* | *HLA-B* | var_6_31084723 | frameshift_variant | p.218-223CysSerSerAspIlePro/Ter | 0.016 | 91 | 142 | 0.707 | 0.0064 |
| *THADA* | *THADA* | var_2_43817961 | splice_acceptor_variant | - | 0.0004 | 14 | 9 | 0.238 | 0.0073 |
| *THADA* | *THADA* | var_2_43817962 | splice_acceptor_variant | - | 0.0004 | 12 | 9 | 0.239 | 0.0075 |
| *IFT172* | *GCKR* | rs150246251 | stop_gained | p.Arg1507Ter | 0.00024 | 4 | 0 | 5.34 | 0.011 |
| *ABCB9* | *MPHOSPH9* | var_12_123419979 | splice_acceptor_variant | - | 0.00019 | 4 | 0 | 8.12 | 0.013 |
| *DPY19L4* | *TP53INP1* | rs200830188 | splice_acceptor_variant | - | 0.0011 | 10 | 2 | 3.71 | 0.014 |
| *SNAPC4* | *GPSM1* | var_9_139278009 | frameshift_variant | p.Ser537Ter | 0.00042 | 5 | 4 | 5.19 | 0.017 |
| *C11orf21* | *IGF2* | rs3214127 | frameshift_variant | p.-119-120Ter | 0.034 | 166 | 130 | 1.29 | 0.017 |
| *SPATA31D5P* | *TLE1* | var_9_84528573 | splice_acceptor_variant | - | 0.00034 | 0 | 8 | 0.0938 | 0.017 |
| *RBM28* | *LEP* | var_7_127961426 | stop_gained | p.Arg486Ter | 0.00025 | 0 | 3 | 0.118 | 0.018 |
| *KRTCAP3* | *GCKR* | rs140428163 | frameshift_variant | p.119Leu/LeuTer | 0.001 | 10 | 20 | 0.437 | 0.019 |
| *C15orf53* | *RASGRP1* | var_15_38988925 | frameshift_variant | p.39-40AlaSer/AlaTer | 0.006 | 63 | 54 | 1.32 | 0.02 |
| *SNX17* | *GCKR* | var_2_27599220 | frameshift_variant | p.408-410AspSerGln/Ter | 0.002 | 46 | 24 | 1.64 | 0.021 |
| *VPS13C* | *C2CD4A/C2CD4B* | var_15_62169177 | stop_gained | p.Glu3364Ter | 0.00016 | 6 | 0 | 5.3 | 0.023 |
| *KCNQ1* | *KCNQ1* | rs11601907 | stop_gained | p.Tyr662Ter | 0.27 | 4247 | 4392 | 1.08 | 0.025 |
| *VPS33B* | *PRC1* | var_15_91553029 | splice_acceptor_variant | - | 0.00041 | 5 | 0 | 5.58 | 0.026 |
| *TIMP4* | *PPARG* | var_3_12195162 | stop_gained | p.Cys176Ter | 0.00065 | 7 | 1 | 3.55 | 0.026 |
| *SLC16A13* | *SLC16A11* | rs202121781 | stop_gained | p.Arg282Ter | 0.00017 | 5 | 0 | 2.65 | 0.028 |
| *PEX11A* | *AP3S2* | var_15_90229661 | splice_acceptor_variant | - | 0.00012 | 4 | 0 | 4.3 | 0.028 |
| *BLM* | *PRC1* | rs367543013 | frameshift_variant | p.256-257AspSer/AspTer | 0.00018 | 0 | 5 | 0.261 | 0.029 |
| *TIGD4* | *TMEM154* | var_4_153691112 | frameshift_variant | p.Phe348Ter | 0.00072 | 5 | 10 | 0.432 | 0.035 |
| *DHTKD1* | *CDC123/CAMK1D* | var_10_12159670 | splice_acceptor_variant | - | 0.0002 | 1 | 5 | 0.209 | 0.035 |
| *RHBDL2* | *MACF1* | var_1_39381293 | stop_gained | p.Tyr112Ter | 0.0014 | 29 | 13 | 1.85 | 0.036 |
| *ATG16L2* | *ARAP1* | var_11_72535166 | splice_acceptor_variant | - | 0.00013 | 4 | 0 | 8.64 | 0.036 |
| *COBLL1* | *COBLL1/GRB14* | var_2_165551295 | frameshift_variant | p.907Leu/PheTer | 0.019 | 81 | 70 | 0.331 | 0.037 |
| *KIF6* | *KCNK16* | rs202222855 | stop_gained | p.Ser244Ter | 0.00025 | 3 | 0 | 3.62 | 0.038 |
| *IGF2BP2* | *IGF2BP2* | var_3_185375092 | frameshift_variant | p.Phe456Ter | 0.00033 | 7 | 1 | 4.09 | 0.039 |
| *SNAPC4* | *GPSM1* | rs3812565 | frameshift_variant | p.1259L/LPQPGPEKGALDLEX | 0.46 | 15090 | 13994 | 0.958 | 0.039 |
| *ZNF14* | *CILP2* | var_19_19822199 | stop_gained | p.630-631PheArg/PheTer | 0.00025 | 0 | 2 | 0.229 | 0.04 |
| *KRTAP5-5* | *DUSP8* | var_11_1651596 | frameshift_variant | p.176-194SSCCKPYCCQSSCCKPYCC/X | 0.16 | 4304 | 4202 | 0.934 | 0.041 |
| *HMGCS2* | *NOTCH2* | rs1048438 | stop_gained | p.297Tyr/TerTyr | 0.00025 | 0 | 3 | 0.215 | 0.043 |
| *FAM135A* | *C6orf57* | var_6_71195923 | stop_gained | p.Arg250Ter | 0.00035 | 9 | 2 | 3.63 | 0.043 |
| *ZNF14* | *CILP2* | var_19_19822283 | stop_gained | p.Arg603Ter | 0.00012 | 0 | 3 | 0.251 | 0.043 |
| *OTOG* | *KCNJ11* | var_11_17631829 | frameshift_variant | p.Ser679Ter | 0.00042 | 0 | 7 | 0.155 | 0.044 |
| *NANOS2* | *GIPR* | var_19_46417550 | frameshift_variant | p.134Arg/ArgTer | 0.0003 | 1 | 5 | 0.428 | 0.045 |
| *SPDYE1* | *GCK* | var_7_44042207 | frameshift_variant | p.93Ser/SerTer | 9.8e-05 | 0 | 5 | 0.304 | 0.046 |
| *PMPCA* | *GPSM1* | var_9_139311505 | stop_gained | p.Tyr246Ter | 0.00013 | 3 | 0 | 0.986 | 0.046 |
| *P2RX7* | *HNF1A* | var_12_121603952 | stop_gained | p.Arg236Ter | 0.00016 | 1 | 6 | 0.353 | 0.047 |
| *PLIN1* | *AP3S2* | var_15_90213359 | stop_gained | p.Cys150Ter | 0.00013 | 0 | 4 | 0.183 | 0.047 |
| *FGF6* | *CCND2* | rs375467953 | initiator_codon_variant | p.Met1Leu | 0.00017 | 4 | 2 | 2.33 | 0.047 |
| *DDX52* | *HNF1B* | var_17_35985993 | stop_gained | p.Gln362Ter | 0.00013 | 1 | 4 | 0.181 | 0.047 |
| *PRR3* | *POU5F1/TCF19* | rs371871050 | stop_gained | p.Arg171Ter | 0.00082 | 8 | 2 | 2.51 | 0.048 |
| *CRIPAK* | *MAEA* | rs373049641 | stop_gained | p.Arg39Ter | 0.006 | 111 | 98 | 0.805 | 0.048 |
| *VPS4B* | *BCL2* | var_18_61067295 | frameshift_variant | p.259Leu/ProTer | 0.0002 | 6 | 0 | 6.53 | 0.048 |
| *KIF6* | *KCNK16* | var_6_39330288 | frameshift_variant | p.74Ser/CysTer | 0.00033 | 2 | 4 | 0.261 | 0.049 |
| *RFC4* | *ST6GAL1* | rs370046824 | splice_acceptor_variant | - | 0.00066 | 2 | 6 | 0.21 | 0.049 |
| *DHX16* | *POU5F1/TCF19* | rs368358552 | stop_gained | p.Gln370Ter | 0.00012 | 2 | 0 | 4.61 | 0.049 |

**Supplementary Table 17**: **Most significant variant associations within the Monogenic gene set.** Shown are the 25 nonsynonymous variants within the Monogenic gene set that achieved the lowest *p*-values in the exome sequence single-variant analysis (two-sided, N=45,231 individuals). Columns are analogous to those in **Extended Data Item 5**, with two additional columns added. The PPA column shows the posterior probability of causal association of the variant, calculated under a model (see **Methods**) where Monogenic diabetes genes have prior probability of T2D-relevance of 100%. The Clinvar column shows the clinical significance of the variant, as annotated in the Clinvar database [66]; variants not present in Clinvar are annotated with "-". †: Consequence of var_9_135946962 is p.694-727PVPPTGDSGAPPVTPTGDSETAPVPPTGDSGAPPPCAAHG*LRGPPRDPHG*LRDRPRAAHG*LRGPPX

| Gene | Variant | Cons | Impact | Change | MAF | Case | Ctrl | OR | P | PPA | Clinvar |
|---|---|---|---|---|---|---|---|---|---|---|---|
| *HNF1A* | var_12_121437091 | Mis | Med. | p.Glu508Lys | 0.006 | 93 | 33 | 2.25 | 9.1e-05 | 0.72 | Uncertain_significance |
| *BLK* | var_8_11400824 | Mis | Med. | p.Gln31Glu | 0.00058 | 0 | 7 | 0.17 | 0.0042 | 0.66 | - |
| *CEL* | var_9_135940077 | Mis | Med. | p.Thr93Pro | 0.0005 | 10 | 1 | 3.2 | 0.005 | 0.66 | - |
| *NEUROD1* | var_2_182543074 | Mis | Med. | p.Leu172Phe | 0.00035 | 11 | 1 | 4.08 | 0.0065 | 0.64 | - |
| *KLF11* | rs146238335 | Mis | Med. | p.Glu181Lys | 0.0045 | 46 | 30 | 1.58 | 0.0065 | 0.64 | Likely_benign |
| *KLF11* | rs61755332 | Mis | Med. | p.Ser124Cys | 0.00042 | 1 | 11 | 0.248 | 0.0071 | 0.63 | - |
| *HNF1A* | rs142318174 | Mis | Med. | p.Gly52Ala | 0.0034 | 20 | 30 | 0.416 | 0.01 | 0.59 | - |
| *CEL* | rs199524286 | Mis | Med. | p.Thr474Met | 0.0012 | 13 | 4 | 1.98 | 0.017 | 0.51 | - |
| *HNF4A* | rs371124358 | Mis | Med. | p.Arg310Gln | 0.003 | 6 | 0 | 4.44 | 0.018 | 0.5 | - |
| *BLK* | rs138547659 | Mis | Med. | p.Ile308Val | 0.0014 | 16 | 7 | 2.52 | 0.024 | 0.49 | - |
| *BLK* | rs147642493 | Mis | Med. | p.Lys11Asn | 6.0e-05 | 2 | 0 | 4.83 | 0.029 | 0.47 | - |
| *HNF1B* | var_17_36093697 | Mis | Med. | p.Asp221Gly | 0.00025 | 3 | 0 | 2.97 | 0.03 | 0.46 | - |
| *CEL* | var_9_135946962 | Stop | High | † | 0.0048 | 42 | 26 | 1.75 | 0.03 | 0.46 | - |
| *HNF1A* | var_12_121434124 | Mis | Med. | p.Gly339Ser | 0.003 | 26 | 11 | 1.69 | 0.032 | 0.46 | Uncertain_significance |
| *BLK* | var_8_11421595 | Mis | Med. | p.Arg499Pro | 0.00046 | 5 | 0 | 4.2 | 0.037 | 0.43 | - |
| *HNF1B* | var_17_36091622 | Mis | Med. | p.His337Asp | 9.7e-05 | 4 | 0 | 5.67 | 0.038 | 0.42 | - |
| *KLF11* | var_2_10188234 | Mis | Med. | p.Gln257Arg | 0.00013 | 4 | 0 | 3.68 | 0.039 | 0.42 | - |
| *PAX4* | var_7_127255014 | Mis | Med. | p.Glu86Lys | 0.00017 | 1 | 4 | 0.107 | 0.042 | 0.41 | - |
| *KLF11* | rs201432055 | Mis | Med. | p.Val374Met | 0.00058 | 6 | 1 | 2.73 | 0.044 | 0.4 | - |
| *KLF11* | var_2_10186292 | Mis | Med. | p.Ile20Val | 0.00058 | 2 | 6 | 0.285 | 0.045 | 0.4 | - |
| *KLF11* | rs138601862 | Mis | Med. | p.His178Tyr | 8.3e-05 | 1 | 0 | 2.9 | 0.045 | 0.4 | - |
| *PDX1* | var_13_28494624 | Mis | Med. | p.Leu117Val | 0.00013 | 4 | 0 | 9.25 | 0.046 | 0.4 | - |
| *KLF11* | var_2_10186319 | Mis | Med. | p.Arg29Trp | 0.00017 | 0 | 4 | 0.306 | 0.046 | 0.4 | - |
| *BLK* | var_8_11420510 | Stop | High | p.Trp401Ter | 9.7e-05 | 3 | 0 | 2.71 | 0.048 | 0.4 | - |
| *PDX1* | var_13_28498620 | Mis | Med. | p.Gly212Arg | 0.00021 | 6 | 0 | 2.42 | 0.049 | 0.39 | - |

**Supplementary Table 18**: **Posterior probability conversion table.** Based on *p*-values from the exome sequence analysis for nonsynonymous variants within established T2D GWAS loci, together with an independent analysis of a subset of these variants on the Illumina Exome Array, we estimated the posterior probability of association for arbitrary nonsynonymous variants within the exome sequence analysis. The posterior probability estimates are a function of the observed *p*-value in the (two-sided, N=45,231 individual) exome sequence single-variant analysis (rows in the table, with $-\log_{10}(p)$ shown in the first column) and the prior likelihood that the variant is associated with T2D. The prior likelihood, which quantifies belief in causal variant association before observing any results from our sequence analysis, can be specified in two ways. First (top two rows), via a "gene prior", or prior probability that loss of function of the gene is associated with T2D risk, which could be based on (for example) literature or experimental data implicating the gene in T2D pathogenesis. Second (third and fourth row), via a "variant prior", or the prior probability that the variant itself is associated with T2D risk. Calculations based on the gene prior (top two rows) use estimates from our allelic mask weights (**Methods**) that 33% of missense variants result in gene loss of function.

[See separate Excel file]

**Supplementary Table 19**: **Most significant variant associations within the Mouse NIDD gene set.** Shown are the 25 nonsynonymous variants within the Mouse NIDD gene set that achieved the lowest *p*-values in the exome sequence single-variant analysis (two-sided, N=45,231 individuals). Columns are analogous to those in **Supplementary Table 17**. Variant PPA calculations are based on a gene prior of 23.2%, as estimated from an empirical genetic association enrichment within the Mouse NIDD gene set as described in **Methods**.

| Gene | Variant | Cons | Impact | Change | MAF | Case | Ctrl | OR | P | PPA |
|---|---|---|---|---|---|---|---|---|---|---|
| *HNF1A* | var_12_121437091 | Mis | Med. | p.Glu508Lys | 0.006 | 93 | 33 | 2.25 | 9.1e-05 | 0.31 |
| *HNF1A* | rs1800574 | Mis | Med. | p.Ala98Val | 0.061 | 1059 | 915 | 1.17 | 0.00054 | 0.3 |
| *MADD* | rs140505071 | Mis | Med. | p.Arg117Ser | 0.00024 | 1 | 5 | 0.262 | 0.0033 | 0.27 |
| *MADD* | var_11_47304436 | Mis | Med. | p.Arg593His | 0.0014 | 14 | 3 | 1.93 | 0.0034 | 0.25 |
| *AR* | var_X_66765785 | Mis | Med. | p.Asp266Gly | 0. | 2 | 0 | 5.72 | 0.0066 | 0.23 |
| *NOS3* | rs199688227 | Mis | Med. | p.Arg40Gln | 0.0014 | 18 | 8 | 3.05 | 0.0068 | 0.23 |
| *HNF1A* | rs142318174 | Mis | Med. | p.Gly52Ala | 0.0034 | 20 | 30 | 0.416 | 0.01 | 0.2 |
| *IRS2* | var_13_110434847 | Mis | Med. | p.Ser1185Asn | 0.0016 | 7 | 9 | 0.218 | 0.011 | 0.19 |
| *MADD* | rs35233100 | Stop | High | p.Arg766Ter | 0.053 | 1026 | 1310 | 0.895 | 0.011 | 0.19 |
| *AKT2* | rs184042322 | Mis | Med. | p.Pro50Thr | 0.0021 | 39 | 23 | 1.73 | 0.012 | 0.18 |
| *PPP1R3A* | var_7_113558889 | Mis | Med. | p.Asp55Asn | 0.003 | 2 | 9 | 0.356 | 0.012 | 0.18 |
| *IRS1* | rs201042474 | Mis | Med. | p.His834Tyr | 0.0005 | 1 | 5 | 0.355 | 0.014 | 0.16 |
| *AR* | var_X_66765173 | Mis | Med. | p.Gln62Leu | 0.34 | 1454 | 1473 | 0.737 | 0.015 | 0.16 |
| *HNF1A* | rs1169288 | Mis | Med. | p.Ile27Leu | 0.43 | 16563 | 16278 | 1.04 | 0.016 | 0.15 |
| *MADD* | rs377474784 | Mis | Med. | p.Asn444Ser | 0.00012 | 4 | 0 | 5.27 | 0.017 | 0.15 |
| *PPP1R3A* | var_7_113558630 | Mis | Med. | p.Ser141Asn | 0.0011 | 2 | 11 | 0.33 | 0.019 | 0.14 |
| *CYB5R4* | rs201872238 | Mis | Med. | p.Asp285Val | 0.00017 | 0 | 5 | 0.153 | 0.02 | 0.14 |
| *NOS3* | var_7_150698367 | Mis | Med. | p.Met428Val | 0.00075 | 8 | 1 | 3.43 | 0.021 | 0.14 |
| *MADD* | var_11_47298308 | Mis | Med. | p.Asn330Ser | 0.00019 | 7 | 1 | 2.38 | 0.022 | 0.14 |
| *MADD* | var_11_47295511 | Mis | Med. | p.Val16Leu | 0.003 | 17 | 3 | 2.51 | 0.023 | 0.14 |
| *MADD* | var_11_47346102 | Mis | Med. | p.Ile1566Val | 0.00039 | 13 | 2 | 2.39 | 0.025 | 0.14 |
| *SLC2A4* | var_17_7187827 | Mis | Med. | p.Ala251Ser | 0.00058 | 0 | 7 | 0.186 | 0.025 | 0.14 |
| *MADD* | var_11_47296554 | Mis | Med. | p.Arg168His | 0.00013 | 0 | 5 | 0.243 | 0.028 | 0.13 |
| *FOXM1* | rs201000406 | Mis | Med. | p.Glu161Lys | 6.0e-05 | 2 | 0 | 5.8 | 0.031 | 0.13 |
| *HNF1A* | var_12_121434124 | Mis | Med. | p.Gly339Ser | 0.003 | 26 | 11 | 1.69 | 0.032 | 0.12 |

# 3 Supplementary Figures

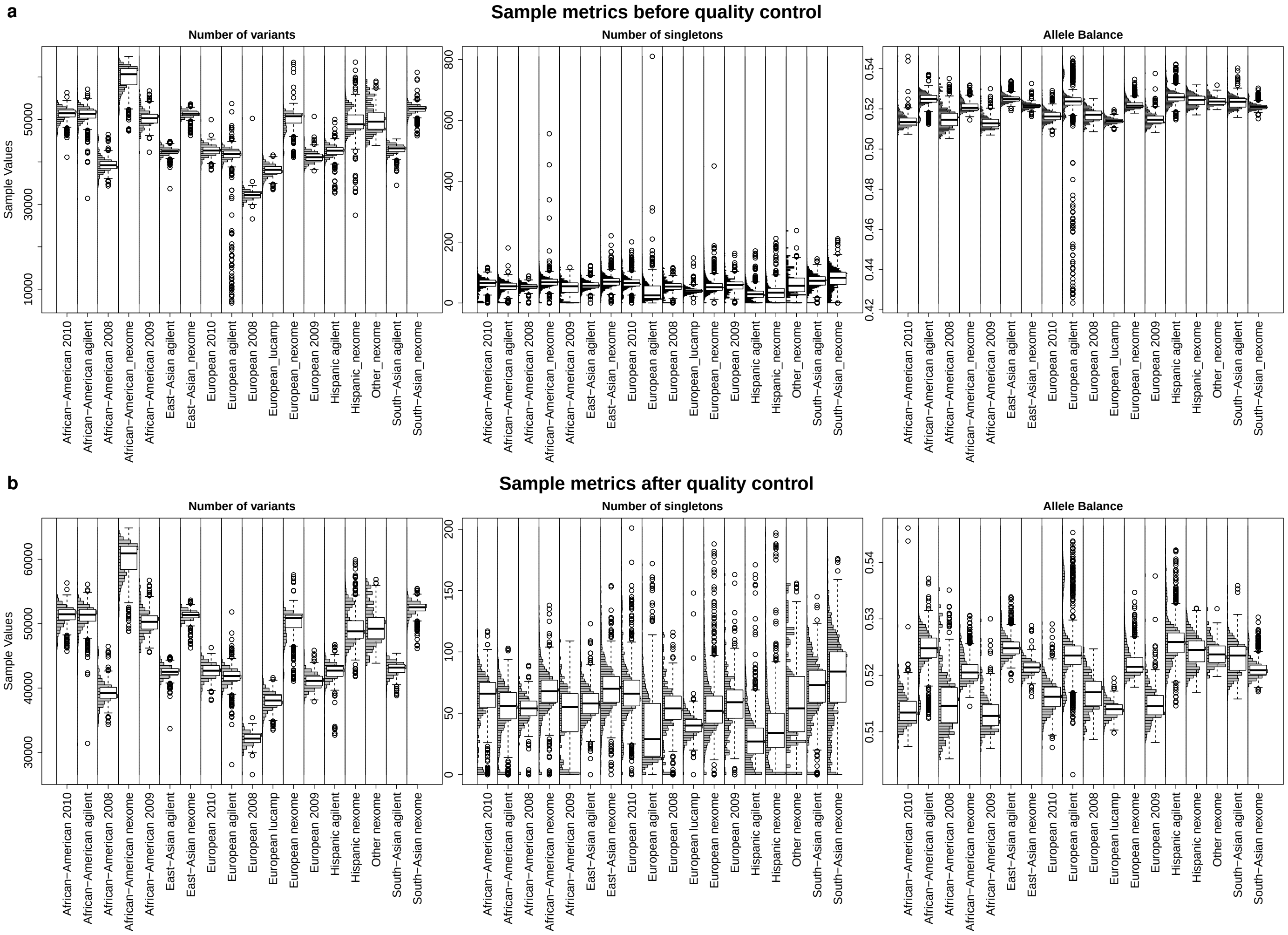


**Supplementary Figure 1**: **Sample quality control metrics.** To perform sample quality control, we computed a series of metrics that informed on the sequencing quality of a sample. We then stratified samples (N=45,231) by ancestry and sequencing technology (i.e. capture technology and year of sequencing), plotted the distribution of metrics for each stratum of samples, and used these plots to visually identify outlier samples for removal by quality control. Shown are (left to right) distributions of the number of variant alleles carried by each sample, the number of variant alleles unique to a sample carried by each sample, and the average fraction of sequence reads supporting a non-reference allele at heterozygous sites within each sample. Distributions are shown for **(a)** all samples from the “Raw” dataset and **(b)** all samples from the “Clean” dataset. Sample strata are labeled by a combination of ancestry and (internal names for) sequencing technology.

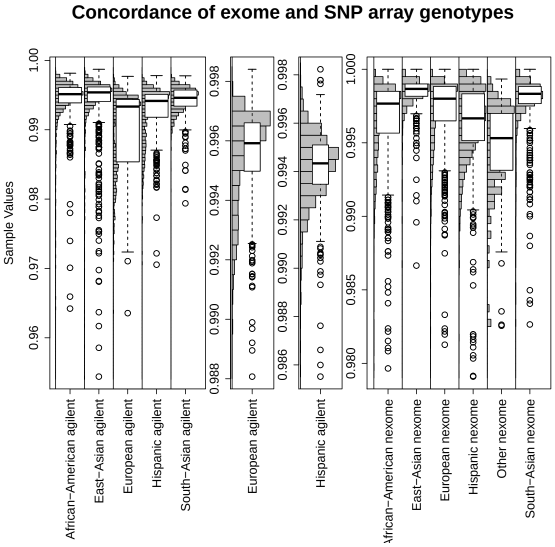


**Supplementary Figure 2**: **Concordance of exome sequence and SNP array genotypes.** We measured concordance between genotypes called non-reference from sequence data and genotypes called at the same sites in the same samples from SNP array data (N=34,529). Samples are stratified via the same manner as in **Supplementary Figure 1**; the y-axis plots the fraction of non-reference genotypes with an identical genotype call in the corresponding SNP array data. We used four different groups of SNP array data in the analysis (**Methods**), resulting in different y-axis scales for different SNP arrays. Hispanic refers to individuals of either Hispanic or Latino ancestry.

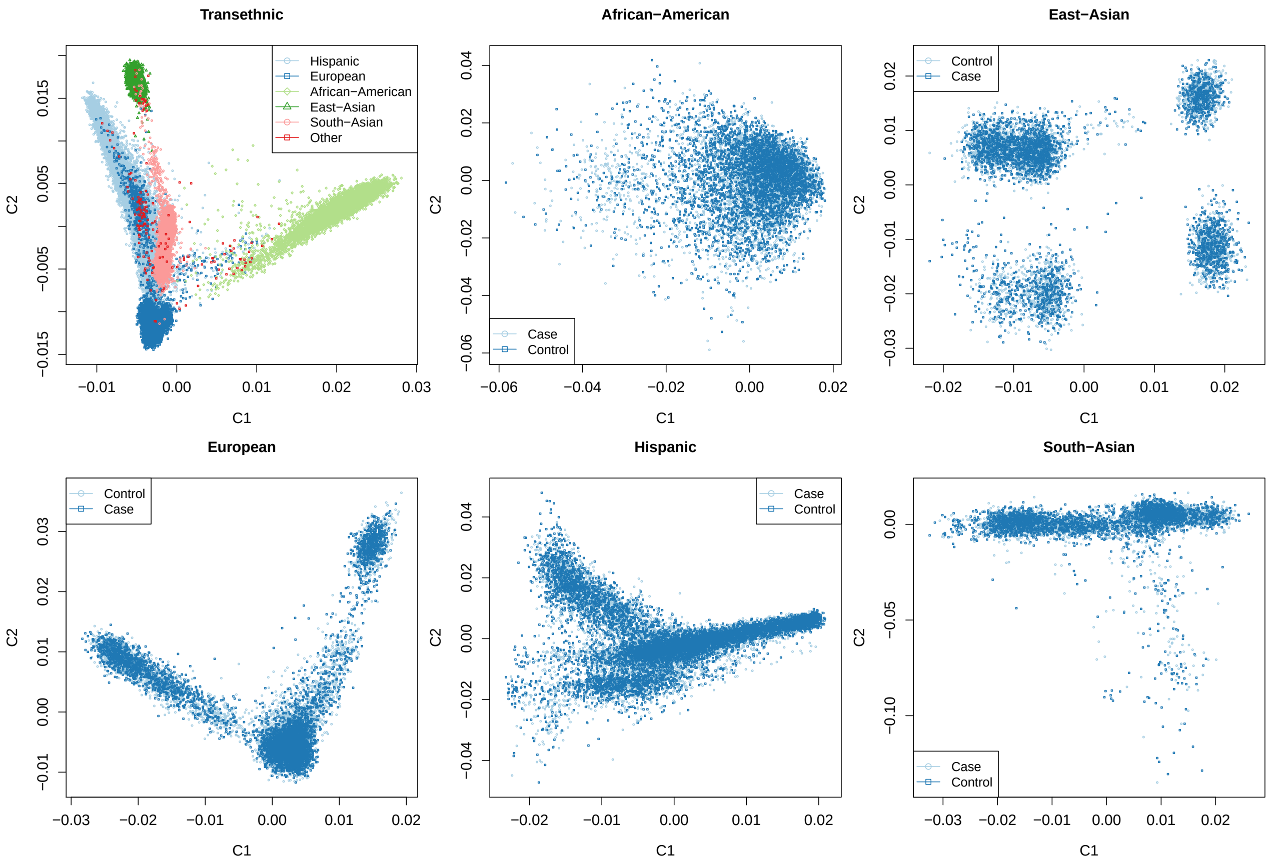


**Supplementary Figure 3**: **Principal component analysis.** We computed principal component analysis (PCA) based on an LD-pruned collection of variants from exome sequence data (N=45,231 individuals). We computed a PCA across all samples (Transethnic; samples colored by reported ancestry) using SNPs common (MAF>1%) in each ancestry, as well as additional PCAs specific to samples from each ancestry (Ancestry labeled plots; samples colored by case/control status for T2D) using a broader set of SNPs common (MAF>1%) in the relevant ancestry.

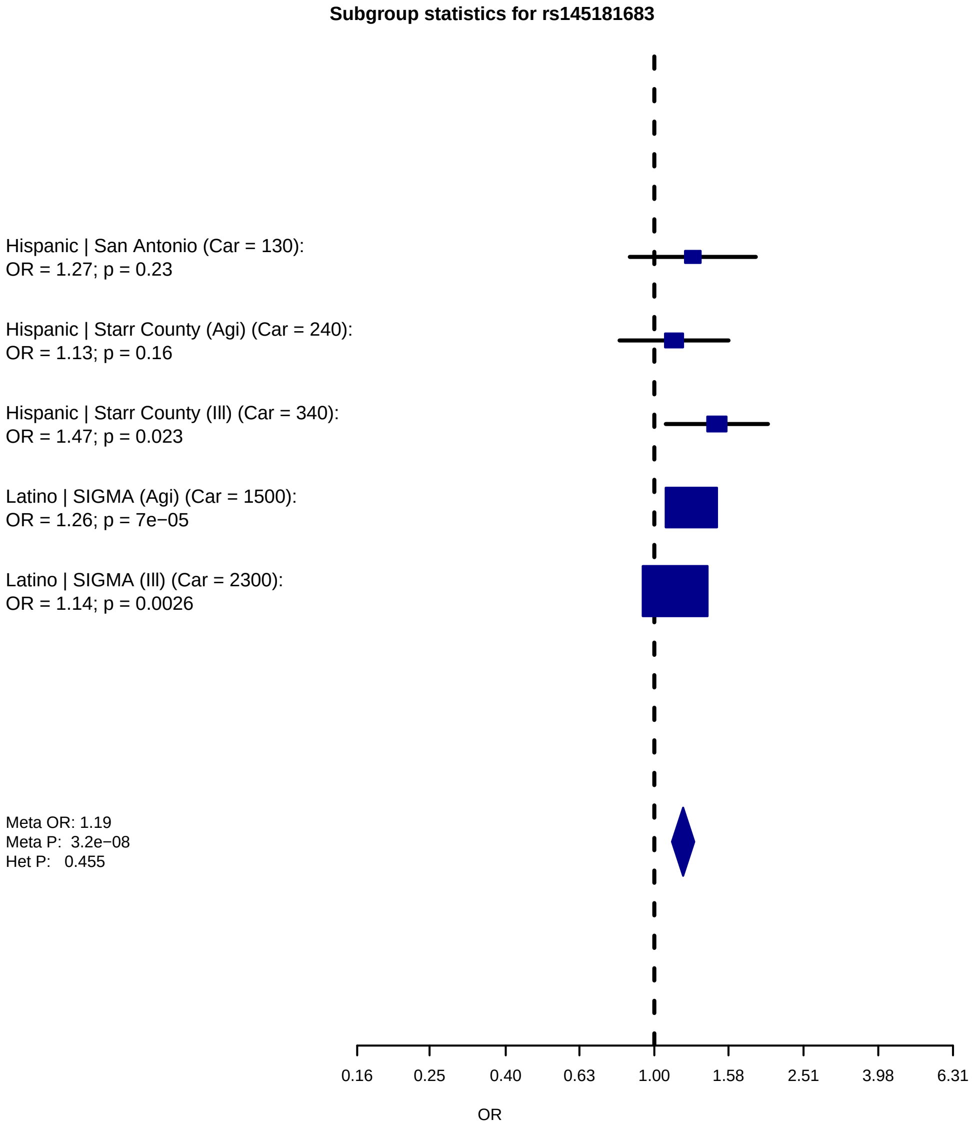


**Supplementary Figure 4**: ***SFI1* subgroup-level associations.** Shown are the *p*-values and odds-ratio estimates for each sample subgroup with at least 10 carriers of the rs145181683 variant in *SFI1* (N=14,469 across all subgroups). Blue boxes indicate odds ratios (sized proportionately to the number of carriers in the subgroup) and black bars indicate standard errors. Car: number of variant carriers. OR: odds-ratio for each subgroup as calculated by the Firth test. *p*: *p*-value for each subgroup as calculated by the (two-sided) EMMAX analysis. Meta: results from the (two-sided) inverse-variance meta-analysis across all 25 sample subgroups (including those not shown in this figure). Het P: *p*-value of one-sided $\chi^2$ test for heterogeneity in odds ratios across sample subgroups.

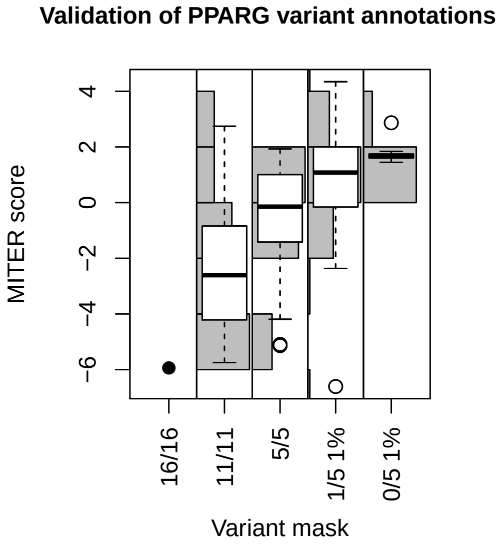


**Supplementary Figure 5**: **Validation of allele deleteriousness within variant masks.** To assess whether the severity ordering of masks in our gene-level analysis correspond to an increasing likelihood that alleles in the mask are deleterious, we used previously published data [67] assessing the extent to which missense variants in the gene *PPARG* impede adipocyte differentiation. For the five masks containing at least one *PPARG* allele, shown are box plots or strip charts of allelic MITER scores (a measure of predicted *PPARG* loss of function, with lower scores suggesting lower function). 11/11 (N=9 variants): min=-5.75, 25%=-4.2, median=-2.6, 75%=-0.84, max=2.7; 5/5 (N=18 variants): min=-5.14, 25%=-1.42, median=-0.15, 75%=1.01, max=1.93; 1/5 1% (N=54 variants): min=-6.61, 25%=-0.16 , median=1.08, 75%=2.00, max=4.34; 0/5 1% (N=7 variants): min=1.45, 25%=1.61, median=1.66, 75%=1.75, max=2.87.

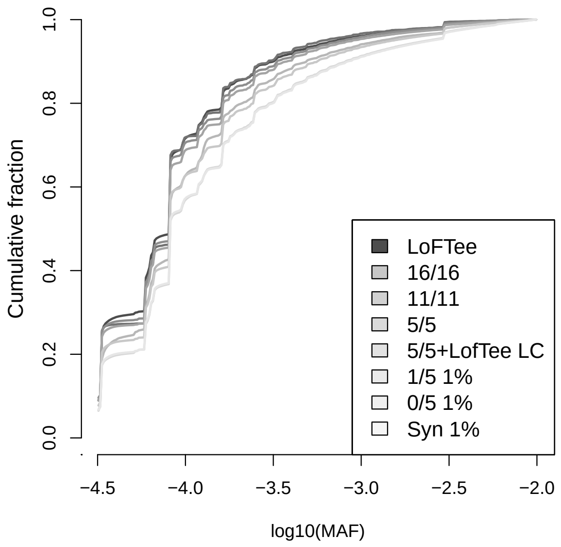


**Supplementary Figure 6**: **Weight estimation for masks.** For each variant mask, we estimated allelic weights corresponding to the fraction of loss-of-function alleles in the mask, under a previously presented [21] model whereby a set of missense alleles is a mixture of fully loss-of-function or fully benign alleles. We estimated this fraction by maximizing the likelihood of the allele frequency distribution, with the LofTee mask used as a reference for loss-of-function alleles and the set of synonymous alleles with frequency below 1% used as a reference for benign alleles. Shown are the cumulative frequency distributions for alleles "unique" to each mask (i.e. absent from all more stringent masks).

**a**

| Mask | Total | | | | | Weighted | | Unique | | | | |
|---|---|---|---|---|---|---|---|---|---|---|---|---|
| | # Var | CAF | OR | Burden | SKAT | OR | Burden | # Var | CAF | OR | Burden | SKAT |
| LoFTee | 4 | 0.00032 | 1.7 | 0.32 | 0.77 | 1.7 | 0.32 | 4 | 0.00032 | 1.7 | 0.32 | 0.77 |
| 16/16 | 4 | 0.00032 | 1.7 | 0.32 | 0.77 | 1.7 | 0.32 | 0 | - | - | - | - |
| 11/11 | 5 | 0.00037 | 1.8 | 0.26 | 0.82 | 1.8 | 0.27 | 1 | 4.6e-05 | 2.2 | 0.55 | 0.6 |
| 5/5 | 40 | 0.0079 | 2 | 1.6e-10 | 5.4e-08 | 2.6 | 2e-10 | 35 | 0.0075 | 2.1 | 2.9e-10 | 5.8e-08 |
| 5/5+LofTee LC 1% | 41 | 0.008 | 2.1 | 8.5e-11 | 4.9e-08 | 2.6 | 1.3e-10 | 1 | 4.6e-05 | 5.1 | 0.22 | 0.15 |
| 1/5 1% | 94 | 0.016 | 1.5 | 2.7e-06 | 2.4e-08 | 2.2 | 6e-09 | 53 | 0.0077 | 1 | 0.97 | 0.46 |
| 0/5 1% | 105 | 0.017 | 1.4 | 4.2e-06 | 6.2e-08 | 2.2 | 4.8e-09 | 10 | 0.00093 | 1.4 | 0.28 | 0.57 |

**b**

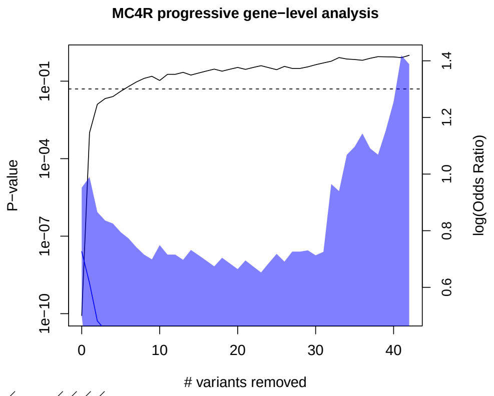


**c**

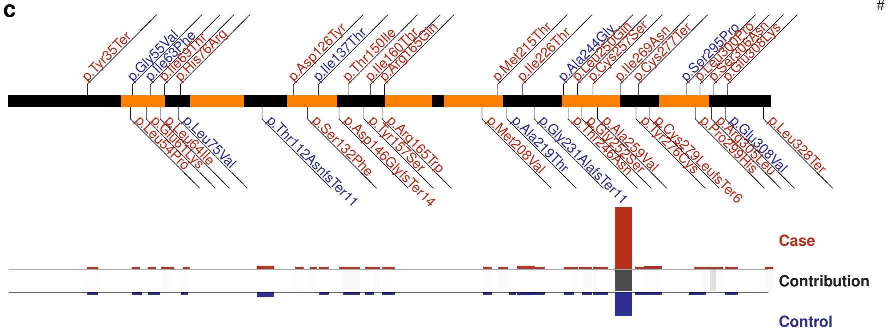


**Supplementary Figure 7**: ***MC4R* gene-level analysis.** Shown is a dissection of the gene-level associations for *MC4R*. All tests are two-sided and N=43,071 unrelated individuals. **(a)** Mask-level statistics for the burden and SKAT tests, as well as the weighted burden test. Each row in the table corresponds to one of the allele masks defined in **Extended Data Item 5**. The first five columns ("Total") show association results for an analysis of all alleles in the mask; the final five columns ("Unique") show association results for analysis of alleles unique to the mask (i.e. not present in more deleterious masks). The "Weighted" columns show association results for a weighted burden test of all alleles in each mask; the weighted burden result used in the main analysis is that in the final row. #Var: the number of variants in the association analysis. CAF: the total combined frequency of all alleles in the analysis. OR: the odds-ratio estimated from the burden (or weighted burden) analysis. Burden: the *p*-value from the burden test. SKAT: the *p*-value from the SKAT analysis. The #Var and CAF columns for the "Total" analysis also apply to the "Weighted" analysis. **(b)** Gene-level association *p*-values for *MC4R*, using the burden test on alleles in the 1/5 1% mask (that achieving greatest statistical significance) after progressive removal of variants ordered by increasing single-variant *p*-value. The left-axis (black line) shows the observed $-\log_{10}(p)$ value, with dashed line indicating nominal significance of $p<0.05$. The right-axis (blue line) shows the estimated effect size ($log(OR)$), with shaded blue indicating the 95% confidence interval and dotted line indicating effect size=0. **(c)** A graphical plot of variants observed in *MC4R* within the 1/5 1% mask. Variants are colored blue (if individual OR < 1) or red (OR > 1). Case (red) and control (blue) frequencies are shown below for each variant, with black boxes shaded according to the contribution of each variant to the gene-level signal (computed as the difference in $\log_{10}(p)$ observed after removal of the variant from the test). The transmembrane domains of *MC4R* are shaded orange. OR: odds ratio

a

| Mask | Total | | | | | Weighted | | Unique | | | | |
|---|---|---|---|---|---|---|---|---|---|---|---|---|
| | # Var | CAF | OR | Burden | SKAT | OR | Burden | # Var | CAF | OR | Burden | SKAT |
| LoFTee | 4 | 9.3e-05 | 0.67 | 0.69 | 0.38 | 0.67 | 0.69 | 4 | 9.3e-05 | 0.67 | 0.69 | 0.38 |
| 16/16 | 4 | 9.3e-05 | 0.67 | 0.69 | 0.38 | 0.67 | 0.69 | 0 | - | - | - | - |
| 11/11 | 12 | 0.00042 | 1.3 | 0.58 | 0.15 | 1.3 | 0.62 | 8 | 0.00032 | 1.6 | 0.39 | 0.15 |
| 5/5 | 70 | 0.049 | 1.3 | 9e-09 | 9.6e-08 | 1.4 | 9.5e-09 | 58 | 0.048 | 1.3 | 1e-08 | 9.1e-08 |
| 5/5+LofTee LC 1% | 73 | 0.049 | 1.3 | 8.5e-09 | 9.9e-08 | 1.4 | 7.5e-09 | 3 | 0.00019 | 1.3 | 0.72 | 0.7 |
| 1/5 1% | 193 | 0.06 | 1.3 | 6.7e-09 | 6.5e-08 | 1.4 | 1.8e-09 | 120 | 0.011 | 1.1 | 0.17 | 0.14 |
| 0/5 1% | 213 | 0.063 | 1.3 | 7.6e-09 | 1.3e-07 | 1.4 | 2.2e-09 | 19 | 0.0024 | 0.99 | 0.95 | 0.75 |

b

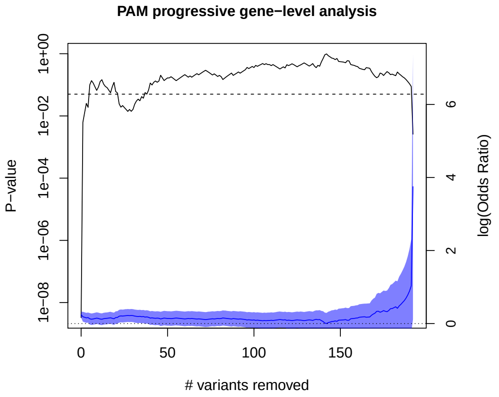


**Supplementary Figure 8**: ***PAM* gene-level analysis.** Shown is a dissection of the gene-level associations for *PAM*. All tests are two-sided and N=43,071 unrelated individuals. Panels are analogous to those in **Supplementary Figure 7**. A graphical plot of variants is not shown due to the large number of variants in *PAM*.

a

| Mask | Total | | | | | Weighted | | Unique | | | | |
|---|---|---|---|---|---|---|---|---|---|---|---|---|
| | # Var | CAF | OR | Burden | SKAT | OR | Burden | # Var | CAF | OR | Burden | SKAT |
| LoFTee | 13 | 0.0012 | 0.46 | 0.0072 | 0.032 | 0.46 | 0.0072 | 13 | 0.0012 | 0.46 | 0.0072 | 0.032 |
| 16/16 | 13 | 0.0012 | 0.46 | 0.0072 | 0.032 | 0.46 | 0.0072 | 0 | - | - | - | - |
| 11/11 | 15 | 0.0013 | 0.5 | 0.013 | 0.041 | 0.49 | 0.011 | 2 | 0.00014 | 1.1 | 0.87 | 0.93 |
| 5/5 | 37 | 0.0045 | 0.47 | 1.4e-06 | 0.00072 | 0.4 | 1.8e-06 | 22 | 0.0032 | 0.46 | 3.5e-05 | 0.0011 |
| 5/5+LofTee LC 1% | 37 | 0.0045 | 0.47 | 1.4e-06 | 0.00072 | 0.4 | 1.8e-06 | 0 | - | - | - | - |
| 1/5 1% | 86 | 0.012 | 0.6 | 4.7e-08 | 3.6e-05 | 0.39 | 1.1e-08 | 49 | 0.0072 | 0.69 | 0.0015 | 0.0015 |
| 0/5 1% | 103 | 0.014 | 0.62 | 5.5e-08 | 2.8e-05 | 0.4 | 1.3e-08 | 17 | 0.0019 | 0.8 | 0.34 | 0.12 |

b

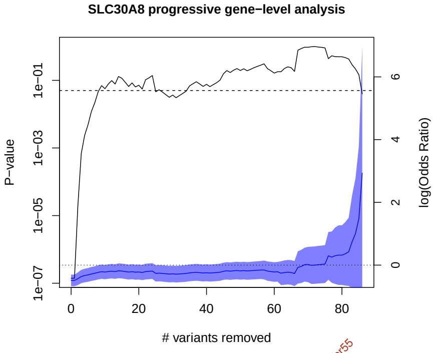


c

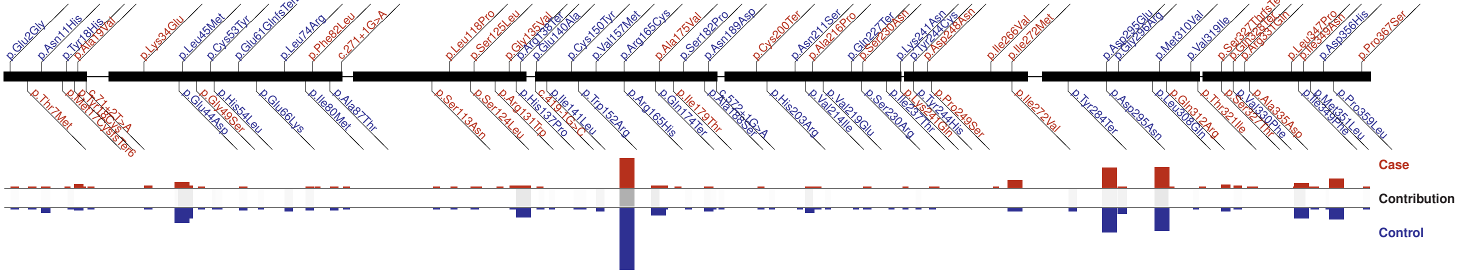


**Supplementary Figure 9**: ***SLC30A8* gene-level analysis.** Shown is a dissection of the gene-level associations for *SLC30A8*. All tests are two-sided and N=43,071 unrelated individuals. Panels are analogous to those in **Supplementary Figure 7**.

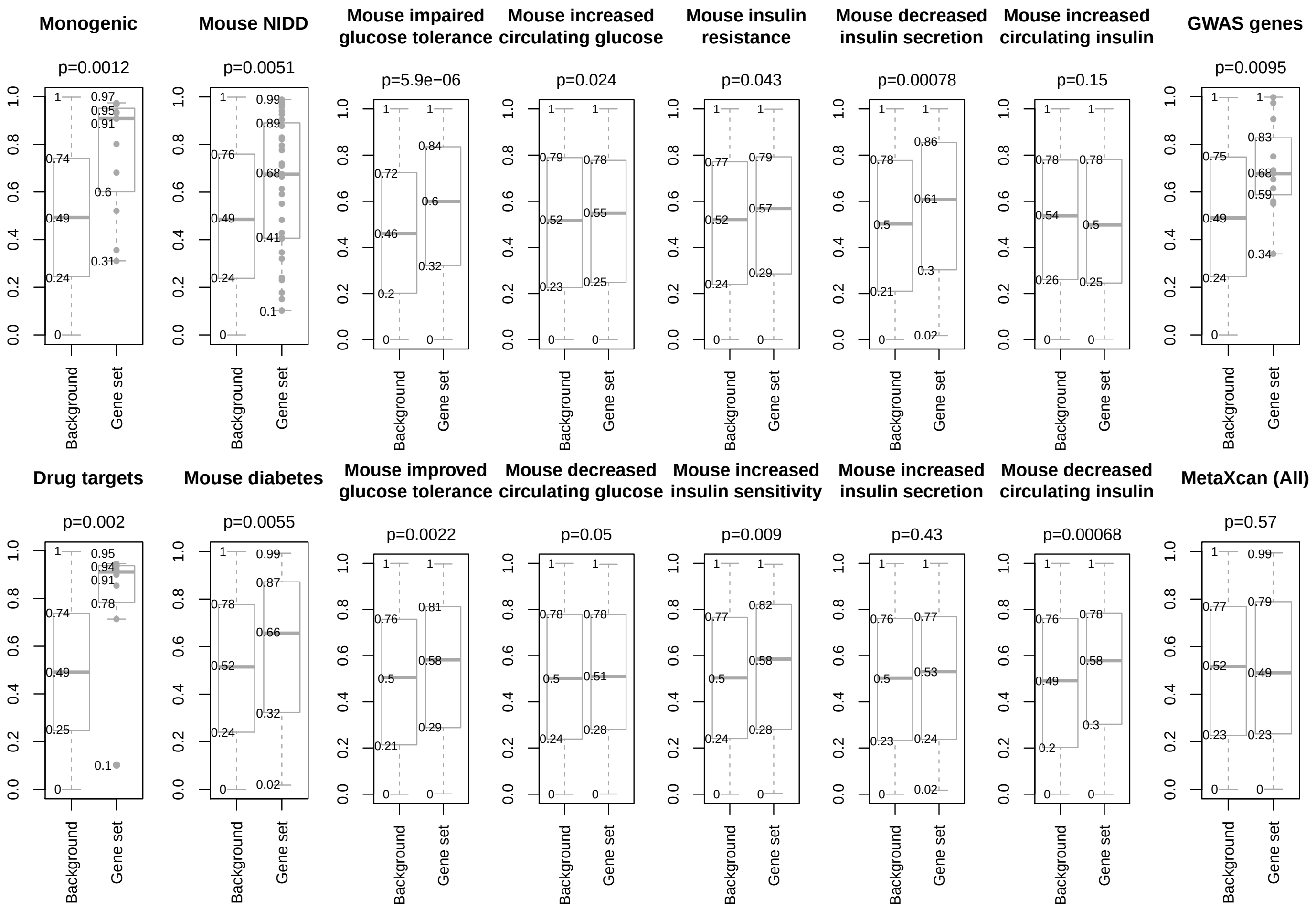


**Supplementary Figure 10**: **Full results from exome sequence gene set analysis.** For various sets of genes implicated as relevant to T2D based on knockout mouse phenotypes, we used a one-side Wilcoxon rank-sum test to compare gene-level association statistics to those of matched comparison genes (**Methods**). Shown are box plots of the distributions of rank percentiles (1 being the highest) for each gene set analyzed; plots are analogous to those in **Figure 2**. Labels indicate the minimum, 25th percentile, median, 75th percentile, and maximum values. Only genes with variation in the exome sequence dataset were included in the analysis. Monogenic: 11 genes (comparison 548 genes). Drug targets: 8 genes (comparison 400 genes). GWAS genes: 11 genes (comparison 537 genes). MetaXcan (All): 19 genes (comparison 938 genes). Mouse NIDD: 31 genes (comparison 1,499 genes). Mouse impaired glucose tolerance: 323 genes (comparison 10,043 genes). Mouse increased circulating glucose: 360 genes (comparison 11,298 genes). Mouse insulin resistance: 179 genes (comparison 7,011 genes). Mouse decreased insulin secretion: 132 genes (comparison 5,364 genes). Mouse increased circulating insulin: 214 genes (comparison 7,800 genes). Mouse diabetes: 72 genes (comparison 3,265 genes). Mouse improved glucose tolerance: 238 genes (comparison 8,492 genes). Mouse decreased circulating glucose: 477 genes (comparison 12,718 genes). Mouse increased insulin sensitivity: 176 genes (comparison 6,819 genes). Mouse increased insulin secretion: 51 genes (comparison 2,395 genes). Mouse decreased circulating insulin: 318 genes (comparison 10,503 genes).

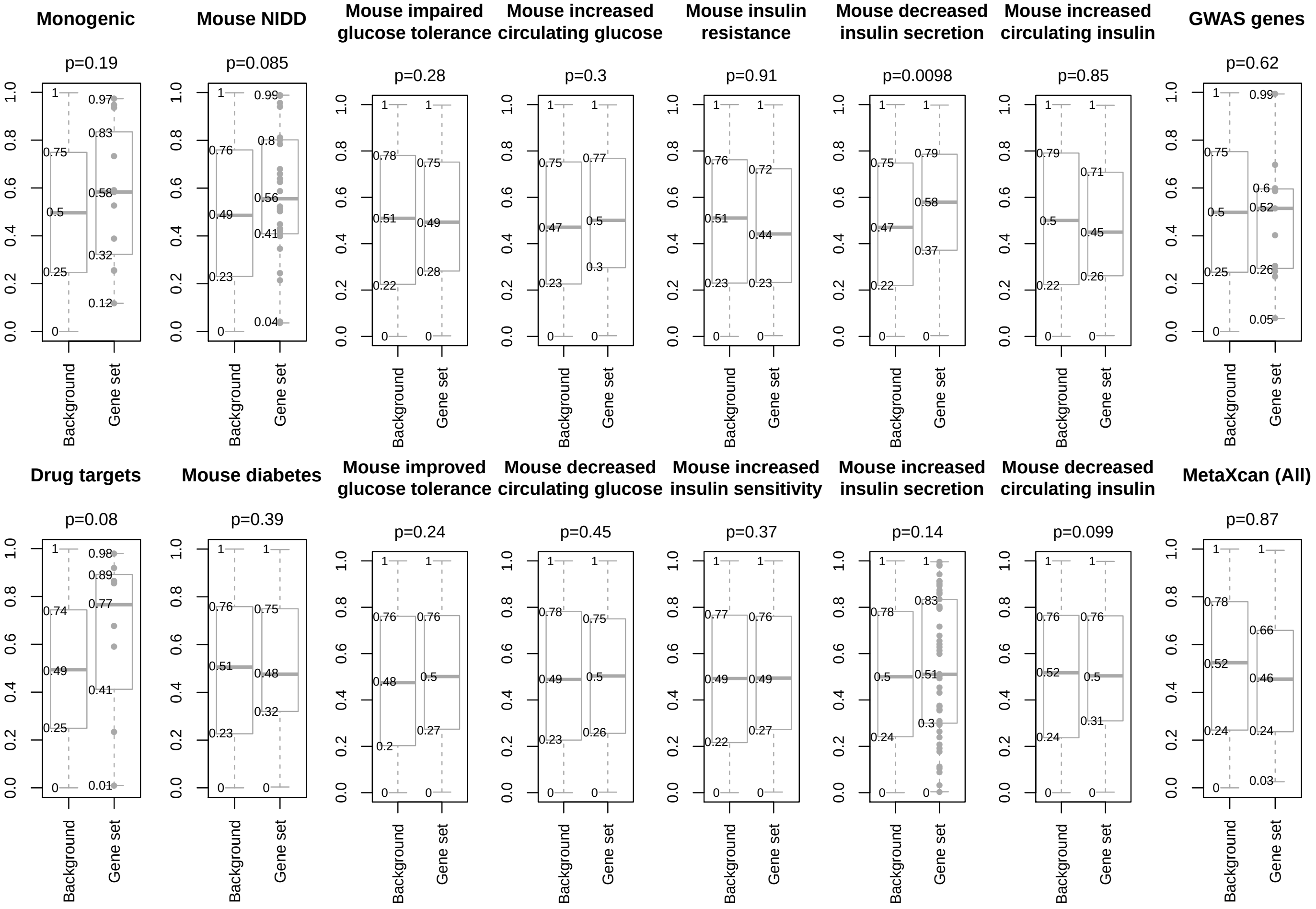


**Supplementary Figure 11**: **Gene set analysis from protein-truncating variants.** To assess the value of nonsynonymous variants in exome sequence gene set analysis, we conducted a similar rank-sum comparison of gene sets as that described in the main text – only using the burden test of protein truncating variants (PTVs, those included in the LofTee mask), rather than the minimum *p*-value burden test, to calculate gene-level associations. Shown are plots, analogous to those in **Supplementary Figure 10**, summarizing these PTV-based comparisons. *P*-values correspond to a one-sided Wilcoxon Rank-Sum test comparing the associations to those of matched comparison genes. Labels indicate the minimum, 25th percentile, median, 75th percentile, and maximum values. Only genes with PTVs in our dataset (a smaller number of genes than in **Supplementary Table 10**) were included in the analysis. Monogenic: 11 genes (comparison 546 genes). Drug targets: 8 genes (comparison 400 genes). GWAS genes: 11 genes (comparison 535 genes). MetaXcan (All): 18 genes (comparison 888 genes). Mouse NIDD: 26 genes (comparison 1,247 genes). Mouse impaired glucose tolerance: 289 genes (comparison 9,033 genes). Mouse increased circulating glucose: 319 genes (comparison 10,008 genes). Mouse insulin resistance: 157 genes (comparison 6,200 genes). Mouse decreased insulin secretion: 110 genes (comparison 4,580 genes). Mouse increased circulating insulin: 183 genes (comparison 6,824 genes). Mouse diabetes: 61 genes (comparison 2,809 genes). Mouse improved glucose tolerance: 210 genes (comparison 7,554 genes). Mouse decreased circulating glucose: 424 genes (comparison 11,228 genes). Mouse increased insulin sensitivity: 150 genes (comparison 5,934 genes). Mouse increased insulin secretion: 46 genes (comparison 2,184 genes). Mouse decreased circulating insulin: 282 genes (comparison 9,353 genes).

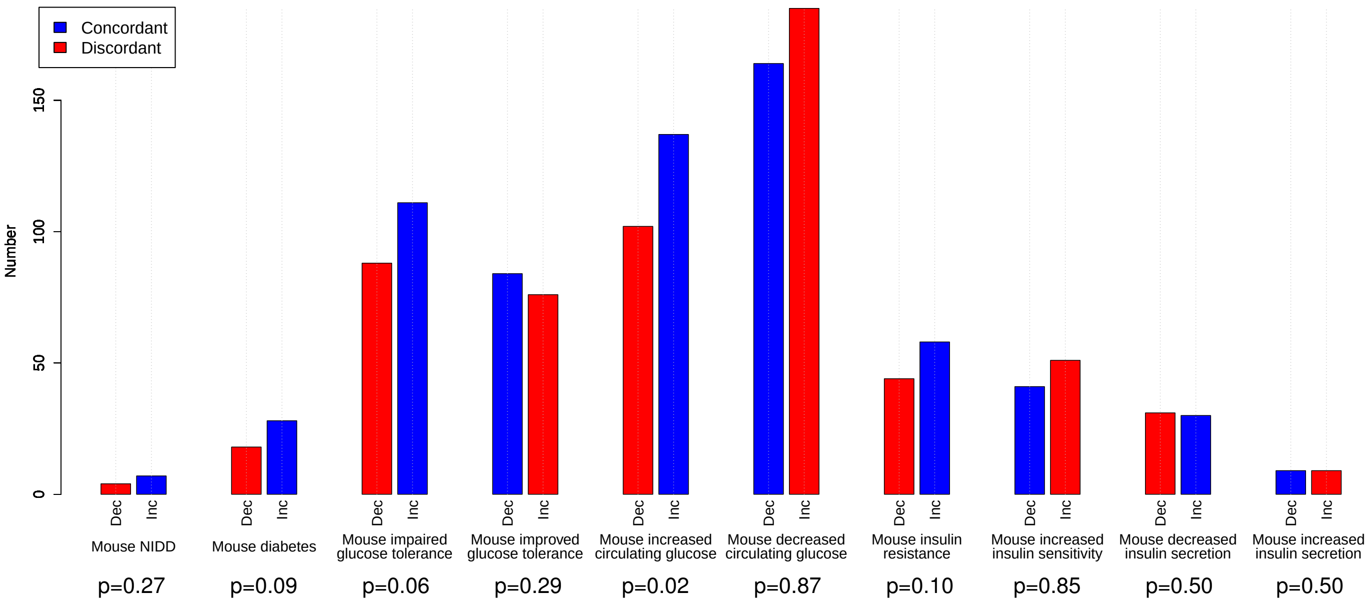


**Supplementary Figure 12**: **Directional consistency of genetic odds ratio estimates and knockout mouse phenotypes.** For each gene set associated with a knockout mouse phenotype for which there was a analogous human phenotype of increased or decreased T2D risk (**Methods**), we calculated the fraction of genes for which the odds-ratio (OR) estimated from the weighted burden test (N=43,071 unrelated individuals) had a direction consistent with what would be predicted from the knockout mouse phenotype. Blue bars correspond to the number of genes with OR estimates concordant with that predicted from the mouse phenotype, while red bars correspond to the number with discordant OR estimates. *p*-values shown below the bars are calculated from a one-sided binomial test of the null hypothesis that $<$ 50% of estimates are concordant. Dec: OR estimate is $<$1. Inc: OR estimate is $>$1.

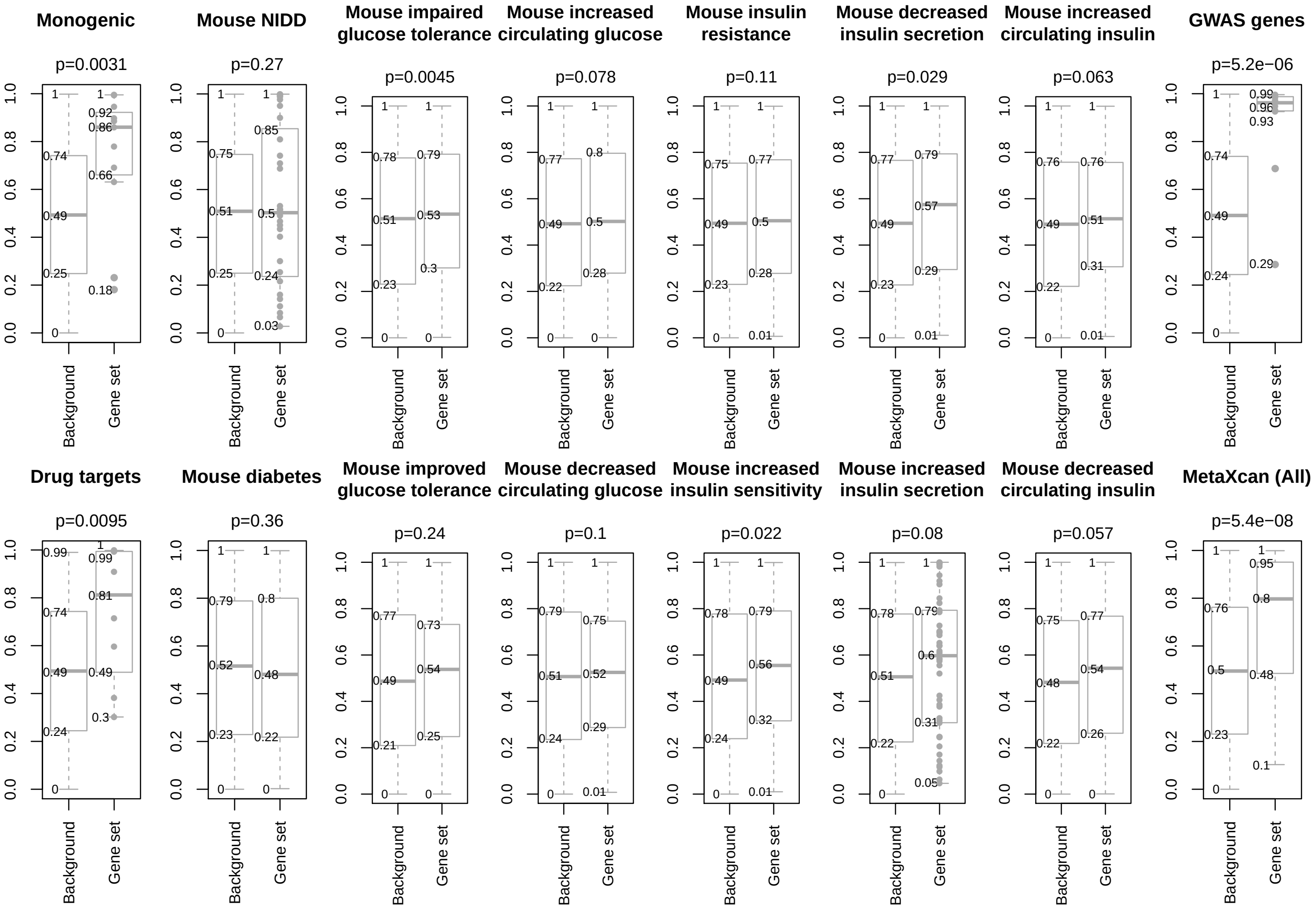


**Supplementary Figure 13**: **Gene set analysis from imputed GWAS statistics.** To assess how similarly array-based GWAS association statistics could identify gene set associations, as compared to exome sequence gene-level association statistics, we conducted a similar rank-sum comparison of gene sets as that described in the main text – only using gene MAGENTA [55] scores from the imputed GWAS rather than the minimum *p*-value burden test to calculate ranks. Shown are plots, analogous to those in **Supplementary Figure 10**, summarizing these GWAS-based comparisons. *P*-values correspond to a one-sided Wilcoxon Rank-Sum test comparing the associations to those of matched comparison genes. Labels indicate the minimum, 25th percentile, median, 75th percentile, and maximum values. Genes on the X chromosome were not analyzed, and only genes with MAGENTA scores were included in the analysis. Monogenic: 11 genes (comparison 547 genes). Drug targets: 8 genes (comparison 399 genes). GWAS genes: 11 genes (comparison 538 genes). MetaXcan (All): 17 genes (comparison 837 genes). Mouse NIDD: 28 genes (comparison 1,350 genes). Mouse impaired glucose tolerance: 304 genes (comparison 9,047 genes). Mouse increased circulating glucose: 329 genes (comparison 10,105 genes). Mouse insulin resistance: 169 genes (comparison 6,461 genes). Mouse decreased insulin secretion: 124 genes (comparison 4,948 genes). Mouse increased circulating insulin: 196 genes (comparison 7,038 genes). Mouse diabetes: 67 genes (comparison 2,975 genes). Mouse improved glucose tolerance: 225 genes (comparison 7,676 genes). Mouse decreased circulating glucose: 436 genes (comparison 11,194 genes). Mouse increased insulin sensitivity: 169 genes (comparison 6,317 genes). Mouse increased insulin secretion: 46 genes (comparison 2,135 genes). Mouse decreased circulating insulin: 300 genes (comparison 9,444 genes) genes).

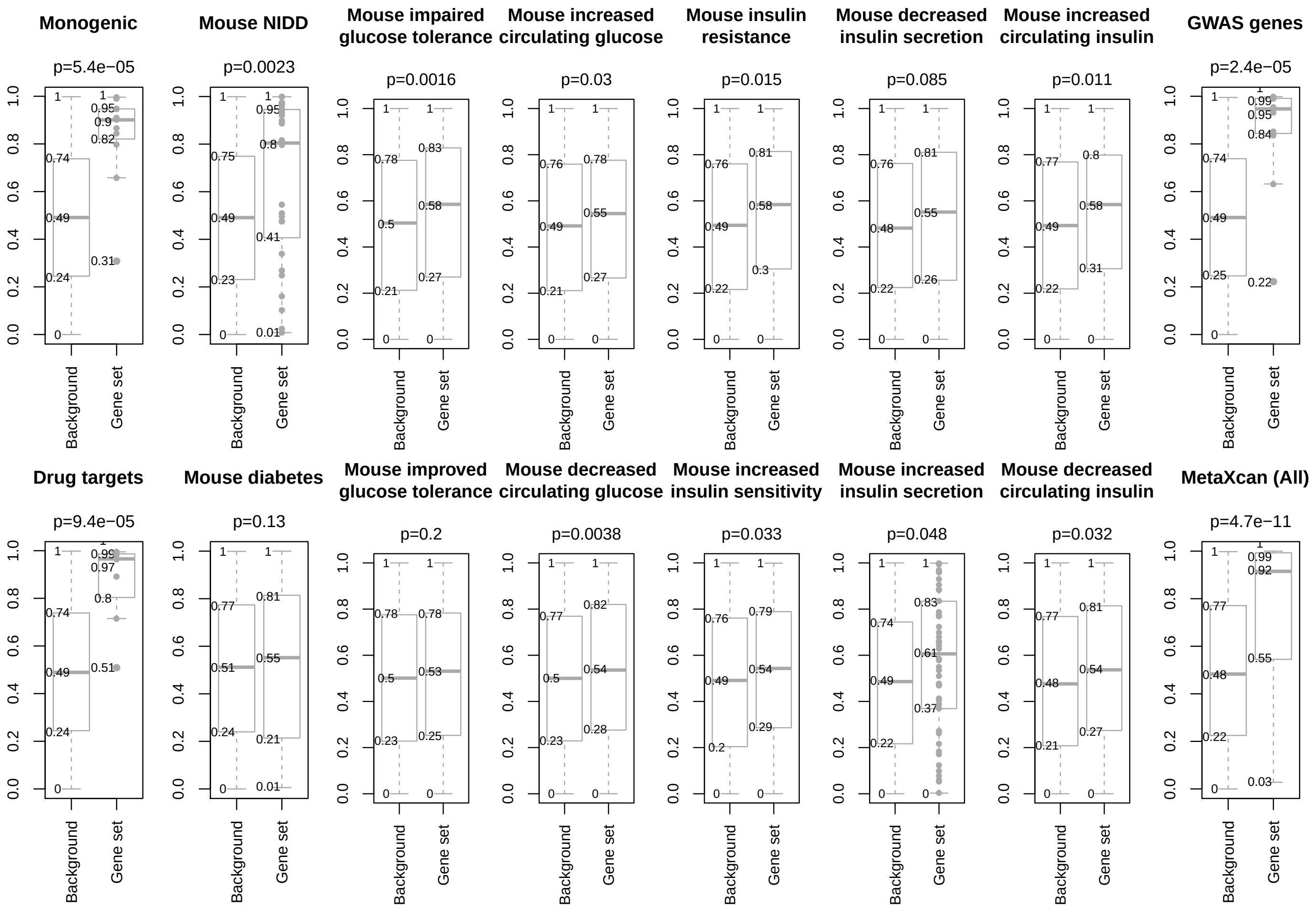


**Supplementary Figure 14**: **Gene set analysis from a larger array-based GWAS.** To assess whether the ability of GWAS statistics to prioritize genes was driven by sample size, rather than fundamental limitations of SNP arrays and imputation, we repeated our rank-sum analysis using gene MAGENTA [55] scores but from a large transethnic T2D GWAS [45] rather than the imputed GWAS in our study. Shown are plots, analogous to those in **Supplementary Figure 13**, summarizing these comparisons. *P*-values correspond to a one-sided Wilcoxon Rank-Sum test comparing the associations to those of matched comparison genes. Labels indicate the minimum, 25th percentile, median, 75th percentile, and maximum values. Genes on the X chromosome were not analyzed, and only genes with MAGENTA scores were included in the analysis. Monogenic: 11 genes (comparison 547 genes). Drug targets: 8 genes (comparison 399 genes). GWAS genes: 11 genes (comparison 538 genes). MetaXcan (All): 17 genes (comparison 837 genes). Mouse NIDD: 28 genes (comparison 1,350 genes). Mouse impaired glucose tolerance: 304 genes (comparison 9,043 genes). Mouse increased circulating glucose: 329 genes (comparison 10,104 genes). Mouse insulin resistance: 169 genes (comparison 6,458 genes). Mouse decreased insulin secretion: 124 genes (comparison 4,945 genes). Mouse increased circulating insulin: 196 genes (comparison 7,034 genes). Mouse diabetes: 67 genes (comparison 2,973 genes). Mouse improved glucose tolerance: 225 genes (comparison 7,673 genes). Mouse decreased circulating glucose: 436 genes (comparison 11,188 genes). Mouse increased insulin sensitivity: 169 genes (comparison 6,314 genes). Mouse increased insulin secretion: 46 genes (comparison 2,134 genes). Mouse decreased circulating insulin: 300 genes (comparison 9,441 genes).

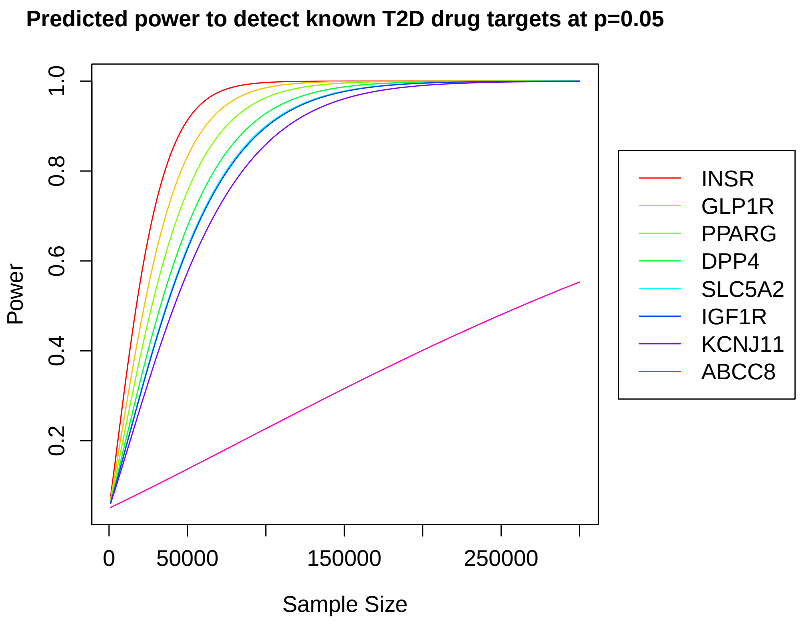


**Supplementary Figure 15**: **Power to exceed nominal significance for T2D drug targets.** Estimated power, as a function of sample size, to detect T2D gene-level associations (at significance $p<0.05$) for genes with genetic effects (aggregate frequency and odds ratios) equal to those estimated for eight established T2D drug targets. Power curves as a function of future sample size (x-axis) are shown and colored separately for each target. This figure is identical to that in **Figure 4a** except with a lower significance threshold used in power calculations.

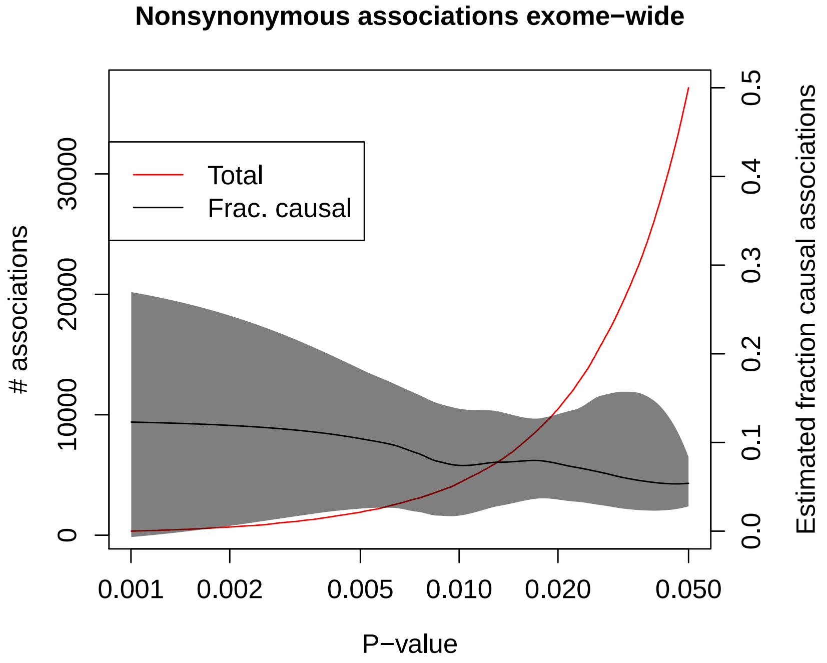


**Supplementary Figure 16**: **Exome-wide posterior estimates.** In addition to estimation of the posterior probability of association (PPA) for nonsynonymous variants within T2D GWAS loci, we also calculated PPA estimates for arbitrary variants exome-wide. Shown are these estimates (black line, gray 95% confidence interval; right axis), as well as the number of total variants (red line; left axis), as a function of single-variant *p*-value observed in our analysis (as calculated by the [two-sided] EMMAX test, N=45,231). This plot is analogous to that in **Extended Data 9b**.

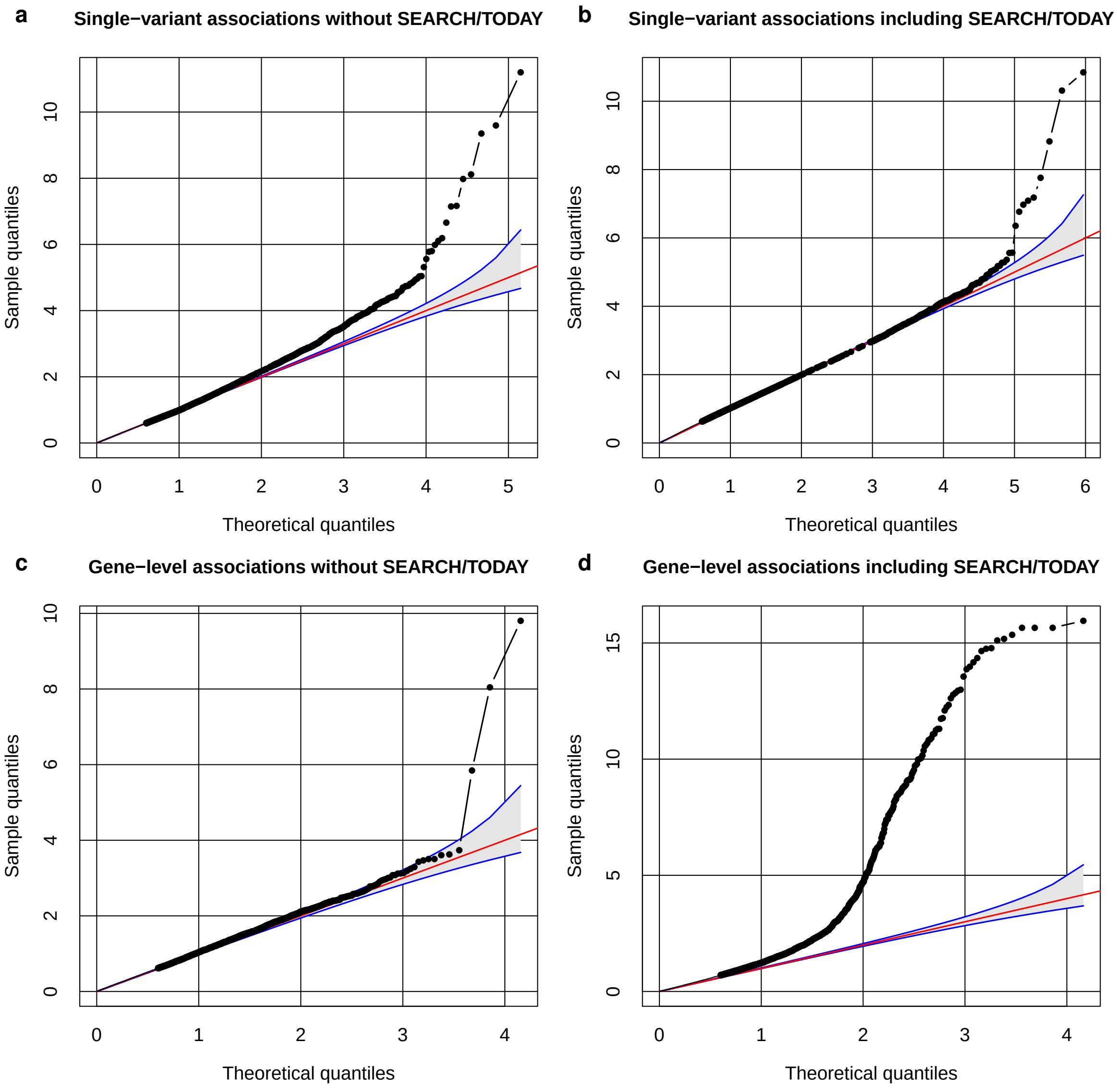


**Supplementary Figure 17**: **Analysis with SEARCH and TODAY samples included.** Among the samples we sequenced were childhood diabetes cases from the SEARCH and TODAY studies (**Supplementary Table 1**). We initially hoped to include these cases in our analysis, but the lack of matched controls within these studies raised concerns about potential association artifacts. To evaluate the inclusion of these cohorts, we compared **(ab)** single-variant and **(cd)** gene-level associations with and without SEARCH and TODAY samples included. **(a)** A QQ plot of single-variant associations computed without SEARCH and TODAY samples (two-sided EMMAX test, N=45,231 individuals). Association statistics are computed via a meta-analysis of ancestry-level (rather than subgroup-level) association statistics, in order to match an analysis with SEARCH and TODAY samples as closely as possible (a subgroup-level meta-analysis is not possible with SEARCH and TODAY due to the absence of controls in those studies). Only variants with minor allele count >15 are shown. **(b)** A QQ plot of single-variant associations with SEARCH and TODAY samples included (two-sided EMMAX test, N=48,741 individuals). **(c)** A QQ plot of (two-sided) gene-level burden associations from the 5/5 mask (N=43,071 unrelated individuals). Only genes with >15 aggregate alternate alleles are shown in the QQ plot. **(d)** A QQ plot of (two-sided) gene-level burden associations from the 5/5 mask with SEARCH and TODAY samples included (N=46,581 unrelated individuals). Red line: expectation of p-values under the null distribution. Blue lines (and gray region): 95% confidence interval of expectations under the null distribution.

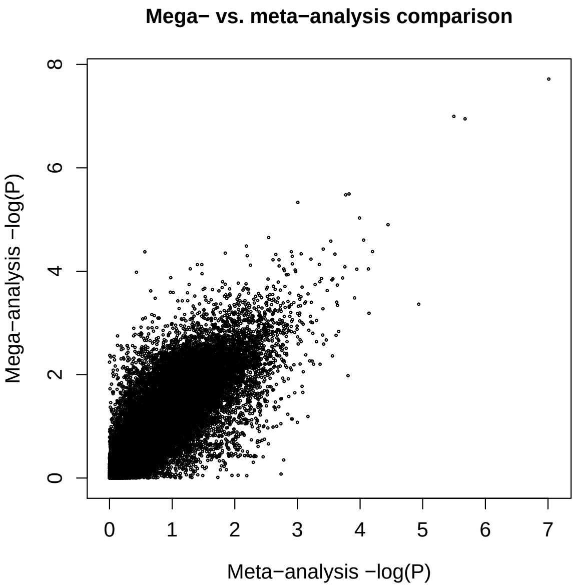


**Supplementary Figure 18**: **Comparison of single-variant mega- and meta-analyses.** To evaluate the extent to which a trans-ancestry mega-analysis (which we applied for gene-level tests) was an appropriate analysis strategy, we compared single-variant mega- and meta-analysis association statistics. We calculated single-variant mega-analysis statistics analogously to gene-level statistics (using a [two-sided] Firth logistic regression test with 10 principal components as covariates). The plot shows a comparison of these statistics (y-axis) to an inverse-variance weighted meta-analysis of subgroup-level (two-sided) Firth association results (x-axis). Only variants included in one of the gene-level masks are plotted. N=43,071 unrelated individuals.

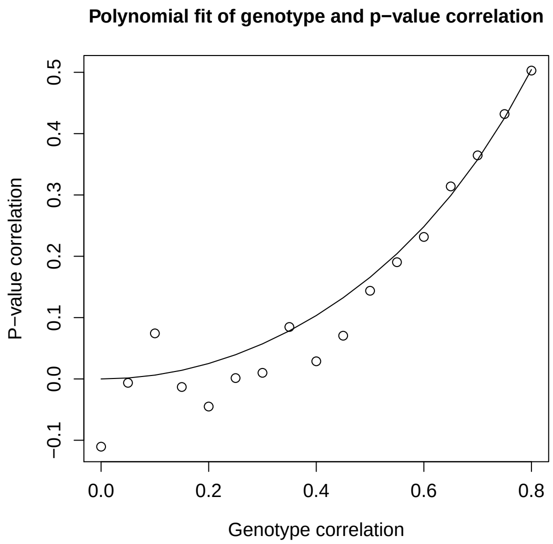


**Supplementary Figure 19**: **Relationship between aggregate genotype correlations and burden test *p*-value correlations.** To evaluate whether a previously reported [24] relationship between single-variant genotype correlations and association p-value correlations also applied to aggregate genotype correlations and burden test *p*-value correlations, we conducted simulations of 1000 gene pairs in 10,000 individuals (500 cases and 500 controls). For each gene pair, we first simulated 50 variants, each of frequency 0.1%, and then calculated a burden score for each individual as the sum of variants carried. We then simulated the burden score of the second gene to have a specified correlation ($\rho$) with the first burden score, by adding random (discrete) noise to the first burden score. We then conducted burden tests between the simulated genotypes and phenotypes of each gene to obtain a pair of simulated *p*-values. We finally binned simulations by actual $\rho$ and calculated *p*-value correlations within each bin. The x-axis of the plot shows the $\rho$ bins, and the y-axis shows the empirically observed correlations among burden score *p*-values within the bin.

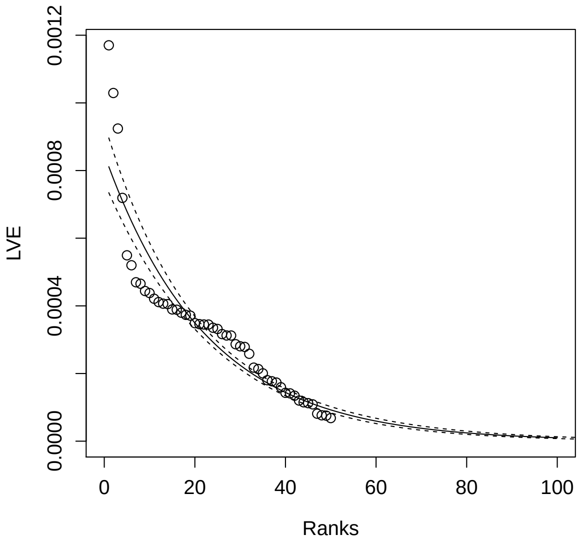


**Supplementary Figure 20**: **Fit of exponential curve to LVE distribution.** To estimate the LVE of the top (true) gene-level signals for T2D, we fit an exponential curve $\exp(an + b)$ to the LVE of the top 50 associations observed in our analysis. Shown is the fitted curve, together with the actual *p*-value ranks and LVE of the top 50 genes. Parameters of the curve are $a = 0.44$ and $b = -7.07$; dashed lines show 95% confidence intervals.

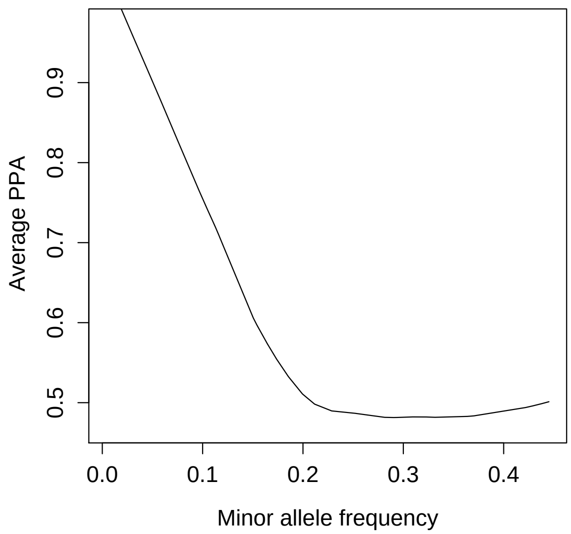


**Supplementary Figure 21**: **Dependence of coding variant PPA on MAF.** Shown is the average PPA of exome-wide significant nonsynonymous variants as a function of their minor allele frequency, as calculated from 40 recently published variants [28]. For various MAF thresholds (x-axis), the y-axis shows the average reported PPA of the variants with MAF below that threshold.

# 4 List of consortia members

## 4.1 AMP-T2D-GENES

Gonçalo R. Abecasis[1,2], Carlos A. Aguilar-Salinas[3], David M. Altshuler[4,5,6,7,8], Gil Atzmon[9,10,11], Francisco Barajas-Olmos[12], Aris Baras[13], Nir Barzilai[10], Graeme I. Bell[14], Thomas W. Blackwell[1], John Blangero[15,16], Michael Boehnke[17], Eric Boerwinkle[18,19], Lori L. Bonnycastle[20], Erwin P. Bottinger[21], Donald W. Bowden[22,23], Jennifer A. Brody[24], Brian Burke[25], Noël P. Burtt[7,8], David J. Carey[26], Lizz Caulkins[8], Federico Centeno-Cruz[12,27], John C. Chambers[28,29,30,31], Juliana Chan[32], Edmund Chan[33], Ling Chen[34], Siying Chen[17], Ching-Yu Cheng[35,36,37,38], Francis S. Collins[20], Cecilia Contreras-Cubas[12], Adolfo Correa[39], Maria Cortes[40], Nancy J. Cox[14,41], Emilio Córdova[12], Dana Dabelea[42,43], Paul S. de Vries[44], Ralph A. DeFronzo[45], Frederick E. Dewey[13], Lawrence Dolan[46], Kimberly L. Drews[25], Ravindranath Duggirala[15,16], Josée Dupuis[47,48,49], Ma Elena Gonzalez[50], Amanda Elliott[8,34], Maria Eugenia Garay-Sevilla[51], Jason Flannick[7,8,52,53], Jose C. Florez[4,6,7,8], James S. Floyd[54], Philippe Frossard[55], Christian Fuchsberger[1,56], Stacey B. Gabriel[40,57], Humberto García-Ortiz[12], Christian Gieger[58,59,60], Benjamin Glaser[61], Clicerio Gonzalez[62], Niels Grarup[63], Leif Groop[64,65,66], Myron Gross[67], Christopher A. Haiman[68], Sohee Han[69], Craig L. Hanis[70], Torben Hansen[63,71], Nancy L. Heard-Costa[47,49,72], Susan R. Heckbert[73], Brian E. Henderson[68], Soo Heon Kwak[74], Anne U. Jackson[75], Young Jin Kim[69,76], Marit E. Jørgensen[77,78,79], Megan Kelsey[25,42], Bong-Jo Kim[69], Ryan Koesterer[8], Heikki A. Koistinen[80,81,82], Jaspal S. Kooner[30,31,83,84], Johanna Kuusisto[85,86,87], Markku Laakso[85,86,87,88], Leslie A. Lange[89,90,91], Joseph B. Leader[26], Juyoung Lee[69], Jong-Young Lee[92,93], Donna M. Lehman[94], H. Lester Kirchner[26], Allan Linneberg[95,96,97,98], Ching-Ti Liu[48], Jianjun Liu[33,99,100], Ruth J. F. Loos[101], Valeriya Lyssenko[65,102], Ronald C. W. Ma[32], Anubha Mahajan[103], Alisa K. Manning[6,7,104,105], Juan Manuel Malacara-Hernandez[51], Anthony Marcketta[13], Angélica Martínez-Hernández[12], Karen Matsuo[17], Elizabeth Mayer-Davis[106], Mark I. McCarthy[103,107,108], James B. Meigs[6,40,109,110], Thomas Meitinger[111,112,113,114], Elvia Mendoza-Caamal[12,27], Josep M. Mercader[7,8,115,116], Hyun Min Kang[1], Karen L. Mohlke[89,117], Andrew D. Morris[118], Andrew P. Morris[119,120,121], Alanna C. Morrison[18], Anne Ndungu[121], Maggie C. Y. Ng[122], Peter Nilsson[123], Christopher J. O'Donnell[6,49,109,124,125,126,127], Colm O'Dushlaine[13], Lorena Orozco[12,27], Colin N. A. Palmer[128], James S. Pankow[129], Anthony J. Payne[121], Oluf B. Pedersen[63,130], Catherine Pihoker[131], Wendy S. Post[132], Michael Preuss[133], Bruce M. Psaty[24,134,135], Asif Rasheed[55], Alexander P. Reiner[136,137], Cristina Revilla-Monsalve[138], Stephen S. Rich[139,140], Neil R. Robertson[103], Jerome I. Rotter[141,142,143], Danish Saleheen[55,144], Nicola Santoro[145,146], Claudia Schurmann[147], Laura J. Scott[1], Mark Seielstad[148,149,150], Yoon Shin Cho[151], E. Shyong Tai[33,37,152,153], Xueling Sim[1,100], Robert Sladek[154,155,156], Kerrin S. Small[157], Xavier Soberón[12], Kyong Soo Park[84,158,159], Timothy D. Spector[157], Konstantin Strauch[59,60,160], Heather M. Stringham[1], Tim M. Strom[112,113,114], Claudia H. T. Tam[161], Tanya M. Teslovich[1,13], Farook Thameem[162], Brian Tomlinson[32], Jason M. Torres[14,121], Russell P. Tracy[163], Tiinamaija Tuomi[164,165,166], Jaakko Tuomilehto[167,168,169,170,171], Teresa Tusié-Luna[3,172], Miriam S. Udler[8,34], Rob M. van Dam[33,100,173], Ramachandran S. Vasan[49,174], Marijana Vujkovic[144], Shuai Wang[48], Ryan P. Welch[17], Jennifer Wessel[175,176], N. William Rayner[103,177], James G. Wilson[178], Daniel R. Witte[79,179,180], Tien-Yin Wong[38,153,181], Wing Yee So[161], Mi Yeong Hwang[69], Yik Ying Teo[182,183], Philip Zeitler[25,42]

## 4.2 T2D-GENES

Gonçalo R. Abecasis[1,2], Marcio Almeida[15], David M. Altshuler[4,5,6,7,8], Jennifer L. Asimit[184], Gil Atzmon[9,10,11], Mathew Barber[185], Nicola L. Beer[186], Graeme I. Bell[14], Jennifer Below[70], Thomas W. Blackwell[1], John Blangero[15,16], Michael Boehnke[17], Donald W. Bowden[22,23], Noël P. Burtt[7,8], John C. Chambers[28,29,30,31], Peng Chen[100], Han Chen[48], Peter S. Chines[1,20], Sungkyoung Choi[187], Claire Churchhouse[7], Pablo Cingolani[188], Belinda K. Cornes[38], Nancy J. Cox[14,41], Aaron G. Day-Williams[184], Ravindranath Duggirala[15,16], Josée Dupuis[47,48,49], Thomas Dyer[15], Shuang Feng[1], Juan Fernandez-Tajes[103], Teresa Ferreira[103], Tasha E. Fingerlin[43], Jason Flannick[7,8,52,53], Jose C. Florez[4,6,7,8], Pierre Fontanillas[7], Timothy M. Frayling[189], Christian Fuchsberger[1,56], Eric R. Gamazon[14], Kyle Gaulton[103], Saurabh Ghosh Anna Gloyn[186], Robert L.

Grossman[14], Jason Grundstad[190], Craig L. Hanis[70], Allison Heath[190], Heather Highland[70], Momoko Hirokoshi[103], Ik-Soo Huh[187], Jeroen R. Huyghe[1], Kamran Ikram[38,153,191,192], Kathleen A. Jablonski[193], Young Jin Kim[69,76], Goo Jun[1], Norihiro Kato[194], Jayoun Kim[187], Kevin Koi-Yau Lam[100], Jaspal S. Kooner[30,31,83,84], Min-Seok Kwon[187], Hae Kyung Im[195], Markku Laakso[85,86,87,88], Selyeong Lee[187], Sungyoung Lee[190], Jaehoon Lee[187], Jong-Young Lee[92,93], Donna M. Lehman[94], Heng Li[7], Cecilia M. Lindgren[103], Xuanyao Liu[196], Oren E. Livne[185], Adam E. Locke[1], Anubha Mahajan[103], Julian B. Maller[197], Alisa K. Manning[6,7,104,105], Taylor J. Maxwell[70], Alexander Mazoure[198], Mark I. McCarthy[103,107,108], James B. Meigs[6,40,109,110], Byungju Min[187], Karen L. Mohlke[89,117], Andrew P. Morris[119,120,121], Solomon K. Musani[39], Yoshihiko Nagai[198], Maggie C. Y. Ng[122], Dan Nicolae[20,185], Sohee Oh[187], Nicholette D. Palmer[199], Taesung Park[187], Toni I. Pollin[200], Inga Prokopenko[103,201], David Reich[7,202], Manuel A. Rivas[103,105,203], C. Ryan King[195], Laura J. Scott[1], Mark Seielstad[148,149,150], Yoon Shin Cho[151], E. Shyong Tai[33,37,152,153], Xueling Sim[1,100], Robert Sladek[154,155,156], Philip Smith[204], Ioanna Tachmazidou[184], Tanya M. Teslovich[1,13], Jason M. Torres[14,121], Vasily Trubetskoy[14], Sara M. Willems[205,206,207], Amy L. Williams[7,202], James G. Wilson[178], Steven Wiltshire[208], Sungho Won[209], Andrew R. Wood[189], Wang Xu[152], Yik Ying Teo[182,183], Joon Yoon[187], Matthew Zawistowski[1], Eleftheria Zeggini[184], Weihua Zhang[29], Sebastian Zöllner[210]

## 4.3 SIGMA

Irma Aguilar-DelfiÌĄn[27], Carlos A. Aguilar-Salinas[3], David M. Altshuler[4,5,6,7,8], Ulices Alvirde[3], Kristin Ardlie[57], Wendy M. Brodeur[57], Noël P. Burtt[7,8], Juan Carlos Fernández-López[27], Federico Centeno-Cruz[12,27], Claire Churchhouse[7], Emilio Córdova[27], Andrew T. Crenshaw[57], Ivette Cruz-Bautista[3], MariÌĄa Elena González-Villalpando[211], Karol Estrada[6,7,212], Timothy Fennell[7], Jose C. Florez[4,6,7,8], Jennifer Franklin[57], Diane Gage[57], Humberto GarciÌĄa-OrtiÌĄz[27], Clicerio González-Villalpando[211], DonajiÌĄ Gómez[3], Christopher A. Haiman[68], Brian E. Henderson[68], Alicia Huerta-Chagoya[3,213], Sergio Islas-Andrade[138], Suzanne B. R. Jacobs[7], MariÌĄa José Gómez-Vázquez[3,214], Laurence N. Kolonel[215], Loic Le Marchand[215], Linda Liliana Muñoz-Hernández[3], MariÌĄa Luisa Ordóñez-Sánchez[3], Daniel G. MacArthur[6,7,212], Scott Mahan[57], Alisa K. Manning[6,7,104,105], Angélica MartiÌĄnez-Hernández[27], Carla Márquez- Luna[27], Elvia Mendoza-Caamal[12,27], Josep M. Mercader[7,8,115,116], Kristine Monroe[68], Hortensia Moreno-MaciÌĄas[216], Jacquelyn Murphy[7], Benjamin Neale[7,212], Robert C. Onofrio[57], Lorena Orozco[12,27], Cristina Revilla- Monsalve[138], Laura Riba[213], Stephan Ripke[7,212], Rosario RodriÌĄguez- Guillén[3], Eunice RodriÌĄguez-Arellano[217], Maribel RodriÌĄguez-Torres[3], Sandra Romero-Hidalgo[27], Tamara Sáenz[3], Xavier Soberón[27], Daniel O. Stram[68], Teresa Tusié-Luna[3,213], Lynne Wilkens[215], Amy L. Williams[7,202], Wendy Winckler[57]

## 4.4 GoT2D

Gonçalo R. Abecasis[1,2], Vineeta Agarwala[7], Peter Algren[64], David M. Altshuler[4,5,6,7,8], Eric Banks[57], Richard N. Bergman[68], Thomas W. Blackwell[1], Michael Boehnke[17], Lori L. Bonnycastle[20], David Buck[103], Noël P. Burtt[7,8], Peter S. Chines[1,20], Francis S. Collins[20], Mark A. DePristo[7], Peter Donnelly[103], Timothy Fennell[7], Jason Flannick[7,8,52,53], Pierre Fontanillas[7], Timothy M. Frayling[189], Christian Fuchsberger[1,56], Stacey B. Gabriel[40,57], Kyle Gaulton[103], Christian Gieger[58,59,60], Harald Grallert[59], Todd Green[7], Leif Groop[64,65,66], Christopher Hartl[7], Andrew T. Hattersley[218], Bryan Howie[185], Martin Hrabé de Angelis[59], Cornelia Huth[59], Jeroen R. Huyghe[1], Bo Isomaa[64], Anne U. Jackson[75], Goo Jun[1], Jasmina Kravic[64], Jennifer Kriebel[59], Ashish Kumar[103], Phoenix Kwan[1], Claes Ladenvall[64], Cecilia M. Lindgren[103], Adam E. Locke[1], Gerton Lunter[103], Clement Ma[1], Anubha Mahajan[103], Alisa K. Manning[6,7,104,105], Mark I. McCarthy[103,107,108], Gil McVean[103], Christa Meisinger[59], Thomas Meitinger[111,112,113,114], Hyun Min Kang[1], Karen L. Mohlke[89,117], Andrew P. Morris[119,120,121], Loukas Moutsianas[103], Martina MuÌĹller-Nurasyid[59], Pål R. Njølstad[27], Richard Pearson[103], John Perry[103], Annette Peters[59], Ryan Poplin[7], Inga Prokopenko[103,201], Wolfgang Rathmann[59], Janina Ried[59], Manuel A. Rivas[103,105,203], Neil R. Robertson[103], Laura J. Scott[1], Khalid Shakir[57], Xueling Sim[1,100], Kerrin S. Small[157], Timothy D. Spector[157], Michael Stitzel[219], Konstantin Strauch[59,60,160], Heather

M. Stringham[1], Tim M. Strom[112,113,114], Adrian Tan[1], Tanya M. Teslovich[1,13], Tiinamaija Toumi[64], Jaakko Tuomilehto[167,168,169,170,171], Martijn van de Bunt[186], N. William Rayner[103,177]

## 4.5 LuCAMP

Anders Albrechtsen[220,221], Gitte Andersen[130], Arne Astrup[222], Lars Bolund[223], Torben Hansen[63,71], Torben Jørgensen[98,224], Karsten Kristiansen[220], Torsten Lauritzen[225], Rasmus Nielsen[220,221], Oluf B. Pedersen[63,130], Thue W. Schwartz[226], Jun Wang[227], Daniel R. Witte[79,179,180]

## 4.6 PRODiGY

Mary-Helen Black[228], Brian Burke[25], Ling Chen[34], Dana Dabelea[42,43], Jasmin Divers[229], Kimberly L. Drews[25], Jason Flannick[7,8,52,53], Jose C. Florez[4,6,7,8], Megan Kelsey[25,42], Alisa K. Manning[6,7,104,105], Josep M. Mercader[7,8,115,116], Toni I. Pollin[200], Nicola Santoro[145,146], Rachana Shah[230], Shylaja Srinivasan[150], Jennifer Todd[231], Philip Zeitler[25,42], Haichen Zhang[232]

## 4.7 ESP

### 4.7.1 BroadGO

Gonçalo R. Abecasis[1,2], Hooman Allayee[233], David M. Altshuler[4,5,6,7,8], Sharon Cresci[234], Mark J. Daly[105,203], Paul I. W. de Bakker[203,235,236], Mark A. DePristo[7], Ron Do[203], Peter Donnelly[103], Deborah N. Farlow[203], Timothy Fennell[7], Stacey B. Gabriel[40,57], Kiran Garimella[237], Stanley L. Hazen[238], Youna Hu[239], Daniel M. Jordan[235,240], Goo Jun[1], Sekar Kathiresan[105,203,235], Adam Kiezun[57], Guillaume Lettre[203,241,242], Mingyao Li[243], Bingshan Li[239], Hyun Min Kang[1], Christopher H. Newton-Cheh[105,203,235], Sandosh Padmanabhan[244,245], Gina M. Peloso[203,235,246,247,248], Sara Pulit[203], Daniel J. Rader[243], David Reich[7,202], Muredach P. Reilly[243], Manuel A. Rivas[103,105,203], Steve Schwartz[136], Laura J. Scott[1], David S. Siscovick[249,250], John A. Spertus[251], Nathaniel O. Stitziel[109], Nina Stoletzki[109,203,235], Shamil R. Sunyaev[109,203,235], Benjamin F. Voight[105,203], Cristen J. Willer[239]

### 4.7.2 HeartGO

L. Adrienne Cupples[47,48,49], Ermeg Akylbekova[252,253], Larry D. Atwood[47], Christie M. Ballantyne[254,255], Maja Barbalic[256], Emelia J. Benjamin[47], Joshua C. Bis[257], Eric Boerwinkle[18,19], Donald W. Bowden[22,23], Jennifer A. Brody[24], Matthew Budoff[258], Greg Burke[23], Sarah Buxbaum[252], Jeff Carr[23], Ida Y. Chen[141], Donna T. Chen[259], Wei-Min Chen[259], Pat Concannon[259], Jacy Crosby[256], Ralph D'Agostino[47], O. Dale Williams[260], Anita L. DeStefano[47], Albert Dreisbach[253], Josée Dupuis[47,48,49], Jaclyn Ellis[91], Aaron R. Folsom[261], Myriam Fornage[262], Ervin Fox[253], Caroline S. Fox[126], Vincent Funari[141], Santhi K. Ganesh[239], Julius Gardin[263], David Goff[23], Ora Gordon[141], R. Graham Barr[264], Wayne Grody[265], Myron Gross[67], Xiuqing Guo[141,143], Ira M. Hall[259], Nancy L. Heard-Costa[47,49,72], Susan R. Heckbert[73], Nicholas Heintz[231], David M. Herrington[23], DeMarc Hickson[252,253], Jie Huang[126], Shih-Jen Hwang[47,126], David R. Jacobs[261], Nancy S. Jenny[231], Craig W. Johnson[137], Andrew D. Johnson[126], Steven Kawut[243], Richard Kronmal[137], Raluca Kurz[141], Chrstina L Wassel[266], Leslie A. Lange[89,90,91], Ethan M. Lange[91,267], Martin G. Larson[47], Mark Lawson[259], Daniel Levy[126,268,269], Cora E. Lewis[270], Dalin Li[141], Honghuang Lin[47], Jiankang Liu[253], Kiang Liu[271], Xiaoming Liu[256], Yongmei Liu[272], Chunyu Liu[49,126,268], William T. Longstreth[137], Cay Loria[126], Thomas Lumley[273], Kathryn Lunetta[47], Rachel Mackey[274], Aaron J. Mackey[259], Ani Manichaikul[259], Taylor J. Maxwell[70], Barbara McKnight[137], James B. Meigs[6,40,109,110], Alanna C. Morrison[18], Solomon K. Musani[39], Josyf C. Mychaleckyj[259], Jennifer A. Nettleton[256], Kari North[91], Christopher J. O'Donnell[6,49,109,124,125,126,127], Daniel O'Leary[275], Frank Ong[141], Walter Palmas[276], James S. Pankow[129], Nathan D. Pankratz[277], Shom Paul[259], Marco Perez[278], Sharina D. Person[270,279], J. Peter Durda[231], Joseph Polak[275], Wendy S. Post[132],

Bruce M. Psaty[24,134,135], Aaron R. Quinlan[259], Leslie J. Raffel[141], Vasan S. Ramachandran[47], Alexander P. Reiner[136,137], Kenneth Rice[24], Stephen S. Rich[139,140], Jerome I. Rotter[141,142,143], Jill P. Sanders[231], Pamela Schreiner[261], Sudha Seshadri[47], Steve Shea[109,240], Stephen Sidney[280], Kevin Silverstein[261], David S. Siscovick[249,250], Nicholas L. Smith[137], Nona Sotoodehnia[137], Asoke Srinivasan[281], Herman A. Taylor[252,253,281], Kent D. Taylor[141,143], Fridtjof Thomas[256], Russell P. Tracy[163], Michael Y. Tsai[261], Kelly A. Volcik[256], Karol Watson[265], Gina Wei[126], Wendy White[281], Kerri L. Wiggins[231], Jemma B. Wilk[47], Gregory Wilson[252], James G. Wilson[178], Phillip Wolf[47], Neil A. Zakai[231]

### 4.7.3 ISGS and SWISS

John Hardy[282,283,284], James F. Meschia[285], Michael A. Nalls[286], Stephen S. Rich[139,140], Andrew Singleton[287], Brad Worrall[259]

### 4.7.4 LungGO

Ibrahim Abdulhamid[288], Frank Accurso[289], Ran Anbar[290], Mary Ann Passero[291], Michael J. Bamshad[131,137], Kathleen C. Barnes[292], Terri Beaty[292], Abigail Bigham[137], Phillip Black[293], Eugene Bleecker[23], Kati Buckingham[137], Daniel Caplan[294], Barbara Chatfield[295], Wei-Min Chen[259], Aaron Chidekel[296], Michael Cho[109,235], David C. Christiani[105], James D. Crapo[297], Julia Crouch[131], Denise Daley[298], Hong Dang[91], Anthony Dang[91], Alicia De Paula[299], Joan DeCelie- Germana[300], Allen Dozor[301,302], Mitch Drumm[91], Maynard Dyson[303], Julia Emerson[131,137], Mary J. Emond[137], Thomas Ferkol[234,304], Robert Fink[305], Cassandra Foster[292], Deborah Froh[259], Li Gao[292], William Gershan[306], Ronald L. Gibson[131,137], Elizabeth Godwin[91], Magdalen Gondor[307], Hector Gutierrez[270], Nadia N. Hansel[292,308], Paul M. Hassoun[292], Peter Hiatt[309], John E. Hokanson[289], Michelle Howenstine[310,311], Laura K. Hummer[292], Jamshed Kanga[312], Yoonhee Kim[313], Michael R. Knowles[91], Michael Konstan[314], Thomas Lahiri[315], Nan Laird[316], Christoph Lange[316], Xihong Lin[316], Lin Lin[235], Tin L. Louie[137], David Lynch[297], Barry Make[297], Anne Marie Cairns[317], Thomas R. Martin[137,318], Steve C. Mathai[292], Rasika A. Mathias[292,319], Sharon McNamara[131], John McNamara[320], Deborah Meyers[23], Susan Millard[321,322], Peter Mogayzel[292], Richard Moss[323], Tanda Murray[292], Dennis Nielson[150], Blakeslee Noyes[324], Wanda O'Neal[91], Brian O'Sullivan[325], David Orenstein[326], Rhonda Pace[91], Peter Pare[327], Elizabeth Perkett[328], Adrienne Prestridge[329], Nicholas M. Rafaels[292], Bonnie Ramsey[131,137], Elizabeth Regan[297], Clement Ren[330], George Retsch-Bogart[91], Michael Rock[331], Antony Rosen[292], Margaret Rosenfeld[131,137], Ingo Ruczinski[308], Andrew Sanford[298], David Schaeffer[332], Cindy Sell[91], Daniel Sheehan[333], Edwin K. Silverman[109,235], Don Sin[305], Terry Spencer[334], Jackie Stonebraker[91], Holly K. Tabor[131,137], Laurie Varlotta[335], Candelaria I. Vergara[292], Fred Wigley[292], Robert A. Wise[292], H. Worth Parker[336,337], Fred A. Wright[91], Mark M. Wurfel[137], Robert Zanni[338], Fei Zou[91]

### 4.7.5 SeattleGO

Joshua M. Akey[137], Michael J. Bamshad[131,137], Carlos D. Bustamante[278], David R. Crosslin[137], Evan E. Eichler[137], Wenqing Fu[137], Adam Gordon[137], Simon Gravel[278], Phil Green[137], Gail P. Jarvik[137], Jill M. Johnsen[137,339], Mengyuan Kan[254], Eimear E. Kenny[278], Jeffrey M. Kidd[278], Fremiet Lara-Garduno[254], Suzanne M. Leal[254], Dajiang J. Liu[254], Sean McGee[137], Deborah A. Nickerson[137], Timothy D. O'Connor[137], Bryan Paeper[137], Mark J. Rieder[340], Peggy D. Robertson[137], Jay Shendure[137], Joshua D. Smith[137], Jacob A. Tennessen[137], Emily H. Turner[137], Gao Wang[254]

### 4.7.6 WHISP

Garnet Anderson[136], Hoda Anton-Culver[341], Themistocles L. Assimes[278], Paul L. Auer[136], Shirley Beresford[136], Chris Bizon[91], Henry Black[342], Robert Brunner[343], Robert Brzyski[256], Dale Burwen[126], Bette Caan[280], Christopher S. Carlson[136,137], Cara L. Carty[136], Rowan Chlebowski[344], Steven Cummings[150], J. David

Curb[345], Charles B. Eaton[346,347], Leslie Ford[126], Nora Franceschini[91], Stephanie M. Fullerton[137], Margery Gass[348], Nancy Geller[126], Gerardo Heiss[91], Barbara V. Howard[349,350], Li Hsu[136], Carolyn M. Hutter[136], John Ioannidis[278], Rebecca Jackson[351], Shuo Jiao[136], Mary Jo O'Sullivan[352], Karen C. Johnson[353], Charles Kooperberg[136], Lewis Kuller[274], Andrea LaCroix[136], Kamakshi Lakshminarayan[261], Dorothy Lane[354], Leslie A. Lange[89,90,91], Ethan M. Lange[91,267], Norman Lasser[355], Erin LeBlanc[356], Cora E. Lewis[270], Kuo-Ping Li[91], Marian Limacher[357], Dan-Yu Lin[91], Benjamin A. Logsdon[136], Shari Ludlam[126], JoAnn E. Manson[109,316], Karen Margolis[261], Lisa Martin[358], Joan McGowan[126], Keri L. Monda[359], Jane Morley Kotchen[360], Lauren Nathan[265], Kari North[91], Judith Ockene[361,362], Ulrike Peters[136], Lawrence S. Phillips[294], Ross L. Prentice[136], Alexander P. Reiner[136,137], John Robbins[363], Jennifer G. Robinson[364], Jacques E. Rossouw[126], Haleh Sangi-Haghpeykar[254], Gloria E. Sarto[365], Sally Shumaker[23], Michael S. Simon[366], Marcia L. Stefanick[278], Evan Stein[367], Hua Tang[323], Kira C. Taylor[368], Cynthia A. Thomson[369], Timothy A. Thornton[137], Linda Van Horn[271], Mara Vitolins[23], Jean Wactawski-Wende[370], Robert Wallace[364], Sylvia Wassertheil-Smoller[47], Donglin Zeng[91]

#### 4.7.7 NHLBI GO ESP Project Team

Deborah Applebaum-Bowden[126], Michael Feolo[371], Weiniu Gan[126], Dina N. Paltoo[126], Jacques E. Rossouw[126], Phyliss Sholinsky[126], Anne Sturcke[371]

### 4.8 CHARGE

Ravinder Abrol[372,373], L. Adrienne Cupples[47,48,49], Kristine H. Allin[63], Najaf Amin[206], Ping An[374], Jennifer L. Aponte[375], Tin Aung[38,192], Abigail S. Baldridge[376], Caterina Barbieri[377], Diane M. Becker[319], Céline Besse[378], Nathan A. Bihlmeyer[379,380], Joshua C. Bis[257], Michael Boehnke[17], Heiner Boeing[381], Eric Boerwinkle[18,19], Anne Boland[378], Cristina Bombieri[382], Ingrid B. Borecki[374], Jette Bork-Jensen[63], Erwin P. Bottinger[21], Donald W. Bowden[22,23], Jennifer A. Brody[24], James B. Brown[221,383], Sean M. Burns[247], Daniel I. Chasman[384], Yuning Chen[48], Ching-Yu Cheng[35,36,37,38], Audrey Y. Chu[384], Adolfo Correa[39], Jacek Czajkowski[374], George Dedoussis[385], Abbas Dehghan[386], Panos Deloukas[184,387,388], Yii- Der I. Chen[143], Josée Dupuis[47,48,49], Margaret G. Ehm[389], Georg B. Ehret[390,391], Gudny Eiriksdottir[392], Stefan A. Escher[393], Aliki-Eleni Farmaki[394], Ele Ferrannini[395], Jose C. Florez[4,6,7,8], Myriam Fornage[262], Keolu Fox[396], Oscar H. Franco[386], Paul W. Franks[173,393,397], Timothy M. Frayling[189], Mattias Frånberg[398,399], Daniel F. Freitag[184,400], Giovanni Gambaro[401], Melissa E. Garcia[287], Richard A. Gibbs[19], Franco Giulianini[384], William A. Goddard III[373], Anuj Goel[402], Mark O. Goodarzi[372], Omri Gottesman[21], Niels Grarup[63], Megan L. Grove[18], Vilmundur Gudnason[392,403], Xiuqing Guo[141,143], Stefan Gustafsson[201], Yang Hai[143], Göran Hallmans[404], Anders Hamsten[398], Torben Hansen[63,71], Kazuo Hara[147], Tamara B. Harris[287], Andrew T. Hattersley[218], Caroline Hayward[405], Jiyoung Heo[406], Bertha Hidalgo[407], Albert Hofman[386], Jing Hua Zhao[207], Jennifer E. Huffman[405], Mohammad K. Ikram[35,38,408], Erik Ingelsson[103,201], Aaron Isaacs[206], Johanna Jakobsdottir[392], Jan-Håkan Jansson[248,397], Sundas Javad[207], Richard A. Jensen[24], Marit E. Jørgensen[77,78,79], Torben Jørgensen[98,224], Maria Karaleftheri[409], Andrew J. Karter[410], Chiea C. Khor[37,411], Andrea Kirkpatrick[373], Aldi T. Kraja[374], Johanna Kuusisto[85,86,87], Markku Laakso[85,86,87,88], Leslie A. Lange[89,90,91], Ethan M. Lange[91,267], Claudia Langenberg[207], Lenore J. Launer[287], Jill C. Layton[412], I.T. Lee[413], Wen-Jane Lee[414], Aaron Leong[6,110], Daniel Levy[126,268,269], Man Li[415], Li Li[389], Jiemin Liao[38,192], WH Linda Kao[416], Cecilia M. Lindgren[103], Allan Linneberg[95,96,97,98], Leonard Lipovich[417,418], Chunyu Liu[49,126,268], Yongmei Liu[272], Ruth J. F. Loos[101], Carlos Lorenzo[45], Yingchang Lu[147], Paul M Ridker[419,420], Giovanni Malerba[382], Vasiliki Mamakou[421], Eirini Marouli[422], Nisa M. Maruthur[423,424], Angela Matchan[184], Rasika A. Mathias[292,319], Roberta McKean[425], Olga McLeod[398], Karina Meidtner[426], James B. Meigs[6,40,109,110], Ginger A. Metcalf[19], Karen L. Mohlke[89,117], Alanna C. Morrison[18], Donna M. Muzny[19], Michael A. Nalls[286], Jill M. Norris[427], Ioanna Ntalla[428], Christopher J. O'Donnell[6,49,109,124,125,126,127], Ben A. Oostra[206], Stephen O'Rahilly[429], Sandosh Padmanabhan[244,245], Nicholette D. Palmer[199], James S. Pankow[129], Dorota Pasko[189], Oluf B. Pedersen[63,130], Gina M. Peloso[203,235,246,247,248],

Andreas Peter[430,431], Marjolein J. Peters[246,432], Ozren Polasek[433], Michael A. Province[374], Bruce M. Psaty[24,134,135], Sridharan Raghavan[6,110], Laura J. Rasmussen-Torvik[376], Nigel W. Rayner[184,434,435], Frida Renström[393], Kenneth Rice[24], Stephen S. Rich[139,140], Jerome I. Rotter[141,142,143], Igor Rudan[436], Maria Sabater- Lleal[398], Cinzia F. Sala[377], Matthias B. Schulze[426,431], Claudia Schurmann[147], Generation Scotland[437], Robert A. Scott[207], Bengt Sennblad[438], Ioannis Serafetinidis[439], Wayne H.-H. Sheu[413,440,441], E. Shyong Tai[33,37,152,153], Angela Silveira[398], David S. Siscovick[249,250], Albert V. Smith[392,403], Jennifer A. Smith[442], Blair Smith[443], Nicole Soranzo[184,444], Lorraine Southam[184,435], Elizabeth K. Speliotes[445], Eli A. Stahl[446], Alena StancÌŇáková[87], Kathleen Stirrups[184,388], Marcus H. Stoiber[383], Rona J. Strawbridge[398], Kent D. Taylor[141,143], Nikos Tentolouris[447], Anastasia Thanopoulou[448], Daniela Toniolo[377], Mina Torres[425], Michela Traglia[377], Emmanouil Tsafantakis[449], André G. Uitterlinden[246], Dhananjay Vaidya[319], Cornelia M. van Duijn[206,450], Tibor V. Varga[451], Rohit Varma[425], Lynne E. Wagenknecht[452], Mark Walker[57,453], Shuai Wang[48], Nicholas J. Wareham[207], Dawn M. Waterworth[375], Hugh Watkins[402], Jennifer Wessel[175,176], Sara M. Willems[205,206,207], James G. Wilson[178], Tien Y. Wong[35,38,192], Hanieh Yaghootkar[189], Lisa R. Yanek[319], Eleftheria Zeggini[184], Eleni Zengini[454,455]

## 4.9 DiscovEHR

Gonçalo R. Abecasis[1,2], Lance J. Adams[26], W. Andrew Faucett[26], Xiaodong Bai[2], Suganthi Balasubramanian[2], Nilanjana Banerjee[2], Aris Baras[13], Leland Barnard[2], Christina Beechert[2], Andrew Blumenfeld[2], Derek Boris[26], Michael Cantor[2], David J. Carey[26], Yating Chai[2], Ryan D. Colonie[26], Giovanni Coppola[2], Amy Damask[2], F. Daniel Davis[26], Aris Economides[2], Gisu Eom[2], Caitlin Forsythe[2], Erin D. Fuller[2], Zhenhua Gu[2], Lauren Gurski[2], Lukas Habegger[2], Young Hahn[2], Dustin N. Hartzel[26], Alicia Hawes[2], Shareef Khalid[2], Michael Lattari[2], Joseph B. Leader[26], David H. Ledbetter[26], H. Lester Kirchner[26], Alexander Li[2], Nan Lin[2], Daren Liu[2], Alexander Lopez[2], Kia Manoochehri[2], Jonathan Marchini[2], Anthony Marcketta[13], Christa L. Martin[26], Evan K. Maxwell[2], Shane McCarthy[2], Raghu P. Metpally[26], Tooraj Mirshahi[26], J. Neil Manus[26], Matthew Oetjens[26], John D. Overton[2], Colm O'Dushlaine[2], Sarah A. Pendergrass[26], John Penn[2], Thomas N. Person[26], Manasi Pradhan[2], Jeffrey G. Reid[2], Thomas D. Schleicher[2], Alan Shuldiner[2], Maria Sotiropoulos Padilla[2], Jeffrey C. Staples[2], Christopher Still[26], Karina Toledo[2], Ricardo H. Ulloa[2], Jen Wagner[26], Louis Widom[2], Huntington F. Willard[26], Marc Williams[26], Sarah E. Wolf[2], Ashish Yadav[2]

## 4.10 Broad Genomics Platform

Adal Abebe[57], Justin Abreu[57], David An[57], Kristin Anderka[57], Scott Anderson[57], Chamara Aneesha Jayesinghe[57], Maryam Aqchour[57], Joshua Araya[57], Samuel Aronson[57], Mehrtash Babadi[57], Sarah Babchuck[57], Samira Bahl[57], Esme Baker[57], Eric Banks[57], Jessica Barbagallo[57], Alexander Baumann[57], Matthew Bemis[57], David Benjamin[57], Louis Bergelson[57], Kylee Bergin[57], Dave Bernick[57], Andrew Bernier[57], Amy Biasella[57], Jonathan Bistline[57], Brendan Blumenstiel[57], Nicole Bolliger[57], Claude Bonnet[57], Patrick Brehio[57], Wendy M. Brodeur[57], Joseph BuAbbud[57], Jody Camarata[57], Jay Carey[57], Yee-Ming Chan[57], Sheila Chandran[57], Nikita Chauhan[57], Aaron Chevalier[57], Carrie Cibulskis[57], Kristian Cibulskis[57], Michelle Cipicchio[57], Kristen Connolly[57], Maura Costello[57], Miguel Covarrubias[57], Vivek Dasari[57], Michael Dasilva[57], Tim De Smet[57], Matthew DeFelice[57], Samuel DeLuca[57], Katerina Dimitriou[57], Jacqueline Dion[57], Christine DiTondo[57], Gary Dlugy[57], Sheila Dodge[57], Teni Dowdell[57], Phil Dunlea[57], Hussein Elgridly[57], M. Erik Husby[57], Yossi Farjoun[57], Anna Farrell[57], Damien Fenske-Corbiere[57], Henry Ferrara[57], Steven Ferriera[57], Nicholas Fitzgerald[57], Mark Fleharty[57], Leo Forconesi[57], Scott Frazer[57], Stacey B. Gabriel[40,57], Laura Gauthier[57], Christina Gearin[57], Jeff Gentry[57], Linley Gerber[57], Diego Gil[57], Alexandra Gkrekos[57], Douglas Gobron[57], George Grant[57], Lisa Green[57], Liraz Greenfeld[57], Jonna Grimsby[57], Namrata Gupta[57], Kunsang Gyaltsen[57], Susanna Hamilton[57], Maegan Harden[57], Andreina Haubold[57], Soo Hee Lee[57], Jen Hendrey[57], Maria Hofbauer[57], Andrew Hollinger[57], Laurie Holmes[57], Tom Howd[57], Steve Huang[57], Dong-Keun Jang[57], Victoria Janik[57], Thibault Jeandet[57], Fontina Kelley[57], David Kennedy[57], Adam Kiezun[57], Kevinson Kim[57], David Kling[57], Jessica Klopp[57], Anna Koutoulas[57], Katie Larkin[57], Erin LaRoche[57], Katie Larsson[57], Zach Leber[57], James

Lee[57], Samuel Lee[57], Matthew Lee[57], Marcia Leffler[57], Niall Lennon[57], Frances Letendre[57], Tsamla Lhanyitsang[57], Lee Lichtenstein[57], Pei Lin[57], Christopher Llanwarne[57], Walter Lo Forte[57], Nadya Lopez Zalba[57], Sophie Low[57], Hayley Lyon[57], Jose M Soto[57], Alyssa Macbeth[57], Vasilia Magnisalis[57], Zainab Mahmod[57], Tsheko Makuwa[57], Lauren Margolin[57], Tamara Mason[57], Scott Matthews[57], Susan McDonough[57], Sheli McDonough[57], Thomas McKenna[57], Jim Meldrim[57], Atanas Mihalev[57], Mariela Mihaleva[57], Tiffany Miller[57], Tyler Miselis[57], David Mohs[57], Ruchi Munshi[57], Moran N Cabili[57], Gregory Nakashian[57], Jared Nedzel[57], Duyen Nguyen[57], Kate Noblett[57], Corey Nolet[57], Nyima Norbu[57], Sam Novod[57], Robert C. Onofrio[57], Caroline Petersen[57], Anthony Philippakis[57], Eliot Polk[57], Sam Pollock[57], Mark Puppo[57], Jason Purnell[57], Matt Putnam[57], Anabella Racioppi[57], Brian Reilly[57], David Roazen[57], Nathan Rodriguez[57], Jason Rose[57], Erika Roth[57], Valentin Ruano-Rubio[57], Gregory Rushton[57], Dennis Ryan[57], John Saccoccio[57], Ahmed Sandakli[57], Takuto Sato[57], Michael Saylor[57], Khalid Shakir[57], Megan Shand[57], Ted Sharpe[57], David Shiga[57], David Siedzik[57], Anu Singh[57], Kara Slowik[57], Andrey Smirnov[57], Sharon Stavropoulos[57], Gregory Stoneham[57], Scott Sutherland[57], Bradley Taylor[57], Joel Thibault[57], Jon Thompson[57], Kathleen Tibbetts[57], Charlotte Tolonen[57], Kristina Tracy[57], Ellen Tsai[57], Adrienne Turi[57], Geraldine Van der Auwera[57], Diolinda Vazl[57], Veronica Vicario[57], Gina Vicente[57], Andy Vo[57], Douglas Voet[57], Sarah Walker[57], Mark Walker[57,453], Cole Walsh[57], John Walsh[57], Emily Wheeler[57], Jill Whitham[57], Jane Wilkinson[57], Michael Wilson[57], David Wilson[57], Ellen Winchester[57], David Wine[57], Alicia Wong[57], Betty Woolf[57], David Zdeb[57], Andrew Zimmer[57]

## 4.11 Affiliations

1. Department of Biostatistics, Center for Statistical Genetics, University of Michigan, Ann Arbor, MI, USA
2. Regeneron Pharmaceuticals, Tarrytown, NY, USA
3. Instituto Nacional de Ciencias Médicas y Nutrición Salvador Zubirán, Mexico City, Mexico
4. Center for Human Genetic Research, Massachusetts General Hospital, Boston, MA, USA
5. Department of Biology, Massachusetts Institute of Technology, Cambridge, MA, USA
6. Department of Medicine, Harvard Medical School, Boston, MA, USA
7. Program in Medical and Population Genetics, Broad Institute of Harvard and MIT, Cambridge, MA, USA
8. Programs in Metabolism and Medical and Population Genetics, Broad Institute of MIT and Harvard, Cambridge, MA, USA
9. Department of Medicine, Department of Genetics, Albert Einstein College of Medicine, New York, NY, USA
10. Departments of Medicine and Genetics, Albert Einstein College of Medicine, NY, USA
11. University of Haifa, Faculty of Natural Science, Haifa, Isarel
12. Instituto Nacional de Medicina Genómica, Mexico City, Mexico
13. Regeneron Genetics Center, Regeneron Pharmaceuticals, Tarrytown, NY, USA
14. Department of Medicine, University of Chicago, Chicago, IL, USA
15. Department of Genetics, Texas Biomedical Research Institute, San Antonio, TX, USA

16. Department of Human Genetics and South Texas Diabetes and Obesity Institute, University of Texas Rio Grande Valley, Edinburg and Brownsville, TX, USA

17. Department of Biostatistics and Center for Statistical Genetics, University of Michigan, Ann Arbor, MI, USA

18. Human Genetics Center, School of Public Health, University of Texas Health Science Center, San Antonio, TX, USA

19. Human Genome Sequencing Center, Baylor College of Medicine, Houston, TX, USA

20. National Human Genome Research Institute, National Institutes of Health, Bethesda, MD, USA

21. The Charles Bronfman Institute for Personalized Medicine, Icahn School of Medicine at Mount Sinai, New York, NY, USA

22. Department of Biochemistry, Wake Forest School of Medicine, Winston-Salem, NC, USA

23. Wake Forest University, Winston-Salem, NC, USA

24. Cardiovascular Health Research Unit, University of Washington, Seattle, WA, USA

25. Biostatistics Center, George Washington University, Washington, DC , USA

26. Geisinger Health System, Danville, PA, USA

27. Instituto Nacional de Medicina Genómica, Mexico City, Mexico

28. Department of Cardiology, Ealing Hospital NHS Trust, Southall, Middlesex, UK

29. Department of Epidemiology and Biostatistics, Imperial College London, London, UK

30. Ealing Hospital National Health Service (NHS) Trust, Middlesex, UK

31. Imperial College Healthcare NHS Trust, London, UK

32. Department of Medicine and Therapeutics, Chinese University of Hong Kong, Hong Kong, China

33. Department of Medicine, Yong Loo Lin School of Medicine, National University of Singapore, National University Health System, Singapore

34. Diabetes Research Center (Diabetes Unit), Department of Medicine, Massachusetts General Hospital, Boston, MA, USA

35. Office of Clinical Sciences, Duke National University of Singapore Graduate Medical School, National University of Singapore, Singapore

36. Ophthalmology and Visual Sciences Academic Clinical Program (Eye ACP), Duke National University of Singapore Graduate Medical School, Singapore

37. Saw Swee Hock School of Public Health, National University of Singapore and National University Health System, Singapore

38. Singapore Eye Research Institute, Singapore National Eye Centre, Singapore

39. Department of Medicine, University of Mississippi Medical Center, Jackson, MS, USA

40. Broad Institute of MIT and Harvard, Cambridge, MA, USA

41. Vanderbilt Genetics Institute, Vanderbilt University, Nashville, TN, USA
42. Children's Hospital Colorado, Aurora, CO, USA
43. Department of Epidemiology, Colorado School of Public Health, Aurora, CO, USA
44. Human Genetics Center, Department of Epidemiology Human Genetics and Environmental Sciences, School of Public Health, University of Texas Health Science Center, San Antonio, TX, USA
45. Department of Medicine, University of Texas Health Science Center, San Antonio, TX, USA
46. Cincinnati Children's Hospital Medical Center, Cincinnati, OH, USA
47. Boston University, Boston, MA, USA
48. Department of Biostatistics, Boston University School of Public Health, Boston, MA, USA
49. National Heart, Lung, and Blood Institute Framingham Heart Study, Framingham, MA, USA
50. Centro de Estudios en Diabetes, Mexico City, Mexico
51. Departments of Medicine and Human Genetics, University of Chicago, Chicago, IL, USA
52. Department of Pediatrics, Harvard Medical School, Boston, MA, USA
53. Division of Genetics and Genomics, Boston Children's Hospital, Boston, MA, USA
54. Department of Medicine and Epidemiology, University of Washington, Seattle, WA, USA
55. Center for Non-Communicable Diseases, Karachi, Pakistan
56. Institute for Biomedicine, Eurac Research, Bolzano, Italy
57. Genomics Platform, Broad Institute of Harvard and MIT, Cambridge, MA, USA
58. German Center for Diabetes Research (DZD e.V.), Neuherberg, Germany
59. Institute of Genetic Epidemiology, Helmholtz Zentrum MuÌĹnchen, Neuherberg, Germany
60. Research Unit of Molecular Epidemiology, Institute of Epidemiology, Helmholtz Zentrum München, German Research Center for Environmental Health, Neuherberg, Germany
61. Endocrinology and Metabolism Service, Hadassah-Hebrew University Medical Center, Jerusalem, Israel
62. Unidad de Diabetes y Riesgo Cardiovascular, Instituto Nacional de Salud Pública, Cuernavaca, Morelos, Mexico
63. The Novo Nordisk Foundation Center for Basic Metabolic Research, Faculty of Health and Medical Sciences, University of Copenhagen, Copenhagen, Denmark
64. Department of Clinical Sciences, Diabetes and Endocrinology, Clinical Research Centre, Lund University, Malmö, Sweden
65. Department of Clinical Sciences, Diabetes and Endocrinology, Lund University Diabetes Centre, Malmö, Sweden
66. Institute for Molecular Genetics Finland, University of Helsinki, Helsinki, Finland

67. Department of Laboratory Medicine and Pathology, University of Minnesota, Minneapolis, MN, USA

68. Department of Preventive Medicine, Keck School of Medicine, University of Southern California, Los Angeles, CA, USA

69. Division of Genome Research, Center for Genome Science, National Institute of Health, Chungcheongbuk-do, Republic of Korea

70. Human Genetics Center, University of Texas Health Science Center, San Antonio, TX, USA

71. Faculty of Health Sciences, University of Southern Denmark, Odense, Denmark

72. Department of Neurology, Boston University School of Medicine, Boston, MA, USA

73. Cardiovascular Health Research Unit and Department of Epidemiology, University of Washington, Seattle, WA, USA

74. Department of Internal Medicine, Seoul National University Hospital, Seoul, Republic of Korea

75. Department of Pathology, University of Michigan, Ann Arbor, MI, USA

76. Department of Neurology, Konkuk University School of Medicine, Seoul, South Korea

77. Greenland Centre for Health Research, University of Greenland, Nuuk, Greenland

78. National Institute of Public Health, University of Southern Denmark, Odense, Denmark

79. Steno Diabetes Center, Gentofte, Denmark

80. Department of Public Health Solutions, National Institute for Health and Welfare, Helsinki, Finland

81. Minerva Foundation Institute for Medical Research, Helsinki, Finland

82. University of Helsinki and Department of Medicine, Helsinki University Central Hospital, Helsinki, Finland

83. National Heart and Lung Institute (NHLI), Imperial College London, Hammersmith Hospital, London, UK

84. National Heart and Lung Institute, Cardiovascular Sciences, Hammersmith Campus, Imperial College London, London, UK

85. Department of Medicin, Kuopio University Hospital, Kuopio, Finland

86. Institute of Clinical Medicine, Internal Medicine, University of Eastern Finland and Kuopio University Hospital, Kuopio, Finland

87. Institute of Clinical Medicine, Internal Medicine, University of Eastern Finland, Kuopio, Finland

88. Department of Medicine, University of Eastern Finland, Kuopio Campus and Kuopio University Hospital, Kuopio, Finland

89. Department of Genetics, University of North Carolina, Chapel Hill, NC, USA

90. Department of Medicine, University of Colorado Denver, Anschutz Medical Campus, Aurora, CO, USA

91. University of North Carolina Chapel Hill, Chapel Hill, NC, USA

92. Center for Genome Science, Korea National Institute of Health, Osong Health Technology Administration Complex, Chungcheongbuk-do, South Korea

93. Department of Business Data Convergence, Chungbuk National University, Gyeonggi-do, Republic of Korea

94. Division of Clinical Epidemiology, Department of Medicine, University of Texas Health Science Center, San Antonio, TX, USA

95. Center for Clinical Research and Prevention, Bispebjerg and Frederiksberg Hospital, Copenhagen, Denmark

96. Department of Clinical Experimental Research, Rigshospitalet, Copenhagen, Denmark

97. Department of Clinical Medicine, Faculty of Health and Medical Sciences, University of Copenhagen, Copenhagen, Denmark

98. Research Centre for Prevention and Health, Glostrup University Hospital, Glostrup, Denmark

99. Genome Institute of Singapore, Agency for Science Technology and Research, Singapore

100. Saw Swee Hock School of Public Health, National University of Singapore, Singapore

101. The Mindich Child Health and Development Institute, Icahn School of Medicine at Mount Sinai, New York, NY, USA

102. University of Bergen, Bergen, Norway

103. Wellcome Trust Centre for Human Genetics, University of Oxford, Oxford, UK

104. Clinical and Translational Epidemiology Unit, Massachusetts General Hospital, Harvard University, Cambridge, MA, USA

105. Massachusetts General Hospital, Boston, MA, USA

106. University of North Carolina, Chapel Hill, NC, USA

107. Oxford NIHR Biomedical Research Centre, Churchill Hospital, Headington, UK

108. Oxford NIHR Biomedical Research Centre, Oxford University Hospitals Trust, Oxford, UK

109. Brigham and Women's Hospital, Boston, MA, USA

110. General Medicine Division, Massachusetts General Hospital, Boston, MA, USA

111. Deutsches Forschungszentrum für Herz-Kreislauferkrankungen (DZHK), Partner Site Munich Heart Alliance, Munich, Germany

112. Human Genetics, Helmholtz Zentrum MuÌĹnchen, Neuherberg, Germany

113. Institute of Human Genetics, Helmholtz Zentrum München, German Research Center for Environmental Health, Neuherberg, Germany

114. Institute of Human Genetics, Technische Universität München, Munich, Germany

115. Center for Human Genetic Research and Diabetes Research Center (Diabetes Unit), Massachusetts General Hospital, Boston, MA, USA

116. Joint BSC-CRG-IRB Research Program in Computational Biology, Barcelona Supercomputing Center, Barcelona, Spain

117. Department of Genetics, University of North Carolina Chapel Hill, Chapel Hill, NC, USA

118. Clinical Research Centre, Centre for Molecular Medicine, Ninewells Hospital and Medical School, Dundee, UK

119. Department of Biostatistics, University of Liverpool, Liverpool, UK

120. Department of Genetic Medicine, Manchester Academic Health Sciences Centre, Manchester, UK

121. Wellcome Centre for Human Genetics, Nuffield Department of Medicine, University of Oxford, Oxford, UK

122. Center for Genomics and Personalized Medicine Research, Center for Diabetes Research, Department of Biochemistry, Department of Internal Medicine, Wake Forest School of Medicine, Winston-Salem, NC, USA

123. Department of Clinical Sciences, Medicine, Lund University, Malmö, Sweden

124. Cardiology Division, Massachusetts General Hospital, Boston, MA, USA

125. Intramural Administration Management Branch, National Heart, Lung, and Blood Institute, NIH, Framingham, MA, USA

126. National Heart, Lung, and Blood Institute, Bethesda, MD, USA

127. Section of Cardiology, Department of Medicine, VA Boston Healthcare, Boston, MA, USA

128. Pat Macpherson Centre for Pharmacogenetics and Pharmacogenomics, Medical Research Institute, Ninewells Hospital and Medical School, Dundee, UK

129. Division of Epidemiology and Community Health, School of Public Health, University of Minnesota, Minneapolis, MN, USA

130. Hagedorn Research Institute, Gentofte, Denmark

131. Seattle Children's Hospital, Seattle, WA, USA

132. Division of Cardiology, Department of Medicine, Johns Hopkins University, Baltimore, MD, USA

133. Charles R. Bronfman Institute of Personalized Medicine, Icahn School of Medicine at Mount Sinai, New York, NY, USA

134. Group Health Research Institute, Seattle, WA, USA

135. Kaiser Permanente Washington Health Research Institute, Seattle, WA, USA

136. Fred Hutchinson Cancer Research Center, Seattle, WA, USA

137. University of Washington, Seattle, WA, USA

138. Instituto Mexicano del Seguro Social SXXI, Mexico City, Mexico

139. Center for Public Health Genomics, Department of Public Health Sciences, University of Virginia, Charlottesville, VA, USA

140. Center for Public Health Genomics, University of Virginia School of Medicine, Charlottesville, VA, USA

141. Cedars-Sinai Medical Center, Los Angeles, CA, USA

142. Departments of Pediatrics and Medicine, Institute for Translational Genomics and Population Sciences, Los Angeles BioMedical Research Institute at Harbor-UCLA Medical Center, Torrance, CA, USA

143. Institute for Translational Genomics and Population Sciences, Los Angeles Biomedical Research Institute at Harbor-UCLA Medical Center, Torrance, CA, USA

144. Department of Biostatistics and Epidemiology, University of Pennsylvania, Philadelphia, PA, USA

145. Department of Pediatrics, Yale University, New Haven, CT, USA

146. Yale School of Medicine, New Haven, CT, USA

147. The Genetics of Obesity and Related Metabolic Traits Program, Icahn School of Medicine at Mount Sinai, New York, NY, USA

148. Blood Systems Research Institute, San Francisco, CA, USA

149. Department of Laboratory Medicine and Institute for Human Genetics, University of California, San Francisco, San Francisco, CA, USA

150. University of California San Francisco, San Francisco, CA, USA

151. Department of Biomedical Science, Hallym University, Gangwon-do, South Korea

152. Department of Medicine, Yong Loo Lin School of Medicine, National University of Singapore, Singapore

153. Duke National University of Singapore Graduate Medical School, Singapore

154. Department of Human Genetics, McGill University, Montréal, Québec, Canada

155. Department of Medicine, Royal Victoria Hospital, Montréal, Québec, Canada

156. McGill University and Génome Québec Innovation Centre, Montreal, Quebec, Canada

157. Department of Twin Research and Genetic Epidemiology, King's College London, London, UK

158. Department of Internal Medicine, Seoul National University College of Medicine, Seoul, Republic of Korea

159. Department of Molecular Medicine and Biopharmaceutical Sciences, Graduate School of Convergence Science and Technology, Seoul National University, Seoul, South Korea

160. Institute of Medical Informatics, Biometry and Epidemiology, Chair of Genetic Epidemiology, Ludwig-Maximilians-Universität, Neuherberg, Germany

161. Hong Kong Institute of Diabetes and Obesity, Chinese University of Hong Kong, Hong Kong, China

162. Department of Biochemistry, Faculty of Medicine, Health Science Center, Kuwait University, Safat, Kuwait

163. Department of Pathology and Laboratory Medicine, Robert Larner, M.D. College of Medicine, University of Vermont, Burlington, VT, USA

164. Department of Endocrinology, Abdominal Centre, Helsinki University Hospital, Helsinki, Finland

165. Folkhälsan Research Centre, Helsinki, Finland

166. Research Programs Unit, Diabetes and Obesity, University of Helsinki, Helsinki, Finland

167. Center for Vascular Prevention, Danube University Krems, Krems, Austria

168. Department of Public Health, University of Helsinki, Helsinki, Finland

169. Diabetes Prevention Unit, National Institute for Health and Welfare, Helsinki, Finland

170. Diabetes Research Group, King Abdulaziz University, Jeddah, Saudi Arabia

171. Instituto de Investigacion Sanitaria del Hospital Universario LaPaz (IdiPAZ), University Hospital LaPaz, Autonomous University of Madrid, Madrid, Spain

172. Instituto de Investigaciones Biomédicas, Departamento de Medicina Genómica y ToxicologiÌĄa, Universidad Nacional Autónoma de México, Mexico City, Mexico

173. Department of Nutrition, Harvard School of Public Health, Boston, MA, USA

174. Preventive Medicine and Epidemiology, Medicine, Boston University School of Medicine, Boston, MA, USA

175. Department of Epidemiology, Fairbanks School of Public Health, Indiana University, Indianapolis, IN, USA

176. Department of Medicine, Indiana University School of Medicine, Indianapolis, IN, USA

177. Department of Human Genetics, Wellcome Trust Sanger Institute, Hinxton, Cambridgeshire, UK

178. Department of Physiology and Biophysics, University of Mississippi Medical Center, Jackson, MS, USA

179. Danish Diabetes Academy, Odense, Denmark

180. Department of Public Health, Aarhus University, Aarhus, Denmark

181. Department of Ophthalmology, Yong Loo Lin School of Medicine, National University of Singapore, National University Health System, Singapore

182. Department of Statistics and Applied Probability, National University of Singapore, Singapore

183. Genome Institute of Singapore, Agency for Science, Technology and Research, Singapore

184. Wellcome Trust Sanger Institute, Hinxton, UK

185. Department of Human Genetics, University of Chicago, Chicago, IL, USA

186. Oxford Centre for Diabetes, Endocrinology and Metabolism, University of Oxford, Oxford, UK

187. Seoul National University, Seoul, South Korea

188. McGill Centre for Bioinformatics, McGill University, Montréal, Québec, Canada

189. Genetics of Complex Traits, University of Exeter Medical School, University of Exeter, Exeter, UK
190. Institute for Genomics and Systems Biology, University of Chicago, Chicago, IL, USA
191. Department of Ophthalmology, Erasmus Medical Center, Rotterdam, Netherlands
192. Department of Ophthalmology, National University of Singapore and National University Health System, Singapore
193. The Biostatistics Center, George Washington University, Washington, DC , USA
194. Department of Gene Diagnostics and Therapeutics, Research Institute, National Center for Global Health and Medicine, Tokyo, Japan
195. Department of Health Studies, University of Chicago, Chicago, IL, USA
196. Graduate School for Integrative Science and Engineering, National University of Singapore, Singapore
197. Department of Statistics, University of Oxford, Oxford, UK
198. McGill University, Montréal, Québec, Canada
199. Center for Genomics and Personalized Medicine Research, Wake Forest School of Medicine, Winston-Salem, NC, USA
200. Department of Medicine, Program for Personalized and Genomic Medicine, University of Maryland School of Medicine, Baltimore, MD, USA
201. Department of Medical Sciences, Molecular Epidemiology and Science for Life Laboratory, Uppsala University, Uppsala, Sweden
202. Department of Genetics, Harvard Medical School, Boston, MA, USA
203. Broad Institute of Harvard and MIT, Cambridge, MA, USA
204. National Institute of Diabetes and Digestive and Kidney Disease, National Institutes of Health, Bethesda, MD, USA
205. Department of Genetic Epidemiology, Erasmus Medical Center, Rotterdam, Netherlands
206. Genetic Epidemiology Unit, Department of Epidemiology, Erasmus University Medical Center, Rotterdam, The Netherlands
207. MRC Epidemiology Unit, University of Cambridge School of Clinical Medicine, Institute of Metabolic Science, Cambridge Biomedical Campus, Cambridge, UK
208. Centre for Medical Research, Western Australian Institute for Medical Research, University of Western Australia, Nedlands, Australia
209. Chung-Ang University, Seoul, South Korea
210. Department of Psychiatry, University of Michigan, Ann Arbor, MI, USA
211. Centro de Estudios en Diabetes, Unidad de Investigacion en Diabetes y Riesgo Cardiovascular, Centro de Investigacion en Salud Poblacional, Instituto Nacional de Salud Publica, Mexico City, Mexico

212. Analytic and Translational Genetics Unit, Massachusetts General Hospital, Boston, MA, USA

213. Instituto de Investigaciones Biomédicas, Unidad de BiologiÌĄa Molecular y Medicina Genómica, UNAM/INCMNSZ, Mexico City, Mexico

214. Universidad Autónoma de Nuevo León, San Nicolás de los Garza, Nuevo León, México

215. Epidemiology Program, University of Hawaii Cancer Center, Honolulu, HI, USA

216. Universidad Autonoma Metropolitana, Mexico City, Mexico

217. Instituto de Seguridad y Servicios Sociales para los Trabajadores del Estado, Mexico City, Mexico

218. Genetics of Diabetes, University of Exeter Medical School, University of Exeter, Exeter, UK

219. Department of Biochemistry and Molecular Biology, Pennsylvania State University, State College, PA, USA

220. Department of Biology, University of Copenhagen, Copenhagen, Denmark

221. Department of Statistics, University of California Berkeley, Berkeley, CA, USA

222. Department of Human Nutrition, Faculty of Life Sciences, University of Copenhagen, Copenhagen, Denmark

223. Department of Human Genetics, University of Aarhus, Aarhus, Denmark

224. Faculty of Medicine, University of Aalborg, Aalborg, Denmark

225. Faculty of Health Sciences, University of Aarhus, Aarhus, Denmark

226. Marie Krogh Center for Metabolic Research, Metabolic Receptology and Enteroendocrinology, Faculty of Health Sciences, University of Copenhagen, Copenhagen, Denmark

227. BGI-Shenzhen, Shenzhen, China

228. Kaiser Permanente Southern California, Pasadena, CA, USA

229. Wake Forest School of Medicine, Winston-Salem, NC, USA

230. Children's Hospital of Philadelphia, Philadelphia, PA, USA

231. University of Vermont, Burlington, VT, USA

232. University of Maryland School of Medicine, Baltimore, MD, USA

233. University of Southern California, Los Angeles, CA, USA

234. Washington University School of Medicine, St. Louis, MO, USA

235. Harvard Medical School, Boston, MA, USA

236. University Medical Center Utrecht, Utrecht, Netherlands

237. University of Oxford, Oxford, UK

238. Cleveland Clinic, Cleveland, OH, USA

239. University of Michigan, Ann Arbor, MI, USA

240. Harvard University, Cambridge, MA, USA
241. Montreal Heart Institute, Montréal, QC, Canada
242. Université de Montréal, Montreal, QC, Canada
243. University of Pennsylvania, Philadelphia, PA, USA
244. Institute of Cardiovascular and Medical Sciences, University of Glasgow, Glasgow, UK
245. University of Glasgow School of Medicine, Glasgow, UK
246. Department of Internal Medicine, Erasmus University Medical Center, Rotterdam, The Netherlands
247. Diabetes Unit, Department of Medicine, Massachusetts General Hospital, Boston, MA, USA
248. Research Unit, Skellefteå, Sweden
249. Cardiovascular Health Research Unit, Departments of Medicine and Epidemiology, University of Washington, Seattle, WA, USA
250. New York Academy of Medicine, New York, NY, USA
251. University of Missouri Kansas City, Kansas City, MO, USA
252. Jackson State University, Jackson, MS, USA
253. University of Mississippi Medical Center, Jackson, MS, USA
254. Baylor College of Medicine, Houston, TX, USA
255. Methodist DeBakey Heart Center, Houston, TX, USA
256. University of Texas Health Science Center, San Antonio, TX, USA
257. Department of Medicine, University of Washington, Seattle, WA, USA
258. Harbor-UCLA Medical Center, Torrance, CA, USA
259. University of Virginia, Charlottesville, VA, USA
260. Florida International University, Miami, FL, USA
261. University of Minnesota, Minneapolis, MN, USA
262. Brown Foundation Institute of Molecular Medicine, University of Texas Health Science Center, San Antonio, TX, USA
263. Hackensack University Medical Center, Hackensack, NJ, USA
264. Columbia University Medical Center, New York, NY, USA
265. University of California Los Angeles, Los Angeles, CA, USA
266. University of California San Diego, La Jolla, CA, USA
267. Department of Genetics and Department of Biostatistics, University of North Carolina, Chapel Hill, NC, USA
268. Framingham Heart Study, Framingham, MA, USA

269. Population Sciences Branch, National Heart, Lung, and Blood Institute, National Institutes of Health, Bethesda, MD, USA

270. University of Alabama at Birmingham, Birmingham, AL, USA

271. Northwestern University, Evanston, IL, USA

272. Department of Epidemiology and Prevention, Division of Public Health Sciences, Wake Forest University, Winston-Salem, NC, USA

273. University of Auckland, Aukland, New Zealand

274. University of Pittsburgh, Pittsburgh, PA, USA

275. Tufts University School of Medicine, Boston, MA, USA

276. Columbia University, New York, NY, USA

277. Indiana University School of Medicine, Indianapolis, IN, USA

278. Stanford University School of Medicine, Stanford, CA, USA

279. University of Alabama at Tuscaloosa, Tuscaloosa, AL, USA

280. Kaiser Permanente Division of Research, Oakland, CA, USA

281. Tougaloo College, Tougaloo, MS, USA

282. Institute of Neurology, London, UK

283. Reta Lila Weston Research Laboratories, London, UK

284. University College London, London, UK

285. Mayo Clinic, Rochester, MN, USA

286. Laboratory of Neurogenetics, National Institute on Aging, Bethesda, MD, USA

287. National Institute on Aging, Bethesda, MD, USA

288. Children's Hospital of Michigan, Detroit, MI, USA

289. University of Colorado, Boulder, CO, USA

290. Upstate Medical University, Oneida, NY, USA

291. Rhode Island Hospital, Providence, RI, USA

292. Johns Hopkins University, Baltimore, MD, USA

293. Children's Mercy Hospital, Kansas City, MO, USA

294. Emory University, Atlanta, GA, USA

295. University of Utah, Salt Lake City, UT, USA

296. A.I. Dupont Institute Medical Center, Wilmington, DE, USA

297. National Jewish Health, Denver, CO, USA

298. University of British Columbia, Vancouver, BC, Canada
299. Ochsner Health System, Jefferson Parish, LA, USA
300. Schneider Children's Hospital, Queens, NY, USA
301. New York Medical College, Valhalla, NY, USA
302. Westchester Medical Center, Valhalla, NY, USA
303. Cook Children's Med. Center, Fort Worth, TX, USA
304. St. Louis Children's Hospital, St. Louis, MO, USA
305. Children's Medical Center of Dayton, Dayton, OH, USA
306. Children's Hospital of Wisconsin, Milwaukee, WI, USA
307. All Children's Hospital Cystic Fibrosis Center, St Petersburg, FL, USA
308. Johns Hopkins University School of Public Health, Baltimore, MD, USA
309. Texas Children's Hospital, Houston, TX, USA
310. Indiana University, Indianapolis, IN, USA
311. Riley Hospital for Children, Indianapolis, IN, USA
312. University of Kentucky, Lexington, KY, USA
313. National Human Genome Research Institute, Bethesda, MD, USA
314. Rainbow Babies and Children's Hospital, Cleveland, OH, USA
315. Vermont Children's Hospital at Fletcher Allen Health Care, VT, USA
316. Harvard School of Public Health, Boston, MA, USA
317. Maine Medical Center, Portland, ME, USA
318. VA Puget Sound Medical Center, Seattle, WA, USA
319. The GeneSTAR Research Program, Division of General Internal Medicine, Department of Medicine, Johns Hopkins University School of Medicine, Baltimore, MD, USA
320. Children's Hospitals and Clinics of Minnesota, Minneapolis, MN, USA
321. DeVos Children's Butterworth Hospital, Grand Rapids, MI, USA
322. Spectrum Health Systems, Worcester, MA, USA
323. Stanford University, Stanford, CA, USA
324. Cardinal Glennon Children's Hospital, St. Louis, MO, USA
325. University of Massachusetts Memorial Health Care, MA, USA
326. Children's Hospital of Pittsburgh, Pittsburgh, PA, USA
327. St. Paul's Hospital, Vancouver, BC, Canada

328. Vanderbilt University, Nashville, TN, USA
329. Children's Memorial Hospital, Chicago, IL, USA
330. University of Rochester, Rochester, NY, USA
331. University of Wisconsin Hospital and Clinics, Madison, WI, USA
332. Nemours Children's Clinic, Jacksonville, FL, USA
333. Children's Hospital of Buffalo, Buffalo, NY, USA
334. Elliot Health System, Manchester, NH, USA
335. St. Christopher's Hospital for Children, Philadelphia, PA, USA
336. Dartmouth-Hitchcock Medical Center, Lebanon, NH, USA
337. New Hampshire Cystic Fibrosis Center, Nashua, NH, USA
338. Monmouth Medical Center, Long Branch, NJ, USA
339. Puget Sound Blood Center, Seattle, WA, USA
340. Adaptive Biotechnologies Corporation, Seattle, WA, USA
341. University of California Irvine, Irvine, CA, USA
342. Rush Medical Center, Chicago, IL, USA
343. University of Nevada, Reno, NV, USA
344. Los Angeles Biomedical Research Institute, Los Angeles, CA, USA
345. University of Hawaii, Honolulu, HI, USA
346. Brown University, Providence, RI, USA
347. Memorial Hospital of Rhode Island, Pawtucket, RI, USA
348. University of Cincinnati, Cincinnati, OH, USA
349. Howard University, Washington, DC, USA
350. MedStar Research Institute, Hyattsville, MD, USA
351. Ohio State University, Columbus, OH, USA
352. University of Miami, Coral Gables, FL, USA
353. University of Tennessee Health Science Center, Memphis, TN, USA
354. State University of New York at Stony Brook, Stony Brook, NY, USA
355. University of Medicine and Dentistry of New Jersey, Newark, NJ, USA
356. Kaiser Permanente Center for Health Research, Portland, OR, USA
357. University of Florida, Gainesville, FL, USA

358. George Washington University Medical Center, Washington, DC, USA
359. Amgen Inc., Newbury Park, CA, USA
360. Medical College of Wisconsin, Wauwatosa, WI, USA
361. Fallon Clinic, Worcester, MA, USA
362. University of Massachusetts, Amherst, MA, USA
363. University of California Davis, Davis, CA, USA
364. University of Iowa, Iowa City, IA, USA
365. University of Wisconsin, Madison, WI, USA
366. Wayne State University, Detroit, MI, USA
367. Medpace Reference Laboratories, Cincinnati, OH, USA
368. University of Louisville, Louisville, KY, USA
369. University of Arizona, Tucson, AZ, USA
370. University of Buffalo, Buffalo, NY, USA
371. National Center for Biotechnology Information, Bethesda, MD, USA
372. Department of Medicine and Department of Biomedical Sciences, Cedars-Sinai Medical Center, Los Angeles, CA, USA
373. Materials and Process Simulation Center, California Institute of Technology, Pasadena, CA, USA
374. Division of Statistical Genomics and Department of Genetics, Washington University School of Medicine, St. Louis, MO, USA
375. Genetics, PCPS, GlaxoSmithKline, RTP, NC, USA
376. Department of Preventive Medicine, Northwestern University Feinberg School of Medicine, Chicago, IL, USA
377. Division of Genetics and Cell Biology, San Raffaele Research institute, Milano, Italy
378. CEA, Institut de Génomique, Centre National de Génotypage, Cedex, France
379. McKusick-Nathans Institute of Genetic Medicine, Johns Hopkins University School of Medicine, Baltimore, MD, USA
380. Predoctoral Training Program in Human Genetics, McKusick-Nathans Institute of Genetic Medicine, Johns Hopkins University School of Medicine, MD, USA
381. Department of Epidemiology, German Institute of Human Nutrition Potsdam RehbruÌĹcke, Nuthetal, Germany
382. Section of Biology and Genetics, Department of Life and Reproduction Sciences, University of Verona, Verona, Italy
383. Department of Genome Dynamics, Lawrence Berkeley National Laboratory, Berkeley, CA, USA

384. Division of Preventive Medicine, Brigham and Women's Hospital, Boston, MA, USA

385. Harokopio University, Athens, Greece

386. Department of Epidemiology, Erasmus University Medical Center, Rotterdam, The Netherlands

387. Princess Al-Jawhara Al-Brahim Centre of Excellence in Research of Hereditary Disorders (PACER-HD), King Abdulaziz University, Jeddah, Saudi Arabia

388. William Harvey Research Institute, Barts and The London School of Medicine and Dentistry, Queen Mary University of London, UK

389. Statistical Genetics, PCPS, GlaxoSmithKline, RTP, NC, USA

390. Division of Cardiology, Geneva University Hospital, Geneva, Switzerland

391. McKusick-Nathans Institute of Genetic Medicine, Johns Hopkins University, Baltimore, MD, USA

392. Icelandic Heart Association, 201 Kopavogur, Iceland

393. Department of Clinical Sciences, Genetic and Molecular Epidemiology Unit, Skåne University Hospital, Malmö, Sweden

394. Department of Nutrition and Dietetics, School of Health Science and Education, Harokopio University, Athens, Greece

395. Department of Clinical and Experimental Medicine, University of Pisa School of Medicine, Pisa, Italy

396. Department of Genome Sciences, University of Washington, Seattle, WA, USA

397. Department of Public Health and Clinical Medicine, Umeå University, Umeå, Sweden

398. Atherosclerosis Research Unit, Department of Medicine Solna, Karolinska Institutet, Stockholm, Sweden

399. Department of Numerical Analysis and Computer Science, SciLifeLab, Stockholm University, Stockholm, Sweden

400. Department of Public Health and Primary Care, Strangeways Research Laboratory, University of Cambridge, Cambridge, UK

401. Division of Nephrology, Department of Internal Medicine and Medical Specialties, Columbus-Gemelli University Hospital, Catholic University, Rome, Italy

402. Department of Cardiovascular Medicine, Wellcome Trust Centre for Human Genetics, University of Oxford, Oxford, UK

403. University of Iceland, 101 Reykjavik, Iceland

404. Department of Biobank Research, Umeå University, Umeå, Sweden

405. MRC Human Genetics Unit, MRC IGMM, University of Edinburgh, Edinburgh, Scotland

406. Department of Biomedical Technology, Sangmyung University, Chungnam, Korea

407. Section on Statistical Genetics, Department of Biostatistics, University of Alabama at Birmingham, Birmingham, AL, USA

408. Memory Aging and Cognition Centre (MACC), National University Health System, Singapore

409. Echinos Medical Centre, Echinos, Greece

410. Division of Research, Kaiser Permanente, Northern California Region, Oakland, CA, USA

411. Division of Human Genetics, Genome Institute of Singapore, Singapore

412. Fairbanks School of Public Health, Indiana University, Indianapolis, IN, USA

413. Division of Endocrine and Metabolism, Department of Internal Medicine, Taichung Veterans General Hospital, Taichung, Taiwan

414. Department of Medical Research, Taichung Veterans General Hospital, Taichung, Taiwan

415. Department of Epidemiology, Johns Hopkins University, Baltimore, MD, USA

416. Department of Medicine, Johns Hopkins University, Baltimore, MD, USA

417. Center for Molecular Medicine and Genetics, Wayne State University, Detroit, MI, USA

418. Department of Neurology, Wayne State University School of Medicine, Detroit, MI, USA

419. Division of Cardiology, Brigham and Women's Hospital, Boston, MA, USA

420. Division of Cardiology, Harvard Medical School, Boston, MA, USA

421. National and Kapodistrian University of Athens, Dromokaiteio Psychiatric Hospital, Athens, Greece

422. University of Athens, Department of Dietetics and Nutritional Science, Harokopio University, Athens, Greece

423. Division of General Internal Medicine, Johns Hopkins University School of Medicine, Baltimore, MD, USA

424. Welch Center for Prevention, Epidemiology, and Clinical Research, Johns Hopkins University, Baltimore, MD, USA

425. USC Eye Institute, Department of Ophthalmology, Keck School of Medicine of the University of Southern California, Los Angeles, CA, USA

426. Department of Molecular Epidemiology, German Institute of Human Nutrition Potsdam-RehbruÌĹcke, Nuthetal, Germany

427. Department of Epidemiology, Colorado School of Public Health, University of Colorado Denver, Aurora, CO, USA

428. Department of Nutrition and Dietetics, Harokopio University, Athens, Greece

429. University of Cambridge Metabolic Research Laboratories, MRC Metabolic Diseases Unit and NIHR Cambridge Biomedical Research Centre, Wellcome Trust-MRC Institute of Metabolic Science, Addenbrooke's Hospital, Cambridge, UK

430. Department of Internal Medicine, Division of Endocrinology, Metabolism, Pathobiochemistry and Clinical Chemistry and Institute of Diabetes Research and Metabolic Diseases, University of TuÌĹbingen, TuÌĹbingen, Germany

431. German Center for Diabetes Research (DZD), Germany

432. The Netherlands Genomics Initiative-sponsored Netherlands Consortium for Healthy Aging (NGI-NCHA), Leiden/Rotterdam, the Netherlands

433. Department of Public Health, Faculty of Medicine, University of Split, Split, Croatia

434. The Oxford Centre for Diabetes, Endocrinology and Metabolism, University of Oxford, Oxford, UK

435. Wellcome Trust Centre for Human Genetics, Oxford, UK

436. Centre for Population Health Sciences, Medical School, University of Edinburgh, Edinburgh, Scotland

437. Generation Scotland, A Collaboration between the University Medical Schools and NHS, Aberdeen, Dundee, Edinburgh, and Glasgow, UK

438. Science for Life Laboratory, Karolinska Institutet, Stockholm, Sweden

439. Department of Gastroenterology, Gennimatas General Hospital, Athens, Greece

440. College of Medicine, National Defense Medical Center, Taipei, Taiwan

441. School of Medicine, National Yang-Ming University, Taipei, Taiwan

442. Department of Epidemiology, School of Public Health, University of Michigan, Ann Arbor, MI, USA

443. Medical Research Institute, University of Dundee, Dundee, UK

444. Department of Hematology, Long Road, Cambridge, UK

445. Department of Internal Medicine, Division of Gastroenterology and Department of Computational Medicine and Bioinformatics, University of Michigan, Ann Arbor, MI, USA

446. Division of Psychiatric Genomics, Icahn School of Medicine at Mount Sinai, New York, NY, USA

447. First Department of Propaedeutic and Internal Medicine, Athens University Medical School, Laiko General Hospital, Athens, Greece

448. Diabetes Centre, 2nd Department of Internal Medicine, National University of Athens, Hippokration General Hospital, Athens, Greece

449. Anogia Medical Centre, Anogia, Greece

450. Center for Medical Systems Biology, Leiden, The Netherlands

451. Department of Clinical Sciences, Genetic and Molecular Epidemiology Unit, Lund University, Skåne University Hospital, Malmö, Sweden

452. Division of Public Health Sciences, Wake Forest School of Medicine, Winston-Salem, NC, USA

453. Institute of Cellular Medicine, Newcastle University, Newcastle-upon-Tyne, UK

454. Dromokaiteio Psychiatric Hospital, Athens, Greece

455. University of Sheffield, Sheffield, UK

# References

[1] Fuchsberger, C. *et al.* The genetic architecture of type 2 diabetes. *Nature* **536**, 41–7 (2016).

[2] Consortium, S. T. . D. *et al.* Sequence variants in slc16a11 are a common risk factor for type 2 diabetes in mexico. *Nature* **506**, 97–101 (2014).

[3] Fu, W. *et al.* Analysis of 6,515 exomes reveals the recent origin of most human protein-coding variants. *Nature* **493**, 216–20 (2013).

[4] Lohmueller, K. E. *et al.* Whole-exome sequencing of 2,000 danish individuals and the role of rare coding variants in type 2 diabetes. *Am J Hum Genet* **93**, 1072–86 (2013).

[5] Fisher, S. *et al.* A scalable, fully automated process for construction of sequence-ready human exome targeted capture libraries. *Genome Biol* **12**, R1 (2011).

[6] Li, H. & Durbin, R. Fast and accurate short read alignment with burrows-wheeler transform. *Bioinformatics* **25**, 1754–60 (2009).

[7] DePristo, M. A. *et al.* A framework for variation discovery and genotyping using next-generation dna sequencing data. *Nat Genet* **43**, 491–8 (2011).

[8] Purcell, S. *et al.* Plink: a tool set for whole-genome association and population-based linkage analyses. *Am J Hum Genet* **81**, 559–75 (2007).

[9] Price, A. L. *et al.* Principal components analysis corrects for stratification in genome-wide association studies. *Nat Genet* **38**, 904–9 (2006).

[10] Mathieson, I. & McVean, G. Differential confounding of rare and common variants in spatially structured populations. *Nat. Genet.* **44**, 243–246 (2012).

[11] McLaren, W. *et al.* The ensembl variant effect predictor. *Genome Biol* **17**, 122 (2016).

[12] Aken, B. L. *et al.* Ensembl 2017. *Nucleic Acids Res* **45**, D635–D642 (2017).

[13] Pujar, S. *et al.* Consensus coding sequence (ccds) database: a standardized set of human and mouse protein-coding regions supported by expert curation. *Nucleic Acids Res* (2017).

[14] Liu, X., Wu, C., Li, C. & Boerwinkle, E. dbnsfp v3.0: a one-stop database of functional predictions and annotations for human nonsynonymous and splice-site snvs. *Hum Mutat* **37**, 235–41 (2016).

[15] Jagadeesh, K. A. *et al.* M-cap eliminates a majority of variants of uncertain significance in clinical exomes at high sensitivity. *Nat Genet* **48**, 1581–6 (2016).

[16] Skol, A. D., Scott, L. J., Abecasis, G. R. & Boehnke, M. Optimal designs for two-stage genome-wide association studies. *Genet Epidemiol* **31**, 776–88 (2007).

[17] Kang, H. M. *et al.* Variance component model to account for sample structure in genome-wide association studies. *Nat Genet* **42**, 348–54 (2010).

[18] Ma, C., Blackwell, T., Boehnke, M., Scott, L. J. & Go, T. D. i. Recommended joint and meta-analysis strategies for case-control association testing of single low-count variants. *Genet Epidemiol* **37**, 539–50 (2013).

[19] Willer, C. J., Li, Y. & Abecasis, G. R. Metal: fast and efficient meta-analysis of genomewide association scans. *Bioinformatics* **26**, 2190–1 (2010).

[20] Consortium, S. T. . D. *et al.* Association of a low-frequency variant in hnf1a with type 2 diabetes in a latino population. *JAMA* **311**, 2305–14 (2014).

[21] Zuk, O. *et al.* Searching for missing heritability: designing rare variant association studies. *Proc Natl Acad Sci U S A* **111**, E455–64 (2014).

[22] Wu, M. C. *et al.* Rare-variant association testing for sequencing data with the sequence kernel association test. *Am J Hum Genet* **89**, 82–93 (2011).

[23] Lee, S. *et al.* Optimal unified approach for rare-variant association testing with application to small-sample case-control whole-exome sequencing studies. *Am J Hum Genet* **91**, 224–37 (2012).

[24] Li, M. X., Gui, H. S., Kwan, J. S. & Sham, P. C. Gates: a rapid and powerful gene-based association test using extended simes procedure. *Am J Hum Genet* **88**, 283–93 (2011).

[25] Han, B., Kang, H. M. & Eskin, E. Rapid and accurate multiple testing correction and power estimation for millions of correlated markers. *PLoS Genet* **5**, e1000456 (2009).

[26] Conneely, K. N. & Boehnke, M. So many correlated tests, so little time! rapid adjustment of p values for multiple correlated tests. *Am J Hum Genet* **81**, 1158–68 (2007).

[27] Mahajan, A. *et al.* Identification and functional characterization of G6PC2 coding variants influencing glycemic traits define an effector transcript at the G6PC2-ABCB11 locus. *PLoS Genet.* **11**, e1004876 (2015).

[28] Mahajan, A. *et al.* Refining the accuracy of validated target identification through coding variant fine-mapping in type 2 diabetes. *Nat Genet* **50**, 559–571 (2018).

[29] Chambers, J. C. *et al.* Common genetic variation near mc4r is associated with waist circumference and insulin resistance. *Nat Genet* **40**, 716–8 (2008).

[30] Morris, A. P. *et al.* Large-scale association analysis provides insights into the genetic architecture and pathophysiology of type 2 diabetes. *Nat Genet* **44**, 981–90 (2012).

[31] Raimondo, A. *et al.* Type 2 diabetes risk alleles reveal a role for peptidylglycine alpha-amidating monooxygenase in beta cell function. *bioRxiv* (2017).

[32] Dewey, F. E. *et al.* Distribution and clinical impact of functional variants in 50,726 whole-exome sequences from the discovehr study. *Science* **354** (2016).

[33] Psaty, B. M. *et al.* Cohorts for heart and aging research in genomic epidemiology (charge) consortium: Design of prospective meta-analyses of genome-wide association studies from 5 cohorts. *Circ Cardiovasc Genet* **2**, 73–80 (2009).

[34] Yu, B. *et al.* Rare exome sequence variants in clcn6 reduce blood pressure levels and hypertension risk. *Circ Cardiovasc Genet* **9**, 64–70 (2016).

[35] Brody, J. A. *et al.* Analysis commons, a team approach to discovery in a big-data environment for genetic epidemiology. *Nat Genet* **49**, 1560–3 (2017).

[36] Chen, H. *et al.* Control for population structure and relatedness for binary traits in genetic association studies via logistic mixed models. *Am J Hum Genet* **98**, 653–66 (2016).

[37] The amp-t2d knowledge portal.

[38] Ramatenki, V. *et al.* Identification of New Lead Molecules Against UBE2NL Enzyme for Cancer Therapy. *Appl. Biochem. Biotechnol.* **182**, 1497–1517 (2017).

[39] Gomez-Ramos, A., Podlesniy, P., Soriano, E. & Avila, J. Distinct X-chromosome SNVs from some sporadic AD samples. *Sci Rep* **5**, 18012 (2015).

[40] Jiang, Y. *et al.* Six novel rare non-synonymous mutations for migraine without aura identified by exome sequencing. *J. Neurogenet.* **29**, 188–194 (2015).

[41] Wishart, D. S. *et al.* Drugbank 5.0: a major update to the drugbank database for 2018. *Nucleic Acids Res* (2017).

[42] Haghverdizadeh, P., Sadat Haerian, M., Haghverdizadeh, P. & Sadat Haerian, B. Abcc8 genetic variants and risk of diabetes mellitus. *Gene* **545**, 198–204 (2014).

[43] Torres, J. M. *et al.* Integrative cross tissue analysis of gene expression identifies novel type 2 diabetes genes. *bioRxiv* (2017).

[44] Barbeira, A. N. *et al.* Exploring the phenotypic consequences of tissue specific gene expression variation inferred from gwas summary statistics. *bioRxiv* (2017).

[45] Mahajan, A. *et al.* Genome-wide trans-ancestry meta-analysis provides insight into the genetic architecture of type 2 diabetes susceptibility. *Nat Genet* **46**, 234–44 (2014).

[46] Flannick, J. & Florez, J. C. Type 2 diabetes: genetic data sharing to advance complex disease research. *Nat Rev Genet* **17**, 535–49 (2016).

[47] Rossin, E. J. *et al.* Proteins encoded in genomic regions associated with immune-mediated disease physically interact and suggest underlying biology. *PLoS Genet* **7**, e1001273 (2011).

[48] Thomsen, S. K. *et al.* Systematic functional characterization of candidate causal genes for type 2 diabetes risk variants. *Diabetes* **65**, 3805–11 (2016).

[49] Gaulton, K. J. *et al.* Genetic fine mapping and genomic annotation defines causal mechanisms at type 2 diabetes susceptibility loci. *Nat Genet* **47**, 1415–25 (2015).

[50] Mahajan, A. *et al.* Fine-mapping of an expanded set of type 2 diabetes loci to single-variant resolution using high-density imputation and islet-specific epigenome maps. *bioRxiv* (2018).

[51] Grotz, A. K., Gloyn, A. L. & Thomsen, S. K. Prioritising causal genes at type 2 diabetes risk loci. *Curr Diab Rep* **17**, 76 (2017).

[52] Das, S. *et al.* Next-generation genotype imputation service and methods. *Nat Genet* **48**, 1284–7 (2016).

[53] Consortium, . G. P. *et al.* A global reference for human genetic variation. *Nature* **526**, 68–74 (2015).

[54] McCarthy, S. *et al.* A reference panel of 64,976 haplotypes for genotype imputation. *Nat Genet* **48**, 1279–83 (2016).

[55] Segre, A. V. *et al.* Common inherited variation in mitochondrial genes is not enriched for associations with type 2 diabetes or related glycemic traits. *PLoS Genet* **6** (2010).

[56] So, H. C., Gui, A. H., Cherny, S. S. & Sham, P. C. Evaluating the heritability explained by known susceptibility variants: a survey of ten complex diseases. *Genet Epidemiol* **35**, 310–7 (2011).

[57] Goldstein, D. B. Common genetic variation and human traits. *N Engl J Med* **360**, 1696–8 (2009).

[58] Brown, L. D., Cai, T. & Dasgupta, A. Interval estimation for a binomial proportion. *Statistical Science* **16**, 101–133 (2001).

[59] Pickrell, J. K. Joint analysis of functional genomic data and genome-wide association studies of 18 human traits. *Am J Hum Genet* **94**, 559–73 (2014).

[60] Finucane, H. K. *et al.* Partitioning heritability by functional annotation using genome-wide association summary statistics. *Nat Genet* **47**, 1228–35 (2015).

[61] Stephens, M. & Balding, D. J. Bayesian statistical methods for genetic association studies. *Nat Rev Genet* **10**, 681–90 (2009).

[62] Boyle, E. A., Li, Y. I. & Pritchard, J. K. An expanded view of complex traits: from polygenic to omnigenic. *Cell* **169**, 1177–86 (2017).

[63] Zhang, S. D. Towards accurate estimation of the proportion of true null hypotheses in multiple testing. *PLoS ONE* **6**, e18874 (2011).

[64] Pounds, S. & Morris, S. W. Estimating the occurrence of false positives and false negatives in microarray studies by approximating and partitioning the empirical distribution of p-values. *Bioinformatics* **19**, 1236–1242 (2003).

[65] Eilbeck, K. *et al.* The Sequence Ontology: a tool for the unification of genome annotations. *Genome Biol.* **6**, R44 (2005).

[66] Landrum, M. J. *et al.* ClinVar: public archive of relationships among sequence variation and human phenotype. *Nucleic Acids Res.* **42**, D980–985 (2014).

[67] Majithia, A. R. *et al.* Prospective functional classification of all possible missense variants in pparg. *Nat Genet* **48**, 1570–1575 (2016).
