# Supplementary material for: Exome sequencing of 20,791 cases of type 2 diabetes and 24,440 controls
Source: Nature. 2019 May 22;570(7759):71–6. doi: 10.1038/s41586-019-1231-2 (PMC6699738; doi:10.1038/s41586-019-1231-2)
Supplement: Supplementary file 2 — Reporting Summary [file 41586_2019_1231_MOESM2_ESM.pdf]

nature research

Corresponding author(s): Jason Flannick

Last updated by author(s): Mar 5, 2019

Reporting Summary

Nature Research wishes to improve the reproducibility of the work that we publish. This form provides structure for consistency and transparency in reporting. For further information on Nature Research policies, see Authors & Referees and the Editorial Policy Checklist.

Statistics

For all statistical analyses, confirm that the following items are present in the figure legend, table legend, main text, or Methods section.

n/a	Confirmed	
☐	☒	The exact sample size (*n*) for each experimental group/condition, given as a discrete number and unit of measurement
☐	☒	A statement on whether measurements were taken from distinct samples or whether the same sample was measured repeatedly
☐	☒	The statistical test(s) used AND whether they are one- or two-sided<br>*Only common tests should be described solely by name; describe more complex techniques in the Methods section.*
☐	☒	A description of all covariates tested
☐	☒	A description of any assumptions or corrections, such as tests of normality and adjustment for multiple comparisons
☐	☒	A full description of the statistical parameters including central tendency (e.g. means) or other basic estimates (e.g. regression coefficient) AND variation (e.g. standard deviation) or associated estimates of uncertainty (e.g. confidence intervals)
☐	☒	For null hypothesis testing, the test statistic (e.g. *F*, *t*, *r*) with confidence intervals, effect sizes, degrees of freedom and *P* value noted<br>*Give P values as exact values whenever suitable.*
☐	☒	For Bayesian analysis, information on the choice of priors and Markov chain Monte Carlo settings
☒	☐	For hierarchical and complex designs, identification of the appropriate level for tests and full reporting of outcomes
☐	☒	Estimates of effect sizes (e.g. Cohen's *d*, Pearson's *r*), indicating how they were calculated

Our web collection on statistics for biologists contains articles on many of the points above.

Software and code

Policy information about availability of computer code

Data collection	No software was used in data collection
Data analysis	All software used in the analysis was open source and is described in the Methods section of the manuscript. Existing software packages used were: RTA v2.7.3, BWA v0.7 , Picard v1, GATK v3.4, VEP v87, Plink 1.9, EPACTS v3.2.4, MetaXcan v0.3, DAPPLE, MAGENTA v2.4, R v3.4, Michigan Imputation Server. Custom scripts (available for download as a zip file) were written to conduct the minimum p-value test, perform the Wilcoxon rank sum test for gene sets, and estimate the fraction of true associations as a function of variant p-value.

For manuscripts utilizing custom algorithms or software that are central to the research but not yet described in published literature, software must be made available to editors/reviewers. We strongly encourage code deposition in a community repository (e.g. GitHub). See the Nature Research guidelines for submitting code & software for further information.

Data

Policy information about availability of data

All manuscripts must include a data availability statement. This statement should provide the following information, where applicable:

- Accession codes, unique identifiers, or web links for publicly available datasets
- A list of figures that have associated raw data
- A description of any restrictions on data availability

Sequence data and phenotypes for this study are available via the database of Genotypes and Phenotypes (dbGAP) and/or the European Genome-phenome Archive, as indicated in Supplementary Table 1.

October 2018

Field-specific reporting

Please select the one below that is the best fit for your research. If you are not sure, read the appropriate sections before making your selection.

☒ Life sciences ☐ Behavioural & social sciences ☐ Ecological, evolutionary & environmental sciences

For a reference copy of the document with all sections, see nature.com/documents/nr-reporting-summary-flat.pdf

Life sciences study design

All studies must disclose on these points even when the disclosure is negative.

Sample size	We conducted a power analysis (described in the text) to demonstrate that the 45,231 samples analyzed in the study provided a significant increase in power to detect rare variant associations compared to previous studies. Power calculations were performed for frequency and effect size combinations in the range of those previously hypothesized to exist for complex diseases like T2D.
Data exclusions	At the time of sample selection for sequencing, samples were excluded if they matched predetermined criteria for T1D or MODY as described in Supplementary Table 1. At the analysis stage, excluded data were of three types. (a) Samples and (b) variants were excluded if they failed quality control analyses (described in Methods). (c) ~3600 cases from the PRODiGY study were excluded because they did not have suitably matched controls, resulting in inflated tests statistics as described in the Methods section. Exclusion criteria during the analysis stage were determined based on inspection of the distribution of data.
Replication	We replicated our significant associations in independent datasets from CHARGE and GHS, as described in the main text. All three exome-wide significant associations were replicated.
Randomization	Samples were allocated according to the cohort in which they were collected. Further control for confounding factors (imprecise ancestry matching even within cohort, technical confounders) were controlled for by including covariates in the regression model used for association analysis
Blinding	As our analysis involved a regression of phenotype on genotype, neither of which can be influenced by the analyst or data collector, blinding was not relevant to our study

Reporting for specific materials, systems and methods

We require information from authors about some types of materials, experimental systems and methods used in many studies. Here, indicate whether each material, system or method listed is relevant to your study. If you are not sure if a list item applies to your research, read the appropriate section before selecting a response.

Materials & experimental systems

n/a	Involved in the study	
☒	☐	Antibodies
☒	☐	Eukaryotic cell lines
☒	☐	Palaeontology
☒	☐	Animals and other organisms
☐	☒	Human research participants
☒	☐	Clinical data

Methods

n/a	Involved in the study	
☒	☐	ChIP-seq
☒	☐	Flow cytometry
☒	☐	MRI-based neuroimaging

Human research participants

Policy information about studies involving human research participants

Population characteristics	Population characteristics are described in Supplementary Table 1. Relevant characteristics include age, sex, and BMI. For most cohorts, patients with T2D had higher BMI and age, except for cohorts from the GoT2D study where lean, young cases and old, obese controls were preferentially selected. Gender distribution varied by cohort.
Recruitment	Patients were recruited originally as a part of numerous cohort studies, described in Supplementary Table 1, each of which had different selection criteria. For most cohorts, patients were recruited over a long period of time and then cases and controls were selected for sequencing based on DNA and phenotyping quality. T2D diagnosis was determined by clinical data and not the participants themselves. Some bias may have occurred in terms of patient response to recruitment but these are unlikely to be correlated with genotype or have a significant effect on our analysis.
Ethics oversight	All samples were approved for use by their home institution’s institutional review board or ethics committee, as previously reported (see references in the Methods section of the manuscript). Samples newly sequenced at The Broad Institute as part of T2D-GENES, SIGMA, and ProDiGY are covered under Partners Human Research Committee protocol # 2017P000445/PHS “Diabetes Genetics and Related Traits”.

Note that full information on the approval of the study protocol must also be provided in the manuscript.
